# Improvements in hematologic markers and decreases in fatigue with pegcetacoplan for patients with paroxysmal nocturnal hemoglobinuria and mild or moderate anemia (hemoglobin ≥10 g/dL) who had received eculizumab or were naive to complement inhibitors

**DOI:** 10.1371/journal.pone.0306407

**Published:** 2024-07-29

**Authors:** Jens Panse, Nicolas Daguindau, Sonia Okuyama, Régis Peffault de Latour, Philippe Schafhausen, Nicole Straetmans, Mohammed Al-Adhami, Emmelie Persson, Raymond Siu Ming Wong

**Affiliations:** 1 Department of Hematology, Oncology, Hemostaseology and Stem Cell Transplantation, University Hospital RWTH Aachen, Aachen, Germany; 2 Center for Integrated Oncology (CIO), Dusseldorf (ABCD), Aachen, Bonn, Cologne, Germany; 3 Centre Hospitalier Annecy Genevois, Épagny-Metz-Tessy, France; 4 Hematology/Oncology Division, Denver Health and Hospital Authority, Denver, CO, United States of America; 5 French Reference Center for Aplastic Anemia and Paroxysmal Nocturnal Hemoglobinuria, Assistance Publique—Hôpitaux de Paris, Université de Paris, Paris, France; 6 Department of Oncology, Hematology and Bone Marrow Transplantation with Section of Pneumology, University Medical Center Hamburg-Eppendorf, Hamburg, Germany; 7 Department of Hematology, Cliniques Universitaires Saint-Luc, Woluwe-Saint-Lambert, Belgium; 8 Apellis Pharmaceuticals, Waltham, MA, United States of America; 9 Swedish Orphan Biovitrum AB, Stockholm, Sweden; 10 Sir Y.K. Pao Centre for Cancer & Department of Medicine and Therapeutics, Prince of Wales Hospital, The Chinese University of Hong Kong, Hong Kong, China; Bursa Ali Osman Sonmez Oncology Hospital, TURKEY

## Abstract

**Background:**

Although complement component 5 inhibitors (C5is) eculizumab and ravulizumab improve paroxysmal nocturnal hemoglobinuria (PNH) outcomes, patients may experience persistent anemia. This post hoc analysis investigated whether the complement component 3-targeted therapy pegcetacoplan also improved hematologic outcomes and reduced fatigue in patients with PNH and mild/moderate anemia.

**Methods:**

Patients with PNH and hemoglobin ≥10.0 g/dL at baseline of PADDOCK (N = 6), PRINCE (N = 8), and PEGASUS (N = 11) were included. Before receiving pegcetacoplan, PADDOCK and PRINCE patients were C5i-naive; PEGASUS patients had hemoglobin <10.5 g/dL despite stably dosed eculizumab. Hemoglobin concentrations, percentages of patients with concentrations ≥12 g/dL, and sex-specific normalization were assessed at baseline and after 16 weeks of pegcetacoplan, as were absolute reticulocyte counts (ARCs) and normalization and fatigue scores and normalization.

**Results:**

From baseline to week 16, mean (SD) hemoglobin concentrations increased in C5i-naive patients (PADDOCK: 10.5 [0.4] to 12.7 [1.1] g/dL; PRINCE: 11.3 [1.0] to 14.0 [1.3] g/dL) and those with suboptimal eculizumab responses (PEGASUS: 10.2 [0.2] to 12.8 [2.6] g/dL). Percentage of patients with hemoglobin ≥12 g/dL increased (PADDOCK: 0 to 60.0% [3 of 5 patients]; PRINCE: 25.0% [2 of 8] to 87.5% [7 of 8]; PEGASUS: 0 to 72.7% [8 of 11]). Sex-specific hemoglobin normalization at week 16 occurred in 40.0% (2 of 5) (PADDOCK), 62.5% (5 of 8) (PRINCE), and 63.6% (7 of 11) (PEGASUS). In all studies, mean ARCs decreased from above normal to normal and ARC normalization increased. Mean Functional Assessment of Chronic Illness Therapy–Fatigue scores improved from below to above or near normal. Two patients had serious adverse events (PEGASUS: post-surgery sepsis, breakthrough hemolysis); breakthrough hemolysis resolved without study discontinuation.

**Conclusion:**

Patients with PNH and mild/moderate anemia who were C5i-naive or who had suboptimal hemoglobin concentrations despite eculizumab treatment had improved hematologic outcomes and reduced fatigue after initiating or switching to pegcetacoplan.

**Trial registration:**

**Trial registration numbers:** PADDOCK (NCT02588833), PRINCE (NCT04085601; EudraCT, 2018-004220-11), PEGASUS (NCT03500549).

## Introduction

Paroxysmal nocturnal hemoglobinuria (PNH) is a rare, acquired disease that leads to thrombophilia, bone marrow failure, and complement-mediated intravascular hemolysis (IVH) in untreated patients [1,2]. In patients with PNH, nonmalignant clonal expansion of hematopoietic stem cells with an acquired mutation in the *PIG-A* gene disrupts the function of complement regulatory proteins CD55 and CD59, which makes descendant PNH red blood cells susceptible to lysis by the complement system and results in hemolysis and anemia [3–5]. Chronic hemolysis and persistent anemia in patients with PNH cause fatigue [6,7], decrease quality of life (QoL) [7,8], and increase the risk of end organ damage [7,9–13].

The introduction of eculizumab, the first complement component 5 inhibitor (C5i), dramatically improved the disease course for patients with PNH and changed the treatment landscape [1]. Subsequently, a second generation C5i, ravulizumab, also has been approved. However, a significant number of patients with PNH who receive C5is, which control IVH, become susceptible to complement component 3 (C3)–mediated extravascular hemolysis (EVH) that occurs as C3 fragments increase the opsonization of PNH red blood cells, targeting them for phagocytosis in the liver and spleen [14,15]. As a result, C5i-treated patients can experience chronic, low-grade hemolysis, resulting in ongoing anemia, persistent fatigue, and transfusion dependence [16,17]. The average hemoglobin concentration of patients with PNH who receive C5is is less than 10.5 g/dL due to ongoing IVH and emerging EVH [16,18–20]. Proximal complement inhibition provides comprehensive control of the hemolysis (IVH and EVH) of PNH, as shown by transfusion independence and improved hemoglobin concentrations, with the possibility of hemoglobin normalization [3,21–24].

Pegcetacoplan is the first targeted C3 therapy approved in the European Union for adult patients with PNH who have anemia after treatment with a C5i for at least 3 months and in the United States for adults with PNH [25,26]. With the approval of pegcetacoplan for use in patients with PNH who have had an adequate response to or who are tolerant of C5is, it is important to evaluate the efficacy of pegcetacoplan in patients with milder anemia, regardless of the C5i treatment experience and response.

As a targeted C3 therapy, pegcetacoplan blocks multiple parts of the complement pathway by binding to and inhibiting activation of C3 and the C3b subunit, thereby inhibiting C3 and C5 convertases [24,27]. Pegcetacoplan has the potential to normalize hematologic parameters, such as hemoglobin concentrations, absolute reticulocyte counts (ARCs), and lactate dehydrogenase (LDH) concentrations, and to decrease fatigue [22,23]. Thereby, pegcetacoplan is able to improve clinical status and QoL for patients with PNH. This post hoc analysis investigated whether pegcetacoplan can improve or even normalize hematologic parameters and reduce fatigue in patients with PNH and mild or moderate anemia (ie, hemoglobin concentrations ≥10.0 g/dL).

## Methods

### Source clinical trials

This was a post hoc analysis of the first 16 weeks of pegcetacoplan monotherapy in a subgroup of patients with PNH and mild or moderate anemia who participated in the PADDOCK (NCT02588833) [21], PRINCE (NCT04085601; EudraCT, 2018-004220-11) [22], or PEGASUS (NCT03500549) [23,24] clinical trials (**Table 1**). In brief, PADDOCK was a phase 1b, multiple ascending dose pilot trial that included only complement inhibitor–naive patients [21]. Patients in PADDOCK self-administered pegcetacoplan subcutaneously (SC) at a dosage of up to 360 mg/d. The PRINCE trial was a 26-week, phase 3, randomized, controlled study of pegcetacoplan versus supportive care in complement inhibitor–naive patients with hemoglobin concentrations below the sex-specific lower limit of normal (LLN) (12.0 g/dL for female patients, 13.6 g/dL for male patients) that was conducted before the approval of C3 therapy in countries where C5is were not widely available [22]. Patients in PRINCE were randomized 2:1 to self-administer SC injections of pegcetacoplan 1080 mg twice weekly or to continue supportive care only (control) for 26 weeks [22]. In PEGASUS, a 48-week, phase 3 study with a 16-week randomized, active–controlled period of SC pegcetacoplan 1080 mg twice weekly or continued eculizumab treatment, followed by a 32-week open-label period in which all patients received pegcetacoplan [23,24]. Patients in PEGASUS had baseline hemoglobin concentrations lower than 10.5 g/dL despite receiving stable doses of eculizumab for at least 3 months before screening [23,24].

**Table 1:** Summary of the trials included in the post hoc analysis.

Original trials	PADDOCK [21] (NCT02588833)	PRINCE [22] (NCT04085601)	PEGASUS [23,24] (NCT03500549)
**Design**	Phase 1b, multiple ascending doses of pegcetacoplan	Phase 3, randomized (pegcetacoplan vs supportive care^a^)	Phase 3, randomized (pegcetacoplan vs eculizumab) for 16 weeks, followed by an open-label period in which patients continued with or switched to pegcetacoplan
**Patient population**	Adults with PNH who were complement inhibitor–naive^b^	Adults with PNH who were complement inhibitor–naive^c^ and had hemoglobin concentrations < the sex-specific LLN	Adults with PNH and hemoglobin concentrations <10.5 g/dL despite stable eculizumab dosing for ≥3 months
**Enrollment period**	September 16, 2015- March 15, 2018	August 27, 2019- December 29, 2019	June 14, 2018- November 14, 2019

LDH, lactate dehydrogenase; LLN, lower limit of normal; PNH, paroxysmal nocturnal hemoglobinuria.

^a^Blood transfusions, anticoagulants, corticosteroids, and supplements (iron, folate, and vitamin B_12_).

^b^Patients with prior eculizumab treatment were excluded.

^c^Patients with eculizumab or ravulizumab use within 3 months of screening were excluded.

### Post hoc analysis

A summary of this this post hoc analysis is provided in **Table 2**. The analysis included patients from PADDOCK, PRINCE, and PEGASUS with mild or moderate anemia (ie, hemoglobin concentrations of at least 10.0 g/dL) at baseline who had no transfusions within 14 days before the baseline hemoglobin measurement. From PRINCE, only patients initially randomized to pegcetacoplan were included. From PEGASUS, patients randomized to either pegcetacoplan or eculizumab treatment were included; for patients randomized to pegcetacoplan, data from the 16-week randomized control period were analyzed and for patients randomized to eculizumab, data from week 16 (before receiving pegcetacoplan in the randomized control period) and after 16 weeks of pegcetacoplan monotherapy (ie, after switching from eculizumab to pegcetacoplan in the open label period) were analyzed. The current analyses examined the intent-to-treat patient populations, defined as patients eligible to receive pegcetacoplan who received at least 1 dose of pegcetacoplan (PADDOCK) and all randomized patients (PRINCE, PEGASUS) within the included subpopulation.

**Table 2:** Overview of the post hoc analysis.

Study element	Description
**Patient population**	Patients from PADDOCK, PRINCE, and PEGASUS with PNH and mild/moderate anemia (ie, hemoglobin concentrations ≥10.0 g/dL) at baseline and no transfusions within 14 days before baseline
**Treatment**	**PADDOCK:** SC pegcetacoplan
	**PRINCE:** SC pegcetacoplan
	**PEGASUS:** SC pegcetacoplan or SC pegcetacoplan during the open-label period after receiving IV eculizumab during the randomized period
**Key efficacy outcomes at baseline**^**a**^ **and after 16 weeks of pegcetacoplan monotherapy**	Hemoglobin concentrations
	Percentage of patients with hemoglobin concentrations ≥12 g/dL
	Percentage of patients with hemoglobin concentrations ≥ sex-specific LLN^b^
	ARCs
	Percentage of patients with ARC normalization^c^
	LDH concentrations
	Percentage of patients with LDH normalization^d^
	FACIT-Fatigue scores
	Percentage of patients with FACIT-Fatigue score normalization^e^
**Safety**	**PADDOCK:** AEs that commenced on or after first study drug administration through 30 days after the last dose were considered TEAEs; safety events were considered through day 113 as the week 16 endpoint
	**PRINCE:** AEs that commenced on or after first study drug administration through 16 weeks of pegcetacoplan monotherapy were considered TEAEs. Patients who escaped from the control group to the pegcetacoplan arm were not included in the analysis
	**PEGASUS:** For patients randomized to pegcetacoplan, AEs that started during pegcetacoplan monotherapy during the 16-week randomized control period were considered TEAEs For patients randomized to eculizumab, AEs that started during the first 16 weeks of pegcetacoplan monotherapy in the open-label period were considered TEAEs

AE, adverse event; ARC, absolute reticulocyte count; FACIT-Fatigue, Functional Assessment of Chronic Illness Therapy-Fatigue; IV, intravenous; LDH, lactate dehydrogenase; LLN, lower limit of normal; PNH, paroxysmal nocturnal hemoglobinuria; SC, subcutaneous; TEAE, treatment-emergent adverse event.

^a^Baseline for patients from PEGASUS who were randomized to pegcetacoplan was baseline of the randomized period; baseline for patients randomized to eculizumab was week 16 of the randomized control period (ie, before switching from eculizumab to pegcetacoplan).

^b^For male patients, 13.6 g/dL and for female patients, 12.0 g/dL and the absence of transfusions.

^c^Defined as ≤ULN of 120×10^9^ cells/L and the absence of transfusions.

^d^Defined as ≤ULN of 226 U/L and the absence of transfusions.

^e^Defined as ≥43.6 (population norm) [28] and the absence of transfusions.

### Outcomes

Demographics, clinical characteristics, laboratory measurements, and Functional Assessment of Chronic Illness Therapy–Fatigue Scale (FACIT-Fatigue) scores were assessed at study baseline for all patients. Efficacy outcomes were measured at baseline of the trials and week 16 of pegcetacoplan monotherapy for all patients except those randomized to eculizumab in PEGASUS; for these patients, baseline was the end of eculizumab treatment (week 16) and the week 16 outcomes were measured after receiving 16 weeks of pegcetacoplan monotherapy. Hemoglobin outcomes included concentrations, percentage of patients with concentrations of 12 g/dL or higher, and sex-specific normalization. Sex-specific hemoglobin normalization was defined as hemoglobin concentrations of at least the sex-specific LLN (12.0 g/dL for female patients, 13.6 g/dL for male patients) and the absence of transfusions. Other hematologic outcomes include ARCs, the percentage of patients with ARC normalization (≤ULN [120×10^9^ cells/L] and the absence of transfusions, LDH concentrations, the percentage of patients with LDH normalization (ie, LDH ≤ULN [226 U/L] and the absence of transfusions), and the percentage of patients who required a transfusion. Mean FACIT-Fatigue scores and the percentage of patients with FACIT-Fatigue score normalization (ie, ≥43.6 [population norm] [28] and the absence of transfusions) were analyzed. Scores for FACIT-Fatigue range from 0 to 52, with higher scores indicating less fatigue [28]. De-identified patient data can be found in S1 File (**S1 Table in S6 File**).

### Safety

Adverse events (AEs) were coded to System Organ Class and Preferred Term using the Medical Dictionary for Regulatory Activities (MedDRA^®^) Version 21.0 (PADDOCK) or Version 23.0 (PRINCE, PEGASUS). A treatment-emergent AE (TEAE) was defined as an AE that commenced on or after the time of first study drug administration through 30 days (PADDOCK) or as long as 8 weeks (PRINCE) after the last dose. In PADDOCK, safety events were considered through day 113 as week 16 endpoint. In PEGASUS, AEs that started during their first 16 weeks of pegcetacoplan monotherapy were considered TEAEs. For patients randomized to pegcetacoplan, this was the 16-week randomized controlled period and for patients randomized to eculizumab, this was the first 16 weeks of pegcetacoplan monotherapy in the open-label period. Investigators judged the relatedness of each AE to pegcetacoplan using prespecified criteria.

### Statistical analysis

Results from this post hoc analysis, including percentages, means, medians, standard deviations (SDs), and ranges, were reported descriptively for the PADDOCK, PRINCE, and PEGASUS trials. Missing data were not imputed. Patients who withdrew from the study, were missing data at the specified timepoint, or received transfusions were classified as non-responders for normalization measures.

### Study approval

The individual study protocols were designed and monitored in accordance with the ethical principles of Good Clinical Practice and the Declaration of Helsinki [21–24] and approved by an institutional review board or independent ethics committee at each center. Each patient provided written informed consent before undergoing study-related procedures.

## Results

### Trial populations

This post hoc analysis of the PADDOCK [21], PRINCE [22], and PEGASUS [23,24] clinical trials included patients with PNH and hemoglobin concentrations of at least 10.0 g/dL at baseline (N = 25). Six patients from PADDOCK, 8 from PRINCE, and 11 from PEGASUS were included [21–24]. Seven of the patients from PEGASUS had been randomized to pegcetacoplan during the randomized control period and 4 were randomized to eculizumab and then switched to pegcetacoplan during the open-label period. One patient in PADDOCK who stopped dosing at day 29 left the study due to physician decision and was excluded from the normalization analysis but included in the safety analysis.

### Baseline characteristics

Demographic and baseline characteristics for patients with PNH and mild or moderate anemia at baseline of PADDOCK, PRINCE, and PEGASUS are shown in **Table 3**. Mean age was comparable across studies. In PADDOCK and PRINCE, most patients were Asian, and in PEGASUS, most patients were White. The median time since PNH diagnosis was 6.4 years for patients in PADDOCK and 8.0 years in PEGASUS; patients in PRINCE were diagnosed more recently, with a median time since diagnosis of 1.5 years. Patients in PADDOCK and PRINCE were complement inhibitor–naive at baseline per exclusion criteria. Patients in PEGASUS had been receiving eculizumab for a median (range) duration of 5.2 (0.2–16.7) years, with 73% (8 of 11 patients) receiving 900-mg eculizumab every 2 weeks and 3 patients receiving 1200 mg every 2 weeks.

**Table 3:** Baseline demographic and clinical characteristics for patients with PNH and mild/moderate anemia^a^ in PADDOCK, PRINCE, and PEGASUS.

Demographics	PADDOCK N = 6	PRINCE N = 8	PEGASUS N = 11
**Age, mean (range), y**	41 (22–67)	44 (30–63)	40 (22–53)
**Female patients, n (%)**	3 (50.0)	2 (25.0)	6 (54.5)
**Race, n (%)**			
Asian	5 (83.3)	7 (87.5)	2 (18.2)
White	0	0	7 (63.6)
Other	1 (16.7)	1 (12.5)	0
Not reported	0	0	2 (18.2)
**Clinical characteristics**			
BMI, mean (SD), kg/m^2^	27.1 (5.4)	24.5 (2.0)	25.3 (4.3)
Time since PNH diagnosis, median (range), y	6.4 (1.8–16.4)	1.5 (0.4–14.0)	8.0 (3.4–27.0)
No transfusions in previous 12 months, n (%)	2 (33.3)	2 (25.0)	7 (63.6)
≥4 transfusions in previous 12 months, n (%)	3 (50.0)	0	3 (27.3)
**Laboratory measurements, mean (SD)**			
Hemoglobin, g/dL^b^	10.5 (0.4)	11.3 (1.0)	10.2 (0.2)
ARC, ×10^9^ cells/L^c^	198.8 (72.4)	241.0 (55.4)	265.0 (87.4)
LDH, U/L^d^	1935.8 (629.7)	2153.1 (640.3)	202.7 (44.1)
**Patient-reported outcome**			
FACIT-Fatigue score^e^	36.7 (8.1)	35.6 (10.2)	28.4 (9.0)

ARC, absolute reticulocyte count; BMI, body mass index; FACIT-Fatigue, Functional Assessment of Chronic Illness Therapy-Fatigue; LDH, lactate dehydrogenase; PNH, paroxysmal nocturnal hemoglobinuria.

^a^Defined a hemoglobin concentration ≥10.0 g/dL.

^b^Normal reference range: Male patients, 13.6–18 g/dL; female patients, 12–16 g/dL.

^c^Normal reference ranges: All patients (PADDOCK), 30–100×10^9^ cells/L; all patients (PEGASUS), 30–120×10^9^ cells/L; male patients (PRINCE), 10–140×10^9^ cells/L and female patients (PRINCE), 10–120×10^9^ cells/L.

^d^Normal reference range: 113–226 U/L.

^e^General population norm: 43.6. Defined by Cella et al [28].

In PADDOCK, 4 of 6 patients (66.7%) with mild or moderate anemia had received at least 1 transfusion in the past 12 months; in PRINCE, 6 of 8 patients (75.0%) had received at least 1 transfusion in the 12 months prior (**Table 3**). In PEGASUS, more than one-third of patients (4 of 11 [36.4%]) had at least 1 transfusion in the previous 12 months. Three of 6 patients in PADDOCK (50.0%), 0 of 8 in PRINCE, and 3 of 11 in PEGASUS (27.3%) had at least 4 transfusions in the past 12 months. Patients included in this post hoc analysis had mean (SD) hemoglobin concentrations at baseline of 10.5 (0.4) g/dL (PADDOCK), 11.3 (1.0) g/dL (PRINCE), and 10.2 (0.2) g/dL (PEGASUS). Mean ARCs at baseline were above the ULN in all studies. As expected, patients in PADDOCK and PRINCE had pronounced LDH elevations at baseline, with concentrations 8.6× and 9.5× ULN of 226 U/L, respectively. The mean LDH concentration for PEGASUS patients at baseline was 202.7 U/L, which was somewhat below the ULN. In all studies, mean FACIT-Fatigue scores were below the general population norm of 43.6 [28].

### Efficacy

**Fig 1:**
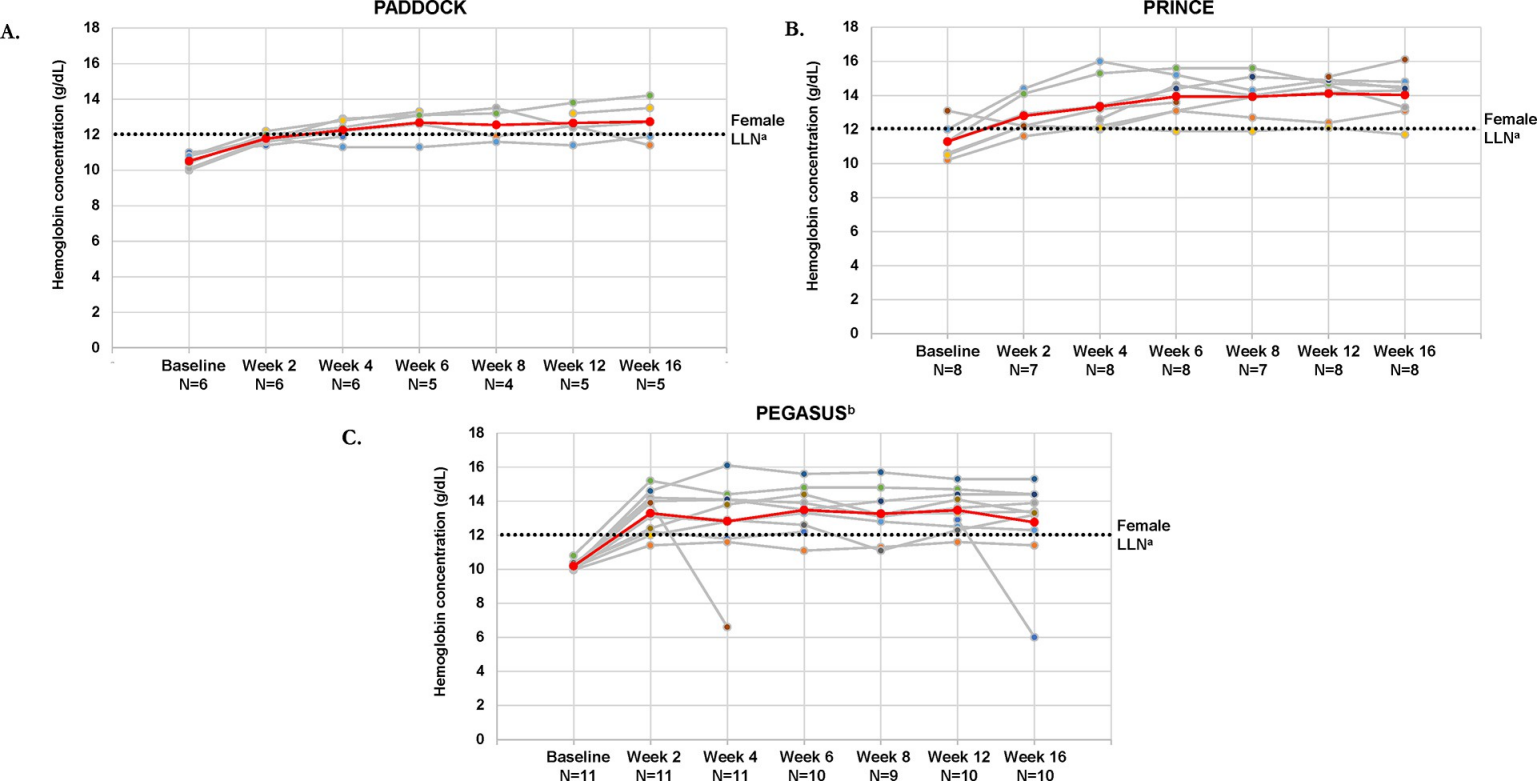
Hemoglobin concentrations over time for patients with PNH and mild/moderate anemia (hemoglobin ≥10 g/dL) for individual patients and the total populations for the PADDOCK (A), PRINCE (B), and PEGASUS (C) trials. Gray lines represent individual patient hemoglobin concentrations and the red line represents the mean hemoglobin concentrations of patients with mild/moderate anemia in each trial. LLN, lower limit of normal; PNH, paroxysmal nocturnal hemoglobinuria. ^a^LLN for female patients, 12 g/dL. ^b^This analysis was performed using week 16 as the baseline for patients who received eculizumab.

Mean and individual hemoglobin concentrations increased in the majority of patients with mild or moderate anemia after 16 weeks of pegcetacoplan in PADDOCK, PRINCE, and PEGASUS; mean hemoglobin concentrations at week 16 were greater than 12.0 g/dL in all 3 trials. In PADDOCK, mean (SD) hemoglobin concentration increased from 10.5 (0.4) g/dL at baseline to 12.7 (1.1) g/dL at week 16 (**Fig 1A**); in PRINCE, hemoglobin concentrations increased from 11.3 (1.0) g/dL to 14.0 (1.3) g/dL (**Fig 1B)**. In PEGASUS, hemoglobin concentrations increased from 10.2 (0.2) g/dL at baseline to 12.8 (2.6) g/dL at week 16 (**Fig 1C**). One patient in PEGASUS had a decrease in hemoglobin concentration at week 4; this decrease aligned with a breakthrough hemolysis that was deemed not related to the study drug, potentially triggered by post-surgery sepsis. Another patient in PEGASUS had a decreased hemoglobin concentration at week 16 that was associated with breakthrough hemolysis in the context of a respiratory infection and also considered not related to pegcetacoplan.

The mean hemoglobin concentration increases from baseline to week 16 in the subpopulations with mild or moderate anemia were consistent with observations in the primary analyses of the total populations for each study (**S1A Fig in S6 File**). Hemoglobin concentrations were consistently lower in the total study populations than in the populations with mild or moderate anemia.

**Fig 2:**
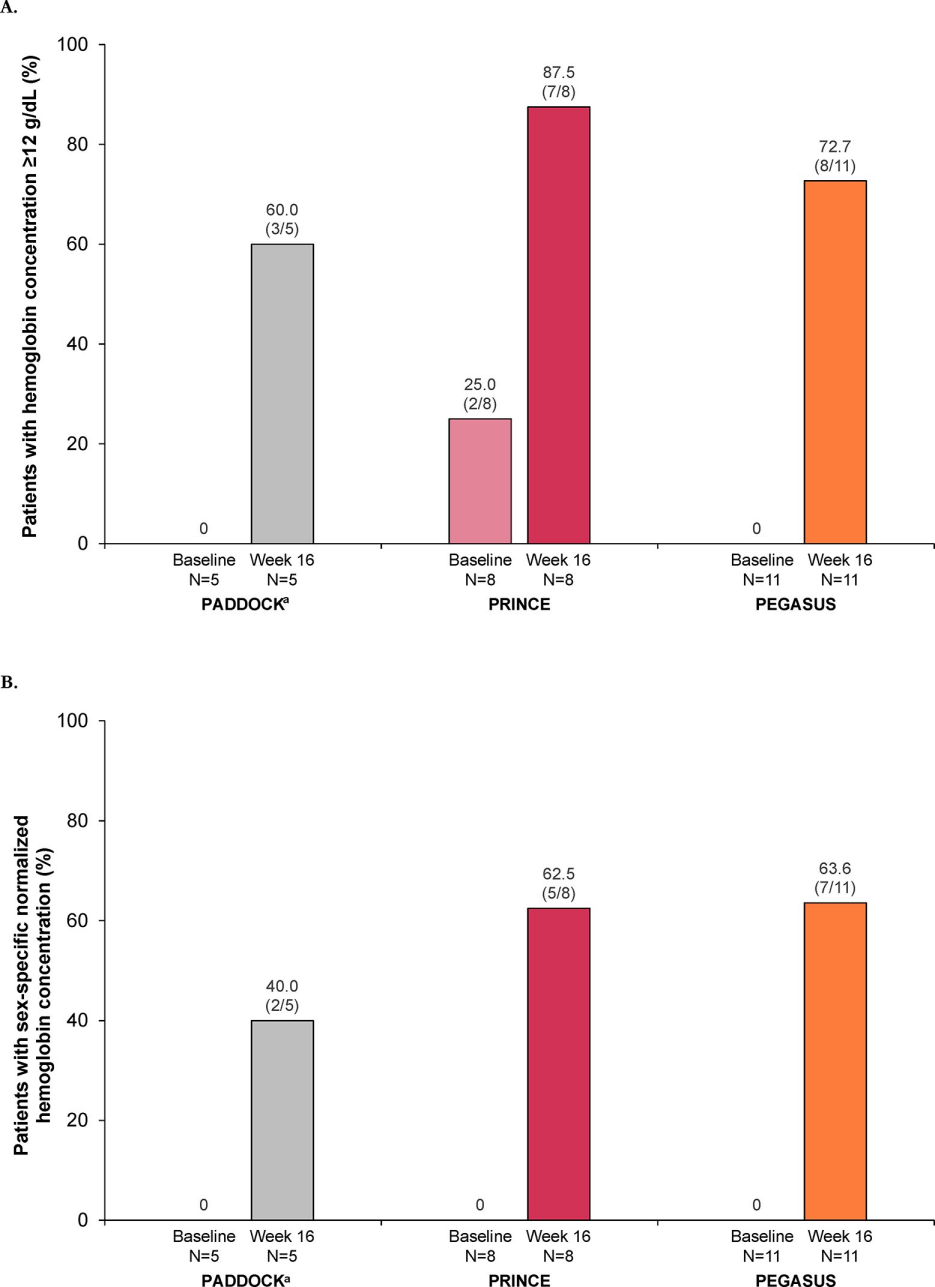
Percentage of patients with a hemoglobin concentration ≥12 g/dL (A) or sex-specific hemoglobin normalization (B) at baseline and after 16 weeks of pegcetacoplan for patients with PNH and mild or moderate anemia (hemoglobin ≥10 g/dL) in the PADDOCK, PRINCE, and PEGASUS trials. The hemoglobin response and normalization analyses included all patients in the post hoc analysis unless noted otherwise. Patients who received transfusions, withdrew from the study, or were missing data at each time point were classified as nonresponders. Sex-specific hemoglobin normalization was defined as hemoglobin concentrations greater than or equal to the sex-specific LLN (13.6 g/dL, male patients; 12.0 g/dL, female patients) and the absence of transfusions. LLN, lower limit of normal; PNH, paroxysmal nocturnal hemoglobinuria. ^a^Data from 1 patient who stopped dosing at day 29 and left the study per physician decision were not included in this analysis.

The percentages of patients with mild or moderate anemia who had hemoglobin concentrations of at least 12 g/dL in patients increased after 16 weeks of pegcetacoplan in the 3 studies (**Fig 2A**); hemoglobin concentrations of at least 12 g/dL occurred in 60% (3 of 5) of patients in PADDOCK, 87.5% (7 of 8) in PRINCE, and 72.7% (8 of 11) in PEGASUS. At baseline, none of the patients had sex-specific hemoglobin normalization; at week 16, 40.0% (2 of 5) of patients in PADDOCK, 62.5% (5 of 8) in PRINCE, and 63.6% (7 of 11) in PEGASUS had sex-specific hemoglobin normalization (**Fig 2B**).

No patients from PADDOCK or PRINCE who had mild or moderate anemia required a transfusion through week 16 of pegcetacoplan treatment. Two of 11 patients (18.2%) in PEGASUS required a transfusion through week 16 pegcetacoplan. One patient who required transfusion had developed an upper respiratory infection that was deemed likely to have triggered the hemolysis event; the patient was discharged from the hospital after the event and remained in the study. The other patient needing transfusion had jugular vein thrombosis in the setting of pneumonia infection in the presence of renal failure, sepsis, and circulatory collapse, which was deemed unrelated to pegcetacoplan and resolved on the day of onset, following heparin administration; this patient eventually discontinued the trial.

**Fig 3:**
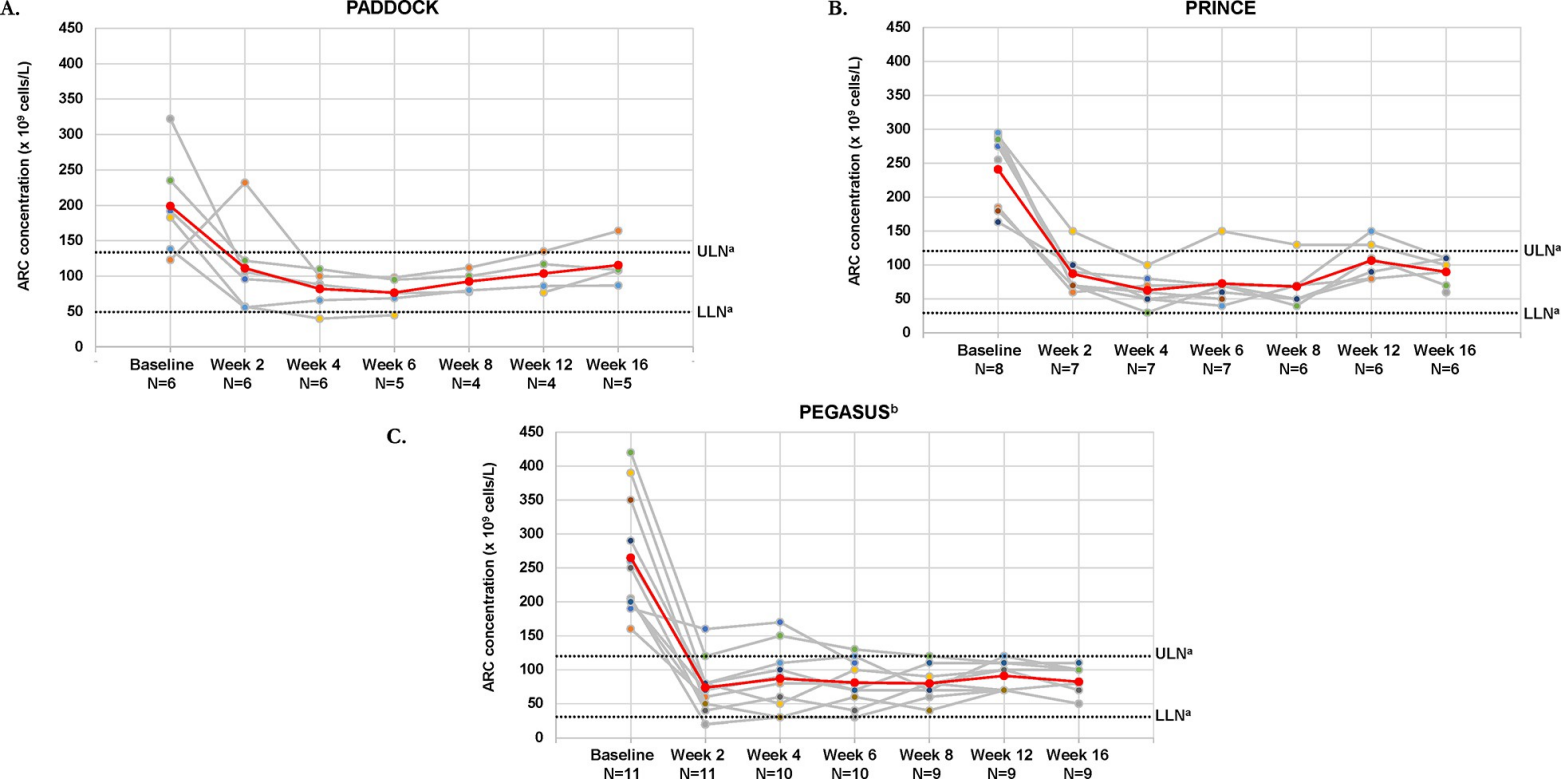
ARCs over time for patients with PNH and mild/moderate anemia (hemoglobin ≥10 g/dL) for individual patients and the total populations for the PADDOCK (A), PRINCE (B), and PEGASUS (C) trials. Gray lines represent individual patient ARCs and the red line represents the mean ARCs of patients with mild/moderate anemia in each trial. ARC, absolute reticulocyte count; LLN, lower limit of normal; PNH, paroxysmal nocturnal hemoglobinuria; ULN, upper limit of normal. ^a^Normal reference ranges: 30–100×10^9^ cells/L. ^b^This analysis was performed using week 16 as the baseline for patients who received eculizumab.

In the 3 studies, mean ARCs for patients with mild or moderate anemia were above the ULN at baseline and decreased to within (PRINCE, PEGASUS) or near (PADDOCK) normal limits by week 16 (**Fig 3**). At week 16, ARCs for individual patients with available data were within or near normal limits, except for 1 patient in PADDOCK who still had an elevated ARC. Both the total and mildly or moderately anemic populations showed a marked decrease in ARCs from baseline to week 16 (**S1B Fig in S6 File**).

**Fig 4:**
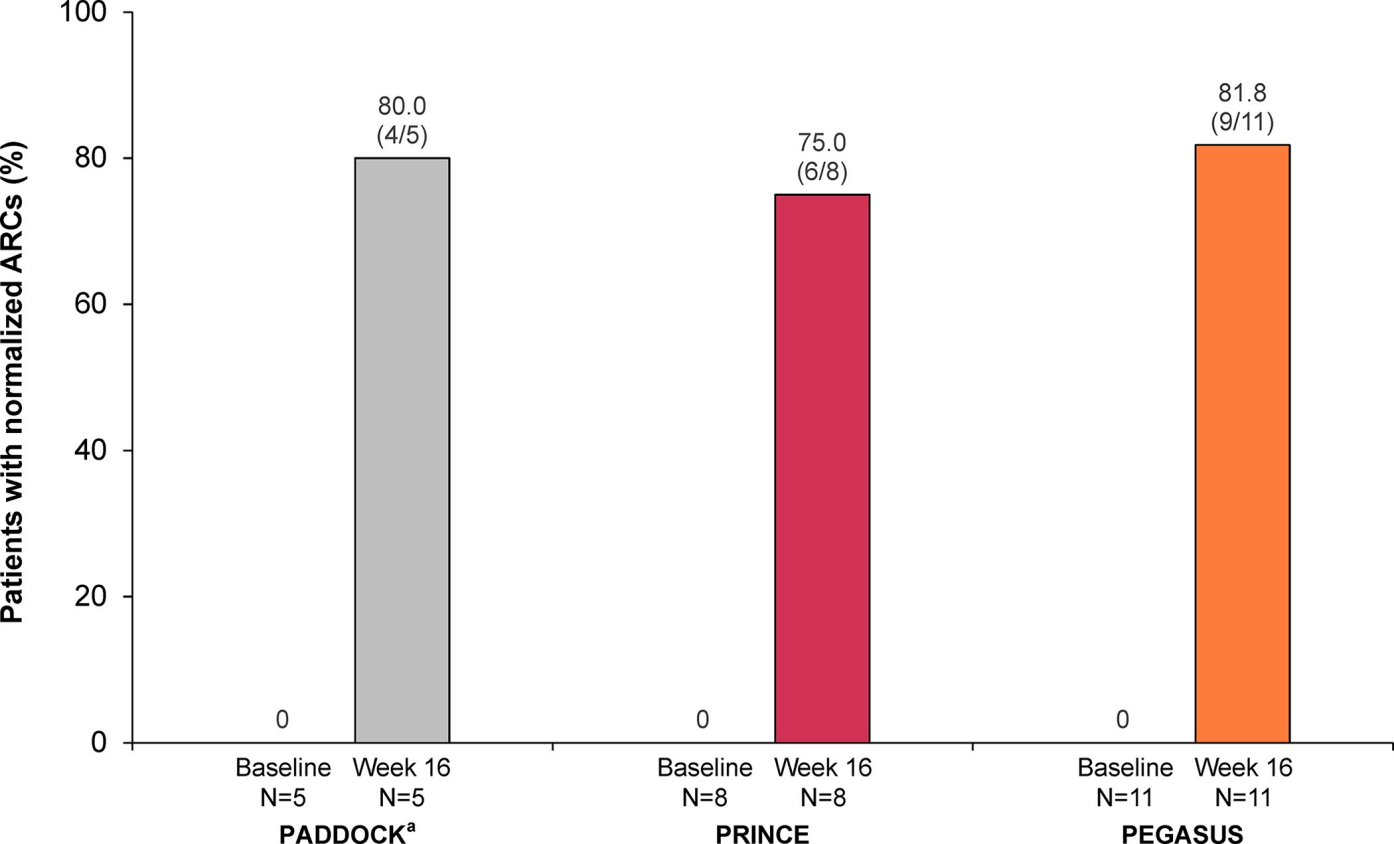
ARC normalization at baseline and after 16 weeks of pegcetacoplan treatment are shown for patients with PNH and mild/moderate anemia (hemoglobin ≥10.0 g/dL) in the PADDOCK, PRINCE, and PEGASUS trials. The normalization analysis included all patients in the post hoc analysis unless noted otherwise. Patients who received transfusions, withdrew from the study, or were missing data at each time point were classified as nonresponders. Normalization of ARC is defined as ≤ULN (120×10^9^ cells/L) and the absence of transfusions. ARC, absolute reticulocyte count; LLN, lower limit of normal; PNH, paroxysmal nocturnal hemoglobinuria; ULN, upper limit of normal. ^a^Data from 1 patient who stopped dosing at day 29 and left the study per physician decision were not included in this analysis.

At week 16, ARC normalization had occurred in 80.0% (4 of 5 patients), 75.0% (6 of 8), and 81.8% (9 of 11) of patients evaluable for normalization in PADDOCK, PRINCE, and PEGASUS, respectively (**Fig 4**).

**Fig 5:**
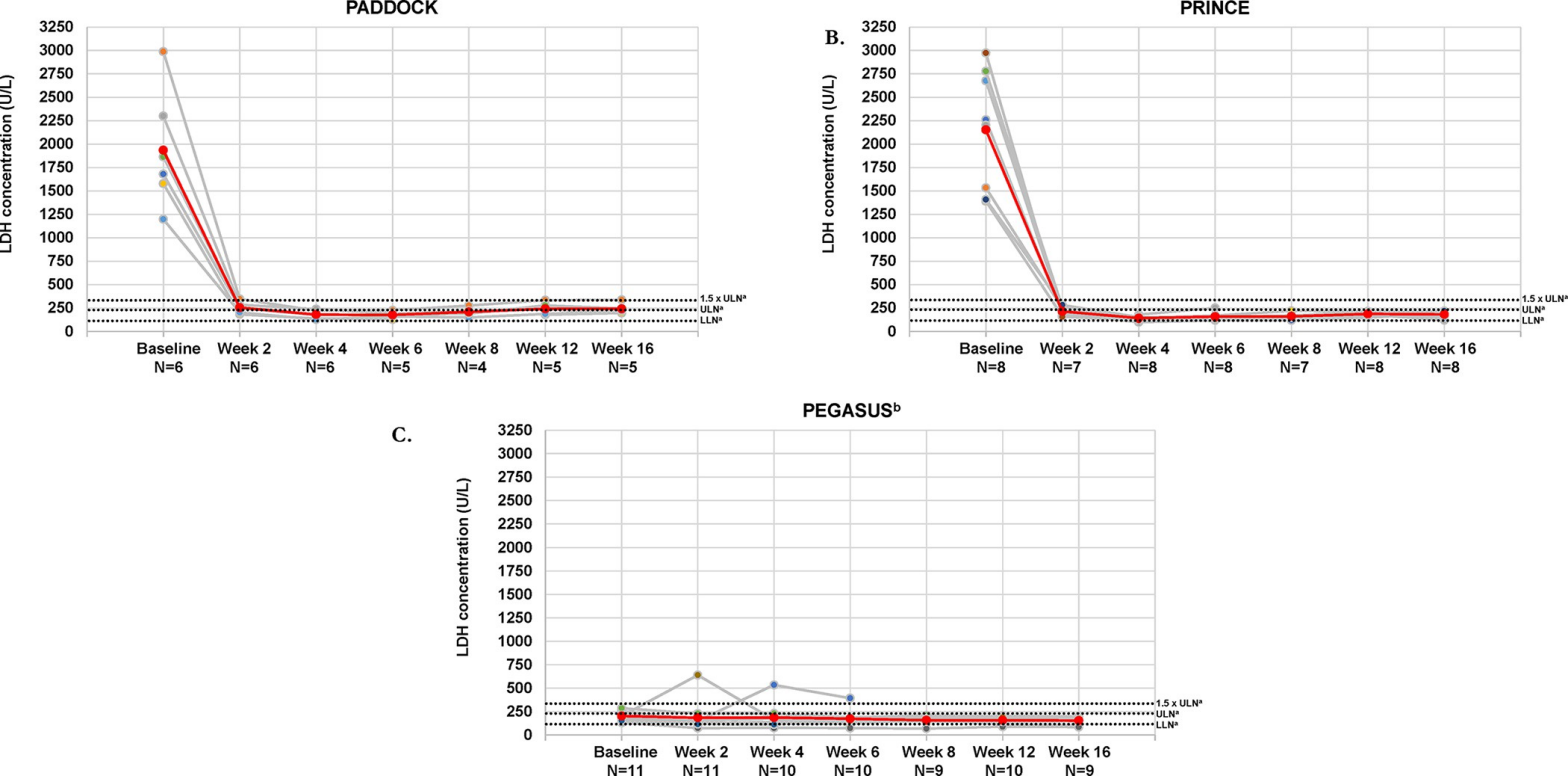
LDH concentrations over time for patients with PNH and mild/moderate anemia (hemoglobin ≥10 g/dL) for individual patients and the total populations for the PADDOCK (A), PRINCE (B), and PEGASUS (C) trials. Gray lines represent individual patient LDH concentrations and the red line represents the mean LDH concentration of patients with mild/moderate anemia in each trial. LDH, lactate dehydrogenase; LLN, lower limit of normal; PNH, paroxysmal nocturnal hemoglobinuria; ULN, upper limit of normal. ^a^Normal reference range: 113–226 U/L. ^b^This analysis was performed using week 16 as the baseline for patients who received eculizumab.

In patients with mild or moderate anemia who were complement inhibitor–naive at baseline, mean LDH concentrations decreased from well above the ULN at baseline to slightly above the ULN (PADDOCK) (**Fig 5A**) or within the normal range (PRINCE) (**Fig 5B**) at week 16. Patients in PEGASUS had mean (SD) LDH concentrations below the ULN (202.7 [44.1] U/L) at baseline, and these concentrations decreased at week 16 (154.9 [36.2] U/L) (**Fig 5C**). Changes in LDH concentrations from baseline to week 16 of pegcetacoplan were comparable between the total and mild/moderate anemia populations in all studies (**S1C Fig in S6 File**).

**Fig 6:**
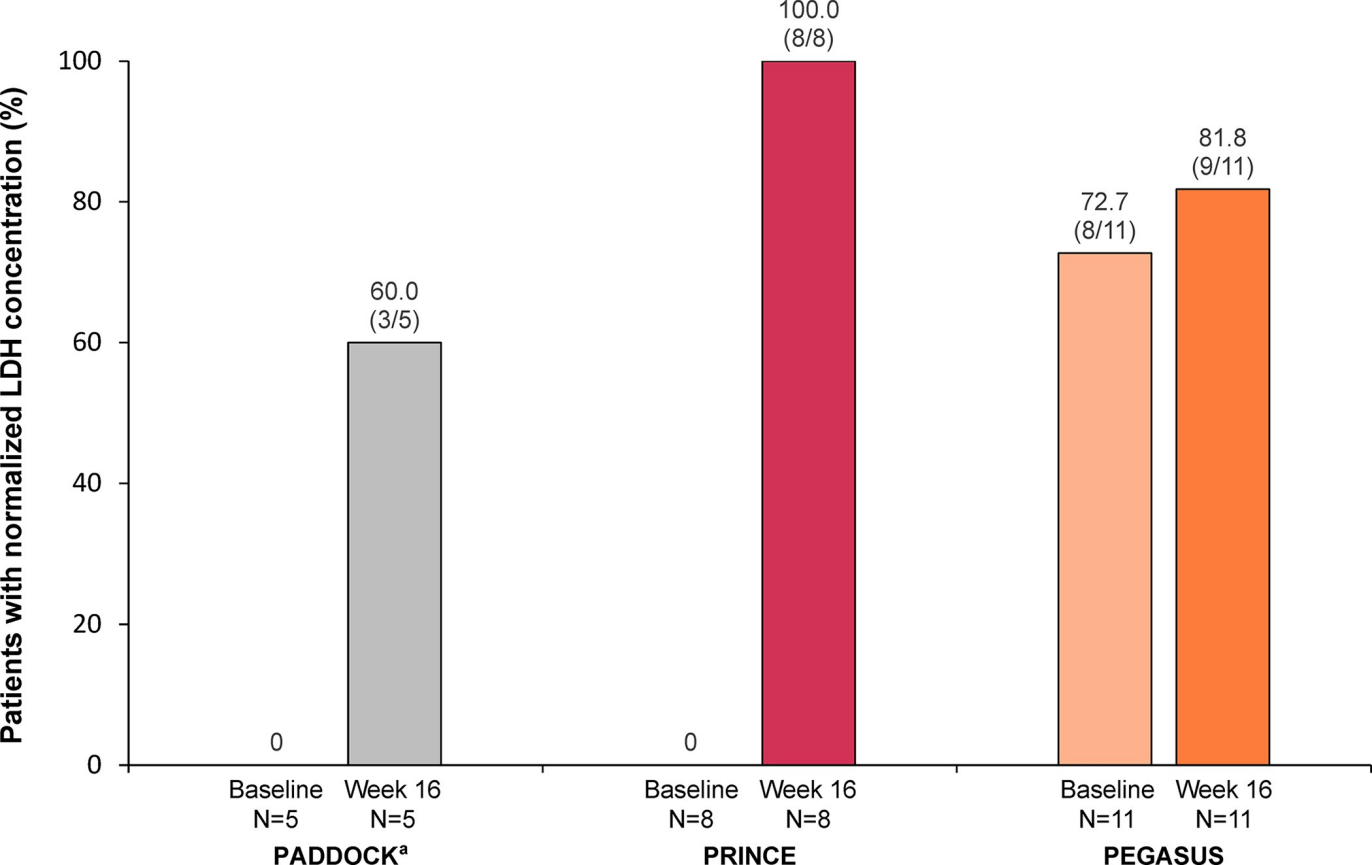
LDH analysis at baseline and after 16 weeks of pegcetacoplan treatment for patients with PNH and mild/moderate anemia (hemoglobin ≥10.0 g/dL) in the PADDOCK, PRINCE, and PEGASUS trials. The normalization analysis included all patients in the post hoc analysis unless noted otherwise. Patients who received transfusions, withdrew from the study, or were missing data at each time point were classified as nonresponders. Normalization of LDH is defined as ≤226 U/L and the absence of transfusions. LDH, lactate dehydrogenase; LLN, lower limit of normal; PNH, paroxysmal nocturnal hemoglobinuria; ULN, upper limit of normal. ^a^Data from 1 patient who stopped dosing at day 29 and left the study per physician decision were not included in this analysis.

In PADDOCK and PRINCE, no patients with mild or moderate anemia had normalized LDH at baseline; after 16 weeks of pegcetacoplan, 60% (3 of 5) of patients from PADDOCK and 100.0% (8 of 8) from PRINCE had normalized LDH (**Fig 6**). In patients from PEGASUS, normalization of LDH increased slightly from 72.7% (8 of 11) of patients at baseline to 81.8% (9 of 11) after 16 weeks of pegcetacoplan.

**Fig 7:**
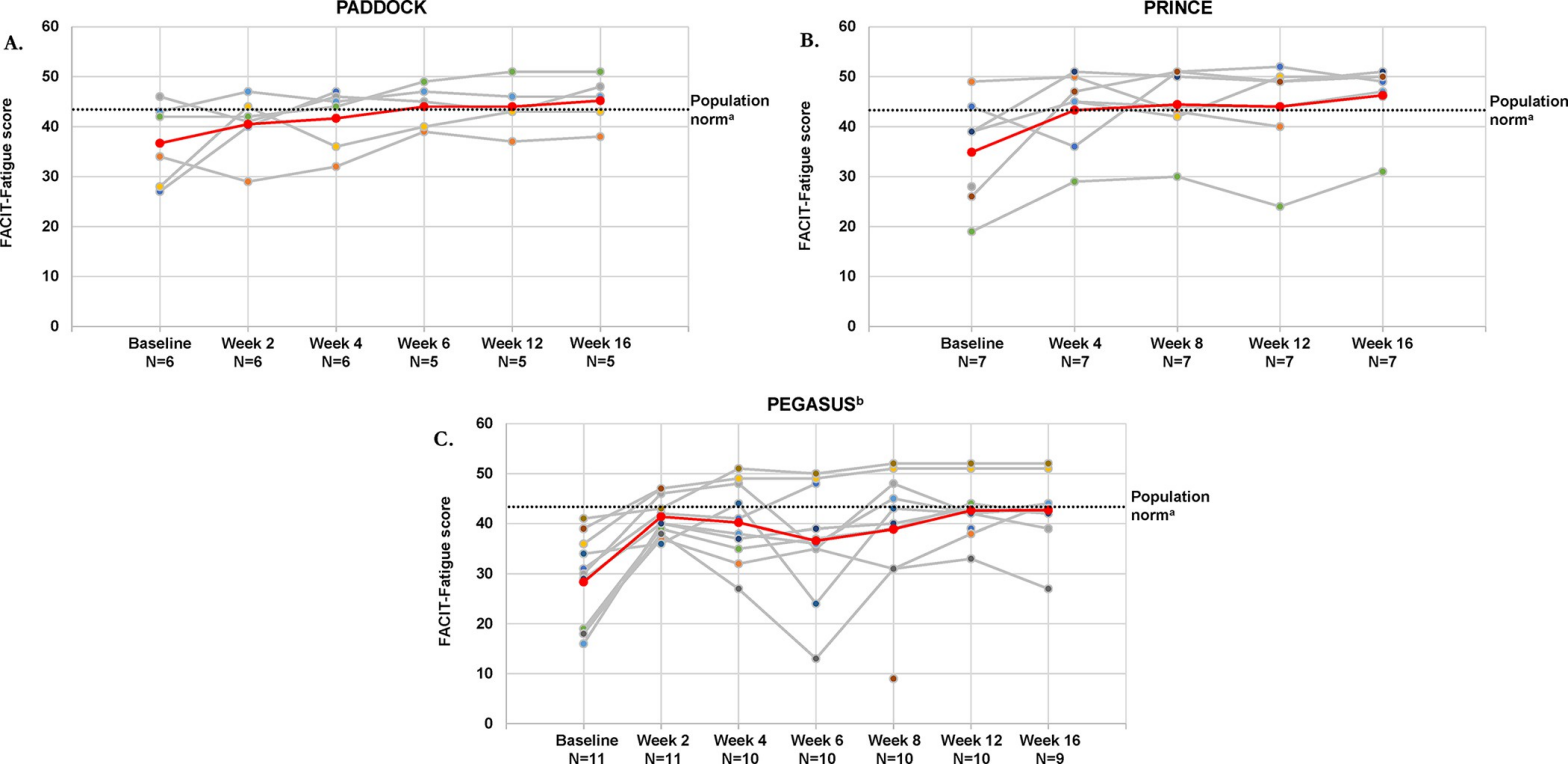
FACIT-Fatigue scores over time for patients with PNH and mild/moderate anemia (hemoglobin ≥10 g/dL) for individual patients and the total populations for the PADDOCK (A), PRINCE (B), and PEGASUS (C) trials. Gray lines represent individual patient FACIT-Fatigue scores and the red line represents the mean FACIT-Fatigue scores of patients with mild/moderate anemia in each trial. FACIT-Fatigue, Functional Assessment of Chronic Illness Therapy-Fatigue; PNH, paroxysmal nocturnal hemoglobinuria. ^a^General population norm: 43.6. Defined by Cella et al [28]. ^b^This analysis was performed using week 16 as the baseline for patients who received eculizumab.

In patients with mild or moderate anemia, mean (SD) FACIT-Fatigue scores improved from below the general population norm at baseline to above the norm at week 16 in PADDOCK (36.7 [8.1] to 45.2 [5.0]) (**Fig 7A**) and PRINCE (35.6 [10.2] to 46.3 [7.0]) (**Fig 7B**). In PEGASUS, mean (SD) FACIT-Fatigue scores were well below the population norm at baseline and increased to slightly below the population norm at week 16 (28.4 [9.0] to 42.7 [7.2]) (**Fig 7C**). In both the full patient populations and the mild/moderate anemia populations, the mean FACIT-Fatigue scores for patients with mild/moderate anemia increased from below the population norm at baseline to above or near the norm after 16 weeks of pegcetacoplan (**S1D Fig in S6 File**).

**Fig 8:**
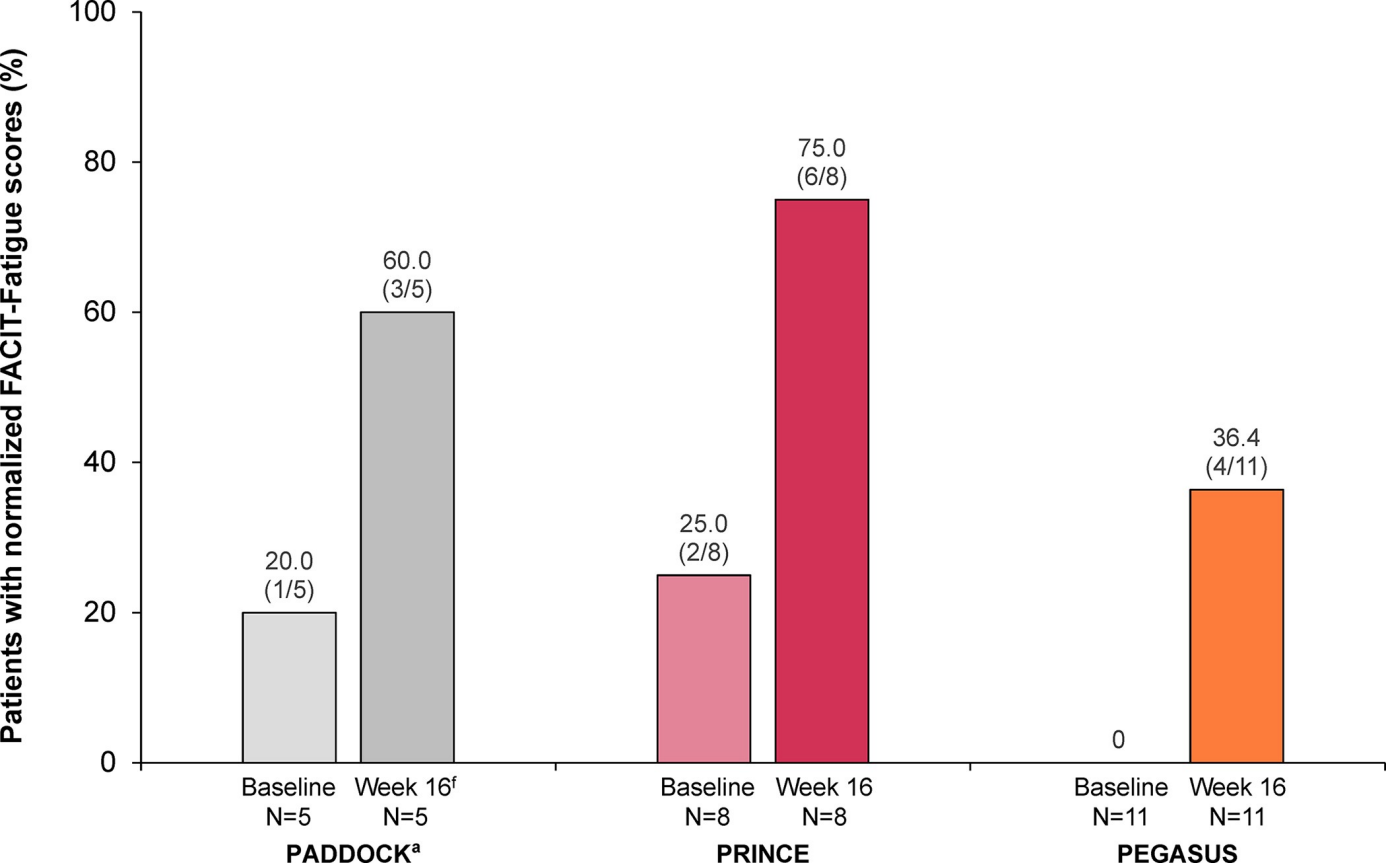
FACIT-Fatigue score normalization at baseline and after 16 weeks of pegcetacoplan treatment are shown for patients with PNH and mild/moderate anemia (hemoglobin ≥10.0 g/dL) in the PADDOCK, PRINCE, and PEGASUS trials. The normalization analysis included all patients in the post hoc analysis unless noted otherwise. Patients who received transfusions, withdrew from the study, or were missing data at each time point were classified as nonresponders. Normalization of FACIT-Fatigue was defined as an increase in FACIT-Fatigue score to greater than or equal the general population mean of 43.6 [28] and the absence of transfusions. FACIT-Fatigue, Functional Assessment of Chronic Illness Therapy-Fatigue; PNH, paroxysmal nocturnal hemoglobinuria. ^a^Data from 1 patient who stopped dosing at day 29 and left the study per physician decision were not included in this analysis.

The percentage of patients with normalized FACIT-Fatigue scores increased markedly in patients with mild or moderate anemia from all 3 studies (**Fig 8**).

### Safety

In all 3 trials, most patients with mild or moderate anemia at baseline reported at least 1 TEAE (**Table 4**). None of these patients in PADDOCK or PRINCE reported a serious AE. In PEGASUS, 2 of 11 patients (18.2%) reported a serious AE, including 1 patient with post-surgery biliary sepsis (after a cholecystectomy and a second surgery 2 days later for biliary prosthesis replacement) followed by mesenteric ischemia and 1 patient with breakthrough hemolysis following a complement-amplifying condition of an upper respiratory condition. The patient with the biliary sepsis event recovered; the event was judged as related to pegcetacoplan treatment. In response to the serious breakthrough hemolysis event, the patient’s pegcetacoplan dose was escalated and the patient received 2 transfusions. The event resolved and did not lead to study discontinuation; the investigator deemed this serious AE as not related to pegcetacoplan. There were no other serious AEs or TEAEs of hemolysis in any patient.

**Table 4:** Safety outcomes for patients with PNH and mild/moderate anemia^a^ in PADDOCK, PRINCE, and PEGASUS.

n (%)	PADDOCK N = 6^b^	PRINCE N = 8	PEGASUS N = 11
**Any TEAEs**	4 (66.7)	7 (87.5)	11 (100.0)
**Serious AEs**	0	0	2 (18.2)^c^
**Injection site reactions**	0	1 (12.5)	4 (36.4)
**Infections and infestations** ^**d**^	1 (16.7)	1 (12.5)	6 (54.5)
**Thrombotic events**	0	0	0

AE, adverse event; PNH, paroxysmal nocturnal hemoglobinuria; TEAE, treatment-emergent adverse event.

^a^Defined as a hemoglobin concentration ≥10.0 g/dL.

^b^One patient in the PADDOCK trial who stopped pegcetacoplan treatment on day 29 and left the study due to physician decision was included in this safety analysis.

^c^The serious AEs were documented as 1 patient with post-surgery sepsis followed by mesenteric ischemia and 1 patient with breakthrough hemolysis that was deemed not related to pegcetacoplan and did not lead to study discontinuation.

^d^Observed infections included nasopharyngitis, urinary tract infection, viral infection, gastrointestinal infection, tonsillitis, and vulvovaginal mycotic infection.

Injection site reactions occurred in 0 of 6 patients in PADDOCK, 1 of 8 in PRINCE (12.5%), and 4 of 11 in PEGASUS (36.4%). Infections and infestations occurred in 1 of 6 patients in PADDOCK (16.7%), 1 of 8 in PRINCE (12.5%), and 6 of 11 in PEGASUS (54.5%). Observed infections included nasopharyngitis, urinary tract infection, viral infection, gastrointestinal infection, tonsillitis, and vulvovaginal mycotic infection. There were no thrombotic events in this subpopulation in the 3 studies.

## Discussion

Pegcetacoplan represents a new era of PNH treatment by targeting earlier steps of complement pathway activation [24,27]. In this post hoc study, patients with mild or moderate anemia who were C5i treatment–naive had hematologic improvements and decreased fatigue after initiating pegcetacoplan. Likewise, patients with PNH and mild or moderate anemia (ie, hemoglobin concentrations of at least 10 g/dL) despite stable dosing of the C5i eculizumab had clinically meaningful improvements in hematologic outcomes and diminished fatigue after switching to pegcetacoplan. Hemolysis control with C5is may be incomplete because of C3-mediated EVH [1,20]. Consequently, C5is may not normalize hemoglobin concentrations [18,29], as shown by the experience of C5i-treated patients, of whom more than 20% are transfusion-dependent and as many as 89% experience fatigue, which is known to be associated with chronic, low-grade hemolysis [16,17].

Patients with PNH receiving C5is who have mild or moderate anemia have a high burden of disease despite higher hemoglobin concentrations. Among C5i-treated patients with hemoglobin concentrations of 10.5 g/dL or higher, hemolytic (transfusions, breakthrough hemolysis) and health-related QoL outcomes, including fatigue, are comparable to those of patients with hemoglobin concentrations lower than 10.5 g/dL [30,31]. In a survey of C5i-treated patients with hemoglobin concentrations of 10.5 g/dL or higher, 28% reported breakthrough hemolysis in the past 12 months [30]. C5i-treated patients with hemoglobin of 10.5 g/dL or higher had a mean FACIT-Fatigue score of 34.6, well below the general population norm of 43.6 [28,31].

In the current analysis, patients from PEGASUS, who had received stable eculizumab dosing for at least 3 months, experienced at baseline what would be considered a typical response to eculizumab. Before pegcetacoplan treatment, their mean hemoglobin concentration of 10.2 g/dL was comparable to the concentrations of less than 10.5 g/dL generally observed in patients receiving eculizumab and ravulizumab [18–20]. In the current study, 27% of patients from PEGASUS were receiving a higher-than-recommended dose of eculizumab prior to pegcetacoplan initiation [32], much like previous reports of 30% to 43% of eculizumab-treated patients with PNH receiving higher-than-recommended eculizumab maintenance dosages [24,33]. Although patients in PEGASUS who had mild or moderate anemia at baseline had received stable, sometimes elevated, eculizumab dosages and had LDH values lower than 1.5× ULN (considered controlled while receiving a C5i [34]) and a mean hemoglobin concentration of 10.2 g/dL, 36% (4 of 11) of patients had received at least 1 transfusion in the 12 months prior to baseline. After switching to pegcetacoplan, the mean hemoglobin concentrations increased to 12.8 g/dL after 16 weeks and 18% (2 of 11) of patients had required transfusions; one in the setting of an upper respiratory infection and the other in the setting of pneumonia infection with several concomitant conditions. Mean ARCs decreased from above the ULN at baseline to within the normal range at week 16. Mean LDH concentrations, which were approaching the ULN at baseline, decreased further with 16 weeks of pegcetacoplan treatment. These additional gains of increased hemoglobin concentrations, reduced intravascular hemolysis, and reduced transfusion requirements while receiving on-label pegcetacoplan dosages demonstrate the importance of aiming for greater disease control in patients with PNH and mild or moderate anemia.

Results from PADDOCK and PRINCE patients with mild or moderate anemia support the efficacy of pegcetacoplan in those who were complement inhibitor naive. In patients from both trials, mean hemoglobin concentrations increased from 11.3 g/dL or lower to 12.7 g/dL or higher and mean ARC and LDH decreased from well above the ULN to near or within normal limits after 16 weeks of pegcetacoplan treatment.

Pegcetacoplan also reduced fatigue in these patients regardless of C5i history. Mean scores for FACIT-Fatigue were below the population norm at baseline in all 3 studies and improved to above (PADDOCK, PRINCE) or approaching (PEGASUS) normal after 16 weeks of pegcetacoplan.

The advent of targeted C3 therapy has made further improvements in hemoglobin concentrations and greater reductions in fatigue an achievable goal for many patients with PNH, regardless of C5i experience or hemolysis severity. In the total population of the randomized controlled period of the PEGASUS trial, in which patients with PNH received either pegcetacoplan or eculizumab monotherapy, greater percentages in the pegcetacoplan group had hemoglobin concentrations of at least 12 g/dL (37% [15 of 41] with pegcetacoplan vs. 0 of 39 with eculizumab), sex-specific normalization of hemoglobin concentrations (34% [14 of 41] vs. 0 of 39), normalization of ARC (78% [32 of 41] vs. 3% [1 of 39]), and normalization of LDH (71% [29 of 41] vs. 15% [6 of 39]) [23]. In the current analysis, 72.7% (8 of 11) of PEGASUS patients had hemoglobin concentrations of at least 12 g/dL at 16 weeks after switching to pegcetacoplan. In addition, ARC was normalized in 81.8% (9 of 11) of patients; the rate of LDH normalization was the same (81.8% [9 of 11]). Primary results from the PRINCE study showed hemoglobin concentrations of at least 12 g/dL in 48.6% (17 of 35) of the complement inhibitor-naive patients who received pegcetacoplan for 26 weeks and sex-specific hemoglobin normalization in 45.7% (16 of 35); ARC was normalized in 60.0% (21 of 35) and LDH in 65.7% (23 of 35) [22]. In the current analysis, improvement was also observed in eculizumab-naive patients with PNH and mild or moderate anemia. After 16 weeks of pegcetacoplan treatment, 60.0% (3 of 5) patients in PADDOCK and 87.5% (7 of 8) patients in PRINCE had hemoglobin concentrations of at least 12 g/dL; more than 75% had ARC normalization and 60% to 100% had LDH normalization. Normalization of fatigue was more pronounced in complement inhibitor–naive patients; however, the complement inhibitor–experienced patients had a lower mean FACIT-Fatigue score at baseline. Overall, these findings indicate that patients with PNH and mild or moderate anemia may benefit from pegcetacoplan.

Pegcetacoplan was well-tolerated in patients with PNH and mild or moderate anemia. Two serious AEs were reported (both in PEGASUS): 1 patient with sepsis post-surgery and 1 patient with breakthrough hemolysis following a complement-amplifying condition of an upper respiratory condition. The breakthrough hemolysis event was rated as not related to treatment and resolved without study discontinuation. Because the strong complement cascade inhibition of proximal complement inhibitors for PNH, including pegcetacoplan, such therapies result in a large, circulating clones of PNH red blood cells, a reflection of their efficacy; however, more PNH red blood cells are then susceptible to complement-mediated hemolysis [35,36]. Two recent reports, one with real-world data and one with clinical trial data, have provided evidence that the breakthrough hemolysis events in pegcetacoplan-treated patients with PNH can be managed by short-term intensive dosing of pegcetacoplan and, in some cases, the temporary addition of eculizumab [35,36].

Few injection site reactions were reported, and infections and infestations occurred in less than 20% of patients in PADDOCK and PRINCE and 55% of patients in PEGASUS. There were no thrombotic events. The absence of thrombotic events related to pegcetacoplan in this subpopulation of patients with mild or moderate anemia aligns with a previous analysis of the total study populations of PRINCE and PEGASUS [22–24].

Limitations of this study include the post hoc nature of the analysis and the small number of patients with mild or moderate anemia in the pegcetacoplan clinical trials. Within the analyzed population, data were missing for some patients, which further reduced the patient numbers and limited the generalizability of these findings. However, to our knowledge, this is the first study evaluating complement-targeted therapies in the subgroup of patients with PNH and mild or moderate anemia. Future studies that extend the analysis to a larger sample size over a longer duration are needed to identify effects of pegcetacoplan on long-term outcomes, including end organ damage and AEs such as acute hemolytic events, in this population.

## Conclusions

Targeted C3 therapy with pegcetacoplan represents a new generation of treatment for patients with PNH. Pegcetacoplan normalized hematologic parameters and reduced fatigue in substantial percentages of clinical trial patients with PNH with mild or moderate anemia. Patients from the PEGASUS trial who had mild or moderate anemia at baseline despite stable eculizumab dosing, of whom more than one-third required transfusions and more than one-fourth required eculizumab dose escalation, represent the typical response to eculizumab. These patients had clinically meaningful hematologic responses and decreased fatigue after switching to pegcetacoplan. Likewise, pegcetacoplan improved hematologic parameters and diminished fatigue in C5i-naive patients with mild or moderate anemia from the PADDOCK and PRINCE trials. Pegcetacoplan was generally safe and well-tolerated in patients with PNH and mild or moderate anemia. The current findings suggest that patients with mild or moderate anemia benefited from targeted C3 therapy and should strive for additional improvement in hematologic parameters.

## Supporting information

S1 File (PPTX)

S2 File (PPTX)

S3 File (PPTX)

S4 File (PDF)

S5 File (PDF)

S6 File S1 Table. De-identified patient data. **S1 Fig A**. Hemoglobin concentrations (baseline vs. 16 weeks of pegcetacoplan) for patients with PNH and mild/moderate anemia (hemoglobin ≥10 g/dL) in the PADDOCK, PRINCE, and PEGASUS trials. **S1 Fig B**. ARCs (baseline vs. 16 weeks of pegcetacoplan) for patients with PNH and mild/moderate anemia (hemoglobin ≥10 g/dL) in the PADDOCK, PRINCE, and PEGASUS trials. **S1 Fig C**. LDH concentrations (baseline vs. 16 weeks of pegcetacoplan) for patients with PNH and mild/moderate anemia (hemoglobin ≥10 g/dL) in the PADDOCK, PRINCE, and PEGASUS trials. **S1 Fig D**. FACIT-Fatigue score analysis (baseline vs. 16 weeks of pegcetacoplan) for patients with PNH and mild/moderate anemia (hemoglobin ≥10 g/dL) in the PADDOCK, PRINCE, and PEGASUS trials.
(PDF)
