# Supplementary material for: Improvements in hematologic markers and decreases in fatigue with pegcetacoplan for patients with paroxysmal nocturnal hemoglobinuria and mild or moderate anemia (hemoglobin ≥10 g/dL) who had received eculizumab or were naive to complement inhibitors
Source: PLoS One. 2024 Jul 29;19(7):e0306407. doi: 10.1371/journal.pone.0306407 (PMC11285951; doi:10.1371/journal.pone.0306407)
Supplement: S1 File — (PPTX) [file pone.0306407.s001.pptx]

## Slide 1
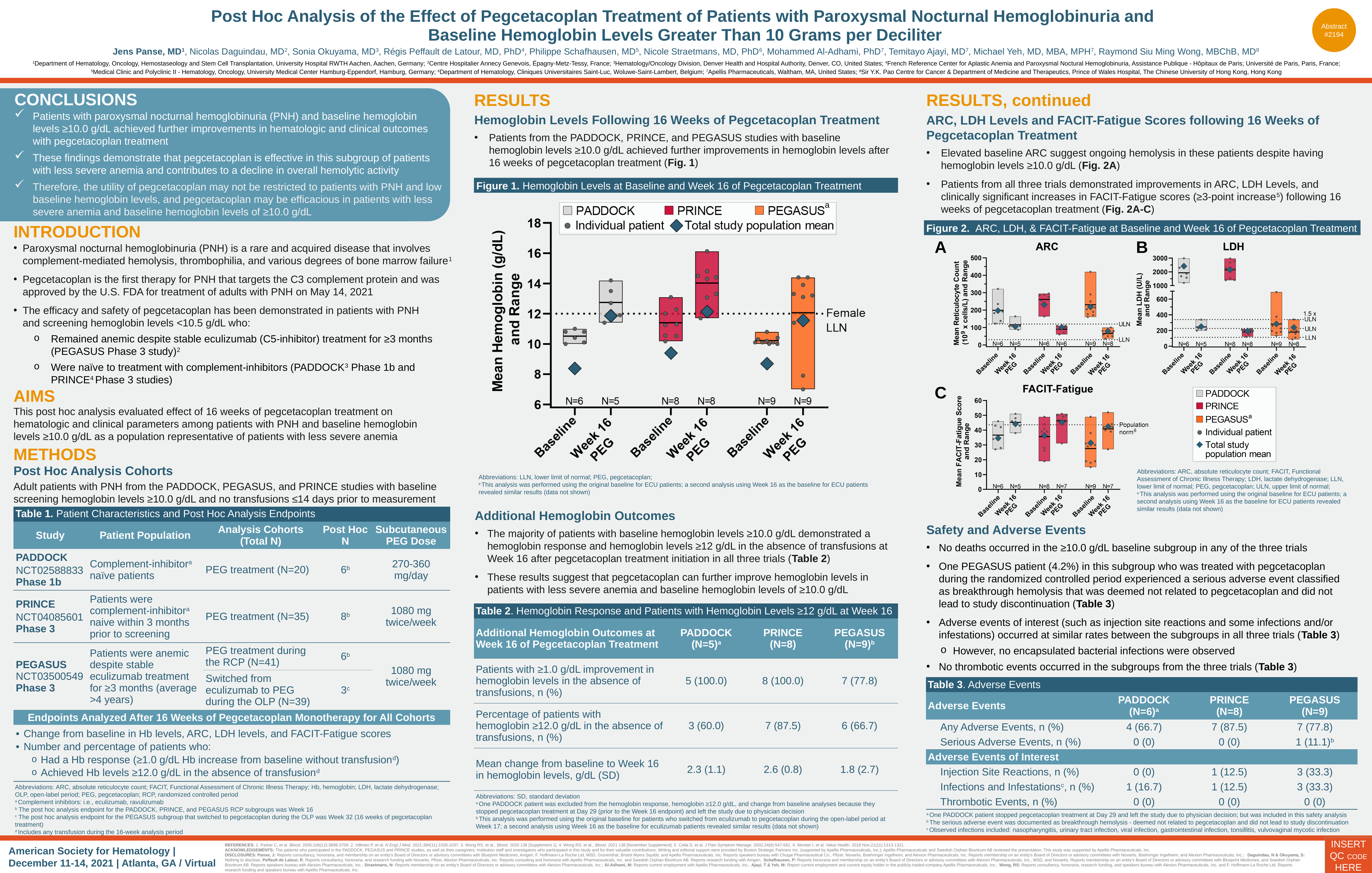

Post Hoc Analysis of the Effect of Pegcetacoplan Treatment of Patients with Paroxysmal Nocturnal Hemoglobinuria and
Baseline Hemoglobin Levels Greater Than 10 Grams per Deciliter
Abstract #2194
Jens Panse, MD1, Nicolas Daguindau, MD2, Sonia Okuyama, MD3, Régis Peffault de Latour, MD, PhD4, Philippe Schafhausen, MD5, Nicole Straetmans, MD, PhD6, Mohammed Al-Adhami, PhD7, Temitayo Ajayi, MD7, Michael Yeh, MD, MBA, MPH7, Raymond Siu Ming Wong, MBChB, MD8
1Department of Hematology, Oncology, Hemostaseology and Stem Cell Transplantation, University Hospital RWTH Aachen, Aachen, Germany; 2Centre Hospitalier Annecy Genevois, Épagny-Metz-Tessy, France; 3Hematology/Oncology Division, Denver Health and Hospital Authority, Denver, CO, United States; 4French Reference Center for Aplastic Anemia and Paroxysmal Noctural Hemoglobinuria, Assistance Publique - Hôpitaux de Paris; Université de Paris, Paris, France; 5Medical Clinic and Polyclinic II - Hematology, Oncology, University Medical Center Hamburg-Eppendorf, Hamburg, Germany; 6Department of Hematology, Cliniques Universitaires Saint-Luc, Woluwe-Saint-Lambert, Belgium; 7Apellis Pharmaceuticals, Waltham, MA, United States; 8Sir Y.K. Pao Centre for Cancer & Department of Medicine and Therapeutics, Prince of Wales Hospital, The Chinese University of Hong Kong, Hong Kong
CONCLUSIONS
RESULTS
RESULTS, continued
Patients with paroxysmal nocturnal hemoglobinuria (PNH) and baseline hemoglobin levels ≥10.0 g/dL achieved further improvements in hematologic and clinical outcomes with pegcetacoplan treatment
These findings demonstrate that pegcetacoplan is effective in this subgroup of patients with less severe anemia and contributes to a decline in overall hemolytic activity
Therefore, the utility of pegcetacoplan may not be restricted to patients with PNH and low baseline hemoglobin levels, and pegcetacoplan may be efficacious in patients with less severe anemia and baseline hemoglobin levels of ≥10.0 g/dL
Hemoglobin Levels Following 16 Weeks of Pegcetacoplan Treatment
Patients from the PADDOCK, PRINCE, and PEGASUS studies with baseline hemoglobin levels ≥10.0 g/dL achieved further improvements in hemoglobin levels after 16 weeks of pegcetacoplan treatment (Fig. 1)
ARC, LDH Levels and FACIT-Fatigue Scores following 16 Weeks of Pegcetacoplan Treatment
Elevated baseline ARC suggest ongoing hemolysis in these patients despite having hemoglobin levels ≥10.0 g/dL (Fig. 2A)
Patients from all three trials demonstrated improvements in ARC, LDH Levels, and clinically significant increases in FACIT-Fatigue scores (≥3-point increase5) following 16 weeks of pegcetacoplan treatment (Fig. 2A-C)
Figure 1. Hemoglobin Levels at Baseline and Week 16 of Pegcetacoplan Treatment
a
INTRODUCTION
Figure 2. ARC, LDH, & FACIT-Fatigue at Baseline and Week 16 of Pegcetacoplan Treatment
A
B
C
Abbreviations: ARC, absolute reticulocyte count; FACIT, Functional Assessment of Chronic Illness Therapy; LDH, lactate dehydrogenase; LLN, lower limit of normal; PEG, pegcetacoplan; ULN, upper limit of normal;
a This analysis was performed using the original baseline for ECU patients; a second analysis using Week 16 as the baseline for ECU patients revealed similar results (data not shown)
Paroxysmal nocturnal hemoglobinuria (PNH) is a rare and acquired disease that involves complement-mediated hemolysis, thrombophilia, and various degrees of bone marrow failure1
Pegcetacoplan is the first therapy for PNH that targets the C3 complement protein and was approved by the U.S. FDA for treatment of adults with PNH on May 14, 2021
The efficacy and safety of pegcetacoplan has been demonstrated in patients with PNH
and screening hemoglobin levels <10.5 g/dL who:
Remained anemic despite stable eculizumab (C5-inhibitor) treatment for ≥3 months (PEGASUS Phase 3 study)2
Were naïve to treatment with complement-inhibitors (PADDOCK3 Phase 1b and
PRINCE4 Phase 3 studies)
AIMS
This post hoc analysis evaluated effect of 16 weeks of pegcetacoplan treatment on hematologic and clinical parameters among patients with PNH and baseline hemoglobin levels ≥10.0 g/dL as a population representative of patients with less severe anemia
METHODS
Post Hoc Analysis Cohorts
Adult patients with PNH from the PADDOCK, PEGASUS, and PRINCE studies with baseline screening hemoglobin levels ≥10.0 g/dL and no transfusions ≤14 days prior to measurement
Abbreviations: LLN, lower limit of normal; PEG, pegcetacoplan;
a This analysis was performed using the original baseline for ECU patients; a second analysis using Week 16 as the baseline for ECU patients revealed similar results (data not shown)
Additional Hemoglobin Outcomes
The majority of patients with baseline hemoglobin levels ≥10.0 g/dL demonstrated a hemoglobin response and hemoglobin levels ≥12 g/dL in the absence of transfusions at Week 16 after pegcetacoplan treatment initiation in all three trials (Table 2)
These results suggest that pegcetacoplan can further improve hemoglobin levels in patients with less severe anemia and baseline hemoglobin levels of ≥10.0 g/dL
| Table 1. Patient Characteristics and Post Hoc Analysis Endpoints | | | | |
| --- | --- | --- | --- | --- |
| Study | Patient Population | Analysis Cohorts (Total N) | Post Hoc N | Subcutaneous PEG Dose |
| PADDOCK NCT02588833 Phase 1b | Complement-inhibitora naïve patients | PEG treatment (N=20) | 6b | 270-360 mg/day |
| PRINCE NCT04085601 Phase 3 | Patients were complement-inhibitora naive within 3 months prior to screening | PEG treatment (N=35) | 8b | 1080 mg twice/week |
| PEGASUS NCT03500549 Phase 3 | Patients were anemic despite stable eculizumab treatment for ≥3 months (average >4 years) | PEG treatment during the RCP (N=41) | 6b | 1080 mg twice/week |
| | | Switched from eculizumab to PEG during the OLP (N=39) | 3c | |
| Endpoints Analyzed After 16 Weeks of Pegcetacoplan Monotherapy for All Cohorts | | | | |
| Change from baseline in Hb levels, ARC, LDH levels, and FACIT-Fatigue scores Number and percentage of patients who: Had a Hb response (≥1.0 g/dL Hb increase from baseline without transfusiond) Achieved Hb levels ≥12.0 g/dL in the absence of transfusiond | | | | |
Safety and Adverse Events
No deaths occurred in the ≥10.0 g/dL baseline subgroup in any of the three trials
One PEGASUS patient (4.2%) in this subgroup who was treated with pegcetacoplan during the randomized controlled period experienced a serious adverse event classified as breakthrough hemolysis that was deemed not related to pegcetacoplan and did not lead to study discontinuation (Table 3)
Adverse events of interest (such as injection site reactions and some infections and/or infestations) occurred at similar rates between the subgroups in all three trials (Table 3)
However, no encapsulated bacterial infections were observed
No thrombotic events occurred in the subgroups from the three trials (Table 3)
| Table 2. Hemoglobin Response and Patients with Hemoglobin Levels ≥12 g/dL at Week 16 | | | |
| --- | --- | --- | --- |
| Additional Hemoglobin Outcomes at Week 16 of Pegcetacoplan Treatment | PADDOCK (N=5)a | PRINCE (N=8) | PEGASUS (N=9)b |
| Patients with ≥1.0 g/dL improvement in hemoglobin levels in the absence of transfusions, n (%) | 5 (100.0) | 8 (100.0) | 7 (77.8) |
| Percentage of patients with hemoglobin ≥12.0 g/dL in the absence of transfusions, n (%) | 3 (60.0) | 7 (87.5) | 6 (66.7) |
| Mean change from baseline to Week 16 in hemoglobin levels, g/dL (SD) | 2.3 (1.1) | 2.6 (0.8) | 1.8 (2.7) |
| Table 3. Adverse Events | | | |
| --- | --- | --- | --- |
| Adverse Events | PADDOCK (N=6)a | PRINCE (N=8) | PEGASUS (N=9) |
| Any Adverse Events, n (%) | 4 (66.7) | 7 (87.5) | 7 (77.8) |
| Serious Adverse Events, n (%) | 0 (0) | 0 (0) | 1 (11.1)b |
| Adverse Events of Interest | | | |
| Injection Site Reactions, n (%) | 0 (0) | 1 (12.5) | 3 (33.3) |
| Infections and Infestationsc, n (%) | 1 (16.7) | 1 (12.5) | 3 (33.3) |
| Thrombotic Events, n (%) | 0 (0) | 0 (0) | 0 (0) |
Abbreviations: ARC, absolute reticulocyte count; FACIT, Functional Assessment of Chronic Illness Therapy; Hb, hemoglobin; LDH, lactate dehydrogenase; OLP, open-label period; PEG, pegcetacoplan; RCP, randomized controlled period
a Complement inhibitors: i.e., eculizumab, ravulizumab
b The post hoc analysis endpoint for the PADDOCK, PRINCE, and PEGASUS RCP subgroups was Week 16
c The post hoc analysis endpoint for the PEGASUS subgroup that switched to pegcetacoplan during the OLP was Week 32 (16 weeks of pegcetacoplan treatment)
d Includes any transfusion during the 16-week analysis period
Abbreviations: SD, standard deviation
a One PADDOCK patient was excluded from the hemoglobin response, hemoglobin ≥12.0 g/dL, and change from baseline analyses because they stopped pegcetacoplan treatment at Day 29 (prior to the Week 16 endpoint) and left the study due to physician decision
b This analysis was performed using the original baseline for patients who switched from eculizumab to pegcetacoplan during the open-label period at Week 17; a second analysis using Week 16 as the baseline for eculizumab patients revealed similar results (data not shown)
a One PADDOCK patient stopped pegcetacoplan treatment at Day 29 and left the study due to physician decision; but was included in this safety analysis
b The serious adverse event was documented as breakthrough hemolysis - deemed not related to pegcetacoplan and did not lead to study discontinuation
c Observed infections included: nasopharyngitis, urinary tract infection, viral infection, gastrointestinal infection, tonsillitis, vulvovaginal mycotic infection
INSERT QC CODE HERE
REFERENCES: 1. Parker C, et al. Blood. 2005;106(12):3699-3709. 2. Hillmen P, et al. N Engl J Med. 2021;384(11):1028-1037. 3. Wong RS, et al., Blood. 2020 136 [Supplement 1]. 4. Wong RS, et al., Blood. 2021 138 [November Supplement]. 5. Cella D, et al. J Pain Symptom Manage. 2002;24(6):547-561. 6. Montan I, et al. Value Health. 2018 Nov;21(11):1313-1321.
ACKNOWLEDGEMENTS: The patients who participated in the PADDOCK, PEGASUS and PRINCE studies, as well as their caregivers; Institution staff and investigators who participated in this study and for their valuable contributions; Writing and editorial support were provided by Boston Strategic Partners Inc. (supported by Apellis Pharmaceuticals, Inc.); Apellis Pharmaceuticals and Swedish Orphan Biovitrum AB reviewed the presentation; This study was supported by Apellis Pharmaceuticals, Inc.
DISCLOSURES: Panse, J: Reports consultancy, honoraria, and membership on an entity's Board of Directors or advisory committees with Blueprint Medicines, Amgen, F. Hoffmann-La Roche Ltd, MSD, Grünenthal, Bristol Myers Squibb, and Apellis Pharmaceuticals, Inc. Reports speakers bureau with Chugai Pharmaceutical Co., Pfizer, Novartis, Boehringer Ingelheim, and Alexion Pharmaceuticals, Inc. Reports membership on an entity's Board of Directors or advisory committees with Novartis, Boehringer Ingelheim, and Alexion Pharmaceuticals, Inc.; Daguindau, N & Okuyama, S: Nothing to disclose; Peffault de Latour, R: Reports consultancy, honoraria, and research funding with Novartis, Pfizer, Alexion Pharmaceuticals, Inc. Reports consulting and honoraria with Apellis Pharmaceuticals, Inc. and Swedish Orphan Biovitrum AB. Reports research funding with Amgen; Schafhausen, P: Reports honoraria and membership on an entity's Board of Directors or advisory committees with Alexion Pharmaceuticals, Inc., MSD, and Novartis. Reports membership on an entity's Board of Directors or advisory committees with Blueprint Medicines, and Swedish Orphan Biovitrum AB. Reports speakers bureau with Alexion Pharmaceuticals, Inc.; Straetmans, N: Reports membership on an entity's Board of Directors or advisory committees with Alexion Pharmaceuticals, Inc.; Al-Adhami, M: Reports current employment with Apellis Pharmaceuticals, Inc.; Ajayi, T & Yeh, M: Report current employment and current equity holder in the publicly traded-company Apellis Pharmaceuticals, Inc.; Wong, RS: Reports consultancy, honoraria, research funding, and speakers bureau with Alexion Pharmaceuticals, Inc. and F. Hoffmann-La Roche Ltd. Reports research funding and speakers bureau with Apellis Pharmaceuticals, Inc.
American Society for Hematology | December 11-14, 2021 | Atlanta, GA / Virtual
