# Supplementary material for: Improvements in hematologic markers and decreases in fatigue with pegcetacoplan for patients with paroxysmal nocturnal hemoglobinuria and mild or moderate anemia (hemoglobin ≥10 g/dL) who had received eculizumab or were naive to complement inhibitors
Source: PLoS One. 2024 Jul 29;19(7):e0306407. doi: 10.1371/journal.pone.0306407 (PMC11285951; doi:10.1371/journal.pone.0306407)
Supplement: S2 File — (PPTX) [file pone.0306407.s002.pptx]

## Slide 1
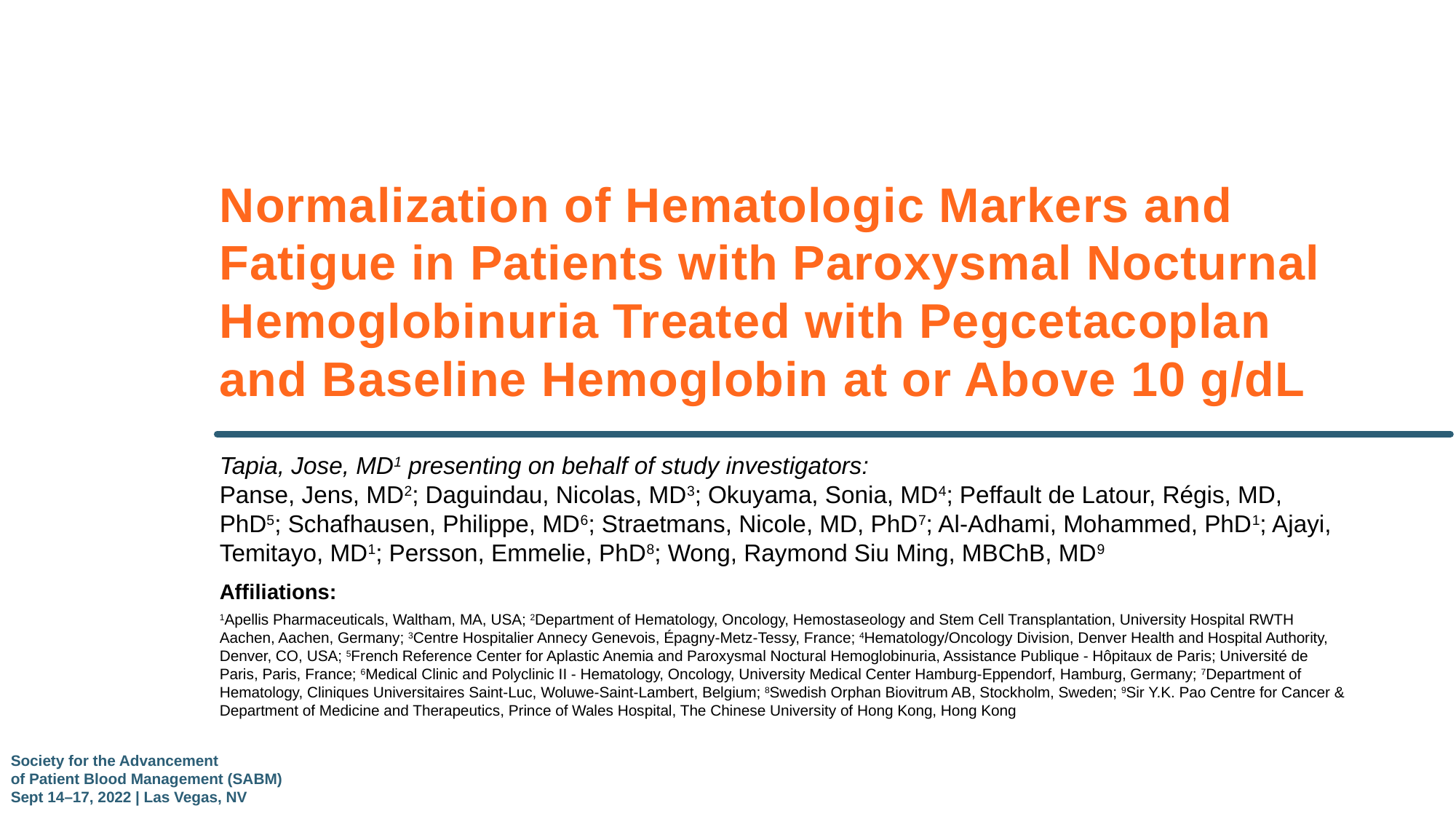

# Normalization of Hematologic Markers and Fatigue in Patients with Paroxysmal Nocturnal Hemoglobinuria Treated with Pegcetacoplan and Baseline Hemoglobin at or Above 10 g/dL
Tapia, Jose, MD1 presenting on behalf of study investigators:
Panse, Jens, MD2; Daguindau, Nicolas, MD3; Okuyama, Sonia, MD4; Peffault de Latour, Régis, MD, PhD5; Schafhausen, Philippe, MD6; Straetmans, Nicole, MD, PhD7; Al-Adhami, Mohammed, PhD1; Ajayi, Temitayo, MD1; Persson, Emmelie, PhD8; Wong, Raymond Siu Ming, MBChB, MD9
Affiliations:
1Apellis Pharmaceuticals, Waltham, MA, USA; 2Department of Hematology, Oncology, Hemostaseology and Stem Cell Transplantation, University Hospital RWTH Aachen, Aachen, Germany; 3Centre Hospitalier Annecy Genevois, Épagny-Metz-Tessy, France; 4Hematology/Oncology Division, Denver Health and Hospital Authority, Denver, CO, USA; 5French Reference Center for Aplastic Anemia and Paroxysmal Noctural Hemoglobinuria, Assistance Publique - Hôpitaux de Paris; Université de Paris, Paris, France; 6Medical Clinic and Polyclinic II - Hematology, Oncology, University Medical Center Hamburg-Eppendorf, Hamburg, Germany; 7Department of Hematology, Cliniques Universitaires Saint-Luc, Woluwe-Saint-Lambert, Belgium; 8Swedish Orphan Biovitrum AB, Stockholm, Sweden; 9Sir Y.K. Pao Centre for Cancer & Department of Medicine and Therapeutics, Prince of Wales Hospital, The Chinese University of Hong Kong, Hong Kong
Society for the Advancement
of Patient Blood Management (SABM)
Sept 14–17, 2022 | Las Vegas, NV

## Slide 2
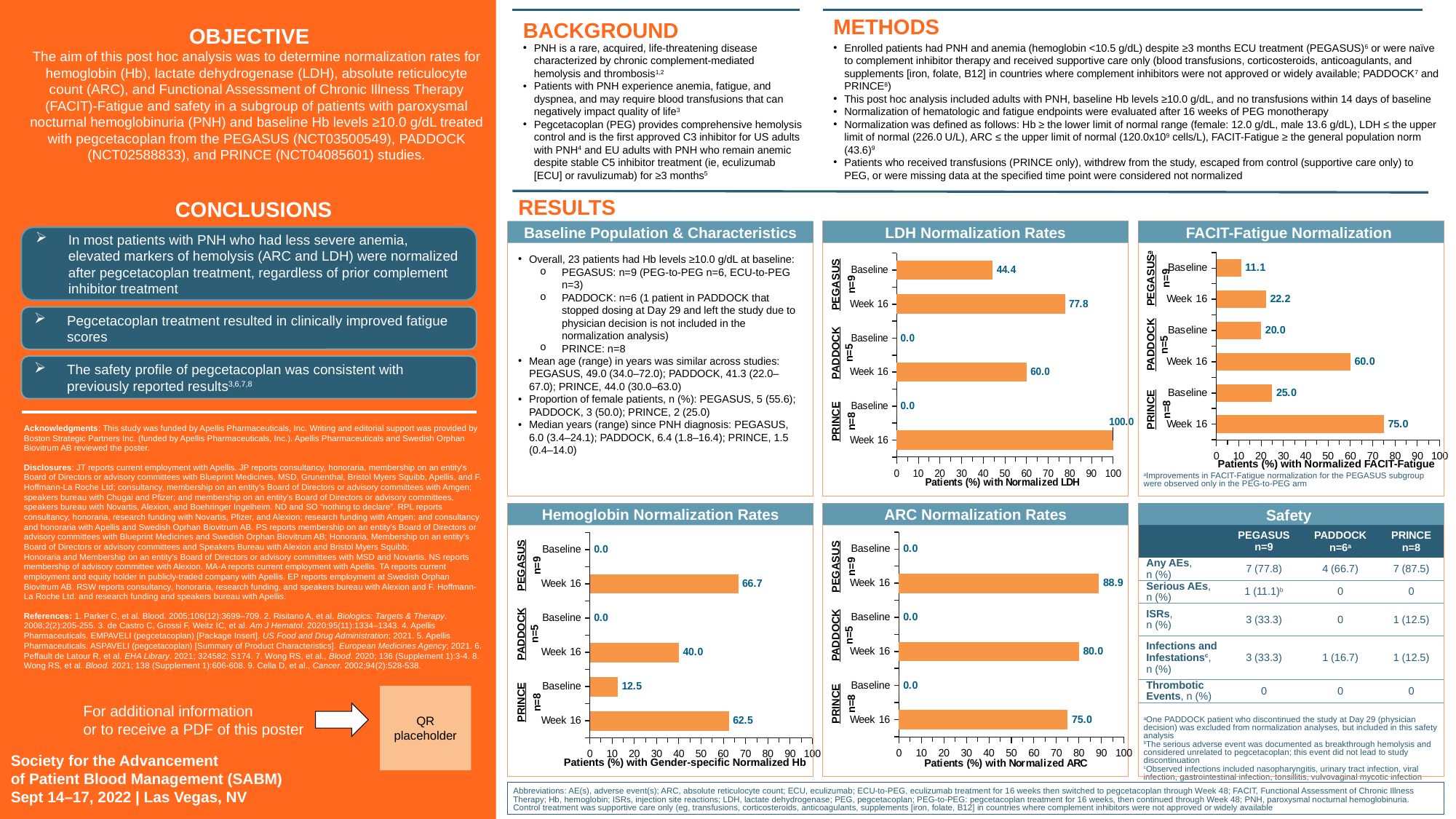

METHODS
BACKGROUND
OBJECTIVE
The aim of this post hoc analysis was to determine normalization rates for hemoglobin (Hb), lactate dehydrogenase (LDH), absolute reticulocyte count (ARC), and Functional Assessment of Chronic Illness Therapy (FACIT)-Fatigue and safety in a subgroup of patients with paroxysmal nocturnal hemoglobinuria (PNH) and baseline Hb levels ≥10.0 g/dL treated with pegcetacoplan from the PEGASUS (NCT03500549), PADDOCK (NCT02588833), and PRINCE (NCT04085601) studies.
PNH is a rare, acquired, life-threatening disease characterized by chronic complement-mediated hemolysis and thrombosis1,2
Patients with PNH experience anemia, fatigue, and dyspnea, and may require blood transfusions that can negatively impact quality of life3
Pegcetacoplan (PEG) provides comprehensive hemolysis control and is the first approved C3 inhibitor for US adults with PNH4 and EU adults with PNH who remain anemic despite stable C5 inhibitor treatment (ie, eculizumab [ECU] or ravulizumab) for ≥3 months5
Enrolled patients had PNH and anemia (hemoglobin <10.5 g/dL) despite ≥3 months ECU treatment (PEGASUS)6 or were naïve to complement inhibitor therapy and received supportive care only (blood transfusions, corticosteroids, anticoagulants, and supplements [iron, folate, B12] in countries where complement inhibitors were not approved or widely available; PADDOCK7 and PRINCE8)
This post hoc analysis included adults with PNH, baseline Hb levels ≥10.0 g/dL, and no transfusions within 14 days of baseline
Normalization of hematologic and fatigue endpoints were evaluated after 16 weeks of PEG monotherapy
Normalization was defined as follows: Hb ≥ the lower limit of normal range (female: 12.0 g/dL, male 13.6 g/dL), LDH ≤ the upper limit of normal (226.0 U/L), ARC ≤ the upper limit of normal (120.0x109 cells/L), FACIT-Fatigue ≥ the general population norm (43.6)9
Patients who received transfusions (PRINCE only), withdrew from the study, escaped from control (supportive care only) to PEG, or were missing data at the specified time point were considered not normalized
RESULTS
CONCLUSIONS
Baseline Population & Characteristics
LDH Normalization Rates
FACIT-Fatigue Normalization
In most patients with PNH who had less severe anemia, elevated markers of hemolysis (ARC and LDH) were normalized after pegcetacoplan treatment, regardless of prior complement inhibitor treatment
### Chart
| Category | Column1 |
|---|---|
| Week 16 | 100.0 |
| Baseline | 0.0 |
| Week 16 | 60.0 |
| Baseline | 0.0 |
| Week 16 | 77.8 |
| Baseline | 44.4 |
### Chart
| Category | Column1 |
|---|---|
| Week 16 | 75.0 |
| Baseline | 25.0 |
| Week 16 | 60.0 |
| Baseline | 20.0 |
| Week 16 | 22.2 |
| Baseline | 11.1 |Overall, 23 patients had Hb levels ≥10.0 g/dL at baseline:
PEGASUS: n=9 (PEG-to-PEG n=6, ECU-to-PEG n=3)
PADDOCK: n=6 (1 patient in PADDOCK that stopped dosing at Day 29 and left the study due to physician decision is not included in the normalization analysis)
PRINCE: n=8
Mean age (range) in years was similar across studies: PEGASUS, 49.0 (34.0–72.0); PADDOCK, 41.3 (22.0–67.0); PRINCE, 44.0 (30.0–63.0)
Proportion of female patients, n (%): PEGASUS, 5 (55.6); PADDOCK, 3 (50.0); PRINCE, 2 (25.0)
Median years (range) since PNH diagnosis: PEGASUS, 6.0 (3.4–24.1); PADDOCK, 6.4 (1.8–16.4); PRINCE, 1.5 (0.4–14.0)
PEGASUSa
n=9
PEGASUS
n=9
PADDOCK
n=5
Pegcetacoplan treatment resulted in clinically improved fatigue scores
PADDOCK
n=5
The safety profile of pegcetacoplan was consistent with previously reported results3,6,7,8
PRINCE
n=8
PRINCE
n=8
Acknowledgments: This study was funded by Apellis Pharmaceuticals, Inc. Writing and editorial support was provided by Boston Strategic Partners Inc. (funded by Apellis Pharmaceuticals, Inc.). Apellis Pharmaceuticals and Swedish Orphan Biovitrum AB reviewed the poster.
Disclosures: JT reports current employment with Apellis. JP reports consultancy, honoraria, membership on an entity's Board of Directors or advisory committees with Blueprint Medicines, MSD, Grunenthal, Bristol Myers Squibb, Apellis, and F. Hoffmann-La Roche Ltd; consultancy, membership on an entity's Board of Directors or advisory committees with Amgen; speakers bureau with Chugai and Pfizer; and membership on an entity's Board of Directors or advisory committees, speakers bureau with Novartis, Alexion, and Boehringer Ingelheim. ND and SO “nothing to declare”. RPL reports consultancy, honoraria, research funding with Novartis, Pfizer, and Alexion; research funding with Amgen; and consultancy and honoraria with Apellis and Swedish Oprhan Biovitrum AB. PS reports membership on an entity's Board of Directors or advisory committees with Blueprint Medicines and Swedish Orphan Biovitrum AB; Honoraria, Membership on an entity's Board of Directors or advisory committees and Speakers Bureau with Alexion and Bristol Myers Squibb; Honoraria and Membership on an entity's Board of Directors or advisory committees with MSD and Novartis. NS reports membership of advisory committee with Alexion. MA-A reports current employment with Apellis. TA reports current employment and equity holder in publicly-traded company with Apellis. EP reports employment at Swedish Orphan Biovitrum AB. RSW reports consultancy, honoraria, research funding, and speakers bureau with Alexion and F. Hoffmann-La Roche Ltd. and research funding and speakers bureau with Apellis.
References: 1. Parker C, et al. Blood. 2005;106(12):3699–709. 2. Risitano A, et al. Biologics: Targets & Therapy. 2008;2(2):205-255. 3. de Castro C, Grossi F, Weitz IC, et al. Am J Hematol. 2020;95(11):1334–1343. 4. Apellis Pharmaceuticals. EMPAVELI (pegcetacoplan) [Package Insert]. US Food and Drug Administration; 2021. 5. Apellis Pharmaceuticals. ASPAVELI (pegcetacoplan) [Summary of Product Characteristics]. European Medicines Agency; 2021. 6. Peffault de Latour R, et al. EHA Library. 2021; 324582; S174. 7. Wong RS, et al., Blood. 2020; 136 (Supplement 1):3-4. 8. Wong RS, et al. Blood. 2021; 138 (Supplement 1):606-608. 9. Cella D, et al., Cancer. 2002;94(2):528-538.
Patients (%) with Normalized FACIT-Fatigue
aImprovements in FACIT-Fatigue normalization for the PEGASUS subgroup were observed only in the PEG-to-PEG arm
Safety
Hemoglobin Normalization Rates
ARC Normalization Rates
| | PEGASUS n=9 | PADDOCK n=6a | PRINCE n=8 |
| --- | --- | --- | --- |
| Any AEs, n (%) | 7 (77.8) | 4 (66.7) | 7 (87.5) |
| Serious AEs, n (%) | 1 (11.1)b | 0 | 0 |
| ISRs, n (%) | 3 (33.3) | 0 | 1 (12.5) |
| Infections and Infestationsc, n (%) | 3 (33.3) | 1 (16.7) | 1 (12.5) |
| Thrombotic Events, n (%) | 0 | 0 | 0 |
### Chart
| Category | Column1 |
|---|---|
| Week 16 | 75.0 |
| Baseline | 0.0 |
| Week 16 | 80.0 |
| Baseline | 0.0 |
| Week 16 | 88.9 |
| Baseline | 0.0 |
### Chart
| Category | Column1 |
|---|---|
| Week 16 | 62.5 |
| Baseline | 12.5 |
| Week 16 | 40.0 |
| Baseline | 0.0 |
| Week 16 | 66.7 |
| Baseline | 0.0 |PEGASUS
n=9
PEGASUS
n=9
PADDOCK
n=5
PADDOCK
n=5
QR placeholder
PRINCE
n=8
PRINCE
n=8
For additional information
or to receive a PDF of this poster
aOne PADDOCK patient who discontinued the study at Day 29 (physician decision) was excluded from normalization analyses, but included in this safety analysis
bThe serious adverse event was documented as breakthrough hemolysis and considered unrelated to pegcetacoplan; this event did not lead to study discontinuation
cObserved infections included nasopharyngitis, urinary tract infection, viral infection, gastrointestinal infection, tonsillitis, vulvovaginal mycotic infection
Society for the Advancement
of Patient Blood Management (SABM)
Sept 14–17, 2022 | Las Vegas, NV
Patients (%) with Gender-specific Normalized Hb
Abbreviations: AE(s), adverse event(s); ARC, absolute reticulocyte count; ECU, eculizumab; ECU-to-PEG, eculizumab treatment for 16 weeks then switched to pegcetacoplan through Week 48; FACIT, Functional Assessment of Chronic Illness Therapy; Hb, hemoglobin; ISRs, injection site reactions; LDH, lactate dehydrogenase; PEG, pegcetacoplan; PEG-to-PEG: pegcetacoplan treatment for 16 weeks, then continued through Week 48; PNH, paroxysmal nocturnal hemoglobinuria. Control treatment was supportive care only (eg, transfusions, corticosteroids, anticoagulants, supplements [iron, folate, B12] in countries where complement inhibitors were not approved or widely available
