# Supplementary material for: Improvements in hematologic markers and decreases in fatigue with pegcetacoplan for patients with paroxysmal nocturnal hemoglobinuria and mild or moderate anemia (hemoglobin ≥10 g/dL) who had received eculizumab or were naive to complement inhibitors
Source: PLoS One. 2024 Jul 29;19(7):e0306407. doi: 10.1371/journal.pone.0306407 (PMC11285951; doi:10.1371/journal.pone.0306407)
Supplement: S4 File — (PDF) [file pone.0306407.s004.pdf]

DocuSign Envelope ID: 4E48AFE9-1161-4C0E-AFC1-6F60772C3D6B

16.1.5 SIGNATURES INVESTIGATORS

PRINCIPAL OR COORDINATING INVESTIGATOR(S) SIGNATURE(S)

OR SPONSOR'S RESPONSIBLE MEDICAL OFFICER

**STUDY TITLE:**	APL2-308 (PRINCE)<br>A Phase 3, Randomized, Multicenter, Open-Label, Controlled Study to Evaluate the Efficacy and Safety of Pegcetacoplan in Patients with Paroxysmal Nocturnal Hemoglobinuria (PNH)
**STUDY AUTHOR(S):**	Apellis Pharmaceuticals, Inc.

I have read this report and confirm that to the best of my knowledge it accurately describes the conduct and results of the study

INVESTIGATOR OR SPONSOR'S RESPONSIBLE MEDICAL OFFICER:

Dr. Raymond Wong

The Chinese University of Hong Kong

Raymond Wong

DocuSigned by:
Dr. Raymond Wong
Signer Name: Dr. Raymond Wong
Signing Reason: I approve this document
Signing Time: 28-Oct-2021 | 11:01 EDT
404F72E852D34B3A85123F03AF453CF1

28-Oct-2021 | 11:01 EDT

(Printed Name, Affiliation) (Signature) (Date)
