# Supplementary material for: Improvements in hematologic markers and decreases in fatigue with pegcetacoplan for patients with paroxysmal nocturnal hemoglobinuria and mild or moderate anemia (hemoglobin ≥10 g/dL) who had received eculizumab or were naive to complement inhibitors
Source: PLoS One. 2024 Jul 29;19(7):e0306407. doi: 10.1371/journal.pone.0306407 (PMC11285951; doi:10.1371/journal.pone.0306407)
Supplement: S5 File — (PDF) [file pone.0306407.s005.pdf]

16.1.3 LIST OF IRBS AND/OR IECS, SAMPLE CONSENT FORMS AND WRITTEN SUBJECT INFORMATION

16.1.3.1 List of IRBs and/or IECs

Central IRBs/IECs	Investigator	Site Number
Northern A Health and Disability Ethics Committee<br>Freyberg Building<br>20 Aitken Street<br>PO Box 5013<br>Wellington 6011<br>New Zealand	Dr. Humphrey W Pullon<br>Department of Haematology<br>Waikato Hospital<br>Pembroke Street<br>Hamilton 3240<br>New Zealand	2301
Northern A Health and Disability Ethics Committee<br>Freyberg Building<br>20 Aitken Street<br>PO Box 5013<br>Wellington 6011<br>New Zealand	Dr. Denis O'Keeffe<br>Department of Haematology<br>Waikato Hospital<br>Pembroke Street<br>Hamilton 3240<br>New Zealand	2301
**Chairperson:**	Dr. Brian Fergus	
Northern A Health and Disability Ethics Committee<br>Freyberg Building<br>20 Aitken Street<br>PO Box 5013<br>Wellington 6011<br>New Zealand	Dr. R Spearing<br>Canterbury Regional Cancer & Haematology Service<br>524 Hagley Avenue<br>Christchurch 8011<br>New Zealand	2302
Northern A Health and Disability Ethics Committee<br>Freyberg Building<br>20 Aitken Street<br>PO Box 5013<br>Wellington 6011<br>New Zealand	Dr. P Ganly<br>Canterbury Regional Cancer & Haematology Service<br>524 Hagley Avenue<br>Christchurch 8011<br>New Zealand	2302
**Chairperson:**	Dr. Brian Fergus	
Advarra IRB	Dr. Eloy Roman<br>Lakes Research	3201

Central IRBs/IECs	Investigator	Site Number
6940 Columbia Gateway Drive, Suite 110<br>Columbia, MD 21046 USA	5801 NW 151 Street,<br>Suite #302,<br>Miami Lakes,<br>FL 33014<br>US	
**Chairperson:**	Sara Harnish, J.D., Executive Chair	
Human Research Protection Unit, Faculty of Medicine Siriraj Hospital, Mahidol University	Dr. Surapol Issaragrisil	2401
**Chairperson:**	Siriporn Pitimana-aree	
Ethical Clearance Committee on Human Rights Related to Research Involving Human Subjects Faculty of Medicine Ramathibodi Hospital, Mahidol University	Dr. Pimjai Niparuck	2402
**Chairperson:**	Chusak Okascharoen	
Institutional Review Board, Royal Thai Army Medical Department	Dr. Numbenjapon	2403
**Chairperson:**	Col. Suthee Panichkul	

Local IRBs/IECs	Investigator	Site Number
Medical Research & Ethics Committee (MREC)	Dr. Jameela Sathar	2501
**Chairperson:**	Dr. Hjh Salina Abdul Aziz	
Institutional Review Board of the University of Hong Kong/Hospital Authority Hong Kong West Cluster	Dr. Eric Tse	2601

Local IRBs/IECs	Investigator	Site Number
**Chairperson:**	Prof. Sydney Tang	
The Joint Chinese University of Hong Kong – New Territories East Cluster Clinical Research Ethics Committee	Dr. Raymond Wong	2602
**Chairperson:**	Prof. Benny C.Y. Zee	

16.1.3.2 Sample Informed Consent Forms

The Informed Consent forms listed below are provided on the following pages:

- APL2-CP-PNH-204 - Master Informed Consent NZ V1 - 13Oct15
- APL2-CP-PNH-204 - Master Informed Consent NZ V2 - 10Jun16
- APL2-CP-PNH-204 - Master Informed Consent NZ V3 - 11Oct16
- APL2-CP-PNH-204 - Master Informed Consent NZ V4 - 14Aug17
- APL2-CP-PNH-204 - Master Informed Consent Asia V1 - 2May17
- APL2-CP-PNH-204 - Master Informed Consent Asia V2 - 31Aug17
- APL2-CP-PNH-204 - Master Informed Consent Asia V3 - 12Dec17
- APL2-CP-PNH-204 - Master Informed Consent Asia V4 - 02Oct18
- APL2-CP-PNH-204 - Master Informed Consent Asia V5 - 20Dec18
- APL2-CP-PNH-204 - Master Informed Consent US V1 - 24 Jan18
- APL2-CP-PNH-204 - Master Informed Consent US V2 - 23Aug18

Insert Header with institution's name or institution's letterhead

PARTICIPANT INFORMATION SHEET

[Insert site name]

**Short Title**	A Phase Ib, Open Label, Multiple Ascending Dose, Pilot Study to Assess the Safety, Preliminary Efficacy and Pharmacokinetics of Subcutaneously Administered APL-2 in Subjects with Paroxysmal Nocturnal Hemoglobinuria (PNH). – PADDOCK		
**Protocol Number**	APL2-CP-PNH-204	**Ethics Number**	<<Insert #>>
**Project Sponsor**	Apellis Pharmaceuticals, Inc.<br>6400 Westwind Way, Suite A, Louisville, KY, 40014		
**Local Sponsor**	Clinical Network Services (CNS) Ltd<br>PO Box 78312, Grey Lynn, Auckland, 1245		
**Principal Investigator**	<<Insert Name>>		
**Location**	<<Insert Location>>		
**Study related phone number**	<<Insert Contact Number>>		

You are being invited to take part in a research study of the trial drug APL-2. This consent form gives you important information about this study to help you decide if you want to take part. It describes the purpose of this study, the study procedures, the possible risks, and provides information about your rights as a study participant.

Please take the time to read this information carefully. You should talk to your study doctor and study staff about this study and ask any questions you have. You can also discuss this study with other people such as your family/whanau or general practitioner (GP). If you decide to take part in this study, you will be asked to sign and date this form. You will receive a copy of the signed form. After you sign this form, your study doctor and study staff will do some tests to see if you meet the study requirements. Your study doctor will tell your GP about your participation in this study.

Who is funding this study?

Apellis Pharmaceuticals, Inc. ("Apellis") is funding this clinical study. Apellis is the "sponsor" of the study, which means that Apellis takes responsibility for starting, managing, and financing the study. <<Insert Site Name>> is being paid by Apellis to do this study.

Voluntary participation and withdrawal from this study

Your participation in this study is voluntary. You are free to say yes or no. Even if you join this study, you may stop at any time.

A description of this clinical trial will be available on the internet at http://www.ClinicalTrials.gov. This website will not include information that can identify you. At most, the website will include a summary of the results. You can search this website at any time.

1. WHAT YOU SHOULD KNOW ABOUT THIS STUDY

a. Why is this study being done?

This is a research study to test a new investigational drug. An investigational drug is one that is not approved by any regulatory authority (e.g. FDA, Medsafe etc) for use outside a clinical trial.

You are being invited to participate in this research study because you have been diagnosed with paroxysmal nocturnal hemoglobinuria (PNH). PNH is a rare disorder that causes the red blood cells to break down too early. Many PNH patients suffer from complications such as blood clots or low red blood cell counts (anemia) even after receiving standard treatment.

The purpose of this research study is to gather scientific information about the safety, tolerability and initial evidence of activity of the investigational drug APL-2 in people with PNH who have not been treated with Soliris® {an approved medication (not available in NZ) that blocks the breakdown of red blood cells} in the past. The study will help determine how APL-2 behaves inside the body, how it is eliminated from the body, the effect it has on the body after multiple doses and if it can prevent red blood cells from breaking down.

b. How does APL-2 works?

Your body has numerous biological mechanisms designed to attack and kill off invaders, such as bacteria. One of these mechanisms, called the complement cascade, is part of your normal immune system. Normally, only invaders are destroyed by the complement cascade as your own cells are protected against its attacks. However, patients with PNH possess red blood cells that are defective and are not properly protected, which results in the destruction of these defective red blood cells by your own immune system. The breakdown of a large number of red blood cells in this manner has been shown to trigger episodes of PNH. Based on the research conducted prior to this Phase Ib study, the investigational study drug, which is a peptide (small protein), is expected to decrease the activity of the complement cascade and prevent the destruction of defective red blood cells. Results from the ongoing/completed studies also provided good assurance of the safety for the doses of APL-2 in this study.

c. Dosing of APL-2

APL-2 will be given daily, as a subcutaneous (the fat tissue under your skin) injection and it will be given either, by a nurse in the clinic, or by a nurse who visits your home.

There will be 2 cohorts (groups) in the study and each group will include 3 participants with PNH. Groups will be dosed sequentially i.e. group 1 will be dosed before group 2. You will not have a choice as to which group you are assigned to.

TABLE 1: Doses and injection volumes of APL-2

	Dose (Days 1 to 84)
**Group 1**	180 mg APL-2 / day
**Group 2**	To be determined but will not exceed 270 mg APL-2 / day

The decision to proceed to the next group will be agreed by a safety monitoring committee which will include <<Insert PI Name>> and other doctors involved in the study together with members of the sponsor company, Apellis. The safety monitoring committee will review all the safety data available from participants already dosed in the study and will determine if it is safe to enrol the next group of participants. The first review will take place after the first group of 3 participants have completed Day 28.

You will receive APL-2 for 28 days, if there is a clear reduction in the breakdown of the red blood cells by Day 28 you will continue to receive APL-2 for a further 56 days.

The study can be stopped at any time, based on evaluation of the side effects of the study medication.

d. Will you know whether you are receiving active treatment?

This study will be done as an open label study which means all participants will receive APL-2 and you will know exactly which group you are assigned to.

2. WHAT WILL HAPPEN IF YOU TAKE PART IN THIS STUDY

a. What will you be responsible for if you take part in this study?

If you decide to take part in this study you will be asked to sign this ethics committee-approved informed consent form and you must meet specific entry rules.

You must also agree to the following:

- Be willing and able to attend all visits at the study center and be available for the home visits.
- Immunisation is required to participate in the study. Vaccinations may be given during the screening visit if you have not received the required immunisation within 2 years of the screening visit. There immunisations will be free as they are required as part of the trial.
- Be willing to allow frequent blood tests during the study and at subsequent follow up visits.
- You will be requested to keep the 'study-supplied small freezer plugged in to a power source during your study treatment. If there is a power failure, you should contact the community nurse or the clinic site.
- Follow the instructions given by the study doctor and study staff.
- Take an antibiotic throughout the study to prevent infections.
- Have all procedures performed.
- Males must agree to no sperm donation at any time during the study.
- Females who are able to have children must agree to use an effective contraception for the duration of the study. Your study doctor can advise you on effective methods of contraception.
- Make sure you take only medications approved by a study doctor. Tell the study doctor what medications you are currently taking or have taken in the past. Also, tell him / her before starting any new medications and/or if there are changes to your current medications.

b. What types of tests or procedures will be involved with this study?

If you decide to take part in the study, some tests will be done to see if you are eligible. These tests include hepatitis B, C and HIV. If you do not want any of the tests done, you should not take part in this study. If the test results show that you do not meet the study requirements, you will not be able to start the study. If you pass the screening assessments, we will ask you to return to <<Insert Site Name>> to begin the study.

If you have not received the required immunisations within 2 year before the screening visit vaccination will be given at least 15 days before you receive the first dose of APL-2 and a booster about 2 months later (Day 57 of the study).

You will receive daily injections which will be done by the study staff when you visit the clinic site or by a trained study nurse at home.

<<Insert Site Name>> will contact your regular doctor (GP) about your study participation. If you do not want your GP told, you will not be able to take part in the study. We may need to contact your GP about any health issues or parts of your medical history that might affect you taking part in the study.

You will be given a Participant Identification Card if you participate in this study, stating the name of the study and the study doctor's contact information. **This card should be carried with you at all times** so that you can contact the study doctor at any time and so you can show it to any other doctor or dentist that you might visit while in this study.

c. What are the study details? What procedures will you have?

Your total time in this study is expected to be approximately 160 days. If more tests are needed, your study doctor may ask you to schedule additional visits which could extend your total time in the study. Your study doctor or Apellis may choose to withdraw you from this research study at any time which could decrease your total time in the study.

The study will have 3 parts:

- Part 1: You will receive APL-2 for 28 days and will include at least 8 visits to the study center and a community nurse will conduct 22 visits in your home.
- Part 2: If there is a reduction in hemolysis (breakdown of red blood cells) by Day 28 you will receive APL-2 for a further 56 days and will include at least 5 visits to the study center and a community nurse will conduct 51 visits in your home. If no reduction in hemolysis by Day 28, treatment will be stopped and you will skip Part 2 and enter Part 3 for safety follow-up visits.
- Part 3: Safety follow-up will include 4 visits to the study center.

The day you have your first dose of study drug (APL-2) is called Day 1. All other days are counted back or forward from Day 1. You will be monitored throughout the study for any changes in health. At the start of the study the <<Insert Site Name>> staff will review your medical history to ensure you are eligible for the study. Throughout the entire duration of the study, the <<Insert Site Name>> staff will review any other medications you may be taking and any potential side effects to ensure your safety.

The following activities will be performed during site visits / home visits:

**Screening**<br>**Screening Visits (2 visits) Day -30 to Day -1**
Before we can do any tests, you will be asked to give written informed consent, below are details of assessments that need to be done:<br>• Questions about your health and what medication you are taking<br>• Review of your demographic (personal) information such as date of birth, ethnicity, and race<br>• Review the study requirements and restrictions<br>• Height and weight<br>• Set of vital signs (pulse, blood pressure and breathing rate) and temperature<br>• Full physical exam<br>• An ECG is a painless test that looks at the electrical activity of your heart by putting small sticky patches on certain areas of your body while you are lying down. These patches are connected to thin wires which are again connected to a machine which will interpret the electrical activity of your heart and then print a report.<br>• Set of routine blood and urine samples, blood samples relating to safety and PNH diagnosis<br>• Blood tests for HIV, Hepatitis B and Hepatitis C Pregnancy test (females) +/- hormone test (postmenopausal females)<br>• Vaccination if required will be done during the screening period after your eligibility has been confirmed. Your doctor will discuss with you what vaccinations you may need and will also take into account vaccinations that you have previously received.
**A final decision about whether you can take part will then be made when all your screening results are available.**

**Site Visits (Day 1 onwards)**
**APL-2 dosing:**<br>• You will receive the first 3 daily doses of APL-2 (Day 1 to 3) as well as doses on Day 8, 15 and 22 at the hospital clinic. You will need to stay in the clinic for 2.5 hours after receiving the first dose of APL-2 on Day 1 so we can make sure that you are okay.

Other assessments done throughout the study:

- You will be asked what medications you are taking and if you have had any recent illnesses or changes in your condition.
- Preventative Antibiotics will be taken from Day 1 until 14 days after the last dose of APL-2
- Full physical exam
- An ECG (recording of the electrical activity of your heart).
- Set of vital signs (pulse, blood pressure and breathing rate) and temperature. If the visit includes study drug administration, vital signs will be measured before and after you receive the study drug.
- Set of routine blood and urine samples, blood samples relating to safety and PNH diagnosis
- You will be assessed for signs/symptoms of blood clots.
- Pregnancy test (for females who are able to become pregnant).
- FACIT (Fatigue Scale) is a 13 item questionnaire which you will need to complete during clinic visits on Days 1, 15, 29, 43, 71, 99 and 134.
- Monitor any side effects: you will be asked to notify of any unfavourable health or medical events, referred to as adverse events, experienced from the last visit.
- Vaccination (booster) on Day 57

Home Visits

If you participate in this study, a small freezer will be shipped directly to your home by the sponsor of this study. The study drug that will be administered to you at home will be stored in this freezer. The freezer will be locked, and only the community nurse will be able to unlock it. Please note, the community nurse is separate from the study staff at the study doctor's clinic. The community nurse will be responsible for ensuring that the freezer in your home, supplied by the study sponsor, maintains the study drug at the appropriate temperature.

Home visits will be conducted by a community nurse on study days 4-7, 9-14, 16-21, and 23-28, 30-35, 37-42, 44-56, 58-70, 72-84. The following procedures will be performed at each home visit:

- You will be asked what medications you are taking and if you have had any illnesses or changes in your condition.
- Your vital signs (temperature, resting pulse and blood pressure, respiratory rate) will be measured before and after you receive the study drug.
- You will receive a dose of the study drug.
- The injection site where the study drug was given will be assessed and the surrounding area will be checked for redness, swelling and hardness. You will be asked about pain and tenderness.
- Between Day 23 and Day 28 safety blood samples will be collected to measure LD (Lactate Dehydrogenase). LD is released from the cells into the blood when cells are damaged or destroyed.
- You will be assessed for signs/symptoms of blood clots.
- This community nurse will remain in your home with you for one hour after the injection of the study drug.
- These visits will likely take approximately 2 - 4 hours.

APL-2 dosing:

- From Day 4 to Day 84 daily doses of APL-2 will be given by a trained nurse at your home with the exception of Days 8, 15, 22, 29, 36, 43, 57 and 71 (see above).

Safety Follow-up period If you have completed Part 1 of the study and there is no reduction in the breakdown of your red blood cells by Day 28, treatment will be stopped and you will skip Part 2 and enter Part 3 for safety follow-up visits. If a reduction is shown you will receive APL-2 for a further 56 days and then enter Part 3 for safety follow-up visits.

You will be asked to return to the clinic site for follow-up visits on Days 85, 99 and 113.

After you have completed all follow-up procedures you need to return for the Exit Visit on Day 134.

You will not receive any study drug at these visits. These visits will include the following tests and procedures:

- You will be asked what medications you are taking and if you have had any illnesses or changes in your condition.
- Your vital signs (temperature, resting pulse and blood pressure, respiratory rate) will be measured.
- You will be assessed for signs/symptoms of blood clots.
- Electrocardiogram (ECG) – Day 85 and 134.
- Physical examination – Day 85 and 134.
- Collection of a urine sample for routine lab tests.
    - For women who are able to get pregnant, urine will be collected at Day 99 and 134 for a pregnancy test.
- Collection of blood.
- You will be asked to complete a Fatigue Scale questionnaire at Day 99 and 134.

3. WHAT WILL HAPPEN TO MY TEST SAMPLES

The following blood samples will be collected throughout the study:

- Samples to determine regular routine blood counts and blood chemistry, to monitor your general health. (Haematology and Chemistry)
- Blood tests for HIV, Hepatitis B and Hepatitis C (Serology)
- Blood tests to determine how quickly your blood clots (Coagulation Profile)
- Pregnancy Test (If applicable)

All these routine samples will be sent to a Central lab overseas for testing and destroyed at the end of the study by internationally accepted means e.g. incineration.

The following blood samples will also be collected throughout the study. These samples will be sent to different locations for testing.

- Blood samples which determine the level of study drug in your blood (Pharmacokinetics).
- Blood samples to determine the effects of study drug on your body (Pharmacodynamics).
- Blood samples for antigenicity (The ability to cause the production of antibodies to the study drug).
- Blood samples for cytometry testing (This test is able to identify the type/amount of cells in different parts of the cell cycle).

These blood samples may be stored for up to 1 year after the end of treatment after which time they will be destroyed by internationally accepted means e.g. incineration.

The maximum amount of blood collected from each participant during the study will be approximately 341 mls (Approximately 23 tablespoons).

All samples will be coded; identified only by each participant's study number and initials. Only hospital and laboratory staff will have access to the blood samples.

The proposed blood tests include a screening test for HIV (also called the 'AIDS' virus) and viruses causing hepatitis. You will receive information and counselling before the test. If a test shows you have HIV, you will have follow-up counselling and medical advice. Signing the consent form means that you agree to have this testing; it will not be done without your consent.

<u>Additional information if you are from Maori descent:</u>

We understand that many Maori consider their tissue to be tapu and that participation in this type of study requires careful consideration. You may wish to discuss the study with your whanau or obtain the blessing of your iwi for participation in this research. They may have a position on participation in these types of studies and issues such as sending blood samples overseas and access to results of the study. Should you have any concerns regarding appropriate practice/tikanga to address cultural issues arising from your participation in the study it is recommended you consult with a kaumatua.

4. SAFETY: POTENTIAL RISKS AND DISCOMFORTS

a. Are there any risks from this study?

There may be risks to being in this study from APL-2 or from some of the procedures or tests done in this study.

If you take part in this study, you or your family members should tell the study doctor or <<Insert Site Name>> staff immediately if you have any unusual health problems, injuries, or side effects, even if you do not think these problems are caused by the study or APL-2.

If there is any new important safety information or other information that could affect your willingness to participate in this study, your study doctor will let you know.

b. What are the likely risks with APL-2?

You may feel discomfort during some of the tests and study procedures. Possible risks, side effects, and discomforts include:

- Infections – you may be at increased risk for infections caused by certain types of organisms such as Streptococcus pneumonia, Neisseria Meningitidis, or Haemophilus influenza.
- Allergic reactions – all drugs have a potential risk of an allergic reaction, which if not treated promptly, could become life-threatening.
- Blood samples - you may have discomfort or pain when your blood is collected. You may feel faint or pass out. There is also a slight possibility of infection, bruising, or bleeding at the puncture site. Your anaemia (low red blood cell count) could worsen from having frequent blood draws
- ECG - skin irritation is rare but could occur during an ECG from the electrodes or gel that is used
- Antibiotic therapy – there is a possibility that you will experience some side effects from the antibiotic treatment you will need to take during this study. Side effects such as nausea, sickness, diarrhoea and skin rashes may occur and if you experience any of these you should inform the nurse or doctor. The doctor may decide that you should change to a different antibiotic to try and reduce these symptoms.
- Injection site reactions - there is a possibility that you could experience redness, swelling, and pain or tenderness at the site of injection. There is also a slight possibility of infection.
- Vaccination is required: Like any medication, vaccines, can cause side effects. The most common side effects are mild. The side effects associated with getting vaccines are almost always mild (such as redness and swelling where the shot was given) and go away within a few days.

c. Any unforeseen risks?

Since the study drug APL-2 is investigational when taken alone or in combination with other

medications, there may be other risks that are unknown.

d. Could APL-2 be harmful to an unborn or breastfed baby?

You should discuss pregnancy risks with your doctor before signing this consent form. Women who are pregnant, planning to become pregnant or breast feeding may not participate in this research study. Taking the study drug may involve unknown risks to a pregnant woman, an embryo, fetus (unborn baby) or nursing infant. If you (or your partner) become pregnant while in this study, the sponsor may ask to follow the outcome of the pregnancy. The study drug will be stopped and your participation in this study will be ended.

Before starting this research study and during the study, females able to have children will have pregnancy tests done. Talk to your doctor about the best method of birth control to use while you are in this study. It is important that you call your study doctor at <<Insert Number>> right away if you become pregnant or father a child during the course of this study.

5. BENEFITS OF PARTICIPATION

Are there any benefits to taking part in this study?

You may or may not benefit from participation in this study. There is no guarantee that you will benefit from participating in this study. The information from this study might help to develop better treatments in the future for people with PNH. An indirect benefit of taking part in this study is that you will receive free medical tests at screening and during the study.

What are the alternatives to taking part? Do you have any other choices?

You do not have to be in this study to receive treatment for your PNH. You have the option to receive standard treatment for PNH. Standard treatment may include medications such as prednisone, or blood thinners; blood transfusions; or bone marrow transplantation. The study doctor will discuss with you the risks and benefits of the alternative treatments.

6. POTENTIAL COSTS/ REIMBURSEMENTS

There will be no charge to you for your participation in this study. The study drug, study-related procedures, and study visits will be provided at no charge to you.

Will you be paid for your samples (such as blood or urine) or medical information?

You will not be paid for the use of your samples or any information, discoveries or inventions obtained from your samples or medical information.

You will be reimbursed for any reasonable travel, accommodation, parking, meals and other expenses associated with the research project visit, this will based on the distance that you have to travel to the hospital.

7. WHAT ARE THE RIGHTS OF PARTICIPANTS IN THIS STUDY?

a. Participation is voluntary
Participation in any research project is voluntary. If you do not wish to take part, you do not have to. If you decide to take part and later change your mind, you are free to withdraw from the project at any stage. This will not affect your routine treatment, your relationship with those treating you or with <<Insert Site Name>>.
If you do decide to take part, you will be given this Participant Information and Consent Form to sign and you will be given a copy to keep.

b. New information
Sometimes during the course of a research project, new information becomes available about the treatment that is being studied. If this happens, your study doctor will tell you about it and discuss with you whether you want to continue in the research project. If you decide to withdraw, your study

doctor will make arrangements for your regular health care to continue. If you decide to continue in the research project you may be asked to sign an updated consent form.

Also, on receiving new information, your study doctor might consider it to be in your best interests to withdraw you from the research project. If this happens, he/ she will explain the reasons and arrange for your regular health care to continue.

c. Right to access information collected during the study

In accordance with relevant New Zealand privacy and other relevant laws, you have the right to request access to your information collected and stored by the research team. You also have the right to request that any information with which you disagree be corrected. Please contact the study team member named at the end of this document if you would like to access your information.

8. CONFIDENTIALITY

By signing the consent form you consent to the study doctor and relevant research staff collecting and using personal information about you for the research project. Any information obtained in connection with this research project that can identify you will remain confidential. Your name, address or phone number will not be on the study forms; instead you will be identified by a unique study number, date of birth and initials. Your information will only be used for the purpose of this research project and it will only be disclosed with your permission, except as required by law.

Information about you may be obtained from your health records held at this and other health services for the purpose of this research. By signing the consent form you agree to the study team accessing health records if they are relevant to your participation in this research project.

Your health records and any information obtained during the research project are subject to inspection (for the purpose of verifying the procedures and the data) by the relevant authorities (such as the Ethics Committee that has reviewed and approved this study) and authorised representatives of the Sponsor, Apellis, and <<Insert Site Name>>, or as required by law. All personnel reviewing your medical records will be required to keep the information confidential in accordance with law. By signing the Consent Form, you authorise release of, or access to, this confidential information to the relevant study personnel and regulatory authorities as noted above.

When the research project ends the study data must be analysed, so the results of the study may not be available until about six months after the research finishes. The study doctors and/or Apellis may decide to discuss or publish the results of the study. This may include publication in journals, presentation at conferences or other professional forums. In any publication, information will be provided in such a way that you cannot be identified. Results of the study will be provided to you, if you wish.

Information about your participation in this research project may be recorded in your health records.

Any information obtained for the purpose of this research project that can identify you will be treated as confidential and securely stored. It will be disclosed only with your permission, or as required by law.

Upon completion of the trial, the study file containing information about you will be archived for a minimum of fifteen years.

9. COMPENSATION FOR INJURY

If you were injured as a result of treatment given as part of this study, which is unlikely, you won't be eligible for compensation from ACC. However, compensation would be available from the study's sponsor, Apellis, in line with industry guidelines. We can give you a copy of these guidelines if you wish.

If you have private health or life insurance, you may wish to check with your insurer that taking part in this study won't affect your cover.

10. WHAT WILL HAPPEN AFTER THE STUDY ENDS, OR IF YOU PULL OUT?

If you decide to withdraw
Please notify a member of the research team before you withdraw. This notice will allow that person or the research supervisor to discuss any health risks or special requirements linked to withdrawing, such as follow up visits.

If you do withdraw your consent during the research project, information already collected will be retained to ensure that the results of the research project can be measured properly and to comply with law. You should be aware that data collected by the sponsor up to the time you withdraw will form part of the research project results. If you do not want them to do this, you must tell them before you join the research project.

Why the study might be unexpectedly stopped
This research project may be stopped unexpectedly for a variety of reasons. These may include reasons such as:
• Unacceptable side effects
• The drug being shown not to be effective
• The drug being shown to work and not need further testing.

Will the study treatment continue to be available after the study finished?

If it is found the drug does have a beneficial effect in reducing haemolysis (breakdown of red blood cells), and no safety concerns are raised, the company may, at its sole discretion, after consultation with the Health and Disability Ethics Committees (HDECs) and the Health Research Council (HRC) of New Zealand's Standing Committee on Therapeutic Trials (SCOTT), elect to continue the drug therapy, either by including patients, like yourself, in a further treatment cohort, or by commencing an extension study.

Where can you go for more information about the study, or to raise concerns or complaints?

If you have any questions, concerns or complaints about the study at any stage, you can contact:

<Insert PI Name>>, lead study doctor
Phone: <<Insert Number>>
Research Coordinator: <<Insert Number>>

If you want to talk to someone who isn't involved with the study, you can contact an independent health and disability advocate. Specialist advocates, are available here.

Phone: 0800 555 050
Fax: 0800 2 SUPPORT (0800 2787 7678)
Email: advocacy@hdc.org.nz

You can also contact the health and disability ethics committee (HDEC) that approved this study on:

Phone: 0800 4 ETHICS (438 442)
Email: hdecs@moh.govt.nz

For Maori health support, or to discuss any concerns or issues regarding this study, please contact Maori Health team, Phone: [insert number].

12. DO I HAVE TO DECIDE STRAIGHT AWAY?

No, you do not have to decide straight away. You should take some time to consider whether or not to participate. Study staff will let you know when they will need your decision. The following steps are useful in helping you reach a decision.

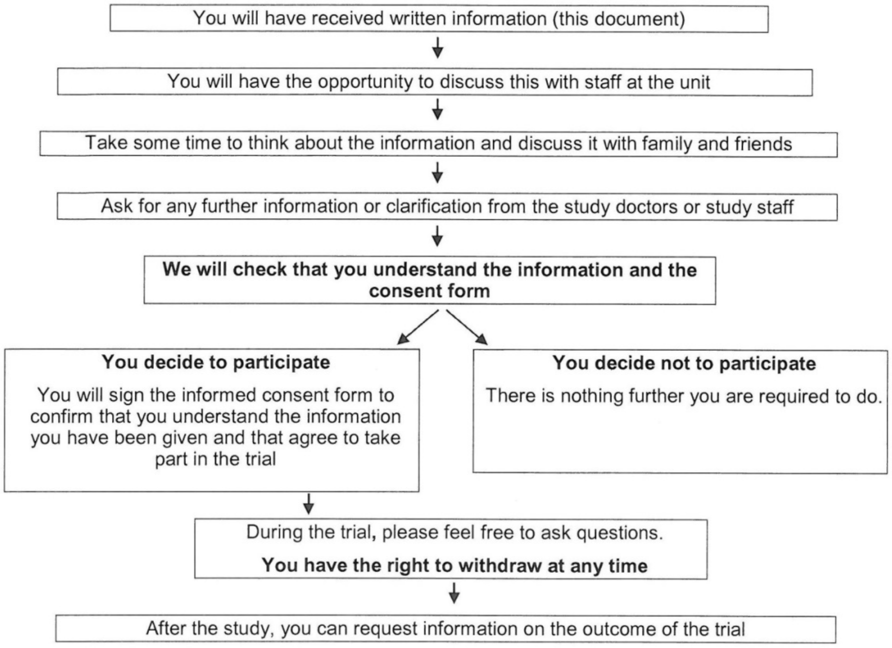
You will have received written information (this document)
You will have the opportunity to discuss this with staff at the unit
Take some time to think about the information and discuss it with family and friends
Ask for any further information or clarification from the study doctors or study staff
We will check that you understand the information and the consent form
You decide to participate
You will sign the informed consent form to confirm that you understand the information you have been given and that agree to take part in the trial
You decide not to participate
There is nothing further you are required to do.
During the trial, please feel free to ask questions.
You have the right to withdraw at any time
After the study, you can request information on the outcome of the trial

Insert Header with institution's name or institution's letterhead

INFORMED CONSENT FORM

**Short Title**	Nocturnal Hemoglobinuria (PNH) – PADDOCK
**Protocol Number**	APL2-CP-PNH-204
**Principal Investigator**	<<Insert PI Name>>

Declaration by participant:
I have read and I understand this version of the Participant Information Sheet. I have had the opportunity to ask questions and I am satisfied with the answers I have received.

I have had the opportunity to discuss the study with my family/whanau.

I freely agree to participate in this study and understand that I may withdraw from the study at any time.

I understand that:

- My participation in this study is confidential and I will not be identified in study reports.
- My medical records may be reviewed by Apellis, companies working with them, auditors and health or regulatory agencies for the purpose of checking the accuracy of the information recorded for the study
- Some of my samples will be sent overseas for analysis and they will be destroyed as described in the Information Sheet.
- If I decide to withdraw from the study, information already collected may continue to be processed. I may be asked to attend follow-up visits.
- The study drugs or tests will be stopped if they appear harmful to me.
- I should contact site staff if I have any side effects.
- My GP and/or specialist will be informed of my participation in this study and relevant medical records may be accessed.
- I am not giving up any of my legal rights by signing this consent form.
- This trial is being carried out for the principal benefit of Apellis. If I suffer an injury as a result of my participation in the trial, they will pay me compensation in line with industry guidelines.
- I understand that there may be risks associated with the treatment in the event of myself or my partner becoming pregnant. I undertake to inform my partner of the risks and to take responsibility for the prevention of pregnancy

Statement by Participant I hereby consent to take part in this study. I understand that I will receive a signed copy of this consent form for my records.

________________________________ (full name)

________________________________ (signature)

__ __ / __ __ __ / __ __ (Date)

Statement by Consenter (Investigator/designee) I have discussed this study with the above named participant. The participant appeared to fully understand the information provided about the study.

________________________________ (full name)

________________________________ (signature)

__ __ / __ __ __ / __ __ (Date) __ __ :__ __ (Time)

Insert Header with institution's name or institution's letterhead

PARTICIPANT INFORMATION SHEET

[Insert site name]

**Short Title**	A Phase Ib, Open Label, Multiple Ascending Dose, Pilot Study to Assess the Safety, Preliminary Efficacy and Pharmacokinetics of Subcutaneously Administered APL-2 in Subjects with Paroxysmal Nocturnal Hemoglobinuria (PNH). – PADDOCK		
**Protocol Number**	APL2-CP-PNH-204	**Ethics Number**	<<Insert #>>
**Project Sponsor**	Apellis Pharmaceuticals, Inc.<br>6400 Westwind Way, Suite A, Louisville, KY, 40014		
**Local Sponsor**	Clinical Network Services (CNS) Ltd<br>PO Box 78312, Grey Lynn, Auckland, 1245		
**Principal Investigator**	<<Insert Name>>		
**Location**	<<Insert Location>>		
**Study related phone number**	<<Insert Contact Number>>		

You are being invited to take part in a research study of the trial drug APL-2. This consent form gives you important information about this study to help you decide if you want to take part. It describes the purpose of this study, the study procedures, the possible risks, and provides information about your rights as a study participant.

Please take the time to read this information carefully. You should talk to your study doctor and study staff about this study and ask any questions you have. You can also discuss this study with other people such as your family/whanau or general practitioner (GP). If you decide to take part in this study, you will be asked to sign and date this form. You will receive a copy of the signed form. After you sign this form, your study doctor and study staff will do some tests to see if you meet the study requirements. Your study doctor will tell your GP about your participation in this study.

Who is funding this study?

Apellis Pharmaceuticals, Inc. ("Apellis") is funding this clinical study. Apellis is the "sponsor" of the study, which means that Apellis takes responsibility for starting, managing, and financing the study. <<Insert Site Name>> is being paid by Apellis to do this study.

Voluntary participation and withdrawal from this study

Your participation in this study is voluntary. You are free to say yes or no. Even if you join this study, you may stop at any time.

A description of this clinical trial will be available on the internet at http://www.ClinicalTrials.gov. This website will not include information that can identify you. At most, the website will include a summary of the results. You can search this website at any time.

1. WHAT YOU SHOULD KNOW ABOUT THIS STUDY

a. Why is this study being done?

This is a research study to test a new investigational drug. An investigational drug is one that is not approved by any regulatory authority (e.g. FDA, Medsafe etc) for use outside a clinical trial.

You are being invited to participate in this research study because you have been diagnosed with paroxysmal nocturnal hemoglobinuria (PNH). PNH is a rare disorder that causes the red blood cells to break down too early**.** Many PNH patients suffer from complications such as blood clots or low red blood cell counts (anemia) even after receiving standard treatment.

The purpose of this research study is to gather scientific information about the safety, tolerability and initial evidence of activity of the investigational drug APL-2 in people with PNH who have not been treated with Soliris® {an approved medication (not available in NZ) that blocks the breakdown of red blood cells} in the past. The study will help determine how APL-2 behaves inside the body, how it is eliminated from the body, the effect it has on the body after multiple doses and if it can prevent red blood cells from breaking down.

b. How does APL-2 works?

Your body has numerous biological mechanisms designed to attack and kill off invaders, such as bacteria. One of these mechanisms, called the complement cascade, is part of your normal immune system. Normally, only invaders are destroyed by the complement cascade as your own cells are protected against its attacks. However, patients with PNH possess red blood cells that are defective and are not properly protected, which results in the destruction of these defective red blood cells by your own immune system. The breakdown of a large number of red blood cells in this manner has been shown to trigger episodes of PNH. Based on the research conducted prior to this Phase Ib study, the investigational study drug, which is a peptide (small protein), is expected to decrease the activity of the complement cascade and prevent the destruction of defective red blood cells. Results from the ongoing/completed studies also provided good assurance of the safety for the doses of APL-2 in this study.

c. Dosing of APL-2

APL-2 will be given daily, as a subcutaneous (the fat tissue under your skin) injection and it will be given either, by a nurse in the clinic, or by a nurse who visits your home.

There will be 2 cohorts (groups) in the study and each group will include 3 participants with PNH. Groups will be dosed sequentially i.e. group 1 will be dosed before group 2. It may be possible for you to participate in more than one cohort if your study doctor agrees.

TABLE 1: Doses and injection volumes of APL-2

	Dose (Days 1 to 84)
**Group 1**	180 mg APL-2 / day
**Group 2**	270 mg APL-2 / day

The decision to proceed to the next group will be agreed by a safety monitoring committee which will include <<Insert PI Name>> and other doctors involved in the study together with members of the sponsor company, Apellis. The safety monitoring committee will review all the safety data available from participants already dosed in the study and will determine if it is safe to enrol the next group of participants. The first review will take place after the first group of 3 participants have completed Day 28.

You will receive APL-2 for 28 days. Your study doctor and Apellis will review the available safety and other study data and if there is evidence of perceived clinical benefit, you will continue to receive APL-2 for a further 56 days.

The study can be stopped at any time, based on evaluation of the side effects of the study medication.

d. Will you know whether you are receiving active treatment?

This study will be done as an open label study which means all participants will receive APL-2 and you will know exactly which group you are assigned to.

2. WHAT WILL HAPPEN IF YOU TAKE PART IN THIS STUDY

a. What will you be responsible for if you take part in this study?

If you decide to take part in this study you will be asked to sign this ethics committee-approved informed consent form and you must meet specific entry rules.

You must also agree to the following:

- Be willing and able to attend all visits at the study center and be available for the home visits.
- Immunisation is required to participate in the study. Vaccinations may be given during the screening visit if you have not received the required immunisation within 2 years of the screening visit. There immunisations will be free as they are required as part of the trial.
- Be willing to allow frequent blood tests during the study and at subsequent follow up visits.
- You will be requested to keep the 'study-supplied small freezer plugged in to a power source during your study treatment. If there is a power failure, you should contact the community nurse or the clinic site.
- Follow the instructions given by the study doctor and study staff.
- Take an antibiotic throughout the study to prevent infections.
- Have all procedures performed.
- Males must agree to no sperm donation at any time during the study.
- Females who are able to have children must agree to use an effective contraception for the duration of the study. Your study doctor can advise you on effective methods of contraception.
- Make sure you take only medications approved by a study doctor. Tell the study doctor what medications you are currently taking or have taken in the past. Also, tell him / her before starting any new medications and/or if there are changes to your current medications.

b. What types of tests or procedures will be involved with this study?

If you decide to take part in the study, some tests will be done to see if you are eligible. These tests include hepatitis B, C and HIV. If you do not want any of the tests done, you should not take part in this study. If the test results show that you do not meet the study requirements, you will not be able to start the study. If you pass the screening assessments, we will ask you to return to <<Insert Site Name>> to begin the study.

If you have not received the required immunisations within 2 year before the screening visit vaccination will be given at least 15 days before you receive the first dose of APL-2 and a booster vaccination against Neisseria meningitides about 2 months later (Day 57 of the study). In addition a vaccination booster may be required against pneumococcus on Day 1 depending on the vaccination previously received, your study doctor will discuss this with you.

You will receive daily injections which will be done by the study staff when you visit the clinic site or by a trained study nurse at home.

<<Insert Site Name>> will contact your regular doctor (GP) about your study participation. If you do not want your GP told, you will not be able to take part in the study. We may need to contact your

GP about any health issues or parts of your medical history that might affect you taking part in the study.

You will be given a Participant Identification Card if you participate in this study, stating the name of the study and the study doctor's contact information. **This card should be carried with you at all times** so that you can contact the study doctor at any time and so you can show it to any other doctor or dentist that you might visit while in this study.

c. What are the study details? What procedures will you have?

Your total time in this study is expected to be approximately 160 days. If more tests are needed, your study doctor may ask you to schedule additional visits which could extend your total time in the study. Your study doctor or Apellis may choose to withdraw you from this research study at any time which could decrease your total time in the study.

The study will have 3 parts:

- Part 1: You will receive APL-2 for 28 days and will include at least 8 visits to the study center and a community nurse will conduct 22 visits in your home.
- Part 2: If there is evidence of perceived clinical benefit by Day 28 you will receive APL-2 for a further 56 days and will include at least 5 visits to the study center and a community nurse will conduct 51 visits in your home. If no no evidence of perceived clinical benefit by Day 28, treatment will be stopped and you will skip Part 2 and enter Part 3 for safety follow-up visits.
- Part 3: Safety follow-up will include 4 visits to the study center.

The day you have your first dose of study drug (APL-2) is called Day 1. All other days are counted back or forward from Day 1. You will be monitored throughout the study for any changes in health. At the start of the study the <<Insert Site Name>> staff will review your medical history to ensure you are eligible for the study. Throughout the entire duration of the study, the <<Insert Site Name>> staff will review any other medications you may be taking and any potential side effects to ensure your safety.

The following activities will be performed during site visits / home visits:

**Screening**<br>**Screening Visits (2 visits) Day -30 to Day -1**
Before we can do any tests, you will be asked to give written informed consent, below are details of assessments that need to be done:<br>• Questions about your health and what medication you are taking<br>• Review of your demographic (personal) information such as date of birth, ethnicity, and race<br>• Review the study requirements and restrictions<br>• Height and weight<br>• Set of vital signs (pulse, blood pressure and breathing rate) and temperature<br>• Full physical exam<br>• An ECG is a painless test that looks at the electrical activity of your heart by putting small sticky patches on certain areas of your body while you are lying down. These patches are connected to thin wires which are again connected to a machine which will interpret the electrical activity of your heart and then print a report.<br>• Set of routine blood and urine samples, blood samples relating to safety and PNH diagnosis<br>• Blood tests for HIV, Hepatitis B and Hepatitis C Pregnancy test (females) +/- hormone test (postmenopausal females)<br>• Vaccination if required will be done during the screening period after your eligibility has been confirmed. Your doctor will discuss with you what vaccinations you may need and will also take into account vaccinations that you have previously received.
**A final decision about whether you can take part will then be made when all your screening results are available.**

Site Visits (Day 1 onwards)
**APL-2 dosing:**<br>• You will receive the first 3 daily doses of APL-2 (Day 1 to 3) as well as doses on Day 8, 15 and 22 at the hospital clinic. You will need to stay in the clinic for 2.5 hours after receiving the first dose of APL-2 on Day 1 so we can make sure that you are okay.<br><br>**Other assessments done throughout the study:**<br>• You will be asked what medications you are taking and if you have had any recent illnesses or changes in your condition.<br>• Preventative Antibiotics will be taken from Day 1 until 14 days after the last dose of APL-2<br>• Full physical exam<br>• An ECG (recording of the electrical activity of your heart).<br>• Set of vital signs (pulse, blood pressure and breathing rate) and temperature. If the visit includes study drug administration, vital signs will be measured before and after you receive the study drug.<br>• Set of routine blood and urine samples, blood samples relating to safety and PNH diagnosis<br>• You will be assessed for signs/symptoms of blood clots.<br>• Pregnancy test (for females who are able to become pregnant).<br>• FACIT (Fatigue Scale) is a 13 item questionnaire which you will need to complete during clinic visits on Days 1, 15, 29, 43, 71, 99 and 134.<br>• Monitor any side effects: you will be asked to notify of any unfavourable health or medical events, referred to as adverse events, experienced from the last visit.<br>• Vaccination against Neisseria meningitides (booster) on Day 57<br>• Vaccination booster may be required against pneumococcus on Day 1 depending on the vaccination previously received
**Home Visits**
If you participate in this study, a small freezer will be shipped directly to your home by the sponsor of this study. The study drug that will be administered to you at home will be stored in this freezer. The freezer will be locked, and only the community nurse will be able to unlock it. Please note, the community nurse is separate from the study staff at the study doctor's clinic. The community nurse will be responsible for ensuring that the freezer in your home, supplied by the study sponsor, maintains the study drug at the appropriate temperature.<br><br>Home visits will be conducted by a community nurse on study days 4-7, 9-14, 16-21, and 23-28, 30-35, 37-42, 44-56, 58-70, 72-84. The following procedures will be performed at each home visit:<br>• You will be asked what medications you are taking and if you have had any illnesses or changes in your condition.<br>• Your vital signs (temperature, resting pulse and blood pressure, respiratory rate) will be measured before and after you receive the study drug.<br>• You will receive a dose of the study drug.<br>• The injection site where the study drug was given will be assessed and the surrounding area will be checked for redness, swelling and hardness. You will be asked about pain and tenderness.<br>• Between Day 23 and Day 28 safety blood samples will be collected to measure LD (Lactate Dehydrogenase). LD is released from the cells into the blood when cells are damaged or destroyed.<br>• You will be assessed for signs/symptoms of blood clots.<br>• This community nurse will remain in your home with you for one hour after the injection of the study drug.<br>• These visits will likely take approximately 2 - 4 hours.<br>**APL-2 dosing:**

- From Day 4 to Day 84 daily doses of APL-2 will be given by a trained nurse at your home with the exception of Days 8, 15, 22, 29, 36, 43, 57 and 71 (see above).

Safety Follow-up period If you have completed Part 1 of the study and there is no reduction in the breakdown of your red blood cells by Day 28, treatment will be stopped and you will skip Part 2 and enter Part 3 for safety follow-up visits. If a reduction is shown you will receive APL-2 for a further 56 days and then enter Part 3 for safety follow-up visits.

You will be asked to return to the clinic site for follow-up visits on Days 85, 99 and 113.

After you have completed all follow-up procedures you need to return for the Exit Visit on Day 134.

You will not receive any study drug at these visits. These visits will include the following tests and procedures:

- You will be asked what medications you are taking and if you have had any illnesses or changes in your condition.
- Your vital signs (temperature, resting pulse and blood pressure, respiratory rate) will be measured.
- You will be assessed for signs/symptoms of blood clots.
- Electrocardiogram (ECG) – Day 85 and 134.
- Physical examination – Day 85 and 134.
- Collection of a urine sample for routine lab tests.
    - For women who are able to get pregnant, urine will be collected at Day 99 and 134 for a pregnancy test.
- Collection of blood.
- You will be asked to complete a Fatigue Scale questionnaire at Day 99 and 134.

3. WHAT WILL HAPPEN TO MY TEST SAMPLES

The following blood samples will be collected throughout the study:

- Samples to determine regular routine blood counts and blood chemistry, to monitor your general health. (Haematology and Chemistry)
- Blood tests for HIV, Hepatitis B and Hepatitis C (Serology)
- Blood tests to determine how quickly your blood clots (Coagulation Profile)
- Pregnancy Test (If applicable)

All these routine samples will be sent to a Central lab overseas for testing and destroyed at the end of the study by internationally accepted means e.g. incineration.

The following blood samples will also be collected throughout the study. These samples will be sent to different locations for testing.

- Blood samples which determine the level of study drug in your blood (Pharmacokinetics).
- Blood samples to determine the effects of study drug on your body (Pharmacodynamics).
- Blood samples for antigenicity (The ability to cause the production of antibodies to the study drug).
- Blood samples for cytometry testing (This test is able to identify the type/amount of cells in different parts of the cell cycle).

These blood samples may be stored for up to 1 year after the end of treatment after which time they will be destroyed by internationally accepted means e.g. incineration.

The maximum amount of blood collected from each participant during the study will be approximately 341 mls (Approximately 23 tablespoons).

All samples will be coded; identified only by each participant's study number and initials. Only hospital and laboratory staff will have access to the blood samples.

The proposed blood tests include a screening test for HIV (also called the 'AIDS' virus) and viruses causing hepatitis. You will receive information and counselling before the test. If a test shows you have HIV, you will have follow-up counselling and medical advice. Signing the consent form means that you agree to have this testing; it will not be done without your consent.

Additional information if you are from Maori descent:

We understand that many Maori consider their tissue to be tapu and that participation in this type of study requires careful consideration. You may wish to discuss the study with your whanau or obtain the blessing of your iwi for participation in this research. They may have a position on participation in these types of studies and issues such as sending blood samples overseas and access to results of the study. Should you have any concerns regarding appropriate practice/tikanga to address cultural issues arising from your participation in the study it is recommended you consult with a kaumatua.

4. SAFETY: POTENTIAL RISKS AND DISCOMFORTS

a. Are there any risks from this study?

There may be risks to being in this study from APL-2 or from some of the procedures or tests done in this study.

If you take part in this study, you or your family members should tell the study doctor or <<Insert Site Name>> staff immediately if you have any unusual health problems, injuries, or side effects, even if you do not think these problems are caused by the study or APL-2.

If there is any new important safety information or other information that could affect your willingness to participate in this study, your study doctor will let you know.

b. What are the likely risks with APL-2?

You may feel discomfort during some of the tests and study procedures. Possible risks, side effects, and discomforts include:

- Infections – you may be at increased risk for infections caused by certain types of organisms such as Streptococcus pneumonia, Neisseria Meningitidis, or Haemophilus influenza.
- Allergic reactions – all drugs have a potential risk of an allergic reaction, which if not treated promptly, could become life-threatening.
- Blood samples - you may have discomfort or pain when your blood is collected. You may feel faint or pass out. There is also a slight possibility of infection, bruising, or bleeding at the puncture site. Your anaemia (low red blood cell count) could worsen from having frequent blood draws
- ECG - skin irritation is rare but could occur during an ECG from the electrodes or gel that is used
- Antibiotic therapy – there is a possibility that you will experience some side effects from the antibiotic treatment you will need to take during this study. Side effects such as nausea, sickness, diarrhoea and skin rashes may occur and if you experience any of these you should inform the nurse or doctor. The doctor may decide that you should change to a different antibiotic to try and reduce these symptoms.
- Injection site reactions - there is a possibility that you could experience redness, swelling, and pain or tenderness at the site of injection. There is also a slight possibility of infection.

- Vaccination is required: Like any medication, vaccines, can cause side effects. The most common side effects are mild. The side effects associated with getting vaccines are almost always mild (such as redness and swelling where the shot was given) and go away within a few days.

c. Any unforeseen risks?

Since the study drug APL-2 is investigational when taken alone or in combination with other medications, there may be other risks that are unknown.

d. Could APL-2 be harmful to an unborn or breastfed baby?

You should discuss pregnancy risks with your doctor before signing this consent form. Women who are pregnant, planning to become pregnant or breast feeding may not participate in this research study. Taking the study drug may involve unknown risks to a pregnant woman, an embryo, fetus (unborn baby) or nursing infant. If you (or your partner) become pregnant while in this study, the sponsor may ask to follow the outcome of the pregnancy. The study drug will be stopped and your participation in this study will be ended.

Before starting this research study and during the study, females able to have children will have pregnancy tests done. Talk to your doctor about the best method of birth control to use while you are in this study. It is important that you call your study doctor at <<Insert Number>> right away if you become pregnant or father a child during the course of this study.

5. BENEFITS OF PARTICIPATION

Are there any benefits to taking part in this study?

You may or may not benefit from participation in this study. There is no guarantee that you will benefit from participating in this study. The information from this study might help to develop better treatments in the future for people with PNH. An indirect benefit of taking part in this study is that you will receive free medical tests at screening and during the study.

What are the alternatives to taking part? Do you have any other choices?

You do not have to be in this study to receive treatment for your PNH. You have the option to receive standard treatment for PNH. Standard treatment may include medications such as prednisone, or blood thinners; blood transfusions; or bone marrow transplantation. The study doctor will discuss with you the risks and benefits of the alternative treatments.

6. POTENTIAL COSTS/ REIMBURSEMENTS

There will be no charge to you for your participation in this study. The study drug, study-related procedures, and study visits will be provided at no charge to you.

Will you be paid for your samples (such as blood or urine) or medical information?

You will not be paid for the use of your samples or any information, discoveries or inventions obtained from your samples or medical information.

You will be reimbursed for any reasonable travel, accommodation, parking, meals and other expenses associated with the research project visit, this will based on the distance that you have to travel to the hospital.

7. WHAT ARE THE RIGHTS OF PARTICIPANTS IN THIS STUDY?

a. Participation is voluntary

Participation in any research project is voluntary. If you do not wish to take part, you do not have to. If you decide to take part and later change your mind, you are free to withdraw from the project at

any stage. This will not affect your routine treatment, your relationship with those treating you or with <<Insert Site Name>>.
If you do decide to take part, you will be given this Participant Information and Consent Form to sign and you will be given a copy to keep.

b. New information
Sometimes during the course of a research project, new information becomes available about the treatment that is being studied. If this happens, your study doctor will tell you about it and discuss with you whether you want to continue in the research project. If you decide to withdraw, your study doctor will make arrangements for your regular health care to continue. If you decide to continue in the research project you may be asked to sign an updated consent form.

Also, on receiving new information, your study doctor might consider it to be in your best interests to withdraw you from the research project. If this happens, he/ she will explain the reasons and arrange for your regular health care to continue.

c. Right to access information collected during the study

In accordance with relevant New Zealand privacy and other relevant laws, you have the right to request access to your information collected and stored by the research team. You also have the right to request that any information with which you disagree be corrected. Please contact the study team member named at the end of this document if you would like to access your information.

8. CONFIDENTIALITY

By signing the consent form you consent to the study doctor and relevant research staff collecting and using personal information about you for the research project. Any information obtained in connection with this research project that can identify you will remain confidential. Your name, address or phone number will not be on the study forms; instead you will be identified by a unique study number, date of birth and initials. Your information will only be used for the purpose of this research project and it will only be disclosed with your permission, except as required by law.

Information about you may be obtained from your health records held at this and other health services for the purpose of this research. By signing the consent form you agree to the study team accessing health records if they are relevant to your participation in this research project.

Your health records and any information obtained during the research project are subject to inspection (for the purpose of verifying the procedures and the data) by the relevant authorities (such as the Ethics Committee that has reviewed and approved this study) and authorised representatives of the Sponsor, Apellis, and <<Insert Site Name>>, or as required by law. All personnel reviewing your medical records will be required to keep the information confidential in accordance with law. By signing the Consent Form, you authorise release of, or access to, this confidential information to the relevant study personnel and regulatory authorities as noted above.

When the research project ends the study data must be analysed, so the results of the study may not be available until about six months after the research finishes. The study doctors and/or Apellis may decide to discuss or publish the results of the study. This may include publication in journals, presentation at conferences or other professional forums. In any publication, information will be provided in such a way that you cannot be identified. Results of the study will be provided to you, if you wish.

Information about your participation in this research project may be recorded in your health records.

Any information obtained for the purpose of this research project that can identify you will be treated as confidential and securely stored. It will be disclosed only with your permission, or as required by law.

Upon completion of the trial, the study file containing information about you will be archived for a minimum of fifteen years.

9. COMPENSATION FOR INJURY

If you were injured as a result of treatment given as part of this study, which is unlikely, you won't be eligible for compensation from ACC. However, compensation would be available from the study's sponsor, Apellis, in line with industry guidelines. We can give you a copy of these guidelines if you wish.

If you have private health or life insurance, you may wish to check with your insurer that taking part in this study won't affect your cover.

10. WHAT WILL HAPPEN AFTER THE STUDY ENDS, OR IF YOU PULL OUT?

If you decide to withdraw
Please notify a member of the research team before you withdraw. This notice will allow that person or the research supervisor to discuss any health risks or special requirements linked to withdrawing, such as follow up visits.

If you do withdraw your consent during the research project, information already collected will be retained to ensure that the results of the research project can be measured properly and to comply with law. You should be aware that data collected by the sponsor up to the time you withdraw will form part of the research project results. If you do not want them to do this, you must tell them before you join the research project.

Why the study might be unexpectedly stopped
This research project may be stopped unexpectedly for a variety of reasons. These may include reasons such as:

- Unacceptable side effects
- The drug being shown not to be effective
- The drug being shown to work and not need further testing.

Will the study treatment continue to be available after the study finished?

If it is found the drug does have a beneficial effect in reducing haemolysis (breakdown of red blood cells), and no safety concerns are raised, the company may, at its sole discretion, after consultation with the Health and Disability Ethics Committees (HDECs) and the Health Research Council (HRC) of New Zealand's Standing Committee on Therapeutic Trials (SCOTT), elect to continue the drug therapy, either by including patients, like yourself, in a further treatment cohort, or by commencing an extension study.

Where can you go for more information about the study, or to raise concerns or complaints?

If you have any questions, concerns or complaints about the study at any stage, you can contact:

<Insert PI Name>>, lead study doctor
Phone: <<Insert Number>>
Research Coordinator: <<Insert Number>>

If you want to talk to someone who isn't involved with the study, you can contact an independent health and disability advocate. Specialist advocates, are available here.

Phone: 0800 555 050
Fax: 0800 2 SUPPORT (0800 2787 7678)
Email: advocacy@hdc.org.nz

You can also contact the health and disability ethics committee (HDEC) that approved this study on:

Phone: 0800 4 ETHICS (438 442)

Email: hdecs@moh.govt.nz

For Maori health support, or to discuss any concerns or issues regarding this study, please contact Maori Health team, Phone: [insert number].

12. DO I HAVE TO DECIDE STRAIGHT AWAY?

No, you do not have to decide straight away. You should take some time to consider whether or not to participate**.** Study staff will let you know when they will need your decision. The following steps are useful in helping you reach a decision.

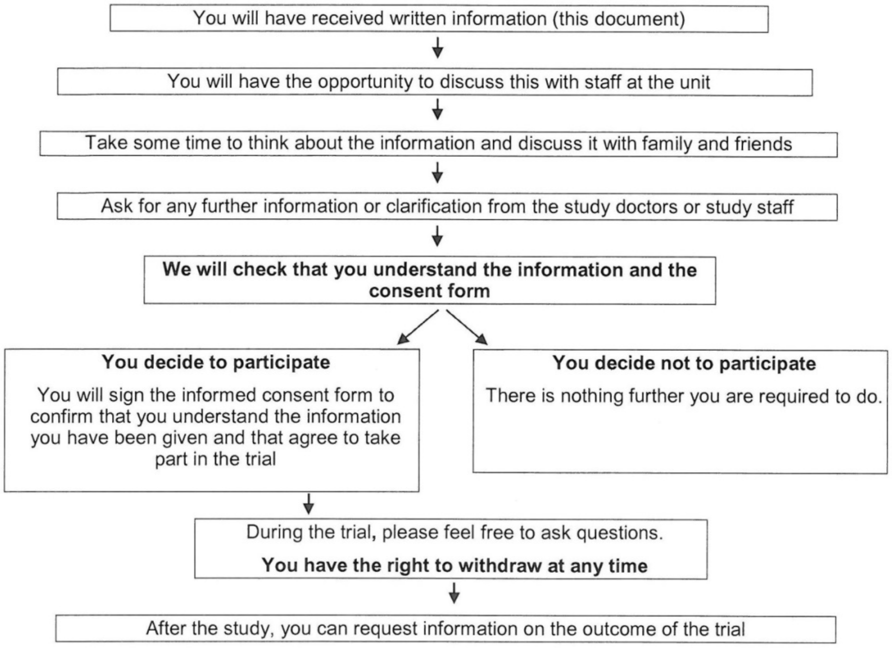

Insert Header with institution's name or institution's letterhead

INFORMED CONSENT FORM

**Short Title**	Nocturnal Hemoglobinuria (PNH) – PADDOCK
**Protocol Number**	APL2-CP-PNH-204
**Principal Investigator**	<<Insert PI Name>>

Declaration by participant:

I have read and I understand this version of the Participant Information Sheet. I have had the opportunity to ask questions and I am satisfied with the answers I have received.

I have had the opportunity to discuss the study with my family/whanau.

I freely agree to participate in this study and understand that I may withdraw from the study at any time.

I understand that:

- My participation in this study is confidential and I will not be identified in study reports.
- My medical records may be reviewed by Apellis, companies working with them, auditors and health or regulatory agencies for the purpose of checking the accuracy of the information recorded for the study
- Some of my samples will be sent overseas for analysis and they will be destroyed as described in the Information Sheet.
- If I decide to withdraw from the study, information already collected may continue to be processed. I may be asked to attend follow-up visits.
- The study drugs or tests will be stopped if they appear harmful to me.
- I should contact site staff if I have any side effects.
- My GP and/or specialist will be informed of my participation in this study and relevant medical records may be accessed.
- I am not giving up any of my legal rights by signing this consent form.
- This trial is being carried out for the principal benefit of Apellis. If I suffer an injury as a result of my participation in the trial, they will pay me compensation in line with industry guidelines.
- I understand that there may be risks associated with the treatment in the event of myself or my partner becoming pregnant. I undertake to inform my partner of the risks and to take responsibility for the prevention of pregnancy

Statement by Participant I hereby consent to take part in this study. I understand that I will receive a signed copy of this consent form for my records.

________________________________ (full name)

________________________________ (signature)

__ __ / __ __ __ / __ __ (Date)

Statement by Consenter (Investigator/designee) I have discussed this study with the above named participant. The participant appeared to fully understand the information provided about the study.

________________________________ (full name)

________________________________ (signature)

__ __ / __ __ __ / __ __ (Date) __ __ :__ __ (Time)

Waikato District Health Board
Te Hanga Whaioranga Mō Te Iwi – Building Healthy Communities

PARTICIPANT INFORMATION SHEET PART 2B
WAIKATO DHB

**Short Title**	A Phase Ib, Open Label, Multiple Ascending Dose, Pilot Study to Assess the Safety, Preliminary Efficacy and Pharmacokinetics of Subcutaneously Administered APL-2 in Subjects with Paroxysmal Nocturnal Hemoglobinuria (PNH). – PADDOCK		
**Protocol Number**	APL2-CP-PNH-204	**Ethics Number**	15/NTA/152
**Project Sponsor**	Apellis Pharmaceuticals, Inc.<br>6400 Westwind Way, Suite A, Louisville, KY, 40014		
**Local Sponsor**	Clinical Network Services (CNS) Ltd<br>PO Box 78312, Grey Lynn, Auckland, 1245		
**Principal Investigator**	Dr Humphrey Pullon		
**Location**	Waikato Hospital		
**Study related phone number**	07 839 8976		

You are being invited to continue to take part in a research study of the trial drug APL-2. This consent form gives you important information about this study to help you decide if you want to continue in the research study. It describes the purpose of this study, the study procedures, the possible risks, and provides information about your rights as a study participant.

Please take the time to read this information carefully. You should talk to your study doctor and study staff about this study and ask any questions you have. You can also discuss this study with other people such as your family/whanau or general practitioner (GP). If you decide to continue to take part in this study, you will be asked to sign and date this form. You will receive a copy of the signed form. Your study doctor will tell your GP about your continued participation in this study.

Who is funding this study?

Apellis Pharmaceuticals, Inc. ("Apellis") is funding this clinical study. Apellis is the "sponsor" of the study, which means that Apellis takes responsibility for starting, managing, and financing the study. Waikato Hospital is being paid by Apellis to do this study.

Voluntary participation and withdrawal from this study

Your participation in this study is voluntary. You are free to say yes or no. Even if you join this study, you may stop at any time.

A description of this clinical trial will be available on the internet at http://www.ClinicalTrials.gov. This website will not include information that can identify you. At most, the website will include a summary of the results. You can search this website at any time.

1. WHAT YOU SHOULD KNOW ABOUT THIS STUDY

a. Why is this study being done?

This is a research study to test a new investigational drug. An investigational drug is one that is not approved by any regulatory authority (e.g. FDA, Medsafe etc) for use outside a clinical trial.

You are being invited to continue to participate in this research study because your study doctor believes that you have shown clinical benefit since you started to receive APL-2 to treat your disorder of paroxysmal nocturnal hemoglobinuria (PNH). PNH is a rare disorder that causes the red blood cells to break down too early. Many PNH patients suffer from complications such as blood clots or low red blood cell counts (anemia) even after receiving standard treatment.

The research study will continue to gather scientific information about the safety, tolerability and continued evidence of activity of the investigational drug APL-2 in people with PNH who have not been treated with Soliris® {an approved medication (not available in NZ) that blocks the breakdown of red blood cells} in the past. The study will help determine how APL-2 behaves inside the body, how it is eliminated from the body, the effect it has on the body after multiple doses and if it can prevent red blood cells from breaking down.

b. How does APL-2 works?

Your body has numerous biological mechanisms designed to attack and kill off invaders, such as bacteria. One of these mechanisms, called the complement cascade, is part of your normal immune system. Normally, only invaders are destroyed by the complement cascade as your own cells are protected against its attacks. However, patients with PNH possess red blood cells that are defective and are not properly protected, which results in the destruction of these defective red blood cells by your own immune system. The breakdown of a large number of red blood cells in this manner has been shown to trigger episodes of PNH. Based on the research conducted prior to this Phase Ib study, the investigational study drug, which is a peptide (small protein), is expected to decrease the activity of the complement cascade and prevent the destruction of defective red blood cells. Results from the ongoing/completed studies also provided good assurance of the safety for the doses of APL-2 in this study.

c. Dosing of APL-2

APL-2 will continue to be given daily, as a subcutaneous (the fat tissue under your skin) injection and it will be given either, by a nurse in the clinic, or by a nurse who visits your home, workplace, or other location agreed by you.

You have already received APL-2 for 84 days. You are being invited to continue to receive APL-2 for up to 364 days

The safety monitoring committee will continue to review all the safety data available from participants already dosed in the study approximately every 8 weeks.

The study can be stopped at any time, based on evaluation of the side effects of the study medication.

2. WHAT WILL HAPPEN IF YOU CONTINUE TO TAKE PART IN THIS STUDY

a. What will you be responsible for if you continue to take part in this study?

If you decide to continue to take part in this study you will be asked to sign this ethics committee-approved informed consent form and you must meet specific entry rules.

You must also agree to the following:

- Be willing and able to attend all visits at the study center and be available for the off-site nurse visits.

- Immunisation is required to participate in the study. Vaccinations may be given during the screening visit if you have not received the required immunisation within 2 years of the screening visit. There immunisations will be free as they are required as part of the trial. N.B. no further immunisation is required for your continued participation in the study (up to 364 days of treatment)
- Be willing to allow frequent blood tests during the study and at subsequent follow up visits.
- You will be requested to keep the 'study-supplied small freezer plugged in to a power source during your study treatment. If there is a power failure, you should contact the community nurse or the clinic site.
- Follow the instructions given by the study doctor and study staff.
- Take an antibiotic throughout the study to prevent infections.
- Have all procedures performed.
- Males must agree to no sperm donation at any time during the study.
- Females who are able to have children must agree to use an effective contraception for the duration of the study. Your study doctor can advise you on effective methods of contraception.
- Make sure you take only medications approved by a study doctor. Tell the study doctor what medications you are currently taking or have taken in the past. Also, tell him / her before starting any new medications and/or if there are changes to your current medications.

b. What types of tests or procedures will be involved with continuation in this study?

You will receive daily injections which will be done by the study staff when you visit the clinic site or by a trained study nurse at home, workplace, or other location convenient to you.

Dr Humphrey Pullon will contact your regular doctor (GP) about your continued study participation. If you do not want your GP told, you will not be able to continue to take part in the study. We may need to contact your GP about any health issues or parts of your medical history that might affect your continuation in the study.

You were given a Participant Identification Card when you started in this study, stating the name of the study and the study doctor's contact information. **This card should continue to be carried with you at all times** so that you can contact the study doctor at any time and so you can show it to any other doctor or dentist that you might visit while in this study.

c. What are the study details? What procedures will you have?

Your total time in this study is expected to be approximately 414 days. If more tests are needed, your study doctor may ask you to schedule additional visits which could extend your total time in the study. Your study doctor or Apellis may choose to withdraw you from this research study at any time which could decrease your total time in the study.

The study has 4 parts (you have already completed parts 1 and 2A):

- Part 1: You received APL-2 for 28 days and you visited the study centre at least 8 times and a community nurse conducted 22 visits in your home.
- Part 2A: There was evidence of clinical benefit to you by Day 28; therefore, you continued to receive APL-2 for a further 56 days. You visited the study centre 5 more times and a community nurse conducted 51 visits in your home.
- Part 2B: You may continue to receive daily APL-2 treatment for up to 364 days if there is ongoing evidence of clinical benefit to you following review of the available study data. Part 2B will include at least 10 visits to the study center and a community nurse will conduct all other visits in your home, workplace, or other location convenient to you. If there is evidence that you are no longer getting clinical benefit, treatment will be stopped and you will skip Part 2B and enter Part 3 for safety follow-up visits
- Part 3: Safety follow-up will include 4 visits to the study center.

You will continue to be monitored throughout the study for any changes in health. Throughout the entire duration of the study, the *Waikato Hospital* staff will continue to review any other medications you may be taking and any potential side effects to ensure your safety.

The following activities will be performed during site and off-site visits:

Site Visits (Day 1 onwards)
**APL-2 dosing:**<br>• You will receive doses of APL-2 on Day 85, 113, 141, 169, 197, 225, 253, 281, 309 and 337 at the hospital clinic.<br><br>**Other assessments done throughout the study:**<br>• You will be asked what medications you are taking and if you have had any recent illnesses or changes in your condition.<br>• Preventative Antibiotics will be taken from Day 1 until 14 days after the last dose of APL-2<br>• Full physical exam<br>• An ECG (recording of the electrical activity of your heart).<br>• Set of vital signs (pulse, blood pressure and breathing rate) and temperature. If the visit includes study drug administration, vital signs will be measured before and after you receive the study drug.<br>• Set of routine blood and urine samples, blood samples relating to safety and PNH diagnosis<br>• You will be assessed for signs/symptoms of blood clots.<br>• Pregnancy test (for females who are able to become pregnant).<br>• FACIT (Fatigue Scale) is a 13 item questionnaire which you will need to complete during clinic visits on Days 85,141,197, 253, 309, 365 and 414.<br>• Monitor any side effects: you will be asked to notify of any unfavourable health or medical events, referred to as adverse events, experienced from the last visit.
**Home Visits** (or workplace, or other location convenient to the subject)
If you continue to participate in this study, a small freezer will be shipped directly to your home and or other nominated location by the sponsor of this study. The study drug that will be administered to you at home will be stored in this freezer. The freezer will be locked, and only the community nurse will be able to unlock it. Please note, the community nurse is separate from the study staff at the study doctor's clinic. The community nurse will be responsible for ensuring that the freezer in your home, supplied by the study sponsor, maintains the study drug at the appropriate temperature.<br><br>Home visits will be conducted by a community nurse on study days 86-112, 114-140, 142-168, 170-196, 198-224, 226-252, 254-280, 282-308, 310-336 and 338-364. The following procedures will be performed at each home visit:<br>• You will be asked what medications you are taking and if you have had any illnesses or changes in your condition.<br>• Your vital signs (temperature, resting pulse and blood pressure, respiratory rate) will be measured before and after you receive the study drug.<br>• You will receive a dose of the study drug.<br>• The injection site where the study drug was given will be assessed and the surrounding area will be checked for redness, swelling and hardness. You will be asked about pain and tenderness.<br>• You will be assessed for signs/symptoms of blood clots.<br>• This community nurse will remain in your home with you for one hour after the injection of the

study drug.

- These visits will likely take approximately 2 - 4 hours.

APL-2 dosing:

- From Day 85 to Day 364 daily doses of APL-2 will be given by a trained nurse at your home or other location agreed by you, with the exception of Days 85, 113, 141, 169, 197, 225, 253, 281, 309 and 337 (see above).

Safety Follow-up

You will be asked to return to the clinic site for follow-up visits on Days 365, 379, and 393.

After you have completed all follow-up procedures you need to return for the Exit Visit on Day 414.

You will not receive any study drug at these visits. These visits will include the following tests and procedures:

- You will be asked what medications you are taking and if you have had any illnesses or changes in your condition.
- Your vital signs (temperature, resting pulse and blood pressure, respiratory rate) will be measured.
- You will be assessed for signs/symptoms of blood clots.
- Electrocardiogram (ECG) – Day 365.
- Physical examination – Day 365 and 414.
- Collection of a urine sample for routine lab tests.
    - For women who are able to get pregnant, urine will be collected at Day 365, 393 and 414 for a pregnancy test.
- Collection of blood.
- You will be asked to complete a Fatigue Scale questionnaire at Day 365, 393 and 414.

3. WHAT WILL HAPPEN TO MY TEST SAMPLES

The following blood samples will be collected throughout the study:

- Samples to determine regular routine blood counts and blood chemistry, to monitor your general health. (Haematology and Chemistry)
- Blood tests for HIV, Hepatitis B and Hepatitis C (Serology)
- Blood tests to determine how quickly your blood clots (Coagulation Profile)
- Pregnancy Test (If applicable)

All these routine samples will be sent to a Central lab overseas for testing and destroyed at the end of the study by internationally accepted means e.g. incineration.

The following blood samples will also be collected throughout the study. These samples will be sent to different locations for testing.

- Blood samples which determine the level of study drug in your blood (Pharmacokinetics).
- Blood samples to determine the effects of study drug on your body (Pharmacodynamics).
- Blood samples for antigenicity (The ability to cause the production of antibodies to the study drug).
- Blood samples for cytometry testing (This test is able to identify the type/amount of cells in different parts of the cell cycle).

These blood samples may be stored for up to 1 year after the end of treatment after which time they will be destroyed by internationally accepted means e.g. incineration.

The maximum amount of blood collected from each participant during the study will be approximately 580 mls (Approximately 39 tablespoons).

All samples will be coded; identified only by each participant's study number and initials. Only hospital and laboratory staff will have access to the blood samples.

Additional information if you are from Maori descent:

We understand that many Maori consider their tissue to be tapu and that participation in this type of study requires careful consideration. You may wish to discuss the study with your whanau or obtain the blessing of your iwi for participation in this research. They may have a position on participation in these types of studies and issues such as sending blood samples overseas and access to results of the study. Should you have any concerns regarding appropriate practice/tikanga to address cultural issues arising from your participation in the study it is recommended you consult with a kaumatua.

4. SAFETY: POTENTIAL RISKS AND DISCOMFORTS

a. Are there any risks from your contued participation in this study?

There may be risks to continuing in this study from APL-2 or from some of the procedures or tests done in this study.

If you continue to take part in this study, you or your family members should tell the study doctor or *Waikato Hospital* staff immediately if you have any unusual health problems, injuries, or side effects, even if you do not think these problems are caused by the study or APL-2.

If there is any new important safety information or other information that could affect your willingness to participate in this study, your study doctor will let you know.

b. What are the likely risks with APL-2?

You may feel discomfort during some of the tests and study procedures. Possible risks, side effects, and discomforts include:

- Infections – you may be at increased risk for infections caused by certain types of organisms such as Streptococcus pneumonia, Neisseria Meningitidis, or Haemophilus influenza.
- Allergic reactions – all drugs have a potential risk of an allergic reaction, which if not treated promptly, could become life-threatening.
- Blood samples - you may have discomfort or pain when your blood is collected. You may feel faint or pass out. There is also a slight possibility of infection, bruising, or bleeding at the puncture site. Your anaemia (low red blood cell count) could worsen from having frequent blood draws
- ECG - skin irritation is rare but could occur during an ECG from the electrodes or gel that is used
- Antibiotic therapy – there is a possibility that you will experience some side effects from the antibiotic treatment you will need to take during this study. Side effects such as nausea, sickness, diarrhoea and skin rashes may occur and if you experience any of these you should inform the nurse or doctor. The doctor may decide that you should change to a different antibiotic to try and reduce these symptoms.
- Injection site reactions - there is a possibility that you could experience redness, swelling, and pain or tenderness at the site of injection. There is also a slight possibility of infection.

c. Any unforeseen risks?

Since the study drug APL-2 is investigational when taken alone or in combination with other medications, there may be other risks that are unknown.

d. Could APL-2 be harmful to an unborn or breastfed baby?

You should discuss pregnancy risks with your doctor before signing this consent form. Women who are pregnant, planning to become pregnant or breast feeding may not participate in this research study. Taking the study drug may involve unknown risks to a pregnant woman, an embryo, fetus (unborn baby) or nursing infant. If you (or your partner) become pregnant while in this study, the sponsor may ask to follow the outcome of the pregnancy. The study drug will be stopped and your participation in this study will be ended.

Before continuing in this research study and during the study, females able to have children will have pregnancy tests done. Talk to your doctor about the best method of birth control to use while you are in this study. It is important that you call your study doctor at ***07 839 8899 via switch*** right away if you become pregnant or father a child during the course of this study.

5. BENEFITS OF PARTICIPATION

Are there any benefits to your continued participation in this study?

Your study doctor believes that you have shown clinical benefit from participation in the research study to date. You may or may not benefit from continued participation in this study. There is no guarantee that you will continue to benefit from participating in this study. The information from this study might help to develop better treatments in the future for people with PNH. An indirect benefit of taking part in this study is that you will receive free medical tests at screening and during the study.

What are the alternatives to taking part? Do you have any other choices?

You do not have to continue in this study to receive treatment for your PNH. You have the option to receive standard treatment for PNH. Standard treatment may include medications such as prednisone, or blood thinners; blood transfusions; or bone marrow transplantation. The study doctor will discuss with you the risks and benefits of the alternative treatments.

6. POTENTIAL COSTS/ REIMBURSEMENTS

There will be no charge to you for your participation in this study. The study drug, study-related procedures, and study visits will be provided at no charge to you.

Will you be paid for your samples (such as blood or urine) or medical information?

You will not be paid for the use of your samples or any information, discoveries or inventions obtained from your samples or medical information.

You will be reimbursed for any reasonable travel, accommodation, parking, meals and other expenses associated with the research project visit, this will based on the distance that you have to travel to the hospital.

7. WHAT ARE THE RIGHTS OF PARTICIPANTS IN THIS STUDY?

a. Participation is voluntary

Participation in any research project is voluntary. If you do not wish to take part, you do not have to. If you decide to take part and later change your mind, you are free to withdraw from the project at any stage. This will not affect your routine treatment, your relationship with those treating you or with *Waikato Hospital.*

If you do decide to take part, you will be given this Participant Information and Consent Form to sign and you will be given a copy to keep.

b. New information
Sometimes during the course of a research project, new information becomes available about the treatment that is being studied. If this happens, your study doctor will tell you about it and discuss with you whether you want to continue in the research project. If you decide to withdraw, your study doctor will make arrangements for your regular health care to continue. If you decide to continue in the research project you may be asked to sign an updated consent form.

Also, on receiving new information, your study doctor might consider it to be in your best interests to withdraw you from the research project. If this happens, he/ she will explain the reasons and arrange for your regular health care to continue.

c. Right to access information collected during the study

In accordance with relevant New Zealand privacy and other relevant laws, you have the right to request access to your information collected and stored by the research team. You also have the right to request that any information with which you disagree be corrected. Please contact the study team member named at the end of this document if you would like to access your information.

8. CONFIDENTIALITY

By signing the consent form you consent to the study doctor and relevant research staff collecting and using personal information about you for the research project. Any information obtained in connection with this research project that can identify you will remain confidential. Your name, address or phone number will not be on the study forms; instead you will be identified by a unique study number, date of birth and initials. Your information will only be used for the purpose of this research project and it will only be disclosed with your permission, except as required by law.

Information about you may be obtained from your health records held at this and other health services for the purpose of this research. By signing the consent form you agree to the study team accessing health records if they are relevant to your participation in this research project.

Your health records and any information obtained during the research project are subject to inspection (for the purpose of verifying the procedures and the data) by the relevant authorities (such as the Ethics Committee that has reviewed and approved this study) and authorised representatives of the Sponsor, Apellis, and *Waikato Hospital* or as required by law. All personnel reviewing your medical records will be required to keep the information confidential in accordance with law. By signing the Consent Form, you authorise release of, or access to, this confidential information to the relevant study personnel and regulatory authorities as noted above.

When the research project ends the study data must be analysed, so the results of the study may not be available until about six months after the research finishes. The study doctors and/or Apellis may decide to discuss or publish the results of the study. This may include publication in journals, presentation at conferences or other professional forums. In any publication, information will be provided in such a way that you cannot be identified. Results of the study will be provided to you, if you wish.

Information about your participation in this research project may be recorded in your health records.

Any information obtained for the purpose of this research project that can identify you will be treated as confidential and securely stored. It will be disclosed only with your permission, or as required by law.

Upon completion of the trial, the study file containing information about you will be archived for a minimum of fifteen years.

9. COMPENSATION FOR INJURY

If you were injured as a result of treatment given as part of this study, which is unlikely, you won't be eligible for compensation from ACC. However, compensation would be available from the study's sponsor, Apellis, in line with industry guidelines. We can give you a copy of these guidelines if you wish.

If you have private health or life insurance, you may wish to check with your insurer that taking part in this study won't affect your cover.

10. WHAT WILL HAPPEN AFTER THE STUDY ENDS, OR IF YOU PULL OUT?

If you decide to withdraw
Please notify a member of the research team before you withdraw. This notice will allow that person or the research supervisor to discuss any health risks or special requirements linked to withdrawing, such as follow up visits.

If you do withdraw your consent during the research project, information already collected will be retained to ensure that the results of the research project can be measured properly and to comply with law. You should be aware that data collected by the sponsor up to the time you withdraw will form part of the research project results. If you do not want them to do this, you must tell them before you join the research project.

Why the study might be unexpectedly stopped
This research project may be stopped unexpectedly for a variety of reasons. These may include reasons such as:
- Unacceptable side effects
- The drug being shown not to be effective
- The drug being shown to work and not need further testing.

Will the study treatment continue to be available after the study finished?

If it is found the drug does continue to have a beneficial effect in reducing haemolysis (breakdown of red blood cells), and no safety concerns are raised, the company may, at its sole discretion, after consultation with the Health and Disability Ethics Committees (HDECs) of New Zealand's Standing Committee on Therapeutic Trials (SCOTT), elect to continue the drug therapy by offering a further extension to the study.

Where can you go for more information about the study, or to raise concerns or complaints?

If you have any questions, concerns or complaints about the study at any stage, you can contact:

Dr Humphrey Pullon, lead study doctor
Phone: 07 839 8899 and ask operator to page or put you through to Dr. Pullon
Research Coordinator: 07 839 8976

If you want to talk to someone who isn't involved with the study, you can contact an independent health and disability advocate. Specialist advocates, are available here.

Phone:	0800 555 050
Fax:	0800 2 SUPPORT (0800 2787 7678)
Email:	advocacy@hdc.org.nz

You can also contact the health and disability ethics committee (HDEC) that approved this study on:

Phone:	0800 4 ETHICS (438 442)
Email:	hdecs@moh.govt.nz

For Maori health support, or to discuss any concerns or issues regarding this study, please contact Maori Health team, Phone: 07 839 7628.

12. DO I HAVE TO DECIDE STRAIGHT AWAY?

No, you do not have to decide straight away. You should take some time to consider whether or not to continue your participation in the study. Study staff will let you know when they will need your decision. The following steps are useful in helping you reach a decision.

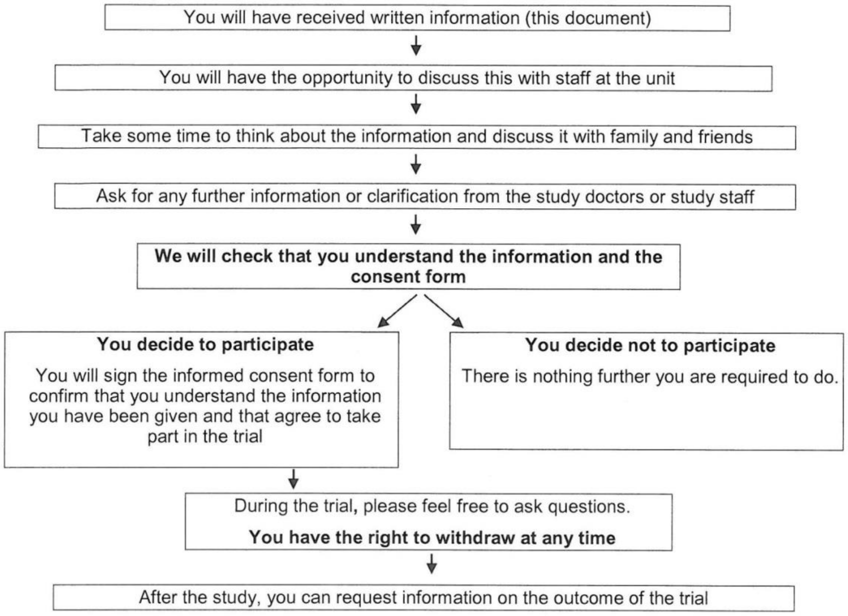

Waikato District Health Board
Te Hanga Whaioranga Mō Te Iwi – Building Healthy Communities

INFORMED CONSENT FORM

**Short Title**	Nocturnal Hemoglobinuria (PNH) – PADDOCK
**Protocol Number**	APL2-CP-PNH-204
**Principal Investigator**	Dr Humphrey Pullon

Declaration by participant:
I have read and I understand this version of the Participant Information Sheet. I have had the opportunity to ask questions and I am satisfied with the answers I have received.

I have had the opportunity to discuss the study with my family/whanau.

I freely agree to continue to participate in this study and understand that I may withdraw from the study at any time.

I understand that:

- My continued participation in this study is confidential and I will not be identified in study reports.
- My medical records may be reviewed by Apellis, companies working with them, auditors and health or regulatory agencies for the purpose of checking the accuracy of the information recorded for the study
- Some of my samples will be sent overseas for analysis and they will be destroyed as described in the Information Sheet.
- If I decide to withdraw from the study, information already collected may continue to be processed. I may be asked to attend follow-up visits.
- The study drugs or tests will be stopped if they appear harmful to me.
- I should contact site staff if I have any side effects.
- My GP and/or specialist will be informed of my participation in this study and relevant medical records may be accessed.
- I am not giving up any of my legal rights by signing this consent form.
- This trial is being carried out for the principal benefit of Apellis. If I suffer an injury as a result of my participation in the trial, they will pay me compensation in line with industry guidelines.
- I understand that there may be risks associated with the treatment in the event of myself or my partner becoming pregnant. I undertake to inform my partner of the risks and to take responsibility for the prevention of pregnancy

**Statement by Participant**	I hereby consent to take part in this study. I understand that I will receive a signed copy of this consent form for my records.<br><br>___________________________________ (full name)<br><br>___________________________________ (signature)<br><br>__ __ / __ __ __ / __ __ (Date)

Statement by Consenter (Investigator/designee) I have discussed this study with the above named participant. The participant appeared to fully understand the information provided about the study.

___________________________________ (full name)

___________________________________ (signature)

__ __ / __ __ __ / __ __ (Date)

PARTICIPANT INFORMATION SHEET PART2C

[insert site]

**Short Title**	A Phase Ib, Open Label, Multiple Ascending Dose, Pilot Study to Assess the Safety, Preliminary Efficacy and Pharmacokinetics of Subcutaneously Administered APL-2 in Subjects with Paroxysmal Nocturnal Hemoglobinuria (PNH). – PADDOCK		
**Protocol Number**	APL2-CP-PNH-204	**Ethics Number**	15/NTA/152
**Project Sponsor**	Apellis Pharmaceuticals, Inc.<br>6400 Westwind Way, Suite A, Louisville, KY, 40014		
**Local Sponsor**	Clinical Network Services (CNS) Ltd<br>PO Box 78312, Grey Lynn, Auckland, 1245		
**Principal Investigator**	*[insert PI]*		
**Location**	*[insert site]*		
**Study related phone number**	*[insert contact details]*		

You are being invited to continue to take part in a research study of the trial drug APL-2. This consent form gives you important information about this study to help you decide if you want to continue in the research study. It describes the purpose of this study, the study procedures, the possible risks, and provides information about your rights as a study participant.

Please take the time to read this information carefully. You should talk to your study doctor and study staff about this study and ask any questions you have. You can also discuss this study with other people such as your family/whanau or general practitioner (GP). If you decide to continue to take part in this study, you will be asked to sign and date this form. You will receive a copy of the signed form. Your study doctor will tell your GP about your continued participation in this study.

Who is funding this study?

Apellis Pharmaceuticals, Inc. ("Apellis") is funding this clinical study. Apellis is the "sponsor" of the study, which means that Apellis takes responsibility for starting, managing, and financing the study. [insert site] is being paid by Apellis to do this study

.

Voluntary participation and withdrawal from this study

Your participation in this study is voluntary. You are free to say yes or no. Even if you join this study, you may stop at any time.

A description of this clinical trial will be available on the internet at http://www.ClinicalTrials.gov. This website will not include information that can identify you. At most, the website will include a summary of the results. You can search this website at any time.

1. WHAT YOU SHOULD KNOW ABOUT THIS STUDY

a. Why is this study being done?

This is a research study to test a new investigational drug. An investigational drug is one that is not approved by any regulatory authority (e.g. FDA, Medsafe etc) for use outside a clinical trial.

You are being invited to continue to participate in this research study because your study doctor believes that you have shown clinical benefit since you started to receive APL-2 to treat your disorder of paroxysmal nocturnal hemoglobinuria (PNH). PNH is a rare disorder that causes the red blood cells to break down too early**.** Many PNH patients suffer from complications such as blood clots or low red blood cell counts (anemia) even after receiving standard treatment.

The research study will continue to gather scientific information about the safety, tolerability and continued evidence of activity of the investigational drug APL-2 in people with PNH who have not been treated with Soliris® {an approved medication (not available in NZ) that blocks the breakdown of red blood cells} in the past. The study will help determine how APL-2 behaves inside the body, how it is eliminated from the body, the effect it has on the body after multiple doses and if it can prevent red blood cells from breaking down.

b. How does APL-2 works?

Your body has numerous biological mechanisms designed to attack and kill off invaders, such as bacteria. One of these mechanisms, called the complement cascade, is part of your normal immune system. Normally, only invaders are destroyed by the complement cascade as your own cells are protected against its attacks. However, patients with PNH possess red blood cells that are defective and are not properly protected, which results in the destruction of these defective red blood cells by your own immune system. The breakdown of a large number of red blood cells in this manner has been shown to trigger episodes of PNH. Based on the research conducted prior to this Phase Ib study, the investigational study drug, which is a peptide (small protein), is expected to decrease the activity of the complement cascade and prevent the destruction of defective red blood cells. Results from the ongoing/completed studies also provided good assurance of the safety for the doses of APL-2 in this study.

c. Dosing of APL-2

APL-2 will continue to be given daily, as a subcutaneous (the fat tissue under your skin) injection and it will be given either, by a nurse in the clinic, or by a nurse who visits your home, workplace, or other location agreed by you.

You have already received APL-2 for 364 days. You are being invited to continue to receive APL-2 for up to 729 days

The safety monitoring committee will continue to review all the safety data available from participants already dosed in the study approximately every 8 weeks.

The study can be stopped at any time, based on evaluation of the side effects of the study medication.

2. WHAT WILL HAPPEN IF YOU CONTINUE TO TAKE PART IN THIS STUDY

a. What will you be responsible for if you continue to take part in this study?

If you decide to continue to take part in this study you will be asked to sign this ethics committee-approved informed consent form and you must meet specific entry rules.

You must also agree to the following:

- Be willing and able to attend all visits at the study center and be available for the off-site nurse visits.
- Immunisation is required to participate in the study. Vaccinations may be given during the screening visit if you have not received the required immunisation within 2 years of the screening visit. There immunisations will be free as they are required as part of the trial. N.B. no further immunisation is required for your continued participation in the study (up to 364 days of treatment)

- Be willing to allow frequent blood tests during the study and at subsequent follow up visits.
- You will be requested to keep the 'study-supplied small freezer plugged in to a power source during your study treatment. If there is a power failure, you should contact the community nurse or the clinic site.
- Follow the instructions given by the study doctor and study staff.
- Take an antibiotic throughout the study to prevent infections.
- Have all procedures performed.
- Males must agree to no sperm donation at any time during the study.
- Females who are able to have children must agree to use an effective contraception for the duration of the study. Your study doctor can advise you on effective methods of contraception.
- Make sure you take only medications approved by a study doctor. Tell the study doctor what medications you are currently taking or have taken in the past. Also, tell him / her before starting any new medications and/or if there are changes to your current medications.

b. What types of tests or procedures will be involved with continuation in this study?

You will receive daily injections which will be done by the study staff when you visit the clinic site or by a trained study nurse at home, workplace, or other location convenient to you.

[insert study doctor]will contact your regular doctor (GP) about your continued study participation. If you do not want your GP told, you will not be able to continue to take part in the study. We may need to contact your GP about any health issues or parts of your medical history that might affect your continuation in the study.

You were given a Participant Identification Card when you started in this study, stating the name of the study and the study doctor's contact information. **This card should continue to be carried with you at all times** so that you can contact the study doctor at any time and so you can show it to any other doctor or dentist that you might visit while in this study.

c. What are the study details? What procedures will you have?

Your total time in this study is expected to be approximately 414 days. If more tests are needed, your study doctor may ask you to schedule additional visits which could extend your total time in the study. Your study doctor or Apellis may choose to withdraw you from this research study at any time which could decrease your total time in the study.

The study has 4 parts (you have already completed parts 1 and 2A):

- Part 1: You received APL-2 for 28 days and you visited the study centre at least 8 times and a community nurse conducted 22 visits in your home.
- Part 2A: There was evidence of clinical benefit to you by Day 28; therefore, you continued to receive APL-2 for a further 56 days. You visited the study centre 5 more times and a community nurse conducted 51 visits in your home.
- Part 2B: You may continue to receive daily APL-2 treatment for up to 364 days if there is ongoing evidence of clinical benefit to you following review of the available study data. Part 2B will include at least 10 visits to the study center and a community nurse will conduct all other visits in your home, workplace, or other location convenient to you. If there is evidence that you are no longer getting clinical benefit, treatment will be stopped and you will skip Part 2B and enter Part 3 for safety follow-up visits
- Part 2C: You may continue to receive daily APL-2 treatment for up to 729 days if there is ongoing evidence of clinical benefit to you following review of the available study data. Part 2C will include at least 6 visits to the study center. If there is evidence that you are no longer getting clinical benefit, treatment will be either dose increased or stopped and you will skip Part 2C and enter Part 3 for safety follow-up visits.
- Part 3: Safety follow-up will include 4 visits to the study center.

You will continue to be monitored throughout the study for any changes in health. Throughout the entire duration of the study, the [insert site]staff will continue to review any other medications you may be taking and any potential side effects to ensure your safety.

The following activities will be performed during site and off-site visits:

Site Visits (Day 1 onwards)

APL-2 dosing:

- You will receive doses of APL-2 on Day 85, 113, 141, 169, 197, 225, 253, 281, 309,337, 365, 421, 477, 561, 645 and 729 at the hospital clinic.

Other assessments done throughout the study:

- You will be asked what medications you are taking and if you have had any recent illnesses or changes in your condition.
- Preventative Antibiotics will be taken from Day 1 until 14 days after the last dose of APL-2
- Full physical exam
- An ECG (recording of the electrical activity of your heart).
- Set of vital signs (pulse, blood pressure and breathing rate) and temperature. If the visit includes study drug administration, vital signs will be measured before and after you receive the study drug.
- Set of routine blood and urine samples, blood samples relating to safety and PNH diagnosis
- You will be assessed for signs/symptoms of blood clots.
- Pregnancy test (for females who are able to become pregnant).
- FACIT (Fatigue Scale) is a 13 item questionnaire which you will need to complete during clinic visits on Days 85,141,197, 253, 309, 365 and421, 477, 561, 645 729, 743, 757 and 778.
- Monitor any side effects: you will be asked to notify of any unfavourable health or medical events, referred to as adverse events, experienced from the last visit.

Self- administration of APL-2 via Subcut pump

If you continue to participate in this study, the study drug that will be administered to you will be stored in your refrigerator. The study drug will be shipped directly to your home via courier. You will receive a new supply of study drug each month.

You will administer APL-2 via subcut pump at similar time each day. You will be asked to record time, date and location of injection daily in diary provided.

APL-2 dosing:

- From Day 85 to Day 729 daily doses of APL-2 will be given via subcut pump administered by yourself at your home or other location, with the exception of Days 85, 113, 141, 169, 197, 225, 253, 281, 309 , 337, 365, 421, 477, 561, 645 and 729 (see above).

Safety Follow-up

You will be asked to return to the clinic site for follow-up visits on Days743, 757 and 778.

After you have completed all follow-up procedures you need to return for the Exit Visit on Day 778.

You will not receive any study drug at these visits. These visits will include the following tests and procedures:

- You will be asked what medications you are taking and if you have had any illnesses or changes in your condition.

- Your vital signs (temperature, resting pulse and blood pressure, respiratory rate) will be measured.
- You will be assessed for signs/symptoms of blood clots.
- Electrocardiogram (ECG) – Day 743, 757 and 778.
- Physical examination – Day 743, 757 and778.
- Collection of a urine sample for routine lab tests.
  - For women who are able to get pregnant, urine will be collected at Day 743, 757 and 778for a pregnancy test.
- Collection of blood.
- You will be asked to complete a Fatigue Scale questionnaire at Day743, 757, and 778.

3. WHAT WILL HAPPEN TO MY TEST SAMPLES

The following blood samples will be collected throughout the study:

- Samples to determine regular routine blood counts and blood chemistry, to monitor your general health. (Haematology and Chemistry)
- Blood tests for HIV, Hepatitis B and Hepatitis C (Serology)
- Blood tests to determine how quickly your blood clots (Coagulation Profile)
- Pregnancy Test (If applicable)

All these routine samples will be sent to a Central lab overseas for testing and destroyed at the end of the study by internationally accepted means e.g. incineration.

The following blood samples will also be collected throughout the study. These samples will be sent to different locations for testing.

- Blood samples which determine the level of study drug in your blood (Pharmacokinetics).
- Blood samples to determine the effects of study drug on your body (Pharmacodynamics).
- Blood samples for antigenicity (The ability to cause the production of antibodies to the study drug).
- Blood samples for cytometry testing (This test is able to identify the type/amount of cells in different parts of the cell cycle).

These blood samples may be stored for up to 1 year after the end of treatment after which time they will be destroyed by internationally accepted means e.g. incineration.

The maximum amount of blood collected from each participant during the study will be approximately 580 mls (Approximately 39 tablespoons).

All samples will be coded; identified only by each participant's study number and initials. Only hospital and laboratory staff will have access to the blood samples.

Additional information if you are from Maori descent:

We understand that many Maori consider their tissue to be tapu and that participation in this type of study requires careful consideration. You may wish to discuss the study with your whanau or obtain the blessing of your iwi for participation in this research. They may have a position on participation in these types of studies and issues such as sending blood samples overseas and access to results of the study. Should you have any concerns regarding appropriate practice/tikanga to address cultural issues arising from your participation in the study it is recommended you consult with a kaumatua.

4. SAFETY: POTENTIAL RISKS AND DISCOMFORTS

a. Are there any risks from your contued participation in this study?

There may be risks to continuing in this study from APL-2 or from some of the procedures or tests done in this study.

If you continue to take part in this study, you or your family members should tell the study doctor or *[insert site]* staff immediately if you have any unusual health problems, injuries, or side effects, even if you do not think these problems are caused by the study or APL-2.

If there is any new important safety information or other information that could affect your willingness to participate in this study, your study doctor will let you know.

b. What are the likely risks with APL-2?

You may feel discomfort during some of the tests and study procedures. Possible risks, side effects, and discomforts include:

- Infections – you may be at increased risk for infections caused by certain types of organisms such as Streptococcus pneumonia, Neisseria Meningitidis, or Haemophilus influenza.
- Allergic reactions – all drugs have a potential risk of an allergic reaction, which if not treated promptly, could become life-threatening.
- Blood samples - you may have discomfort or pain when your blood is collected. You may feel faint or pass out. There is also a slight possibility of infection, bruising, or bleeding at the puncture site. Your anaemia (low red blood cell count) could worsen from having frequent blood draws
- ECG - skin irritation is rare but could occur during an ECG from the electrodes or gel that is used
- Antibiotic therapy – there is a possibility that you will experience some side effects from the antibiotic treatment you will need to take during this study. Side effects such as nausea, sickness, diarrhoea and skin rashes may occur and if you experience any of these you should inform the nurse or doctor. The doctor may decide that you should change to a different antibiotic to try and reduce these symptoms.
- Injection site reactions - there is a possibility that you could experience redness, swelling, and pain or tenderness at the site of injection. There is also a slight possibility of infection.

c. Any unforeseen risks?

Since the study drug APL-2 is investigational when taken alone or in combination with other medications, there may be other risks that are unknown.

d. Could APL-2 be harmful to an unborn or breastfed baby?

You should discuss pregnancy risks with your doctor before signing this consent form. Women who are pregnant, planning to become pregnant or breast feeding may not participate in this research study. Taking the study drug may involve unknown risks to a pregnant woman, an embryo, fetus (unborn baby) or nursing infant. If you (or your partner) become pregnant while in this study, the sponsor may ask to follow the outcome of the pregnancy. The study drug will be stopped and your participation in this study will be ended.

Before continuing in this research study and during the study, females able to have children will have pregnancy tests done. Talk to your doctor about the best method of birth control to use while you are in this study. It is important that you call your study doctor at [insert site details]right away if you become pregnant or father a child during the course of this study.

5. BENEFITS OF PARTICIPATION

Are there any benefits to your continued participation in this study?

Your study doctor believes that you have shown clinical benefit from participation in the research study to date. You may or may not benefit from continued participation in this study. There is no guarantee that you will continue to benefit from participating in this study. The information from this study might help to develop better treatments in the future for people with PNH. An indirect benefit of taking part in this study is that you will receive free medical tests at screening and during the study.

What are the alternatives to taking part? Do you have any other choices?

You do not have to continue in this study to receive treatment for your PNH. You have the option to receive standard treatment for PNH. Standard treatment may include medications such as prednisone, or blood thinners; blood transfusions; or bone marrow transplantation. The study doctor will discuss with you the risks and benefits of the alternative treatments.

6. POTENTIAL COSTS/ REIMBURSEMENTS

There will be no charge to you for your participation in this study. The study drug, study-related procedures, and study visits will be provided at no charge to you.

Will you be paid for your samples (such as blood or urine) or medical information?

You will not be paid for the use of your samples or any information, discoveries or inventions obtained from your samples or medical information.

You will be reimbursed for any reasonable travel, accommodation, parking, meals and other expenses associated with the research project visit, this will based on the distance that you have to travel to the hospital.

7. WHAT ARE THE RIGHTS OF PARTICIPANTS IN THIS STUDY?

a. Participation is voluntary

Participation in any research project is voluntary. If you do not wish to take part, you do not have to. If you decide to take part and later change your mind, you are free to withdraw from the project at any stage. This will not affect your routine treatment, your relationship with those treating you or with[insert site].

If you do decide to take part, you will be given this Participant Information and Consent Form to sign and you will be given a copy to keep.

b. New information

Sometimes during the course of a research project, new information becomes available about the treatment that is being studied. If this happens, your study doctor will tell you about it and discuss with you whether you want to continue in the research project. If you decide to withdraw, your study doctor will make arrangements for your regular health care to continue. If you decide to continue in the research project you may be asked to sign an updated consent form.

Also, on receiving new information, your study doctor might consider it to be in your best interests to withdraw you from the research project. If this happens, he/ she will explain the reasons and arrange for your regular health care to continue.

c. Right to access information collected during the study

In accordance with relevant New Zealand privacy and other relevant laws, you have the right to request access to your information collected and stored by the research team. You also have the right to request that any information with which you disagree be corrected. Please contact the study team member named at the end of this document if you would like to access your information.

8. CONFIDENTIALITY

By signing the consent form you consent to the study doctor and relevant research staff collecting and using personal information about you for the research project. Any information obtained in connection with this research project that can identify you will remain confidential. Your name, address or phone number will not be on the study forms; instead you will be identified by a unique study number, date of birth and initials. Your information will only be used for the purpose of this research project and it will only be disclosed with your permission, except as required by law.

Information about you may be obtained from your health records held at this and other health services for the purpose of this research. By signing the consent form you agree to the study team accessing health records if they are relevant to your participation in this research project.

Your health records and any information obtained during the research project are subject to inspection (for the purpose of verifying the procedures and the data) by the relevant authorities (such as the Ethics Committee that has reviewed and approved this study) and authorised representatives of the Sponsor, Apellis, and [insert site]or as required by law. All personnel reviewing your medical records will be required to keep the information confidential in accordance with law. By signing the Consent Form, you authorise release of, or access to, this confidential information to the relevant study personnel and regulatory authorities as noted above.

When the research project ends the study data must be analysed, so the results of the study may not be available until about six months after the research finishes. The study doctors and/or Apellis may decide to discuss or publish the results of the study. This may include publication in journals, presentation at conferences or other professional forums. In any publication, information will be provided in such a way that you cannot be identified. Results of the study will be provided to you, if you wish.

Information about your participation in this research project may be recorded in your health records.

Any information obtained for the purpose of this research project that can identify you will be treated as confidential and securely stored. It will be disclosed only with your permission, or as required by law.

Upon completion of the trial, the study file containing information about you will be archived for a minimum of fifteen years.

9. COMPENSATION FOR INJURY

If you were injured as a result of treatment given as part of this study, which is unlikely, you won't be eligible for compensation from ACC. However, compensation would be available from the study's sponsor, Apellis, in line with industry guidelines. We can give you a copy of these guidelines if you wish.

If you have private health or life insurance, you may wish to check with your insurer that taking part in this study won't affect your cover.

10. WHAT WILL HAPPEN AFTER THE STUDY ENDS, OR IF YOU PULL OUT?

If you decide to withdraw
Please notify a member of the research team before you withdraw. This notice will allow that person or the research supervisor to discuss any health risks or special requirements linked to withdrawing, such as follow up visits.

If you do withdraw your consent during the research project, information already collected will be retained to ensure that the results of the research project can be measured properly and to comply with law. You should be aware that data collected by the sponsor up to the time you withdraw will form part of the research project results. If you do not want them to do this, you must tell them before you join the research project.

Why the study might be unexpectedly stopped
This research project may be stopped unexpectedly for a variety of reasons. These may include reasons such as:
- Unacceptable side effects
- The drug being shown not to be effective
- The drug being shown to work and not need further testing.

Will the study treatment continue to be available after the study finished?

If it is found the drug does continue to have a beneficial effect in reducing haemolysis (breakdown of red blood cells), and no safety concerns are raised, the company may, at its sole discretion, after consultation with the Health and Disability Ethics Committees (HDECs) of New Zealand's Standing Committee on Therapeutic Trials (SCOTT), elect to continue the drug therapy by offering a further extension to the study.

Where can you go for more information about the study, or to raise concerns or complaints?

If you have any questions, concerns or complaints about the study at any stage, you can contact:

Lead study doctor [insert detail]
Phone: [insert details]
Research Coordinator: [insert detail]

If you want to talk to someone who isn't involved with the study, you can contact an independent health and disability advocate. Specialist advocates, are available here.

Phone:	0800 555 050
Fax:	0800 2 SUPPORT (0800 2787 7678)
Email:	advocacy@hdc.org.nz

You can also contact the health and disability ethics committee (HDEC) that approved this study on:

Phone:	0800 4 ETHICS (438 442)
Email:	hdecs@moh.govt.nz

For Maori health support, or to discuss any concerns or issues regarding this study, please contact Maori Health team, Phone:[insert details].

12. DO I HAVE TO DECIDE STRAIGHT AWAY?

No, you do not have to decide straight away. You should take some time to consider whether or not to continue your participation in the study. Study staff will let you know when they will need your decision. The following steps are useful in helping you reach a decision.

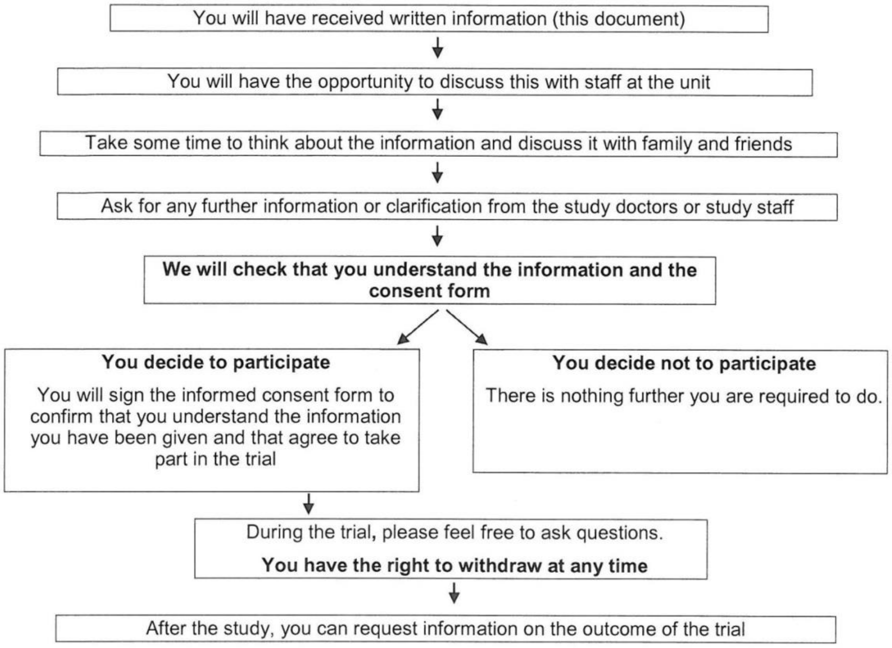
You will have received written information (this document)
You will have the opportunity to discuss this with staff at the unit
Take some time to think about the information and discuss it with family and friends
Ask for any further information or clarification from the study doctors or study staff
We will check that you understand the information and the consent form
You decide to participate
You will sign the informed consent form to confirm that you understand the information you have been given and that agree to take part in the trial
You decide not to participate
There is nothing further you are required to do.
During the trial, please feel free to ask questions.
You have the right to withdraw at any time
After the study, you can request information on the outcome of the trial

[INSERT SITE LOGO]

INFORMED CONSENT FORM

Short Title Nocturnal Hemoglobinuria (PNH) – PADDOCK

Protocol Number APL2-CP-PNH-204

Principal Investigator [insert details]

Declaration by participant:
I have read and I understand this version of the Participant Information Sheet. I have had the opportunity to ask questions and I am satisfied with the answers I have received.

I have had the opportunity to discuss the study with my family/whanau.

I freely agree to continue to participate in this study and understand that I may withdraw from the study at any time.

I understand that:

- My continued participation in this study is confidential and I will not be identified in study reports.
- My medical records may be reviewed by Apellis, companies working with them, auditors and health or regulatory agencies for the purpose of checking the accuracy of the information recorded for the study
- Some of my samples will be sent overseas for analysis and they will be destroyed as described in the Information Sheet.
- If I decide to withdraw from the study, information already collected may continue to be processed. I may be asked to attend follow-up visits.
- The study drugs or tests will be stopped if they appear harmful to me.
- I should contact site staff if I have any side effects.
- My GP and/or specialist will be informed of my participation in this study and relevant medical records may be accessed.
- I am not giving up any of my legal rights by signing this consent form.
- This trial is being carried out for the principal benefit of Apellis. If I suffer an injury as a result of my participation in the trial, they will pay me compensation in line with industry guidelines.
- I understand that there may be risks associated with the treatment in the event of myself or my partner becoming pregnant. I undertake to inform my partner of the risks and to take responsibility for the prevention of pregnancy

Statement by Participant I hereby consent to take part in this study. I understand that I will receive a signed copy of this consent form for my records.

________________________________ (full name)

________________________________ (signature)

__ __ / __ __ __ / __ __ (Date)

Statement by Consenter (Investigator/designee) I have discussed this study with the above named participant. The participant appeared to fully understand the information provided about the study.

________________________________ (full name)

________________________________ (signature)

__ __ / __ __ __ / __ __ (Date)

Insert Header with institution's name or institution's letterhead

PARTICIPANT INFORMATION SHEET

[Insert site name]

**Short Title**	A Phase Ib, Open Label, Multiple Ascending Dose, Pilot Study to Assess the Safety, Preliminary Efficacy and Pharmacokinetics of Subcutaneously Administered APL-2 in Subjects with Paroxysmal Nocturnal Hemoglobinuria (PNH). – PADDOCK		
**Protocol Number**	APL2-CP-PNH-204	**Ethics Number**	<<Insert #>>
**Project Sponsor**	Apellis Pharmaceuticals, Inc.<br>6400 Westwind Way, Suite A, Louisville, KY, 40014		
**Local Sponsor**	Novotech		
**Principal Investigator**	<<Insert Name>>		
**Location**	<<Insert Location>>		
**Study related phone number**	<<Insert Contact Number>>		

You are being invited to take part in the second cohort of a research study of the trial drug APL-2. This consent form gives you important information about this study to help you decide if you want to take part in the research study. It describes the purpose of this study, the study procedures, and the possible risks, and provides information about your rights as a study participant.

Please take the time to read this information carefully. You should talk to your study doctor and study staff about this study and ask any questions you have. You can also discuss this study with other people such as your family or general practitioner (GP). If you decide to take part in this study, you will be asked to sign and date this form. You will receive a copy of the signed form. Your study doctor will tell your GP about your participation in this study.

Who is funding this study?

Apellis Pharmaceuticals, Inc. ("Apellis") is funding this clinical study. Apellis is the "sponsor" of the study, which means that Apellis takes responsibility for starting, managing, and financing the study. <<Insert Site Name>> is being paid by Apellis to do this study.

Voluntary participation and withdrawal from this study

Your participation in this study is voluntary. You are free to say yes or no. Even if you join this study, you may stop at any time.

A description of this clinical trial will be available on the internet at http://www.ClinicalTrials.gov. This website will not include information that can identify you. At most, the website will include a summary of the results. You can search this website at any time.

1. WHAT YOU SHOULD KNOW ABOUT THIS STUDY

a. Why is this study being done?

This is a research study to test a new investigational drug. An investigational drug is one that is not approved by any regulatory authority (e.g. FDA, Department of Health, Medsafe etc) for use outside a clinical trial. Cohort 1 has completed and 2 patients have already completed in excess of 169 days of dosing in Cohort 2. The study was initially set up in New Zealand but insufficient eligible PNH patients exist to complete the study.

You are being invited to participate in this research study because you have been diagnosed with paroxysmal nocturnal hemoglobinuria (PNH). PNH is a rare disorder that causes the red blood cells to break down too early. Many PNH patients suffer from complications such as blood clots or low red blood cell counts (anemia) even after receiving standard treatment.

The research study will continue to gather scientific information about the safety, tolerability and continued evidence of activity of the investigational drug APL-2 in people with PNH who have not been treated with Soliris® in the past. The study will help determine how APL-2 behaves inside the body, how it is eliminated from the body, the effect it has on the body after multiple doses and if it can prevent red blood cells from breaking down.

b. How does APL-2 works?

Your body has numerous biological mechanisms designed to attack and kill off invaders, such as bacteria. One of these mechanisms, called the complement cascade, is part of your normal immune system. Normally, only invaders are destroyed by the complement cascade as your own cells are protected against its attacks. However, patients with PNH possess red blood cells that are defective and are not properly protected, which results in the destruction of these defective red blood cells by your own immune system. The breakdown of a large number of red blood cells in this manner has been shown to trigger episodes of PNH. Based on the research conducted prior to this Phase Ib study, the investigational study drug, which is a peptide (small protein), is expected to decrease the activity of the complement cascade and prevent the destruction of defective red blood cells. Results from the ongoing/completed studies also provided good assurance of the safety for the doses of APL-2 in this study.

c. Dosing of APL-2

APL-2 will be given daily, as a subcutaneous (the fat tissue under your skin) infusion using an infusion pump that you will be trained to use and self administer when fully trained and competent in the use of the pump. Until that time, you will attend the clinical site to receive your study medication.

You will receive 270 mg APL-2 each day for up to 364 days, although the study doctor may increase your dose up to 360 mg APL-2 each day if a higher dose is needed to reduce breakdown of your red blood cells

The safety monitoring committee will review all the safety data available from participants already dosed in the study approximately every 8 weeks.

The study can be stopped at any time, based on evaluation of the side effects of the study medication.

2. WHAT WILL HAPPEN IF YOU TAKE PART IN THIS STUDY

a. What will you be responsible for if you take part in this study?

If you decide to take part in this study you will be asked to sign this ethics committee-approved informed consent form and you must meet specific entry rules.

You must also agree to the following:

- Be willing and able to attend all visits at the study center.
- Immunisation is required to participate in the study. Vaccinations may be given during the screening visit if you have not received the required immunisation within 2 years of the screening visit. There immunisations will be free as they are required as part of the trial. A booster vaccine will be given on Day 57 if required.
- Be willing to allow frequent blood tests during the study and at subsequent follow up visits.
- You will be requested to keep the study drug supplies in your refrigerator (between 0-4°C) If there is a power failure, you should contact the clinic site.
- Follow the instructions given by the study doctor and study staff.
- Take an antibiotic throughout the study to prevent bacterial infections.
- Take an antiviral throughout the study if you are a hepatitis B carrier, to prevent reactivation of the hepatitis B virus.
- Have all procedures performed.
- Males must agree to no sperm donation at any time during the study.
- Females who are able to have children must agree to use an effective contraception for the duration of the study. Your study doctor can advise you on effective methods of contraception.
- Make sure you take only medications approved by a study doctor. Tell the study doctor what medications you are currently taking or have taken in the past. Also, tell him / her before starting any new medications and/or if there are changes to your current medications.

b. What types of tests or procedures will be involved with continuation in this study?

You will receive / administer daily subcutaneous infusions which will be done by the study staff when you visit the clinic site or by you after you have been fully trained to self administer using the infusion pump supplied for sole use in this study.

<<Insert Site Name>> will contact your regular doctor (GP) about your study participation. If you do not want your GP told, you will not be able to continue to take part in the study. We may need to contact your GP about any health issues or parts of your medical history that might affect your participation in the study.

You will be given a Participant Identification Card when you start this study, stating the name of the study and the study doctor's contact information. **This card should be carried with you at all times** so that you can contact the study doctor at any time and so you can show it to any other doctor or dentist that you might visit while in this study.

c. What are the study details? What procedures will you have?

Your total time in this study is expected to be approximately 414 days. If more tests are needed, your study doctor may ask you to schedule additional visits which could extend your total time in the study. Your study doctor or Apellis may choose to withdraw you from this research study at any time which could decrease your total time in the study.

The study has 4 parts;

- Part 1: You will receive APL-2 for 28 days and you will visit the study centre at least 8 times.
- Part 2A: If there is evidence of clinical benefit to you by Day 28; you will continue to receive APL-2 up to Day 84. You will visit the study centre 5 more times.
- Part 2B: If there is still evidence of clinical benefit to you by Day 84, you will continue to receive daily APL-2 treatment up to Day 364. Part 2B will include at least 10 visits to the study center. If there is evidence that you are no longer getting clinical benefit, treatment will be stopped and you will skip Part 2B and enter Part 3 for safety follow-up visits
- Part 3: Safety follow-up will include 4 visits to the study center.

You will be monitored throughout the study for any changes in health. Throughout the entire duration of the study, the <<Insert Site Name>> staff will continue to review any other medications you may be taking and any potential side effects to ensure your safety.

The following activities will be performed during site visits:

**Screening**<br>**Screening Visits (2 visits) Day -30 to Day -1**
Before we can do any tests, you will be asked to give written informed consent, below are details of assessments that need to be done:<br>• Questions about your health and what medication you are taking<br>• Review of your demographic (personal) information such as date of birth, ethnicity, and race<br>• Review the study requirements and restrictions<br>• Height and weight<br>• Set of vital signs (pulse, blood pressure and breathing rate) and temperature<br>• Full physical exam<br>• An ECG, which is a painless test that looks at the electrical activity of your heart by putting small sticky patches on certain areas of your body while you are lying down. These patches are connected to thin wires which are again connected to a machine which will interpret the electrical activity of your heart and then print a report.<br>• Set of routine blood and urine samples, blood samples relating to safety and PNH diagnosis<br>• Blood tests for HIV, Hepatitis B and Hepatitis C<br>• Pregnancy test (females) +/- hormone test (postmenopausal females)<br>• Vaccination if required will be done during the screening period after your eligibility has been confirmed. Your doctor will discuss with you what vaccinations you may need and will also take into account vaccinations that you have previously received.
**A final decision about whether you can take part will then be made when all your screening results are available.**

**Part 1: Site Visits (Day 1 – Day 28)**
**APL-2 dosing:**<br>• You will receive the first 3 daily doses of APL-2 (Day 1 to 3 or beyond. You will continue to attend the hospital clinic on a daily basis until you are competent and confident to use the infusion pump fo self administer the study drug at home), as well as doses on Day 8, 15 and 22 at the hospital clinic. You will need to stay in the clinic for 2.5 hours after receiving the first dose of APL-2 on Day 1 so we can make sure that you are okay.<br><br>**Other assessments done throughout the study:**<br>• You will be asked what medications you are taking and if you have had any recent illnesses or changes in your condition.<br>• Preventative Antibiotics will be taken from Day 1 until 14 days after the last dose of APL-2<br>• Preventative Antivirals will be taken from Day 1 until 14 days after the last dose of APL-2 if you are a hepatitis B carrier<br>• Full physical exam<br>• An ECG (recording of the electrical activity of your heart).<br>• Set of vital signs (pulse, blood pressure and breathing rate) and temperature. If the visit includes study drug administration, vital signs will be measured before and after you receive the study drug.<br>• Set of routine blood and urine samples, blood samples relating to safety and PNH diagnosis<br>• You will be assessed for signs/symptoms of blood clots.<br>• Pregnancy test (for females who are able to become pregnant).<br>• FACIT (Fatigue Scale) is a 13 item questionnaire which you will need to complete during clinic visits on Days 1 and 15.

- Monitor any side effects: you will be asked to notify of any unfavourable health or medical events, referred to as adverse events, experienced from the last visit.
- Vaccination (booster) on Day 57 (during Part 2A or Part 3 Follow up)

You will self administer the study drug on a daily basis (after you have been trained on the use of the infusion pump) on the following study days 4 (or from when you feel confident and are considered competent to self administer) -7, 9-14, 16-21, and 23-28

Part 2A: Site Visits (Day 29 – Day 84)

APL-2 dosing:

You will receive doses on Day 29, 36, 43, 57 and 71 at the hospital clinic. You will self administer the study drug on a daily basis on the following study days 30-35, 37-42, 44-56, 58-70 and 72-84.

Other assessments done throughout the study:

- You will be asked what medications you are taking and if you have had any recent illnesses or changes in your condition.
- Preventative Antibiotics will be taken from Day 1 until 14 days after the last dose of APL-2
- Preventative Antivirals will be taken from Day 1 until 14 days after the last dose of APL-2 if you are a hepatitis B carrier

- Full physical exam
- An ECG (recording of the electrical activity of your heart).
- Set of vital signs (pulse, blood pressure and breathing rate) and temperature. If the visit includes study drug administration, vital signs will be measured before and after you receive the study drug.
- Set of routine blood and urine samples, blood samples relating to safety and PNH diagnosis
- You will be assessed for signs/symptoms of blood clots.
- Pregnancy test (for females who are able to become pregnant).
- FACIT (Fatigue Scale) is a 13 item questionnaire which you will need to complete during clinic visits on Days 29, 43 and 71.
- Monitor any side effects: you will be asked to notify of any unfavourable health or medical events, referred to as adverse events, experienced from the last visit.
- Vaccination (booster) on Day 57

Part 2B: Site Visits (Day 85 onwards)

APL-2 dosing:

- You will receive doses of APL-2 on Day 85, 113, 141, 169, 197, 225, 253, 281, 309 and 337 at the hospital clinic. You will self administer the study drug on a daily basis on all other days in between your hospital clinic visits.

Other assessments done throughout the study:

- You will be asked what medications you are taking and if you have had any recent illnesses or changes in your condition.
- Preventative Antibiotics will be taken from Day 1 until 14 days after the last dose of APL-2
- Preventative Antivirals will be taken from Day 1 until 14 days after the last dose of APL-2 if you are a hepatitis B carrier

- Full physical exam
- An ECG (recording of the electrical activity of your heart).
- Set of vital signs (pulse, blood pressure and breathing rate) and temperature. If the visit includes study drug administration, vital signs will be measured before and after you receive the study

drug.

- Set of routine blood and urine samples, blood samples relating to safety and PNH diagnosis
- You will be assessed for signs/symptoms of blood clots.
- Pregnancy test (for females who are able to become pregnant).
- FACIT (Fatigue Scale) is a 13 item questionnaire which you will need to complete during clinic visits on Days 85,141,197, 253, 309, 365 and 414.
- Monitor any side effects: you will be asked to notify of any unfavourable health or medical events, referred to as adverse events, experienced from the last visit.

Safety Follow-up

You will be asked to return to the clinic site for follow-up visits on Days 365, 379, and 393.

After you have completed all follow-up procedures you need to return for the Exit Visit on Day 414.

You will not receive any study drug at these visits. These visits will include the following tests and procedures:

- You will be asked what medications you are taking and if you have had any illnesses or changes in your condition.
- Your vital signs (temperature, resting pulse and blood pressure, respiratory rate) will be measured.
- You will be assessed for signs/symptoms of blood clots.
- Electrocardiogram (ECG) – Day 365.
- Physical examination – Day 365 and 414.
- Collection of a urine sample for routine lab tests.
    - For women who are able to get pregnant, urine will be collected at Day 365, 393 and 414 for a pregnancy test.
- Collection of blood.
- You will be asked to complete a FACIT (Fatigue Scale) questionnaire at Day 365, 393 and 414.

3. WHAT WILL HAPPEN TO MY TEST SAMPLES

The following blood samples will be collected throughout the study:

- Samples to determine regular routine blood counts and blood chemistry, to monitor your general health. (Haematology and Chemistry)
- Blood tests for HIV, Hepatitis B and Hepatitis C (Serology)
- Blood tests to determine how quickly your blood clots (Coagulation Profile)
- Pregnancy Test (If applicable)

All these routine samples will be sent to a Central lab overseas for testing and destroyed at the end of the study by internationally accepted means e.g. incineration.

The following blood samples will also be collected throughout the study. These samples will be sent to different locations for testing.

- Blood samples which determine the level of study drug in your blood (Pharmacokinetics).
- Blood samples to determine the effects of study drug on your body (Pharmacodynamics).
- Blood samples for immunogenicity (The ability to cause the production of antibodies to the study drug).
- Blood samples for cytometry testing (This test is able to identify the type/amount of cells in different parts of the cell cycle).

These blood samples may be stored for up to 1 year after the end of treatment after which time they will be destroyed by internationally accepted means e.g. incineration.

The maximum amount of blood collected from each participant during the study will be approximately 580 mL (Approximately 39 tablespoons).

All samples will be coded; identified only by each participant's study number and initials. Only hospital and laboratory staff will have access to the blood samples.

4. SAFETY: POTENTIAL RISKS AND DISCOMFORTS

a. Are there any risks from your participation in this study?

There may be risks to taking part in this study from APL-2 or from some of the procedures or tests done in this study.

If you decide to take part in this study, you or your family members should tell the study doctor or <<Insert Site Name>> staff immediately if you have any unusual health problems, injuries, or side effects, even if you do not think these problems are caused by the study or APL-2.

If there is any new important safety information or other information that could affect your willingness to participate in this study, your study doctor will let you know.

b. What are the likely risks with APL-2?

You may feel discomfort during some of the tests and study procedures. Possible risks, side effects, and discomforts include:

- Infections – you may be at increased risk for infections caused by certain types of bacteria such as Streptococcus pneumonia, Neisseria Meningitidis, or Haemophilus influenza.
- Allergic reactions – all drugs have a potential risk of an allergic reaction, which if not treated promptly, could become life-threatening.
- Blood samples - you may have discomfort or pain when your blood is collected. You may feel faint or pass out. There is also a slight possibility of infection, bruising, or bleeding at the puncture site. Your anaemia (low red blood cell count) could worsen from having frequent blood draws
- ECG - skin irritation is rare but could occur during an ECG from the sticky patches or gel that is used
- Antibiotic and antiviral therapy – there is a possibility that you will experience some side effects from the antibiotic treatment you will need to take during this study. Side effects such as nausea, sickness, diarrhoea, dizziness, thrush, and skin rashes may occur and if you experience any of these you should inform the nurse or doctor. The doctor may decide that you should change to a different antibiotic to try and reduce these symptoms.
- Injection site reactions - there is a possibility that you could experience redness, swelling, and pain or tenderness at the site of injection. There is also a slight possibility of infection.

c. Any unforeseen risks?

Since the study drug APL-2 is investigational when taken alone or in combination with other medications, there may be other risks that are unknown.

d. Could APL-2 be harmful to an unborn or breastfed baby?

You should discuss pregnancy risks with your doctor before signing this consent form. Women who are pregnant, planning to become pregnant or breast feeding may not participate in this research study. Taking the study drug may involve unknown risks to a pregnant woman, an embryo, fetus (unborn baby) or nursing infant. If you (or your partner) become pregnant while in this study, the sponsor may ask to follow the outcome of the pregnancy. The study drug will be stopped and your participation in this study will be ended.

Before taking part in this research study and during the study, females able to have children will have pregnancy tests done. Talk to your doctor about the best method of birth control to use while you are in this study. It is important that you call your study doctor at <<***insert phone number***>> right away if you become pregnant or father a child during the course of this study.

5. BENEFITS OF PARTICIPATION

Are there any benefits to your participation in this study?

You may or may not benefit from participation in this study. There is no guarantee that you will continue to benefit from participating in this study. The information from this study might help to develop better treatments in the future for people with PNH. An indirect benefit of taking part in this study is that you will receive free medical tests at screening and during the study.

What are the alternatives to taking part? Do you have any other choices?

You do not have to be in this study to receive treatment for your PNH. You have the option to receive standard treatment for PNH. Standard treatment may include medications such as prednisone, or blood thinners; blood transfusions; or bone marrow transplantation. The study doctor will discuss with you the risks and benefits of the alternative treatments.

6. POTENTIAL COSTS/ REIMBURSEMENTS

There will be no charge to you for your participation in this study. The study drug, study-related procedures, and study visits will be provided at no charge to you.

Will you be paid for your samples (such as blood or urine) or medical information?

You will not be paid for the use of your samples or any information, discoveries or inventions obtained from your samples or medical information.

You will be reimbursed for any reasonable travel, accommodation, parking, meals and other expenses associated with the research project visit, this will based on the distance that you have to travel to the hospital.

7. WHAT ARE THE RIGHTS OF PARTICIPANTS IN THIS STUDY?

a. Participation is voluntary

Participation in any research project is voluntary. If you do not wish to take part, you do not have to. If you decide to take part and later change your mind, you are free to withdraw from the project at any stage. This will not affect your routine treatment, your relationship with those treating you or with <<Insert Site Name>> .

If you do decide to take part, you will be given this Participant Information and Consent Form to sign and you will be given a copy to keep.

b. New information

Sometimes during the course of a research project, new information becomes available about the treatment that is being studied. If this happens, your study doctor will tell you about it and discuss with you whether you want to continue in the research project. If you decide to withdraw, your study doctor will make arrangements for your regular health care to continue. If you decide to continue in the research project you may be asked to sign an updated consent form.

Also, on receiving new information, your study doctor might consider it to be in your best interests to withdraw you from the research project. If this happens, he/ she will explain the reasons and arrange for your regular health care to continue.

c. Right to access information collected during the study

In accordance with relevant New Zealand privacy and other relevant laws, you have the right to request access to your information collected and stored by the research team. You also have the right to request that any information with which you disagree be corrected. Please contact the study team member named at the end of this document if you would like to access your information.

8. CONFIDENTIALITY

By signing the consent form you consent to the study doctor and relevant research staff collecting and using personal information about you for the research project. Any information obtained in connection with this research project that can identify you will remain confidential. Your name, address or phone number will not be on the study forms; instead you will be identified by a unique participant study number, date of birth and initials. Your information will only be used for the purpose of this research project and it will only be disclosed with your permission, except as required by law.

Information about you may be obtained from your health records held at this and other health services for the purpose of this research. By signing the consent form you agree to the study team accessing health records if they are relevant to your participation in this research project.

Your health records and any information obtained during the research project are subject to inspection (for the purpose of verifying the procedures and the data) by the relevant authorities (such as the Ethics Committee that has reviewed and approved this study) and authorised representatives of the Sponsor, Apellis, and <<Insert Site Name>> or as required by law. All personnel reviewing your medical records will be required to keep the information confidential in accordance with law. By signing the Consent Form, you authorise release of, or access to, this confidential information to the relevant study personnel and regulatory authorities as noted above.

When the research project ends the study data must be analysed, so the results of the study may not be available until about six months after the research finishes. The study doctors and/or Apellis may decide to discuss or publish the results of the study. This may include publication in journals, presentation at conferences or other professional forums. In any publication, information will be provided in such a way that you cannot be identified. Results of the study will be provided to you, if you wish.

Information about your participation in this research project may be recorded in your health records.

Any information obtained for the purpose of this research project that can identify you will be treated as confidential and securely stored. It will be disclosed only with your permission, or as required by law.

Upon completion of the trial, the study file containing information about you will be archived for a minimum of fifteen years.

9. COMPENSATION FOR INJURY

If you were injured as a result of treatment given as part of this study, compensation would be available from the study's sponsor, Apellis, in line with industry guidelines. We can give you a copy of these guidelines if you wish.

If you have private health or life insurance, you may wish to check with your insurer that taking part in this study won't affect your cover.

10. WHAT WILL HAPPEN AFTER THE STUDY ENDS, OR IF YOU PULL OUT?

If you decide to withdraw

Please notify a member of the research team before you withdraw. This notice will allow that person or the research supervisor to discuss any health risks or special requirements linked to withdrawing, such as follow up visits.

If you do withdraw your consent during the research project, information already collected will be retained to ensure that the results of the research project can be measured properly and to comply with law. You should be aware that data collected by the sponsor up to the time you withdraw will form part of the research project results. If you do not want them to do this, you must tell them before you join the research project.

Why the study might be unexpectedly stopped
This research project may be stopped unexpectedly for a variety of reasons. These may include reasons such as:
• Unacceptable side effects
• The drug being shown not to be effective
• The drug being shown to work and not need further testing.

Will the study treatment continue to be available after the study finished?

If it is found the drug does continue to have a beneficial effect in reducing haemolysis (breakdown of red blood cells), and no safety concerns are raised, the company may, at its sole discretion, elect to continue the drug therapy by offering a further extension to the study.

Where can you go for more information about the study, or to raise concerns or complaints?

If you have any questions, concerns or complaints about the study at any stage, you can contact:

<<Insert Name>>, lead study doctor
Phone: <<Insert Contact Number>>and ask operator to page or put you through to <<Insert Name>>
Research Coordinator: <<Insert Contact Number>>

If you want to talk to someone who isn't involved with the study, you can contact an independent health and disability advocate. Specialist advocates, are available here.

Phone:
Fax:
Email:

You can also contact the health and disability ethics committee that approved this study on:

Phone:
Email:

12. DO I HAVE TO DECIDE STRAIGHT AWAY?

No, you do not have to decide straight away. You should take some time to consider whether or not to continue your participation in the study. Study staff will let you know when they will need your decision. The following steps are useful in helping you reach a decision.

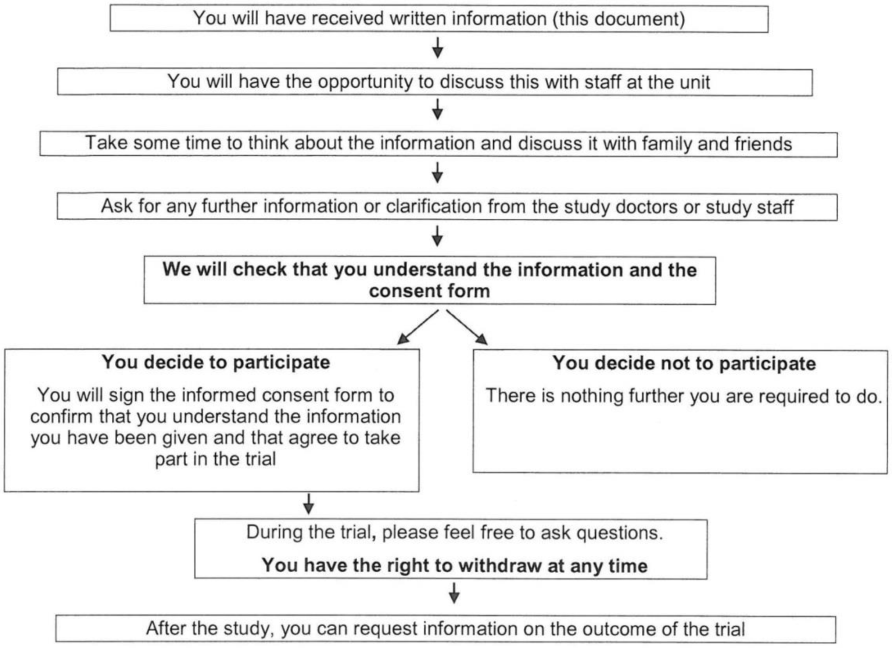
You will have received written information (this document)
You will have the opportunity to discuss this with staff at the unit
Take some time to think about the information and discuss it with family and friends
Ask for any further information or clarification from the study doctors or study staff
We will check that you understand the information and the consent form
You decide to participate
You will sign the informed consent form to confirm that you understand the information you have been given and that agree to take part in the trial
You decide not to participate
There is nothing further you are required to do.
During the trial, please feel free to ask questions.
You have the right to withdraw at any time
After the study, you can request information on the outcome of the trial

Insert Header with institution's name or institution's letterhead

INFORMED CONSENT FORM

**Short Title**	Nocturnal Hemoglobinuria (PNH) – PADDOCK
**Protocol Number**	APL2-CP-PNH-204
**Principal Investigator**	<<Insert Name>>

Declaration by participant:
I have read and I understand this version of the Participant Information Sheet. I have had the opportunity to ask questions and I am satisfied with the answers I have received.

I have had the opportunity to discuss the study with my family.

I freely agree to continue to participate in this study and understand that I may withdraw from the study at any time.

I understand that:

- My participation in this study is confidential and I will not be identified in study reports.
- My medical records may be reviewed by Apellis, companies working with them, auditors and health or regulatory agencies for the purpose of checking the accuracy of the information recorded for the study
- Some of my samples will be sent overseas for analysis and they will be destroyed as described in the Information Sheet.
- If I decide to withdraw from the study, information already collected may continue to be processed. I may be asked to attend follow-up visits.
- The study drugs or tests will be stopped if they appear harmful to me.
- I should contact site staff if I have any side effects.
- My GP and/or specialist will be informed of my participation in this study and relevant medical records may be accessed.
- I am not giving up any of my legal rights by signing this consent form.
- This trial is being carried out for the principal benefit of Apellis. If I suffer an injury as a result of my participation in the trial, they will pay me compensation in line with industry guidelines.
- I understand that there may be risks associated with the treatment in the event of myself or my partner becoming pregnant. I undertake to inform my partner of the risks and to take responsibility for the prevention of pregnancy

**Statement by Participant**	I hereby consent to take part in this study. I understand that I will receive a signed copy of this consent form for my records.<br><br>______________________________ (full name)<br><br>______________________________ (signature)<br><br>__ __ / __ __ __ / __ __ (Date)

Statement by Consenter (Investigator/designee) I have discussed this study with the above named participant. The participant appeared to fully understand the information provided about the study.

______________________________ (full name)

______________________________ (signature)

__ __ / __ __ __ / __ __ (Date)

Insert Header with institution’s name or institution’s letterhead

PARTICIPANT INFORMATION SHEET

[Insert site name]

**Short Title**	A Phase Ib, Open Label, Multiple Ascending Dose, Pilot Study to Assess the Safety, Preliminary Efficacy and Pharmacokinetics of Subcutaneously Administered APL-2 in Subjects with Paroxysmal Nocturnal Hemoglobinuria (PNH). – PADDOCK		
**Protocol Number**	APL2-CP-PNH-204	**Ethics Number**	<<Insert #>>
**Project Sponsor**	Apellis Pharmaceuticals, Inc.<br>6400 Westwind Way, Suite A, Louisville, KY, 40014		
**Local Sponsor**	Novotech		
**Principal Investigator**	<<Insert Name>>		
**Location**	<<Insert Location>>		
**Study related phone number**	<<Insert Contact Number>>		

You are being invited to take part in the second cohort of a research study of the trial drug APL-2. This consent form gives you important information about this study to help you decide if you want to take part in the research study. It describes the purpose of this study, the study procedures, and the possible risks, and provides information about your rights as a study participant.

Please take the time to read this information carefully. You should talk to your study doctor and study staff about this study and ask any questions you have. You can also discuss this study with other people such as your family or general practitioner (GP). If you decide to take part in this study, you will be asked to sign and date this form. You will receive a copy of the signed form. Your study doctor will tell your GP about your participation in this study.

Who is funding this study?

Apellis Pharmaceuticals, Inc. (“Apellis”) is funding this clinical study. Apellis is the "sponsor" of the study, which means that Apellis takes responsibility for starting, managing, and financing the study. <<Insert Site Name>> is being paid by Apellis to do this study.

Voluntary participation and withdrawal from this study

Your participation in this study is voluntary. You are free to say yes or no. Even if you join this study, you may stop at any time.

A description of this clinical trial will be available on the internet at http://www.ClinicalTrials.gov. This website will not include information that can identify you. At most, the website will include a summary of the results. You can search this website at any time.

1. WHAT YOU SHOULD KNOW ABOUT THIS STUDY

a. Why is this study being done?

This is a research study to test a new investigational drug. An investigational drug is one that is not approved by any regulatory authority (e.g. FDA, Department of Health, Medsafe etc) for use outside a clinical trial. Cohort 1 has completed and 2 patients have already completed in excess of 169 days of dosing in Cohort 2. The study was initially set up in New Zealand but insufficient eligible PNH patients exist to complete the study.

You are being invited to participate in this research study because you have been diagnosed with paroxysmal nocturnal hemoglobinuria (PNH). PNH is a rare disorder that causes the red blood cells to break down too early. Many PNH patients suffer from complications such as blood clots or low red blood cell counts (anemia) even after receiving standard treatment.

The research study will continue to gather scientific information about the safety, tolerability and continued evidence of activity of the investigational drug APL-2 in people with PNH who have not been treated with Soliris® in the past. The study will help determine how APL-2 behaves inside the body, how it is eliminated from the body, the effect it has on the body after multiple doses and if it can prevent red blood cells from breaking down.

b. How does APL-2 works?

Your body has numerous biological mechanisms designed to attack and kill off invaders, such as bacteria. One of these mechanisms, called the complement cascade, is part of your normal immune system. Normally, only invaders are destroyed by the complement cascade as your own cells are protected against its attacks. However, patients with PNH possess red blood cells that are defective and are not properly protected, which results in the destruction of these defective red blood cells by your own immune system. The breakdown of a large number of red blood cells in this manner has been shown to trigger episodes of PNH. Based on the research conducted prior to this Phase Ib study, the investigational study drug, which is a peptide (small protein), is expected to decrease the activity of the complement cascade and prevent the destruction of defective red blood cells. Results from the ongoing/completed studies also provided good assurance of the safety for the doses of APL-2 in this study.

c. Dosing of APL-2

APL-2 will be given daily, as a subcutaneous (the fat tissue under your skin) infusion using an infusion pump that you will be trained to use and self administer when fully trained and competent in the use of the pump. Until that time, you will attend the clinical site to receive your study medication.

You will receive 270 mg APL-2 each day for up to 364 days, although the study doctor may increase your dose up to 360 mg APL-2 each day if a higher dose is needed to reduce breakdown of your red blood cells

The safety monitoring committee will review all the safety data available from participants already dosed in the study approximately every 8 weeks.

The study can be stopped at any time, based on evaluation of the side effects of the study medication.

2. WHAT WILL HAPPEN IF YOU TAKE PART IN THIS STUDY

a. What will you be responsible for if you take part in this study?

If you decide to take part in this study you will be asked to sign this ethics committee-approved informed consent form and you must meet specific entry rules.

You must also agree to the following:

- Be willing and able to attend all visits at the study center.
- Immunisation is required to participate in the study. Vaccinations may be given during the screening visit if you have not received the required immunisation within 2 years of the screening visit. There immunisations will be free as they are required as part of the trial. A booster vaccine will be given on Day 57 if required.
- Be willing to allow frequent blood tests during the study and at subsequent follow up visits.
- You will be requested to keep the study drug supplies in your refrigerator (between 2-8°C) If there is a power failure, you should contact the clinic site.
- Follow the instructions given by the study doctor and study staff.
- Take an antibiotic throughout the study to prevent bacterial infections.
- Take an antiviral throughout the study if you are a hepatitis B carrier, to prevent reactivation of the hepatitis B virus.
- Have all procedures performed.
- Males must agree to no sperm donation at any time during the study.
- Females who are able to have children must agree to use an effective contraception for the duration of the study. Your study doctor can advise you on effective methods of contraception.
- Make sure you take only medications approved by a study doctor. Tell the study doctor what medications you are currently taking or have taken in the past. Also, tell him / her before starting any new medications and/or if there are changes to your current medications.

b. What types of tests or procedures will be involved with continuation in this study?

You will receive / administer daily subcutaneous infusions which will be done by the study staff when you visit the clinic site or by you after you have been fully trained to self administer using the infusion pump supplied for sole use in this study.

<<Insert Site Name>> will contact your regular doctor (GP) about your study participation. If you do not want your GP told, you will not be able to continue to take part in the study. We may need to contact your GP about any health issues or parts of your medical history that might affect your participation in the study.

You will be given a Participant Identification Card when you start this study, stating the name of the study and the study doctor's contact information. **This card should be carried with you at all times** so that you can contact the study doctor at any time and so you can show it to any other doctor or dentist that you might visit while in this study.

c. What are the study details? What procedures will you have?

Your total time in this study is expected to be approximately 414 days. If more tests are needed, your study doctor may ask you to schedule additional visits which could extend your total time in the study. Your study doctor or Apellis may choose to withdraw you from this research study at any time which could decrease your total time in the study.

The study has 4 parts;

- Part 1: You will receive APL-2 for 28 days and you will visit the study centre at least 8 times.
- Part 2A: If there is evidence of clinical benefit to you by Day 28; you will continue to receive APL-2 up to Day 84. You will visit the study centre 5 more times.
- Part 2B: If there is still evidence of clinical benefit to you by Day 84, you will continue to receive daily APL-2 treatment up to Day 364. Part 2B will include at least 10 visits to the study center. If there is evidence that you are no longer getting clinical benefit, treatment will be stopped and you will skip Part 2B and enter Part 3 for safety follow-up visits
- Part 3: Safety follow-up will include 4 visits to the study center.

You will be monitored throughout the study for any changes in health. Throughout the entire duration of the study, the <<Insert Site Name>> staff will continue to review any other medications you may be taking and any potential side effects to ensure your safety.

The following activities will be performed during site visits:

**Screening**<br>**Screening Visits (2 visits) Day -30 to Day -1**
Before we can do any tests, you will be asked to give written informed consent, below are details of assessments that need to be done:<br>• Questions about your health and what medication you are taking<br>• Review of your demographic (personal) information such as date of birth, ethnicity, and race<br>• Review the study requirements and restrictions<br>• Height and weight<br>• Set of vital signs (pulse, blood pressure and breathing rate) and temperature<br>• Full physical exam<br>• An ECG, which is a painless test that looks at the electrical activity of your heart by putting small sticky patches on certain areas of your body while you are lying down. These patches are connected to thin wires which are again connected to a machine which will interpret the electrical activity of your heart and then print a report.<br>• Set of routine blood and urine samples, blood samples relating to safety and PNH diagnosis<br>• Blood tests for HIV, Hepatitis B and Hepatitis C<br>• Pregnancy test (females) +/- hormone test (postmenopausal females)<br>• Vaccination if required will be done during the screening period after your eligibility has been confirmed. Your doctor will discuss with you what vaccinations you may need and will also take into account vaccinations that you have previously received.
**A final decision about whether you can take part will then be made when all your screening results are available.**

**Part 1: Site Visits (Day 1 – Day 28)**
**APL-2 dosing:**<br>• You will receive the first 3 daily doses of APL-2 (Day 1 to 3 or beyond. You will continue to attend the hospital clinic on a daily basis until you are competent and confident to use the infusion pump fo self administer the study drug at home), as well as doses on Day 8, 15 and 22 at the hospital clinic. You will need to stay in the clinic for 2.5 hours after receiving the first dose of APL-2 on Day 1 so we can make sure that you are okay.<br><br>**Other assessments done throughout the study:**<br>• You will be asked what medications you are taking and if you have had any recent illnesses or changes in your condition.<br>• Preventative Antibiotics will be taken from Day 1 until 14 days after the last dose of APL-2<br>• Preventative Antivirals will be taken from Day 1 until 14 days after the last dose of APL-2 if you are a hepatitis B carrier<br>• Full physical exam<br>• An ECG (recording of the electrical activity of your heart).<br>• Set of vital signs (pulse, blood pressure and breathing rate) and temperature. If the visit includes study drug administration, vital signs will be measured before and after you receive the study drug.<br>• Set of routine blood and urine samples, blood samples relating to safety and PNH diagnosis<br>• You will be assessed for signs/symptoms of blood clots.<br>• Pregnancy test (for females who are able to become pregnant).<br>• FACIT (Fatigue Scale) is a 13 item questionnaire which you will need to complete during clinic visits on Days 1 and 15.

- Monitor any side effects: you will be asked to notify of any unfavourable health or medical events, referred to as adverse events, experienced from the last visit.
- Vaccination (booster) on Day 57 (during Part 2A or Part 3 Follow up)

You will self administer the study drug on a daily basis (after you have been trained on the use of the infusion pump) on the following study days 4 (or from when you feel confident and are considered competent to self administer) -7, 9-14, 16-21, and 23-28

Part 2A: Site Visits (Day 29 – Day 84)

APL-2 dosing:

You will receive doses on Day 29, 36, 43, 57 and 71 at the hospital clinic. You will self administer the study drug on a daily basis on the following study days 30-35, 37-42, 44-56, 58-70 and 72-84.

Other assessments done throughout the study:

- You will be asked what medications you are taking and if you have had any recent illnesses or changes in your condition.
- Preventative Antibiotics will be taken from Day 1 until 14 days after the last dose of APL-2
- Preventative Antivirals will be taken from Day 1 until 14 days after the last dose of APL-2 if you are a hepatitis B carrier

- Full physical exam
- An ECG (recording of the electrical activity of your heart).
- Set of vital signs (pulse, blood pressure and breathing rate) and temperature. If the visit includes study drug administration, vital signs will be measured before and after you receive the study drug.
- Set of routine blood and urine samples, blood samples relating to safety and PNH diagnosis
- You will be assessed for signs/symptoms of blood clots.
- Pregnancy test (for females who are able to become pregnant).
- FACIT (Fatigue Scale) is a 13 item questionnaire which you will need to complete during clinic visits on Days 29, 43 and 71.
- Monitor any side effects: you will be asked to notify of any unfavourable health or medical events, referred to as adverse events, experienced from the last visit.
- Vaccination (booster) on Day 57

Part 2B: Site Visits (Day 85 onwards)

APL-2 dosing:

- You will receive doses of APL-2 on Day 85, 113, 141, 169, 197, 225, 253, 281, 309 and 337 at the hospital clinic. You will self administer the study drug on a daily basis on all other days in between your hospital clinic visits.

Other assessments done throughout the study:

- You will be asked what medications you are taking and if you have had any recent illnesses or changes in your condition.
- Preventative Antibiotics will be taken from Day 1 until 14 days after the last dose of APL-2
- Preventative Antivirals will be taken from Day 1 until 14 days after the last dose of APL-2 if you are a hepatitis B carrier

- Full physical exam
- An ECG (recording of the electrical activity of your heart).
- Set of vital signs (pulse, blood pressure and breathing rate) and temperature. If the visit includes study drug administration, vital signs will be measured before and after you receive the study

drug.

- Set of routine blood and urine samples, blood samples relating to safety and PNH diagnosis
- You will be assessed for signs/symptoms of blood clots.
- Pregnancy test (for females who are able to become pregnant).
- FACIT (Fatigue Scale) is a 13 item questionnaire which you will need to complete during clinic visits on Days 85,141,197, 253, 309, 365 and 414.
- Monitor any side effects: you will be asked to notify of any unfavourable health or medical events, referred to as adverse events, experienced from the last visit.

Safety Follow-up

You will be asked to return to the clinic site for follow-up visits on Days 365, 379, and 393.

After you have completed all follow-up procedures you need to return for the Exit Visit on Day 414.

You will not receive any study drug at these visits. These visits will include the following tests and procedures:

- You will be asked what medications you are taking and if you have had any illnesses or changes in your condition.
- Your vital signs (temperature, resting pulse and blood pressure, respiratory rate) will be measured.
- You will be assessed for signs/symptoms of blood clots.
- Electrocardiogram (ECG) – Day 365.
- Physical examination – Day 365 and 414.
- Collection of a urine sample for routine lab tests.
    - For women who are able to get pregnant, urine will be collected at Day 365, 393 and 414 for a pregnancy test.
- Collection of blood.
- You will be asked to complete a FACIT (Fatigue Scale) questionnaire at Day 365, 393 and 414.

3. WHAT WILL HAPPEN TO MY TEST SAMPLES

The following blood samples will be collected throughout the study:

- Samples to determine regular routine blood counts and blood chemistry, to monitor your general health. (Haematology and Chemistry)
- Blood tests for HIV, Hepatitis B and Hepatitis C (Serology)
- Blood tests to determine how quickly your blood clots (Coagulation Profile)
- Pregnancy Test (If applicable)

All these routine samples will be sent to a Central lab overseas for testing and destroyed at the end of the study by internationally accepted means e.g. incineration.

The following blood samples will also be collected throughout the study. These samples will be sent to different locations for testing.

- Blood samples which determine the level of study drug in your blood (Pharmacokinetics).
- Blood samples to determine the effects of study drug on your body (Pharmacodynamics).
- Blood samples for immunogenicity (The ability to cause the production of antibodies to the study drug).
- Blood samples for cytometry testing (This test is able to identify the type/amount of cells in different parts of the cell cycle).

These blood samples may be stored for up to 1 year after the end of treatment after which time they will be destroyed by internationally accepted means e.g. incineration.

The maximum amount of blood collected from each participant during the study will be approximately 580 mL (Approximately 39 tablespoons).

All samples will be coded; identified only by each participant's study number and initials. Only hospital and laboratory staff will have access to the blood samples.

4. SAFETY: POTENTIAL RISKS AND DISCOMFORTS

a. Are there any risks from your participation in this study?

There may be risks to taking part in this study from APL-2 or from some of the procedures or tests done in this study.

If you decide to take part in this study, you or your family members should tell the study doctor or <<Insert Site Name>> staff immediately if you have any unusual health problems, injuries, or side effects, even if you do not think these problems are caused by the study or APL-2.

If there is any new important safety information or other information that could affect your willingness to participate in this study, your study doctor will let you know.

b. What are the likely risks with APL-2?

You may feel discomfort during some of the tests and study procedures. Possible risks, side effects, and discomforts include:

- Infections – you may be at increased risk for infections caused by certain types of bacteria such as Streptococcus pneumonia, Neisseria Meningitidis, or Haemophilus influenza.
- Allergic reactions – all drugs have a potential risk of an allergic reaction, which if not treated promptly, could become life-threatening.
- Blood samples - you may have discomfort or pain when your blood is collected. You may feel faint or pass out. There is also a slight possibility of infection, bruising, or bleeding at the puncture site. Your anaemia (low red blood cell count) could worsen from having frequent blood draws
- ECG - skin irritation is rare but could occur during an ECG from the sticky patches or gel that is used
- Antibiotic and antiviral therapy – there is a possibility that you will experience some side effects from the antibiotic treatment you will need to take during this study. Side effects such as nausea, sickness, diarrhoea, dizziness, thrush, and skin rashes may occur and if you experience any of these you should inform the nurse or doctor. The doctor may decide that you should change to a different antibiotic to try and reduce these symptoms.
- Injection site reactions - there is a possibility that you could experience redness, swelling, and pain or tenderness at the site of injection. There is also a slight possibility of infection.

c. Any unforeseen risks?

Since the study drug APL-2 is investigational when taken alone or in combination with other medications, there may be other risks that are unknown.

d. Could APL-2 be harmful to an unborn or breastfed baby?

You should discuss pregnancy risks with your doctor before signing this consent form. Women who are pregnant, planning to become pregnant or breast feeding may not participate in this research study. Taking the study drug may involve unknown risks to a pregnant woman, an embryo, fetus (unborn baby) or nursing infant. If you (or your partner) become pregnant while in this study, the sponsor may ask to follow the outcome of the pregnancy. The study drug will be stopped and your participation in this study will be ended.

Before taking part in this research study and during the study, females able to have children will have pregnancy tests done. Talk to your doctor about the best method of birth control to use while you are in this study. It is important that you call your study doctor at <<***insert phone number***>> right away if you become pregnant or father a child during the course of this study.

5. BENEFITS OF PARTICIPATION

Are there any benefits to your participation in this study?

You may or may not benefit from participation in this study. There is no guarantee that you will continue to benefit from participating in this study. The information from this study might help to develop better treatments in the future for people with PNH. An indirect benefit of taking part in this study is that you will receive free medical tests at screening and during the study.

What are the alternatives to taking part? Do you have any other choices?

You do not have to be in this study to receive treatment for your PNH. You have the option to receive standard treatment for PNH. Standard treatment may include medications such as prednisone, or blood thinners; blood transfusions; or bone marrow transplantation. The study doctor will discuss with you the risks and benefits of the alternative treatments.

6. POTENTIAL COSTS/ REIMBURSEMENTS

There will be no charge to you for your participation in this study. The study drug, study-related procedures, and study visits will be provided at no charge to you.

Will you be paid for your samples (such as blood or urine) or medical information?

You will not be paid for the use of your samples or any information, discoveries or inventions obtained from your samples or medical information.

You will be reimbursed for any reasonable travel, accommodation, parking, meals and other expenses associated with the research project visit, this will based on the distance that you have to travel to the hospital.

7. WHAT ARE THE RIGHTS OF PARTICIPANTS IN THIS STUDY?

a. Participation is voluntary

Participation in any research project is voluntary. If you do not wish to take part, you do not have to. If you decide to take part and later change your mind, you are free to withdraw from the project at any stage. This will not affect your routine treatment, your relationship with those treating you or with <<Insert Site Name>> .

If you do decide to take part, you will be given this Participant Information and Consent Form to sign and you will be given a copy to keep.

b. New information

Sometimes during the course of a research project, new information becomes available about the treatment that is being studied. If this happens, your study doctor will tell you about it and discuss with you whether you want to continue in the research project. If you decide to withdraw, your study doctor will make arrangements for your regular health care to continue. If you decide to continue in the research project you may be asked to sign an updated consent form.

Also, on receiving new information, your study doctor might consider it to be in your best interests to withdraw you from the research project. If this happens, he/ she will explain the reasons and arrange for your regular health care to continue.

c. Right to access information collected during the study

In accordance with relevant New Zealand privacy and other relevant laws, you have the right to request access to your information collected and stored by the research team. You also have the right to request that any information with which you disagree be corrected. Please contact the study team member named at the end of this document if you would like to access your information.

8. CONFIDENTIALITY

By signing the consent form you consent to the study doctor and relevant research staff collecting and using personal information about you for the research project. Any information obtained in connection with this research project that can identify you will remain confidential. Your name, address or phone number will not be on the study forms; instead you will be identified by a unique participant study number, date of birth and initials. Your information will only be used for the purpose of this research project and it will only be disclosed with your permission, except as required by law.

Information about you may be obtained from your health records held at this and other health services for the purpose of this research. By signing the consent form you agree to the study team accessing health records if they are relevant to your participation in this research project.

Your health records and any information obtained during the research project are subject to inspection (for the purpose of verifying the procedures and the data) by the relevant authorities (such as the Ethics Committee that has reviewed and approved this study) and authorised representatives of the Sponsor, Apellis, and <<Insert Site Name>> or as required by law. All personnel reviewing your medical records will be required to keep the information confidential in accordance with law. By signing the Consent Form, you authorise release of, or access to, this confidential information to the relevant study personnel and regulatory authorities as noted above.

When the research project ends the study data must be analysed, so the results of the study may not be available until about six months after the research finishes. The study doctors and/or Apellis may decide to discuss or publish the results of the study. This may include publication in journals, presentation at conferences or other professional forums. In any publication, information will be provided in such a way that you cannot be identified. Results of the study will be provided to you, if you wish.

Information about your participation in this research project may be recorded in your health records.

Any information obtained for the purpose of this research project that can identify you will be treated as confidential and securely stored. It will be disclosed only with your permission, or as required by law.

Upon completion of the trial, the study file containing information about you will be archived for a minimum of fifteen years.

9. COMPENSATION FOR INJURY

If you were injured as a result of treatment given as part of this study, compensation would be available from the study's sponsor, Apellis, in line with industry guidelines. We can give you a copy of these guidelines if you wish.

If you have private health or life insurance, you may wish to check with your insurer that taking part in this study won't affect your cover.

10. WHAT WILL HAPPEN AFTER THE STUDY ENDS, OR IF YOU PULL OUT?

If you decide to withdraw
Please notify a member of the research team before you withdraw. This notice will allow that person or the research supervisor to discuss any health risks or special requirements linked to withdrawing, such as follow up visits.

If you do withdraw your consent during the research project, information already collected will be retained to ensure that the results of the research project can be measured properly and to comply with law. You should be aware that data collected by the sponsor up to the time you withdraw will form part of the research project results. If you do not want them to do this, you must tell them before you join the research project.

Why the study might be unexpectedly stopped
This research project may be stopped unexpectedly for a variety of reasons. These may include reasons such as:
• Unacceptable side effects
• The drug being shown not to be effective
• The drug being shown to work and not need further testing.

Will the study treatment continue to be available after the study finished?

If it is found the drug does continue to have a beneficial effect in reducing haemolysis (breakdown of red blood cells), and no safety concerns are raised, the company may, at its sole discretion, elect to continue the drug therapy by offering a further extension to the study.

Where can you go for more information about the study, or to raise concerns or complaints?

If you have any questions, concerns or complaints about the study at any stage, you can contact:

<<Insert Name>>, lead study doctor
Phone: <<Insert Contact Number>>and ask operator to page or put you through to <<Insert Name>>
Research Coordinator: <<Insert Contact Number>>

If you want to talk to someone who isn't involved with the study, you can contact an independent health and disability advocate. Specialist advocates, are available here.

Phone:
Fax:
Email:

You can also contact the health and disability ethics committee that approved this study on:

Phone:
Email:

12. DO I HAVE TO DECIDE STRAIGHT AWAY?

No, you do not have to decide straight away. You should take some time to consider whether or not to continue your participation in the study. Study staff will let you know when they will need your decision. The following steps are useful in helping you reach a decision.

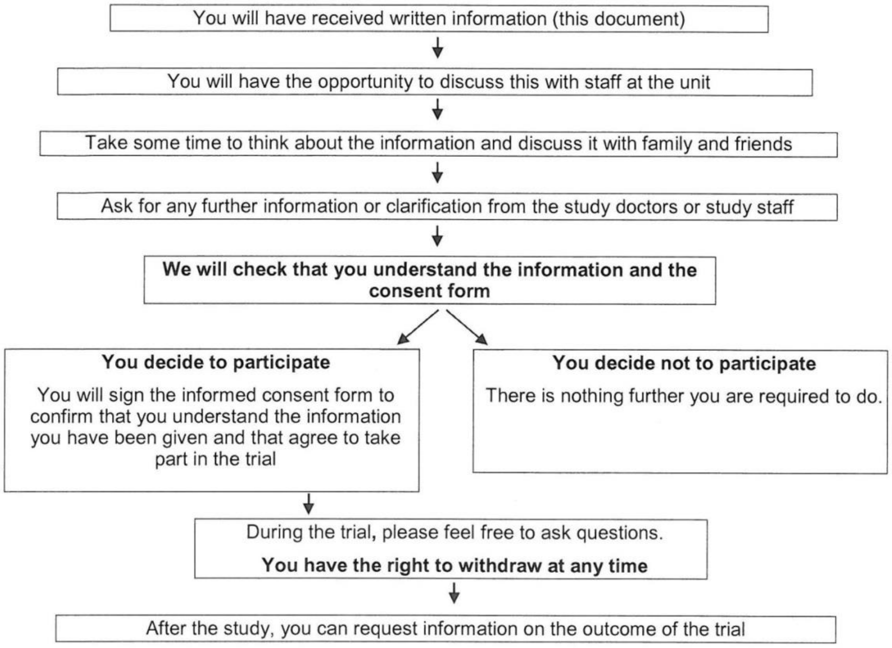
You will have received written information (this document)
You will have the opportunity to discuss this with staff at the unit
Take some time to think about the information and discuss it with family and friends
Ask for any further information or clarification from the study doctors or study staff
We will check that you understand the information and the consent form
You decide to participate
You will sign the informed consent form to confirm that you understand the information you have been given and that agree to take part in the trial
You decide not to participate
There is nothing further you are required to do.
During the trial, please feel free to ask questions.
You have the right to withdraw at any time
After the study, you can request information on the outcome of the trial

Insert Header with institution's name or institution's letterhead

INFORMED CONSENT FORM

**Short Title**	Nocturnal Hemoglobinuria (PNH) – PADDOCK
**Protocol Number**	APL2-CP-PNH-204
**Principal Investigator**	<<Insert Name>>

Declaration by participant:

I have read and I understand this version of the Participant Information Sheet. I have had the opportunity to ask questions and I am satisfied with the answers I have received.

I have had the opportunity to discuss the study with my family.

I freely agree to continue to participate in this study and understand that I may withdraw from the study at any time.

I understand that:

- My participation in this study is confidential and I will not be identified in study reports.
- My medical records may be reviewed by Apellis, companies working with them, auditors and health or regulatory agencies for the purpose of checking the accuracy of the information recorded for the study
- Some of my samples will be sent overseas for analysis and they will be destroyed as described in the Information Sheet.
- If I decide to withdraw from the study, information already collected may continue to be processed. I may be asked to attend follow-up visits.
- The study drugs or tests will be stopped if they appear harmful to me.
- I should contact site staff if I have any side effects.
- My GP and/or specialist will be informed of my participation in this study and relevant medical records may be accessed.
- I am not giving up any of my legal rights by signing this consent form.
- This trial is being carried out for the principal benefit of Apellis. If I suffer an injury as a result of my participation in the trial, they will pay me compensation in line with industry guidelines.
- I understand that there may be risks associated with the treatment in the event of myself or my partner becoming pregnant. I undertake to inform my partner of the risks and to take responsibility for the prevention of pregnancy

**Statement by Participant**	I hereby consent to take part in this study. I understand that I will receive a signed copy of this consent form for my records.<br>____________________________ (full name)<br>____________________________ (signature)<br>__ __ / __ __ __ / __ __ (Date)

Statement by Consenter (Investigator/designee) I have discussed this study with the above named participant. The participant appeared to fully understand the information provided about the study.

____________________________ (full name)

____________________________ (signature)

__ __ / __ __ __ / __ __ (Date)

Insert Header with institution's name or institution's letterhead

PARTICIPANT INFORMATION SHEET

[Insert site name]

**Short Title**	A Phase Ib, Open Label, Multiple Ascending Dose, Pilot Study to Assess the Safety, Preliminary Efficacy and Pharmacokinetics of Subcutaneously Administered APL-2 in Subjects with Paroxysmal Nocturnal Hemoglobinuria (PNH). – PADDOCK		
**Protocol Number**	APL2-CP-PNH-204	**Ethics Number**	<<Insert #>>
**Project Sponsor**	Apellis Pharmaceuticals, Inc.<br>6400 Westwind Way, Suite A, Louisville, KY, 40014		
**Local Sponsor**	Novotech		
**Principal Investigator**	<<Insert Name>>		
**Location**	<<Insert Location>>		
**Study related phone number**	<<Insert Contact Number>>		

You are being invited to take part in the second cohort of a research study of the trial drug APL-2. This consent form gives you important information about this study to help you decide if you want to take part in the research study. It describes the purpose of this study, the study procedures, and the possible risks, and provides information about your rights as a study participant.

Please take the time to read this information carefully. You should talk to your study doctor and study staff about this study and ask any questions you have. You can also discuss this study with other people such as your family or general practitioner (GP). If you decide to take part in this study, you will be asked to sign and date this form. You will receive a copy of the signed form. Your study doctor will tell your GP about your participation in this study.

Who is funding this study?

Apellis Pharmaceuticals, Inc. ("Apellis") is funding this clinical study. Apellis is the "sponsor" of the study, which means that Apellis takes responsibility for starting, managing, and financing the study. <<Insert Site Name>> is being paid by Apellis to do this study.

Voluntary participation and withdrawal from this study

Your participation in this study is voluntary. You are free to say yes or no. Even if you join this study, you may stop at any time.

A description of this clinical trial will be available on the internet at http://www.ClinicalTrials.gov. This website will not include information that can identify you. At most, the website will include a summary of the results. You can search this website at any time.

1. WHAT YOU SHOULD KNOW ABOUT THIS STUDY

a. Why is this study being done?

This is a research study to test a new investigational drug. An investigational drug is one that is not approved by any regulatory authority (e.g. FDA, Department of Health, Medsafe etc) for use outside a clinical trial. Cohort 1 has completed and 2 patients have already completed in excess of 169 days of dosing in Cohort 2. The study was initially set up in New Zealand but insufficient eligible PNH patients exist to complete the study.

You are being invited to participate in this research study because you have been diagnosed with paroxysmal nocturnal hemoglobinuria (PNH). PNH is a rare disorder that causes the red blood cells to break down too early**.** Many PNH patients suffer from complications such as blood clots or low red blood cell counts (anemia) even after receiving standard treatment.

The research study will continue to gather scientific information about the safety, tolerability and continued evidence of activity of the investigational drug APL-2 in people with PNH who have not been treated with Soliris® in the past. The study will help determine how APL-2 behaves inside the body, how it is eliminated from the body, the effect it has on the body after multiple doses and if it can prevent red blood cells from breaking down.

b. How does APL-2 works?

Your body has numerous biological mechanisms designed to attack and kill off invaders, such as bacteria. One of these mechanisms, called the complement cascade, is part of your normal immune system. Normally, only invaders are destroyed by the complement cascade as your own cells are protected against its attacks. However, patients with PNH possess red blood cells that are defective and are not properly protected, which results in the destruction of these defective red blood cells by your own immune system. The breakdown of a large number of red blood cells in this manner has been shown to trigger episodes of PNH. Based on the research conducted prior to this Phase Ib study, the investigational study drug, which is a peptide (small protein), is expected to decrease the activity of the complement cascade and prevent the destruction of defective red blood cells. Results from the ongoing/completed studies also provided good assurance of the safety for the doses of APL-2 in this study.

c. Dosing of APL-2

APL-2 will be given daily, as a subcutaneous (the fat tissue under your skin) infusion using an infusion pump that you will be trained to use and self administer when fully trained and competent in the use of the pump. Until that time, you will attend the clinical site to receive your study medication.

You will receive 270 mg APL-2 each day for up to 364 days, although the study doctor may increase your dose up to 360 mg APL-2 each day if a higher dose is needed to reduce breakdown of your red blood cells

The safety monitoring committee will review all the safety data available from participants already dosed in the study approximately every 8 weeks.

The study can be stopped at any time, based on evaluation of the side effects of the study medication.

2. WHAT WILL HAPPEN IF YOU TAKE PART IN THIS STUDY

a. What will you be responsible for if you take part in this study?

If you decide to take part in this study you will be asked to sign this ethics committee-approved informed consent form and you must meet specific entry rules.

You must also agree to the following:

- Be willing and able to attend all visits at the study center.
- Immunisation is required to participate in the study. Vaccinations may be given during the study visit on Day 15 if you have not received the required immunisation within 2 years of the Day 1 visit screening visit. There immunisations will be free as they are required as part of the trial. Additional booster vaccinations will be given on Day 85, if required.
- Be willing to allow frequent blood tests during the study and at subsequent follow up visits.
- You will be requested to keep the study drug supplies in your refrigerator (between 2-8°C) If there is a power failure, you should contact the clinic site.
- Follow the instructions given by the study doctor and study staff.
- Take an antibiotic throughout the study to prevent bacterial infections.
- Take an antiviral throughout the study if you are a hepatitis B carrier, to prevent reactivation of the hepatitis B virus.
- Have all procedures performed.
- Males must agree to no sperm donation at any time during the study.
- Females who are able to have children must agree to use an effective contraception for the duration of the study. Your study doctor can advise you on effective methods of contraception.
- Make sure you take only medications approved by a study doctor. Tell the study doctor what medications you are currently taking or have taken in the past. Also, tell him / her before starting any new medications and/or if there are changes to your current medications.

b. What types of tests or procedures will be involved with continuation in this study?

You will receive / administer daily subcutaneous infusions which will be done by the study staff when you visit the clinic site or by you after you have been fully trained to self administer using the infusion pump supplied for sole use in this study.

<<Insert Site Name>> will contact your regular doctor (GP) about your study participation. If you do not want your GP told, you will not be able to continue to take part in the study. We may need to contact your GP about any health issues or parts of your medical history that might affect your participation in the study.

You will be given a Participant Identification Card when you start this study, stating the name of the study and the study doctor's contact information. **This card should be carried with you at all times** so that you can contact the study doctor at any time and so you can show it to any other doctor or dentist that you might visit while in this study.

c. What are the study details? What procedures will you have?

Your total time in this study is expected to be approximately 414 days. If more tests are needed, your study doctor may ask you to schedule additional visits which could extend your total time in the study. Your study doctor or Apellis may choose to withdraw you from this research study at any time which could decrease your total time in the study.

The study has 4 parts;

- Part 1: You will receive APL-2 for 28 days and you will visit the study centre at least 8 times.
- Part 2A: If there is evidence of clinical benefit to you by Day 28; you will continue to receive APL-2 up to Day 84. You will visit the study centre 5 more times.
- Part 2B: If there is still evidence of clinical benefit to you by Day 84, you will continue to receive daily APL-2 treatment up to Day 364. Part 2B will include at least 10 visits to the study center. If there is evidence that you are no longer getting clinical benefit, treatment will be stopped and you will skip Part 2B and enter Part 3 for safety follow-up visits
- Part 3: Safety follow-up will include 4 visits to the study center.

You will be monitored throughout the study for any changes in health. Throughout the entire duration of the study, the <<Insert Site Name>> staff will continue to review any other medications you may be taking and any potential side effects to ensure your safety.

The following activities will be performed during site visits:

**Screening**<br>**Screening Visits (2 visits) Day -30 to Day -1**
Before we can do any tests, you will be asked to give written informed consent, below are details of assessments that need to be done:<br>• Questions about your health and what medication you are taking<br>• Review of your demographic (personal) information such as date of birth, ethnicity, and race<br>• Review the study requirements and restrictions<br>• Height and weight<br>• Set of vital signs (pulse, blood pressure and breathing rate) and temperature<br>• Full physical exam<br>• An ECG, which is a painless test that looks at the electrical activity of your heart by putting small sticky patches on certain areas of your body while you are lying down. These patches are connected to thin wires which are again connected to a machine which will interpret the electrical activity of your heart and then print a report.<br>• Set of routine blood and urine samples, blood samples relating to safety and PNH diagnosis<br>• Blood tests for HIV, Hepatitis B and Hepatitis C<br>• Pregnancy test (females) +/- hormone test (postmenopausal females)<br>• Your doctor will discuss with you what vaccinations you may need and will also take into account vaccinations that you have previously received.
**A final decision about whether you can take part will then be made when all your screening results are available.**

**Part 1: Site Visits (Day 1 – Day 28)**
**APL-2 dosing:**<br>• You will receive the first 3 daily doses of APL-2 (Day 1 to 3 or beyond. You will continue to attend the hospital clinic on a daily basis until you are competent and confident to use the infusion pump for self administer the study drug at home), as well as doses on Day 8, 15 and 22 at the hospital clinic. You will need to stay in the clinic for 2.5 hours after receiving the first dose of APL-2 on Day 1 so we can make sure that you are okay.<br><br>**Other assessments done throughout the study:**<br>• You will be asked what medications you are taking and if you have had any recent illnesses or changes in your condition.<br>• Preventative Antibiotics will be taken from Day 1 until 14 days after the last dose of APL-2. The antibiotic will be changed on Day 15 once vaccinations have been received.<br>• Preventative Antivirals will be taken from Day 1 until 14 days after the last dose of APL-2 if you are a hepatitis B carrier<br>• Vaccinations, if required, will be given at the clinic visit on Day 15<br>• Full physical exam<br>• An ECG (recording of the electrical activity of your heart).<br>• Set of vital signs (pulse, blood pressure and breathing rate) and temperature. If the visit includes study drug administration, vital signs will be measured before and after you receive the study drug.<br>• Set of routine blood and urine samples, blood samples relating to safety and PNH diagnosis<br>• You will be assessed for signs/symptoms of blood clots.<br>• Pregnancy test (for females who are able to become pregnant).

- FACIT (Fatigue Scale) is a 13 item questionnaire which you will need to complete during clinic visits on Days 1 and 15.
- Monitor any side effects: you will be asked to notify of any unfavourable health or medical events, referred to as adverse events, experienced from the last visit.
- Vaccination (booster) on Day 57 (during Part 2A or Part 3 Follow up)

You will self administer the study drug on a daily basis (after you have been trained on the use of the infusion pump) on the following study days 4 (or from when you feel confident and are considered competent to self administer) -7, 9-14, 16-21, and 23-28

Part 2A: Site Visits (Day 29 – Day 84)

APL-2 dosing:

You will receive doses on Day 29, 36, 43, 57 and 71 at the hospital clinic. You will self administer the study drug on a daily basis on the following study days 30-35, 37-42, 44-56, 58-70 and 72-84.

Other assessments done throughout the study:

- You will be asked what medications you are taking and if you have had any recent illnesses or changes in your condition.
- Preventative Antibiotics will be taken from Day 1 until 14 days after the last dose of APL-2
- Preventative Antivirals will be taken from Day 1 until 14 days after the last dose of APL-2 if you are a hepatitis B carrier
- Full physical exam
- An ECG (recording of the electrical activity of your heart).
- Set of vital signs (pulse, blood pressure and breathing rate) and temperature. If the visit includes study drug administration, vital signs will be measured before and after you receive the study drug.
- Set of routine blood and urine samples, blood samples relating to safety and PNH diagnosis
- You will be assessed for signs/symptoms of blood clots.
- Pregnancy test (for females who are able to become pregnant).
- FACIT (Fatigue Scale) is a 13 item questionnaire which you will need to complete during clinic visits on Days 29, 43 and 71.
- Monitor any side effects: you will be asked to notify of any unfavourable health or medical events, referred to as adverse events, experienced from the last visit.
-

Part 2B: Site Visits (Day 85 onwards)

APL-2 dosing:

- You will receive doses of APL-2 on Day 85, 113, 141, 169, 197, 225, 253, 281, 309 and 337 at the hospital clinic. You will self administer the study drug on a daily basis on all other days in between your hospital clinic visits.

Other assessments done throughout the study:

- You will be asked what medications you are taking and if you have had any recent illnesses or changes in your condition.
- Preventative Antibiotics will be taken from Day 1 until 14 days after the last dose of APL-2
- Preventative Antivirals will be taken from Day 1 until 14 days after the last dose of APL-2 if you are a hepatitis B carrier
- Booster vaccinations, if required, will be given at the Day 85 clinic visit

- Full physical exam
- An ECG (recording of the electrical activity of your heart).
- Set of vital signs (pulse, blood pressure and breathing rate) and temperature. If the visit includes study drug administration, vital signs will be measured before and after you receive the study drug.
- Set of routine blood and urine samples, blood samples relating to safety and PNH diagnosis
- You will be assessed for signs/symptoms of blood clots.
- Pregnancy test (for females who are able to become pregnant).
- FACIT (Fatigue Scale) is a 13 item questionnaire which you will need to complete during clinic visits on Days 85,141,197, 253, 309, 365 and 414.
- Monitor any side effects: you will be asked to notify of any unfavourable health or medical events, referred to as adverse events, experienced from the last visit.

Safety Follow-up

You will be asked to return to the clinic site for follow-up visits on Days 365, 379, and 393.

After you have completed all follow-up procedures you need to return for the Exit Visit on Day 414.

You will not receive any study drug at these visits. These visits will include the following tests and procedures:

- You will be asked what medications you are taking and if you have had any illnesses or changes in your condition.
- Your vital signs (temperature, resting pulse and blood pressure, respiratory rate) will be measured.
- You will be assessed for signs/symptoms of blood clots.
- Electrocardiogram (ECG) – Day 365.
- Physical examination – Day 365 and 414.
- Collection of a urine sample for routine lab tests.
  - For women who are able to get pregnant, urine will be collected at Day 365, 393 and 414 for a pregnancy test.
- Collection of blood.
- You will be asked to complete a FACIT (Fatigue Scale) questionnaire at Day 365, 393 and 414.

3. WHAT WILL HAPPEN TO MY TEST SAMPLES

The following blood samples will be collected throughout the study:

- Samples to determine regular routine blood counts and blood chemistry, to monitor your general health. (Haematology and Chemistry)
- Blood tests for HIV, Hepatitis B and Hepatitis C (Serology)
- Blood tests to determine how quickly your blood clots (Coagulation Profile)
- Pregnancy Test (If applicable)

All these routine samples will be sent to a Central lab overseas for testing and destroyed at the end of the study by internationally accepted means e.g. incineration.

The following blood samples will also be collected throughout the study. These samples will be sent to different locations for testing.

- Blood samples which determine the level of study drug in your blood (Pharmacokinetics).
- Blood samples to determine the effects of study drug on your body (Pharmacodynamics).
- Blood samples for immunogenicity (The ability to cause the production of antibodies to the study drug).

- Blood samples for cytometry testing (This test is able to identify the type/amount of cells in different parts of the cell cycle).

These blood samples may be stored for up to 1 year after the end of treatment after which time they will be destroyed by internationally accepted means e.g. incineration.

The maximum amount of blood collected from each participant during the study will be approximately 580 mL (Approximately 39 tablespoons).

All samples will be coded; identified only by each participant's study number and initials. Only hospital and laboratory staff will have access to the blood samples.

4. SAFETY: POTENTIAL RISKS AND DISCOMFORTS

a. Are there any risks from your participation in this study?

There may be risks to taking part in this study from APL-2 or from some of the procedures or tests done in this study.

If you decide to take part in this study, you or your family members should tell the study doctor or <<Insert Site Name>> staff immediately if you have any unusual health problems, injuries, or side effects, even if you do not think these problems are caused by the study or APL-2.

If there is any new important safety information or other information that could affect your willingness to participate in this study, your study doctor will let you know.

b. What are the likely risks with APL-2?

You may feel discomfort during some of the tests and study procedures. Possible risks, side effects, and discomforts include:

- Infections – you may be at increased risk for infections caused by certain types of bacteria such as Streptococcus pneumonia, Neisseria Meningitidis, or Haemophilus influenza.
- Allergic reactions – all drugs have a potential risk of an allergic reaction, which if not treated promptly, could become life-threatening.
- Blood samples - you may have discomfort or pain when your blood is collected. You may feel faint or pass out. There is also a slight possibility of infection, bruising, or bleeding at the puncture site. Your anaemia (low red blood cell count) could worsen from having frequent blood draws
- ECG - skin irritation is rare but could occur during an ECG from the sticky patches or gel that is used
- Antibiotic and antiviral therapy – there is a possibility that you will experience some side effects from the antibiotic treatment you will need to take during this study. Side effects such as nausea, sickness, diarrhoea, dizziness, thrush, and skin rashes may occur and if you experience any of these you should inform the nurse or doctor. The doctor may decide that you should change to a different antibiotic to try and reduce these symptoms.
- Injection site reactions - there is a possibility that you could experience redness, swelling, and pain or tenderness at the site of injection. There is also a slight possibility of infection.

c. Any unforeseen risks?

Since the study drug APL-2 is investigational when taken alone or in combination with other

medications, there may be other risks that are unknown.

d. Could APL-2 be harmful to an unborn or breastfed baby?

You should discuss pregnancy risks with your doctor before signing this consent form. Women who are pregnant, planning to become pregnant or breast feeding may not participate in this research study. Taking the study drug may involve unknown risks to a pregnant woman, an embryo, fetus (unborn baby) or nursing infant. If you (or your partner) become pregnant while in this study, the sponsor may ask to follow the outcome of the pregnancy. The study drug will be stopped and your participation in this study will be ended.

Before taking part in this research study and during the study, females able to have children will have pregnancy tests done. Talk to your doctor about the best method of birth control to use while you are in this study. It is important that you call your study doctor at <<***insert phone number***>> right away if you become pregnant or father a child during the course of this study.

5. BENEFITS OF PARTICIPATION

Are there any benefits to your participation in this study?

You may or may not benefit from participation in this study. There is no guarantee that you will continue to benefit from participating in this study. The information from this study might help to develop better treatments in the future for people with PNH. An indirect benefit of taking part in this study is that you will receive free medical tests at screening and during the study.

What are the alternatives to taking part? Do you have any other choices?

You do not have to be in this study to receive treatment for your PNH. You have the option to receive standard treatment for PNH. Standard treatment may include medications such as prednisone, or blood thinners; blood transfusions; or bone marrow transplantation. The study doctor will discuss with you the risks and benefits of the alternative treatments.

6. POTENTIAL COSTS/ REIMBURSEMENTS

There will be no charge to you for your participation in this study. The study drug, study-related procedures, and study visits will be provided at no charge to you.

Will you be paid for your samples (such as blood or urine) or medical information?

You will not be paid for the use of your samples or any information, discoveries or inventions obtained from your samples or medical information.

You will be reimbursed for any reasonable travel, accommodation, parking, meals and other expenses associated with the research project visit, this will based on the distance that you have to travel to the hospital.

7. WHAT ARE THE RIGHTS OF PARTICIPANTS IN THIS STUDY?

a. Participation is voluntary

Participation in any research project is voluntary. If you do not wish to take part, you do not have to. If you decide to take part and later change your mind, you are free to withdraw from the project at any stage. This will not affect your routine treatment, your relationship with those treating you or with <<Insert Site Name>> .

If you do decide to take part, you will be given this Participant Information and Consent Form to sign and you will be given a copy to keep.

b. New information

Sometimes during the course of a research project, new information becomes available about the treatment that is being studied. If this happens, your study doctor will tell you about it and discuss with you whether you want to continue in the research project. If you decide to withdraw, your study

doctor will make arrangements for your regular health care to continue. If you decide to continue in the research project you may be asked to sign an updated consent form.

Also, on receiving new information, your study doctor might consider it to be in your best interests to withdraw you from the research project. If this happens, he/ she will explain the reasons and arrange for your regular health care to continue.

c. Right to access information collected during the study

In accordance with relevant New Zealand privacy and other relevant laws, you have the right to request access to your information collected and stored by the research team. You also have the right to request that any information with which you disagree be corrected. Please contact the study team member named at the end of this document if you would like to access your information.

8. CONFIDENTIALITY

By signing the consent form you consent to the study doctor and relevant research staff collecting and using personal information about you for the research project. Any information obtained in connection with this research project that can identify you will remain confidential. Your name, address or phone number will not be on the study forms; instead you will be identified by a unique participant study number, date of birth and initials. Your information will only be used for the purpose of this research project and it will only be disclosed with your permission, except as required by law.

Information about you may be obtained from your health records held at this and other health services for the purpose of this research. By signing the consent form you agree to the study team accessing health records if they are relevant to your participation in this research project.

Your health records and any information obtained during the research project are subject to inspection (for the purpose of verifying the procedures and the data) by the relevant authorities (such as the Ethics Committee that has reviewed and approved this study) and authorised representatives of the Sponsor, Apellis, and <<Insert Site Name>> or as required by law. All personnel reviewing your medical records will be required to keep the information confidential in accordance with law. By signing the Consent Form, you authorise release of, or access to, this confidential information to the relevant study personnel and regulatory authorities as noted above.

When the research project ends the study data must be analysed, so the results of the study may not be available until about six months after the research finishes. The study doctors and/or Apellis may decide to discuss or publish the results of the study. This may include publication in journals, presentation at conferences or other professional forums. In any publication, information will be provided in such a way that you cannot be identified. Results of the study will be provided to you, if you wish.

Information about your participation in this research project may be recorded in your health records.

Any information obtained for the purpose of this research project that can identify you will be treated as confidential and securely stored. It will be disclosed only with your permission, or as required by law.

Upon completion of the trial, the study file containing information about you will be archived for a minimum of fifteen years.

9. COMPENSATION FOR INJURY

If you were injured as a result of treatment given as part of this study, compensation would be available from the study's sponsor, Apellis, in line with industry guidelines. We can give you a copy of these guidelines if you wish.

If you have private health or life insurance, you may wish to check with your insurer that taking part in this study won't affect your cover.

10. WHAT WILL HAPPEN AFTER THE STUDY ENDS, OR IF YOU PULL OUT?

If you decide to withdraw
Please notify a member of the research team before you withdraw. This notice will allow that person or the research supervisor to discuss any health risks or special requirements linked to withdrawing, such as follow up visits.

If you do withdraw your consent during the research project, information already collected will be retained to ensure that the results of the research project can be measured properly and to comply with law. You should be aware that data collected by the sponsor up to the time you withdraw will form part of the research project results. If you do not want them to do this, you must tell them before you join the research project.

Why the study might be unexpectedly stopped
This research project may be stopped unexpectedly for a variety of reasons. These may include reasons such as:
- Unacceptable side effects
- The drug being shown not to be effective
- The drug being shown to work and not need further testing.

Will the study treatment continue to be available after the study finished?

If it is found the drug does continue to have a beneficial effect in reducing haemolysis (breakdown of red blood cells), and no safety concerns are raised, the company may, at its sole discretion, elect to continue the drug therapy by offering a further extension to the study.

Where can you go for more information about the study, or to raise concerns or complaints?

If you have any questions, concerns or complaints about the study at any stage, you can contact:

<<Insert Name>>, lead study doctor
Phone: <<Insert Contact Number>>and ask operator to page or put you through to <<Insert Name>>
Research Coordinator: <<Insert Contact Number>>

If you want to talk to someone who isn't involved with the study, you can contact an independent health and disability advocate. Specialist advocates, are available here.

Phone:
Fax:
Email:

You can also contact the health and disability ethics committee that approved this study on:

Phone:
Email:

12. DO I HAVE TO DECIDE STRAIGHT AWAY?

No, you do not have to decide straight away. You should take some time to consider whether or not to continue your participation in the study. Study staff will let you know when they will need your decision. The following steps are useful in helping you reach a decision.

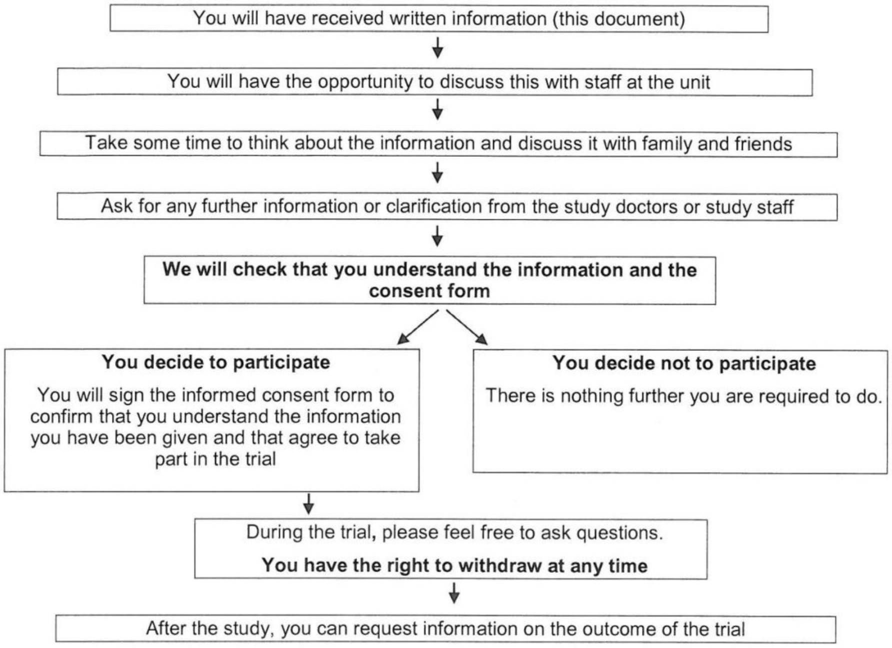
You will have received written information (this document)
You will have the opportunity to discuss this with staff at the unit
Take some time to think about the information and discuss it with family and friends
Ask for any further information or clarification from the study doctors or study staff
We will check that you understand the information and the consent form
You decide to participate
You will sign the informed consent form to confirm that you understand the information you have been given and that agree to take part in the trial
You decide not to participate
There is nothing further you are required to do.
During the trial, please feel free to ask questions.
You have the right to withdraw at any time
After the study, you can request information on the outcome of the trial

Insert Header with institution's name or institution's letterhead

INFORMED CONSENT FORM

**Short Title**	Nocturnal Hemoglobinuria (PNH) – PADDOCK
**Protocol Number**	APL2-CP-PNH-204
**Principal Investigator**	<<Insert Name>>

Declaration by participant:
I have read and I understand this version of the Participant Information Sheet. I have had the opportunity to ask questions and I am satisfied with the answers I have received.

I have had the opportunity to discuss the study with my family.

I freely agree to continue to participate in this study and understand that I may withdraw from the study at any time.

I understand that:

- My participation in this study is confidential and I will not be identified in study reports.
- My medical records may be reviewed by Apellis, companies working with them, auditors and health or regulatory agencies for the purpose of checking the accuracy of the information recorded for the study
- Some of my samples will be sent overseas for analysis and they will be destroyed as described in the Information Sheet.
- If I decide to withdraw from the study, information already collected may continue to be processed. I may be asked to attend follow-up visits.
- The study drugs or tests will be stopped if they appear harmful to me.
- I should contact site staff if I have any side effects.
- My GP and/or specialist will be informed of my participation in this study and relevant medical records may be accessed.
- I am not giving up any of my legal rights by signing this consent form.
- This trial is being carried out for the principal benefit of Apellis. If I suffer an injury as a result of my participation in the trial, they will pay me compensation in line with industry guidelines.
- I understand that there may be risks associated with the treatment in the event of myself or my partner becoming pregnant. I undertake to inform my partner of the risks and to take responsibility for the prevention of pregnancy

**Statement by Participant**	I hereby consent to take part in this study. I understand that I will receive a signed copy of this consent form for my records.<br><br>________________________ (full name)<br><br>________________________ (signature)<br><br>__ __ / __ __ __ / __ __ (Date)

Statement by Consenter (Investigator/designee) I have discussed this study with the above named participant. The participant appeared to fully understand the information provided about the study.

________________________ (full name)

________________________ (signature)

__ __ / __ __ __ / __ __ (Date)

Insert Header with institution's name or institution's letterhead

PARTICIPANT INFORMATION SHEET

[Insert site name]

**Short Title**	A Phase Ib, Open Label, Multiple Ascending Dose, Pilot Study to Assess the Safety, Preliminary Efficacy and Pharmacokinetics of Subcutaneously Administered APL-2 in Subjects with Paroxysmal Nocturnal Hemoglobinuria (PNH). – PADDOCK		
**Protocol Number**	APL2-CP-PNH-204	**Ethics Number**	<<Insert #>>
**Project Sponsor**	Apellis Pharmaceuticals, Inc.<br>6400 Westwind Way, Suite A, Louisville, KY, 40014		
**Local Sponsor**	Novotech		
**Principal Investigator**	<<Insert Name>>		
**Location**	<<Insert Location>>		
**Study related phone number**	<<Insert Contact Number>>		

You are being invited to take part in the second cohort of a research study of the trial drug APL-2. This consent form gives you important information about this study to help you decide if you want to take part in the research study. It describes the purpose of this study, the study procedures, and the possible risks, and provides information about your rights as a study participant.

Please take the time to read this information carefully. You should talk to your study doctor and study staff about this study and ask any questions you have. You can also discuss this study with other people such as your family or general practitioner (GP). If you decide to take part in this study, you will be asked to sign and date this form. You will receive a copy of the signed form. Your study doctor will tell your GP about your participation in this study.

Who is funding this study?

Apellis Pharmaceuticals, Inc. ("Apellis") is funding this clinical study. Apellis is the "sponsor" of the study, which means that Apellis takes responsibility for starting, managing, and financing the study. <<Insert Site Name>> is being paid by Apellis to do this study.

Voluntary participation and withdrawal from this study

Your participation in this study is voluntary. You are free to say yes or no. Even if you join this study, you may stop at any time.

A description of this clinical trial will be available on the internet at http://www.ClinicalTrials.gov. This website will not include information that can identify you. At most, the website will include a summary of the results. You can search this website at any time.

1. WHAT YOU SHOULD KNOW ABOUT THIS STUDY

a. Why is this study being done?

This is a research study to test a new investigational drug. An investigational drug is one that is not approved by any regulatory authority (e.g. FDA, Department of Health, Medsafe etc) for use outside a clinical trial. Cohort 1 has completed and 2 patients have already completed in excess of 169 days of dosing in Cohort 2. The study was initially set up in New Zealand but insufficient eligible PNH patients exist to complete the study.

You are being invited to participate in this research study because you have been diagnosed with paroxysmal nocturnal hemoglobinuria (PNH). PNH is a rare disorder that causes the red blood cells to break down too early**.** Many PNH patients suffer from complications such as blood clots or low red blood cell counts (anemia) even after receiving standard treatment.

The research study will continue to gather scientific information about the safety, tolerability and continued evidence of activity of the investigational drug APL-2 in people with PNH who have not been treated with Soliris® in the past. The study will help determine how APL-2 behaves inside the body, how it is eliminated from the body, the effect it has on the body after multiple doses and if it can prevent red blood cells from breaking down.

b. How does APL-2 works?

Your body has numerous biological mechanisms designed to attack and kill off invaders, such as bacteria. One of these mechanisms, called the complement cascade, is part of your normal immune system. Normally, only invaders are destroyed by the complement cascade as your own cells are protected against its attacks. However, patients with PNH possess red blood cells that are defective and are not properly protected, which results in the destruction of these defective red blood cells by your own immune system. The breakdown of a large number of red blood cells in this manner has been shown to trigger episodes of PNH. Based on the research conducted prior to this Phase Ib study, the investigational study drug, which is a peptide (small protein), is expected to decrease the activity of the complement cascade and prevent the destruction of defective red blood cells. Results from the ongoing/completed studies also provided good assurance of the safety for the doses of APL-2 in this study.

c. Dosing of APL-2

APL-2 will be given daily, as a subcutaneous (the fat tissue under your skin) infusion using an infusion pump that you will be trained to use and self administer when fully trained and competent in the use of the pump. Until that time, you will attend the clinical site to receive your study medication. If you miss any doses of APL-2 or you wish to stop receving APL-2 you should contact your study doctor immediately before stopping.

You will receive 270 mg APL-2 each day for up to 364 days, although the study doctor may increase your dose up to 360 mg APL-2 each day if a higher dose is needed to reduce breakdown of your red blood cells

The safety monitoring committee will review all the safety data available from participants already dosed in the study approximately every 8 weeks.

The study can be stopped at any time, based on evaluation of the side effects of the study medication.

2. WHAT WILL HAPPEN IF YOU TAKE PART IN THIS STUDY

a. What will you be responsible for if you take part in this study?

If you decide to take part in this study you will be asked to sign this ethics committee-approved informed consent form and you must meet specific entry rules.

You must also agree to the following:

- Be willing and able to attend all visits at the study center.
- Immunisation is required to participate in the study. Vaccinations may be given during the study visit on Day 15 if you have not received the required immunisation within 2 years of the Day 1 visit screening visit. There immunisations will be free as they are required as part of the trial. Additional booster vaccinations will be given on Day 85, if required.
- Be willing to allow frequent blood tests during the study and at subsequent follow up visits.
- You will be requested to keep the study drug supplies in your refrigerator (between 2-8°C) If there is a power failure, you should contact the clinic site.
- Follow the instructions given by the study doctor and study staff.
- Take an antibiotic throughout the study to prevent bacterial infections.
- Take an antiviral throughout the study if you are a hepatitis B carrier, to prevent reactivation of the hepatitis B virus.
- Have all procedures performed.
- Males must agree to no sperm donation at any time during the study.
- Females who are able to have children must agree to use an effective contraception for the duration of the study. Your study doctor can advise you on effective methods of contraception.
- Make sure you take only medications approved by a study doctor. Tell the study doctor what medications you are currently taking or have taken in the past. Also, tell him / her before starting any new medications and/or if there are changes to your current medications.

b. What types of tests or procedures will be involved with continuation in this study?

You will receive / administer daily subcutaneous infusions which will be done by the study staff when you visit the clinic site or by you after you have been fully trained to self administer using the infusion pump supplied for sole use in this study.

<<Insert Site Name>> will contact your regular doctor (GP) about your study participation. If you do not want your GP told, you will not be able to continue to take part in the study. We may need to contact your GP about any health issues or parts of your medical history that might affect your participation in the study.

- **You will be given a Participant Identification Card** when you start this study, you must read and understand what is written on your patient card. You should always carry this card with you as it contains important information about the study. If you are seen by any medical staff who are not involved in the study you must show them this card as it includes information they should be aware of when treating you.

c. What are the study details? What procedures will you have?

Your total time in this study is expected to be approximately 414 days. If more tests are needed, your study doctor may ask you to schedule additional visits which could extend your total time in the study. Your study doctor or Apellis may choose to withdraw you from this research study at any time which could decrease your total time in the study.

The study has 4 parts;

- Part 1: You will receive APL-2 for 28 days and you will visit the study centre at least 8 times.
- Part 2A: If there is evidence of clinical benefit to you by Day 28; you will continue to receive APL-2 up to Day 84. You will visit the study centre 5 more times.
- Part 2B: If there is still evidence of clinical benefit to you by Day 84, you will continue to receive daily APL-2 treatment up to Day 364. Part 2B will include at least 10 visits to the study center. If there is evidence that you are no longer getting clinical benefit, treatment will be stopped and you will skip Part 2B and enter Part 3 for safety follow-up visits

- Part 3: Safety follow-up will include 4 visits to the study center.

You will be monitored throughout the study for any changes in health. Throughout the entire duration of the study, the <<Insert Site Name>> staff will continue to review any other medications you may be taking and any potential side effects to ensure your safety.

The following activities will be performed during site visits:

Screening
Screening Visits (2 visits) Day -30 to Day -1

Before we can do any tests, you will be asked to give written informed consent, below are details of assessments that need to be done:

- Questions about your health and what medication you are taking
- Review of your demographic (personal) information such as date of birth, ethnicity, and race
- Review the study requirements and restrictions
- Height and weight
- Set of vital signs (pulse, blood pressure and breathing rate) and temperature
- Full physical exam
- An ECG, which is a painless test that looks at the electrical activity of your heart by putting small sticky patches on certain areas of your body while you are lying down. These patches are connected to thin wires which are again connected to a machine which will interpret the electrical activity of your heart and then print a report.
- Set of routine blood and urine samples, blood samples relating to safety and PNH diagnosis
- Blood tests for HIV, Hepatitis B and Hepatitis C
- Pregnancy test (females) +/- hormone test (postmenopausal females)
- Your doctor will discuss with you what vaccinations you may need and will also take into account vaccinations that you have previously received.

A final decision about whether you can take part will then be made when all your screening results are available.

Part 1: Site Visits (Day 1 – Day 28)

APL-2 dosing:

- You will receive the first 3 daily doses of APL-2 (Day 1 to 3 or beyond. You will continue to attend the hospital clinic on a daily basis until you are competent and confident to use the infusion pump for self administer the study drug at home), as well as doses on Day 8, 15 and 22 at the hospital clinic. You will need to stay in the clinic for 2.5 hours after receiving the first dose of APL-2 on Day 1 so we can make sure that you are okay.

Other assessments done throughout the study:

- You will be asked what medications you are taking and if you have had any recent illnesses or changes in your condition.
- Preventative Antibiotics will be taken from Day 1 until 14 days after the last dose of APL-2. The antibiotic will be changed on Day 15 once vaccinations have been received.
- Preventative Antivirals will be taken from Day 1 until 14 days after the last dose of APL-2 if you are a hepatitis B carrier
- Vaccinations, if required, will be given at the clinic visit on Day 15
- Full physical exam
- An ECG (recording of the electrical activity of your heart).

- Set of vital signs (pulse, blood pressure and breathing rate) and temperature. If the visit includes study drug administration, vital signs will be measured before and after you receive the study drug.
- Set of routine blood and urine samples, blood samples relating to safety and PNH diagnosis
- You will be assessed for signs/symptoms of blood clots.
- Pregnancy test (for females who are able to become pregnant).
- FACIT (Fatigue Scale) is a 13 item questionnaire which you will need to complete during clinic visits on Days 1 and 15.
- Monitor any side effects: you will be asked to notify of any unfavourable health or medical events, referred to as adverse events, experienced from the last visit.
- Vaccination (booster) on Day 57 (during Part 2A or Part 3 Follow up)

You will self administer the study drug on a daily basis (after you have been trained on the use of the infusion pump) on the following study days 4 (or from when you feel confident and are considered competent to self administer) -7, 9-14, 16-21, and 23-28

Part 2A: Site Visits (Day 29 – Day 84)

APL-2 dosing:

You will receive doses on Day 29, 36, 43, 57 and 71 at the hospital clinic. You will self administer the study drug on a daily basis on the following study days 30-35, 37-42, 44-56, 58-70 and 72-84.

Other assessments done throughout the study:

- You will be asked what medications you are taking and if you have had any recent illnesses or changes in your condition.
- Preventative Antibiotics will be taken from Day 1 until 14 days after the last dose of APL-2
- Preventative Antivirals will be taken from Day 1 until 14 days after the last dose of APL-2 if you are a hepatitis B carrier

- Full physical exam
- An ECG (recording of the electrical activity of your heart).
- Set of vital signs (pulse, blood pressure and breathing rate) and temperature. If the visit includes study drug administration, vital signs will be measured before and after you receive the study drug.
- Set of routine blood and urine samples, blood samples relating to safety and PNH diagnosis
- You will be assessed for signs/symptoms of blood clots.
- Pregnancy test (for females who are able to become pregnant).
- FACIT (Fatigue Scale) is a 13 item questionnaire which you will need to complete during clinic visits on Days 29, 43 and 71.
- Monitor any side effects: you will be asked to notify of any unfavourable health or medical events, referred to as adverse events, experienced from the last visit.
-

Part 2B: Site Visits (Day 85 onwards)

APL-2 dosing:

- You will receive doses of APL-2 on Day 85, 113, 141, 169, 197, 225, 253, 281, 309 and 337 at the hospital clinic. You will self administer the study drug on a daily basis on all other days in between your hospital clinic visits.

Other assessments done throughout the study:

- You will be asked what medications you are taking and if you have had any recent illnesses or changes in your condition.

- Preventative Antibiotics will be taken from Day 1 until 14 days after the last dose of APL-2
- Preventative Antivirals will be taken from Day 1 until 14 days after the last dose of APL-2 if you are a hepatitis B carrier
- Booster vaccinations, if required, will be given at the Day 85 clinic visit

- Full physical exam
- An ECG (recording of the electrical activity of your heart).
- Set of vital signs (pulse, blood pressure and breathing rate) and temperature. If the visit includes study drug administration, vital signs will be measured before and after you receive the study drug.
- Set of routine blood and urine samples, blood samples relating to safety and PNH diagnosis
- You will be assessed for signs/symptoms of blood clots.
- Pregnancy test (for females who are able to become pregnant).
- FACIT (Fatigue Scale) is a 13 item questionnaire which you will need to complete during clinic visits on Days 85,141,197, 253, 309, 365 and 414.
- Monitor any side effects: you will be asked to notify of any unfavourable health or medical events, referred to as adverse events, experienced from the last visit.

Safety Follow-up

You will be asked to return to the clinic site for follow-up visits on Days 365, 379, and 393.

After you have completed all follow-up procedures you need to return for the Exit Visit on Day 414.

You will not receive any study drug at these visits. These visits will include the following tests and procedures:

- You will be asked what medications you are taking and if you have had any illnesses or changes in your condition.
- Your vital signs (temperature, resting pulse and blood pressure, respiratory rate) will be measured.
- You will be assessed for signs/symptoms of blood clots.
- Electrocardiogram (ECG) – Day 365.
- Physical examination – Day 365 and 414.
- Collection of a urine sample for routine lab tests.
    - For women who are able to get pregnant, urine will be collected at Day 365, 393 and 414 for a pregnancy test.
- Collection of blood.
- You will be asked to complete a FACIT (Fatigue Scale) questionnaire at Day 365, 393 and 414.

3. WHAT WILL HAPPEN TO MY TEST SAMPLES

The following blood samples will be collected throughout the study:

- Samples to determine regular routine blood counts and blood chemistry, to monitor your general health. (Haematology and Chemistry)
- Blood tests for HIV, Hepatitis B and Hepatitis C (Serology)
- Blood tests to determine how quickly your blood clots (Coagulation Profile)
- Pregnancy Test (If applicable)

All these routine samples will be sent to a Central lab overseas for testing and destroyed at the end of the study by internationally accepted means e.g. incineration.

The following blood samples will also be collected throughout the study. These samples will be sent to different locations for testing.

- Blood samples which determine the level of study drug in your blood (Pharmacokinetics).

- Blood samples to determine the effects of study drug on your body (Pharmacodynamics).
- Blood samples for immunogenicity (The ability to cause the production of antibodies to the study drug).
- Blood samples for cytometry testing (This test is able to identify the type/amount of cells in different parts of the cell cycle).

These blood samples may be stored for up to 1 year after the end of treatment after which time they will be destroyed by internationally accepted means e.g. incineration.

The maximum amount of blood collected from each participant during the study will be approximately 580 mL (Approximately 39 tablespoons).

All samples will be coded; identified only by each participant's study number and initials. Only hospital and laboratory staff will have access to the blood samples.

4. SAFETY: POTENTIAL RISKS AND DISCOMFORTS

a. Are there any risks from your participation in this study?

There may be risks to taking part in this study from APL-2 or from some of the procedures or tests done in this study.

If you decide to take part in this study, you or your family members should tell the study doctor or <<Insert Site Name>> staff immediately if you have any unusual health problems, injuries, or side effects, even if you do not think these problems are caused by the study or APL-2.

If there is any new important safety information or other information that could affect your willingness to participate in this study, your study doctor will let you know.

b. What are the likely risks with APL-2?

You may feel discomfort during some of the tests and study procedures. Possible risks, side effects, and discomforts include:

- Infections – you may be at increased risk for infections caused by certain types of bacteria such as Streptococcus pneumonia, Neisseria Meningitidis, or Haemophilus influenza.
- Allergic reactions – all drugs have a potential risk of an allergic reaction, which if not treated promptly, could become life-threatening.
- Blood samples - you may have discomfort or pain when your blood is collected. You may feel faint or pass out. There is also a slight possibility of infection, bruising, or bleeding at the puncture site. Your anaemia (low red blood cell count) could worsen from having frequent blood draws
- ECG - skin irritation is rare but could occur during an ECG from the sticky patches or gel that is used
- Antibiotic and antiviral therapy – there is a possibility that you will experience some side effects from the antibiotic treatment you will need to take during this study. Side effects such as nausea, sickness, diarrhoea, dizziness, thrush, and skin rashes may occur and if you experience any of these you should inform the nurse or doctor. The doctor may decide that you should change to a different antibiotic to try and reduce these symptoms.
- Injection site reactions - there is a possibility that you could experience redness, swelling, and pain or tenderness at the site of injection. There is also a slight possibility of infection.

c. Any unforeseen risks?

Since the study drug APL-2 is investigational when taken alone or in combination with other

medications, there may be other risks that are unknown.

It is important that you adhere to your APL-2 dosing schedule. APL-2 doses should not be missed. Delayed or missed doses may put you at an increased risk for return of symptoms and potentially serious complications associated with PNH. If you miss any doses you should contact your study doctor immediately.

d. Could APL-2 be harmful to an unborn or breastfed baby?

You should discuss pregnancy risks with your doctor before signing this consent form. Women who are pregnant, planning to become pregnant or breast feeding may not participate in this research study. Taking the study drug may involve unknown risks to a pregnant woman, an embryo, fetus (unborn baby) or nursing infant. If you (or your partner) become pregnant while in this study, the sponsor may ask to follow the outcome of the pregnancy. The study drug will be stopped and your participation in this study will be ended.

Before taking part in this research study and during the study, females able to have children will have pregnancy tests done. Talk to your doctor about the best method of birth control to use while you are in this study. It is important that you call your study doctor at <<***insert phone number***>> right away if you become pregnant or father a child during the course of this study.

5. BENEFITS OF PARTICIPATION

Are there any benefits to your participation in this study?

You may or may not benefit from participation in this study. There is no guarantee that you will continue to benefit from participating in this study. The information from this study might help to develop better treatments in the future for people with PNH. An indirect benefit of taking part in this study is that you will receive free medical tests at screening and during the study.

What are the alternatives to taking part? Do you have any other choices?

You do not have to be in this study to receive treatment for your PNH. You have the option to receive standard treatment for PNH. Standard treatment may include medications such as prednisone, or blood thinners; blood transfusions; or bone marrow transplantation. The study doctor will discuss with you the risks and benefits of the alternative treatments.

6. POTENTIAL COSTS/ REIMBURSEMENTS

There will be no charge to you for your participation in this study. The study drug, study-related procedures, and study visits will be provided at no charge to you.

Will you be paid for your samples (such as blood or urine) or medical information?

You will not be paid for the use of your samples or any information, discoveries or inventions obtained from your samples or medical information.

You will be reimbursed for any reasonable travel, accommodation, parking, meals and other expenses associated with the research project visit, this will based on the distance that you have to travel to the hospital.

7. WHAT ARE THE RIGHTS OF PARTICIPANTS IN THIS STUDY?

a. Participation is voluntary

Participation in any research project is voluntary. If you do not wish to take part, you do not have to. If you decide to take part and later change your mind, you are free to withdraw from the project at any stage. This will not affect your routine treatment, your relationship with those treating you or with <<Insert Site Name>> .

If you do decide to take part, you will be given this Participant Information and Consent Form to sign and you will be given a copy to keep.

b. New information
Sometimes during the course of a research project, new information becomes available about the treatment that is being studied. If this happens, your study doctor will tell you about it and discuss with you whether you want to continue in the research project. If you decide to withdraw, your study doctor will make arrangements for your regular health care to continue. If you decide to continue in the research project you may be asked to sign an updated consent form.

Also, on receiving new information, your study doctor might consider it to be in your best interests to withdraw you from the research project. If this happens, he/ she will explain the reasons and arrange for your regular health care to continue.

c. Right to access information collected during the study

In accordance with relevant New Zealand privacy and other relevant laws, you have the right to request access to your information collected and stored by the research team. You also have the right to request that any information with which you disagree be corrected. Please contact the study team member named at the end of this document if you would like to access your information.

8. CONFIDENTIALITY

By signing the consent form you consent to the study doctor and relevant research staff collecting and using personal information about you for the research project. Any information obtained in connection with this research project that can identify you will remain confidential. Your name, address or phone number will not be on the study forms; instead you will be identified by a unique participant study number, date of birth and initials. Your information will only be used for the purpose of this research project and it will only be disclosed with your permission, except as required by law.

Information about you may be obtained from your health records held at this and other health services for the purpose of this research. By signing the consent form you agree to the study team accessing health records if they are relevant to your participation in this research project.

Your health records and any information obtained during the research project are subject to inspection (for the purpose of verifying the procedures and the data) by the relevant authorities (such as the Ethics Committee that has reviewed and approved this study) and authorised representatives of the Sponsor, Apellis, and <<Insert Site Name>> or as required by law. All personnel reviewing your medical records will be required to keep the information confidential in accordance with law. By signing the Consent Form, you authorise release of, or access to, this confidential information to the relevant study personnel and regulatory authorities as noted above.

When the research project ends the study data must be analysed, so the results of the study may not be available until about six months after the research finishes. The study doctors and/or Apellis may decide to discuss or publish the results of the study. This may include publication in journals, presentation at conferences or other professional forums. In any publication, information will be provided in such a way that you cannot be identified. Results of the study will be provided to you, if you wish.

Information about your participation in this research project may be recorded in your health records.

Any information obtained for the purpose of this research project that can identify you will be treated as confidential and securely stored. It will be disclosed only with your permission, or as required by law.

Upon completion of the trial, the study file containing information about you will be archived for a minimum of fifteen years.

9. COMPENSATION FOR INJURY

If you were injured as a result of treatment given as part of this study, compensation would be available from the study's sponsor, Apellis, in line with industry guidelines. We can give you a copy of these guidelines if you wish.

If you have private health or life insurance, you may wish to check with your insurer that taking part in this study won't affect your cover.

10. WHAT WILL HAPPEN AFTER THE STUDY ENDS, OR IF YOU PULL OUT?

If you decide to withdraw
Please notify a member of the research team before you withdraw. This notice will allow that person or the research supervisor to discuss any health risks or special requirements linked to withdrawing, such as follow up visits.

If you do withdraw your consent during the research project, information already collected will be retained to ensure that the results of the research project can be measured properly and to comply with law. You should be aware that data collected by the sponsor up to the time you withdraw will form part of the research project results. If you do not want them to do this, you must tell them before you join the research project.

Why the study might be unexpectedly stopped
This research project may be stopped unexpectedly for a variety of reasons. These may include reasons such as:
• Unacceptable side effects
• The drug being shown not to be effective
• The drug being shown to work and not need further testing.

Will the study treatment continue to be available after the study finished?

If it is found the drug does continue to have a beneficial effect in reducing haemolysis (breakdown of red blood cells), and no safety concerns are raised, the company may, at its sole discretion, elect to continue the drug therapy by offering a further extension to the study.

Where can you go for more information about the study, or to raise concerns or complaints?

If you have any questions, concerns or complaints about the study at any stage, you can contact:

<<Insert Name>>, lead study doctor
Phone: <<Insert Contact Number>>and ask operator to page or put you through to <<Insert Name>>
Research Coordinator: <<Insert Contact Number>>

If you want to talk to someone who isn't involved with the study, you can contact an independent health and disability advocate. Specialist advocates, are available here.

Phone:
Fax:
Email:

You can also contact the health and disability ethics committee that approved this study on:

Phone:
Email:

12. DO I HAVE TO DECIDE STRAIGHT AWAY?

No, you do not have to decide straight away. You should take some time to consider whether or not to continue your participation in the study. Study staff will let you know when they will need your decision. The following steps are useful in helping you reach a decision.

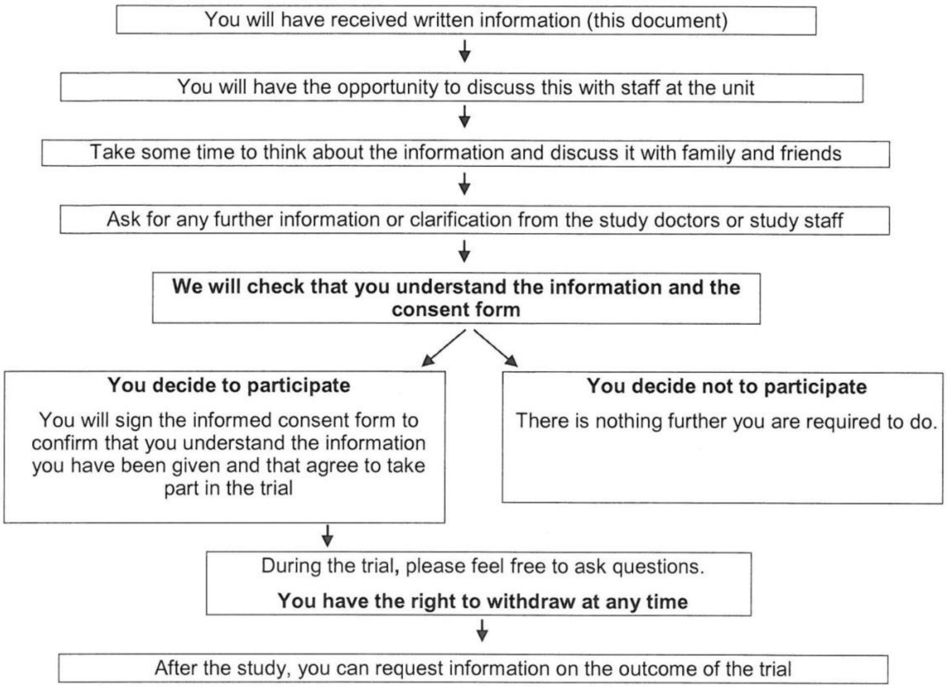

Insert Header with institution's name or institution's letterhead

INFORMED CONSENT FORM

**Short Title**	Nocturnal Hemoglobinuria (PNH) – PADDOCK
**Protocol Number**	APL2-CP-PNH-204
**Principal Investigator**	<<Insert Name>>

Declaration by participant:

I have read and I understand this version of the Participant Information Sheet. I have had the opportunity to ask questions and I am satisfied with the answers I have received.

I have had the opportunity to discuss the study with my family.

I freely agree to continue to participate in this study and understand that I may withdraw from the study at any time.

I understand that:

- My participation in this study is confidential and I will not be identified in study reports.
- My medical records may be reviewed by Apellis, companies working with them, auditors and health or regulatory agencies for the purpose of checking the accuracy of the information recorded for the study
- Some of my samples will be sent overseas for analysis and they will be destroyed as described in the Information Sheet.
- If I decide to withdraw from the study, information already collected may continue to be processed. I may be asked to attend follow-up visits.
- The study drugs or tests will be stopped if they appear harmful to me.
- I should contact site staff if I have any side effects.
- My GP and/or specialist will be informed of my participation in this study and relevant medical records may be accessed.
- I am not giving up any of my legal rights by signing this consent form.
- This trial is being carried out for the principal benefit of Apellis. If I suffer an injury as a result of my participation in the trial, they will pay me compensation in line with industry guidelines.
- I understand that there may be risks associated with the treatment in the event of myself or my partner becoming pregnant. I undertake to inform my partner of the risks and to take responsibility for the prevention of pregnancy

Statement by Participant I hereby consent to take part in this study. I understand that I will receive a signed copy of this consent form for my records.

____ (full name)

____ (signature)

__ __ / __ __ __ / __ __ (Date)

Statement by Consenter (Investigator/designee) I have discussed this study with the above named participant. The participant appeared to fully understand the information provided about the study.

____ (full name)

____ (signature)

__ __ / __ __ __ / __ __ (Date)

Insert Header with institution's name or institution's letterhead

PARTICIPANT INFORMATION SHEET

[Insert site name]

**Short Title**	A Phase Ib, Open Label, Multiple Ascending Dose, Pilot Study to Assess the Safety, Preliminary Efficacy and Pharmacokinetics of Subcutaneously Administered APL-2 in Subjects with Paroxysmal Nocturnal Hemoglobinuria (PNH). – PADDOCK		
**Protocol Number**	APL2-CP-PNH-204	**Ethics Number**	<<Insert #>>
**Project Sponsor**	Apellis Pharmaceuticals, Inc.<br>6400 Westwind Way, Suite A, Louisville, KY, 40014		
**Local Sponsor**	Novotech		
**Principal Investigator**	<<Insert Name>>		
**Location**	<<Insert Location>>		
**Study related phone number**	<<Insert Contact Number>>		

You are being invited to take part in the second cohort of a research study of the trial drug APL-2. This consent form gives you important information about this study to help you decide if you want to take part in the research study. It describes the purpose of this study, the study procedures, and the possible risks, and provides information about your rights as a study participant.

Please take the time to read this information carefully. You should talk to your study doctor and study staff about this study and ask any questions you have. You can also discuss this study with other people such as your family or general practitioner (GP). If you decide to take part in this study, you will be asked to sign and date this form. You will receive a copy of the signed form. Your study doctor will tell your GP about your participation in this study.

Who is funding this study?

Apellis Pharmaceuticals, Inc. ("Apellis") is funding this clinical study. Apellis is the "sponsor" of the study, which means that Apellis takes responsibility for starting, managing, and financing the study. <<Insert Site Name>> is being paid by Apellis to do this study.

Voluntary participation and withdrawal from this study

Your participation in this study is voluntary. You are free to say yes or no. Even if you join this study, you may stop at any time.

A description of this clinical trial will be available on the internet at http://www.ClinicalTrials.gov. This website will not include information that can identify you. At most, the website will include a summary of the results. You can search this website at any time.

1. WHAT YOU SHOULD KNOW ABOUT THIS STUDY

a. Why is this study being done?

This is a research study to test a new investigational drug. An investigational drug is one that is not approved by any regulatory authority (e.g. FDA, Department of Health, Medsafe etc) for use outside a clinical trial. Cohort 1 has completed and 2 patients have already completed in excess of 169 days of dosing in Cohort 2. The study was initially set up in New Zealand but insufficient eligible PNH patients exist to complete the study.

You are being invited to participate in this research study because you have been diagnosed with paroxysmal nocturnal hemoglobinuria (PNH). PNH is a rare disorder that causes the red blood cells to break down too early**.** Many PNH patients suffer from complications such as blood clots or low red blood cell counts (anemia) even after receiving standard treatment.

The research study will continue to gather scientific information about the safety, tolerability and continued evidence of activity of the investigational drug APL-2 in people with PNH who have not been treated with Soliris® in the past. The study will help determine how APL-2 behaves inside the body, how it is eliminated from the body, the effect it has on the body after multiple doses and if it can prevent red blood cells from breaking down.

b. How does APL-2 works?

Your body has numerous biological mechanisms designed to attack and kill off invaders, such as bacteria. One of these mechanisms, called the complement cascade, is part of your normal immune system. Normally, only invaders are destroyed by the complement cascade as your own cells are protected against its attacks. However, patients with PNH possess red blood cells that are defective and are not properly protected, which results in the destruction of these defective red blood cells by your own immune system. The breakdown of a large number of red blood cells in this manner has been shown to trigger episodes of PNH. Based on the research conducted prior to this Phase Ib study, the investigational study drug, which is a peptide (small protein), is expected to decrease the activity of the complement cascade and prevent the destruction of defective red blood cells. Results from the ongoing/completed studies also provided good assurance of the safety for the doses of APL-2 in this study.

c. Dosing of APL-2

APL-2 will be given daily, as a subcutaneous (the fat tissue under your skin) infusion using an infusion pump that you will be trained to use and self administer when fully trained and competent in the use of the pump. Until that time, you will attend the clinical site to receive your study medication. If you miss any doses of APL-2 or you wish to stop receiving APL-2 you should contact your study doctor immediately before stopping.

You will receive 270 mg APL-2 each day for up to 364 days, although the study doctor may increase your dose up to 360 mg APL-2 each day if a higher dose is needed to reduce breakdown of your red blood cells

The safety monitoring committee will review all the safety data available from participants already dosed in the study approximately every 8 weeks.

The study can be stopped at any time, based on evaluation of the side effects of the study medication.

2. WHAT WILL HAPPEN IF YOU TAKE PART IN THIS STUDY

a. What will you be responsible for if you take part in this study?

If you decide to take part in this study you will be asked to sign this ethics committee-approved informed consent form and you must meet specific entry rules.

You must also agree to the following:

- Be willing and able to attend all visits at the study center.
- Immunisation is required to participate in the study. Vaccinations may be given during the study visit on Day 15 if you have not received the required immunisation within 2 years of the Day 1 visit screening visit. There immunisations will be free as they are required as part of the trial. Additional booster vaccinations will be given on Day 85, if required.
- Be willing to allow frequent blood tests during the study and at subsequent follow up visits.
- You will be requested to keep the study drug supplies in your refrigerator (between 2-8ºC) If there is a power failure, you should contact the clinic site.
- Follow the instructions given by the study doctor and study staff.
- Take an antibiotic throughout the study to prevent bacterial infections.
- Take an antiviral throughout the study if you are a hepatitis B carrier, to prevent reactivation of the hepatitis B virus.
- Have all procedures performed.
- Males must agree to use an effective contraception method and agree to no sperm donation at any time during the study and 90 days after their last dose of study drug.
- Females who are able to have children must agree to use an effective contraception for the duration of the study and 90 days after their last dose of study drug. Your study doctor can advise you on effective methods of contraception.
- Make sure you take only medications approved by a study doctor. Tell the study doctor what medications you are currently taking or have taken in the past. Also, tell him / her before starting any new medications and/or if there are changes to your current medications.

b. What types of tests or procedures will be involved with continuation in this study?

You will receive / administer daily subcutaneous infusions which will be done by the study staff when you visit the clinic site or by you after you have been fully trained to self administer using the infusion pump supplied for sole use in this study.

<<Insert Site Name>> will contact your regular doctor (GP) about your study participation. If you do not want your GP told, you will not be able to continue to take part in the study. We may need to contact your GP about any health issues or parts of your medical history that might affect your participation in the study.

- **You will be given a Participant Identification Card** when you start this study, you must read and understand what is written on your patient card. You should always carry this card with you as it contains important information about the study. If you are seen by any medical staff who are not involved in the study you must show them this card as it includes information they should be aware of when treating you.

c. What are the study details? What procedures will you have?

Your total time in this study is expected to be approximately 414 days. If more tests are needed, your study doctor may ask you to schedule additional visits which could extend your total time in the study. Your study doctor or Apellis may choose to withdraw you from this research study at any time which could decrease your total time in the study.

The study has 4 parts;

- Part 1: You will receive APL-2 for 28 days and you will visit the study centre at least 8 times.
- Part 2A: If there is evidence of clinical benefit to you by Day 28; you will continue to receive APL-2 up to Day 84. You will visit the study centre 5 more times.
- Part 2B: If there is still evidence of clinical benefit to you by Day 84, you will continue to receive daily APL-2 treatment up to Day 364. Part 2B will include at least 10 visits to the study center.

If there is evidence that you are no longer getting clinical benefit, treatment will be stopped and you will skip Part 2B and enter Part 3 for safety follow-up visits

- Part 3: Safety follow-up will include 4 visits to the study center.

You will be monitored throughout the study for any changes in health. Throughout the entire duration of the study, the <<Insert Site Name>> staff will continue to review any other medications you may be taking and any potential side effects to ensure your safety.

The following activities will be performed during site visits:

**Screening**<br>**Screening Visits (2 visits) Day -30 to Day -1**
Before we can do any tests, you will be asked to give written informed consent, below are details of assessments that need to be done:<br>• Questions about your health and what medication you are taking<br>• Review of your demographic (personal) information such as date of birth, ethnicity, and race<br>• Review the study requirements and restrictions<br>• Height and weight<br>• Set of vital signs (pulse, blood pressure and breathing rate) and temperature<br>• Full physical exam<br>• An ECG, which is a painless test that looks at the electrical activity of your heart by putting small sticky patches on certain areas of your body while you are lying down. These patches are connected to thin wires which are again connected to a machine which will interpret the electrical activity of your heart and then print a report.<br>• Set of routine blood and urine samples, blood samples relating to safety and PNH diagnosis<br>• Blood tests for HIV, Hepatitis B and Hepatitis C<br>• Pregnancy test (females) +/- hormone test (postmenopausal females)<br>• Your doctor will discuss with you what vaccinations you may need and will also take into account vaccinations that you have previously received.
**A final decision about whether you can take part will then be made when all your screening results are available.**

**Part 1: Site Visits (Day 1 – Day 28)**
**APL-2 dosing:**<br>• You will receive the first 3 daily doses of APL-2 (Day 1 to 3 or beyond. You will continue to attend the hospital clinic on a daily basis until you are competent and confident to use the infusion pump for self administer the study drug at home), as well as doses on Day 8, 15 and 22 at the hospital clinic. You will need to stay in the clinic for 2.5 hours after receiving the first dose of APL-2 on Day 1 so we can make sure that you are okay.<br><br>**Other assessments done throughout the study:**<br>• You will be asked what medications you are taking and if you have had any recent illnesses or changes in your condition.<br>• Preventative Antibiotics will be taken from Day 1 until 14 days after the last dose of APL-2. The antibiotic will be changed on Day 15 once vaccinations have been received.<br>• Preventative Antivirals will be taken from Day 1 until 14 days after the last dose of APL-2 if you are a hepatitis B carrier<br>• Vaccinations, if required, will be given at the clinic visit on Day 15<br>• Full physical exam

- An ECG (recording of the electrical activity of your heart).
- Set of vital signs (pulse, blood pressure and breathing rate) and temperature. If the visit includes study drug administration, vital signs will be measured before and after you receive the study drug.
- Set of routine blood and urine samples, blood samples relating to safety and PNH diagnosis
- You will be assessed for signs/symptoms of blood clots.
- Pregnancy test (for females who are able to become pregnant).
- FACIT (Fatigue Scale) is a 13 item questionnaire which you will need to complete during clinic visits on Days 1 and 15.
- Monitor any side effects: you will be asked to notify of any unfavourable health or medical events, referred to as adverse events, experienced from the last visit.
- Vaccination (booster) on Day 57 (during Part 2A or Part 3 Follow up)

You will self administer the study drug on a daily basis (after you have been trained on the use of the infusion pump) on the following study days 4 (or from when you feel confident and are considered competent to self administer) -7, 9-14, 16-21, and 23-28

Part 2A: Site Visits (Day 29 – Day 84)

APL-2 dosing:

You will receive doses on Day 29, 36, 43, 57 and 71 at the hospital clinic. You will self administer the study drug on a daily basis on the following study days 30-35, 37-42, 44-56, 58-70 and 72-84.

Other assessments done throughout the study:

- You will be asked what medications you are taking and if you have had any recent illnesses or changes in your condition.
- Preventative Antibiotics will be taken from Day 1 until 14 days after the last dose of APL-2
- Preventative Antivirals will be taken from Day 1 until 14 days after the last dose of APL-2 if you are a hepatitis B carrier

- Full physical exam
- An ECG (recording of the electrical activity of your heart).
- Set of vital signs (pulse, blood pressure and breathing rate) and temperature. If the visit includes study drug administration, vital signs will be measured before and after you receive the study drug.
- Set of routine blood and urine samples, blood samples relating to safety and PNH diagnosis
- You will be assessed for signs/symptoms of blood clots.
- Pregnancy test (for females who are able to become pregnant).
- FACIT (Fatigue Scale) is a 13 item questionnaire which you will need to complete during clinic visits on Days 29, 43 and 71.
- Monitor any side effects: you will be asked to notify of any unfavourable health or medical events, referred to as adverse events, experienced from the last visit.
-

Part 2B: Site Visits (Day 85 onwards)

APL-2 dosing:

- You will receive doses of APL-2 on Day 85, 113, 141, 169, 197, 225, 253, 281, 309 and 337 at the hospital clinic. You will self administer the study drug on a daily basis on all other days in between your hospital clinic visits.

Other assessments done throughout the study:

- You will be asked what medications you are taking and if you have had any recent illnesses or changes in your condition.
- Preventative Antibiotics will be taken from Day 1 until 14 days after the last dose of APL-2
- Preventative Antivirals will be taken from Day 1 until 14 days after the last dose of APL-2 if you are a hepatitis B carrier
- Booster vaccinations, if required, will be given at the Day 85 clinic visit

- Full physical exam
- An ECG (recording of the electrical activity of your heart).
- Set of vital signs (pulse, blood pressure and breathing rate) and temperature. If the visit includes study drug administration, vital signs will be measured before and after you receive the study drug.
- Set of routine blood and urine samples, blood samples relating to safety and PNH diagnosis
- You will be assessed for signs/symptoms of blood clots.
- Pregnancy test (for females who are able to become pregnant).
- FACIT (Fatigue Scale) is a 13 item questionnaire which you will need to complete during clinic visits on Days 85,141,197, 253, 309, 365 and 414.
- Monitor any side effects: you will be asked to notify of any unfavourable health or medical events, referred to as adverse events, experienced from the last visit.

Safety Follow-up

You will be asked to return to the clinic site for follow-up visits on Days 365, 379, and 393.

After you have completed all follow-up procedures you need to return for the Exit Visit on Day 414.

You will not receive any study drug at these visits. These visits will include the following tests and procedures:

- You will be asked what medications you are taking and if you have had any illnesses or changes in your condition.
- Your vital signs (temperature, resting pulse and blood pressure, respiratory rate) will be measured.
- You will be assessed for signs/symptoms of blood clots.
- Electrocardiogram (ECG) – Day 365.
- Physical examination – Day 365 and 414.
- Collection of a urine sample for routine lab tests.
  - For women who are able to get pregnant, urine will be collected at Day 365, 393 and 414 for a pregnancy test.
- Collection of blood.
- You will be asked to complete a FACIT (Fatigue Scale) questionnaire at Day 365, 393 and 414.

3. WHAT WILL HAPPEN TO MY TEST SAMPLES

The following blood samples will be collected throughout the study:

- Samples to determine regular routine blood counts and blood chemistry, to monitor your general health. (Haematology and Chemistry)
- Blood tests for HIV, Hepatitis B and Hepatitis C (Serology)
- Blood tests to determine how quickly your blood clots (Coagulation Profile)
- Pregnancy Test (If applicable)

All these routine samples will be sent to a Central lab overseas for testing and destroyed at the end of the study by internationally accepted means e.g. incineration.

The following blood samples will also be collected throughout the study. These samples will be sent to different locations for testing.

- Blood samples which determine the level of study drug in your blood (Pharmacokinetics).
- Blood samples to determine the effects of study drug on your body (Pharmacodynamics).
- Blood samples for immunogenicity (The ability to cause the production of antibodies to the study drug).
- Blood samples for cytometry testing (This test is able to identify the type/amount of cells in different parts of the cell cycle).

These blood samples may be stored for up to 1 year after the end of treatment after which time they will be destroyed by internationally accepted means e.g. incineration.

The maximum amount of blood collected from each participant during the study will be approximately 580 mL (Approximately 39 tablespoons).

All samples will be coded; identified only by each participant's study number and initials. Only hospital and laboratory staff will have access to the blood samples.

4. SAFETY: POTENTIAL RISKS AND DISCOMFORTS

a. Are there any risks from your participation in this study?

There may be risks to taking part in this study from APL-2 or from some of the procedures or tests done in this study.

If you decide to take part in this study, you or your family members should tell the study doctor or <<Insert Site Name>> staff immediately if you have any unusual health problems, injuries, or side effects, even if you do not think these problems are caused by the study or APL-2.

If there is any new important safety information or other information that could affect your willingness to participate in this study, your study doctor will let you know.

b. What are the likely risks with APL-2?

You may feel discomfort during some of the tests and study procedures. Possible risks, side effects, and discomforts include:

- Infections – you may be at increased risk for infections caused by certain types of bacteria such as Streptococcus pneumonia, Neisseria Meningitidis, or Haemophilus influenza.
- Allergic reactions – all drugs have a potential risk of an allergic reaction, which if not treated promptly, could become life-threatening.
- Blood samples - you may have discomfort or pain when your blood is collected. You may feel faint or pass out. There is also a slight possibility of infection, bruising, or bleeding at the puncture site. Your anaemia (low red blood cell count) could worsen from having frequent blood draws
- ECG - skin irritation is rare but could occur during an ECG from the sticky patches or gel that is used
- Antibiotic and antiviral therapy – there is a possibility that you will experience some side effects from the antibiotic treatment you will need to take during this study. Side effects such as nausea, sickness, diarrhoea, dizziness, thrush, and skin rashes may occur and if you experience any of these you should inform the nurse or doctor. The doctor may decide that you should change to a different antibiotic to try and reduce these symptoms.
- Injection site reactions - there is a possibility that you could experience redness, swelling, and pain or tenderness at the site of injection. There is also a slight possibility of infection.

c. Any unforeseen risks?

Since the study drug APL-2 is investigational when taken alone or in combination with other medications, there may be other risks that are unknown.

It is important that you adhere to your APL-2 dosing schedule. APL-2 doses should not be missed. Delayed or missed doses may put you at an increased risk for return of symptoms and potentially serious complications associated with PNH. If you miss any doses you should contact your study doctor immediately.

d. Could APL-2 be harmful to an unborn or breastfed baby?

You should discuss pregnancy risks with your doctor before signing this consent form. Women who are pregnant, planning to become pregnant or breast feeding may not participate in this research study. Taking the study drug may involve unknown risks to a pregnant woman, an embryo, fetus (unborn baby) or nursing infant. If you (or your partner) become pregnant while in this study, the sponsor may ask to follow the outcome of the pregnancy. The study drug will be stopped and your participation in this study will be ended.

Before taking part in this research study and during the study, females able to have children will have pregnancy tests done. Talk to your doctor about the best method of birth control to use while you are in this study. It is important that you call your study doctor at <<***insert phone number***>> right away if you become pregnant or father a child during the course of this study.

5. BENEFITS OF PARTICIPATION

Are there any benefits to your participation in this study?

You may or may not benefit from participation in this study. There is no guarantee that you will continue to benefit from participating in this study. The information from this study might help to develop better treatments in the future for people with PNH. An indirect benefit of taking part in this study is that you will receive free medical tests at screening and during the study.

What are the alternatives to taking part? Do you have any other choices?

You do not have to be in this study to receive treatment for your PNH. You have the option to receive standard treatment for PNH. Standard treatment may include medications such as prednisone, or blood thinners; blood transfusions; or bone marrow transplantation. The study doctor will discuss with you the risks and benefits of the alternative treatments.

6. POTENTIAL COSTS/ REIMBURSEMENTS

There will be no charge to you for your participation in this study. The study drug, study-related procedures, and study visits will be provided at no charge to you.

Will you be paid for your samples (such as blood or urine) or medical information?

You will not be paid for the use of your samples or any information, discoveries or inventions obtained from your samples or medical information.

You will be reimbursed for any reasonable travel, accommodation, parking, meals and other expenses associated with the research project visit, this will based on the distance that you have to travel to the hospital.

7. WHAT ARE THE RIGHTS OF PARTICIPANTS IN THIS STUDY?

a. Participation is voluntary

Participation in any research project is voluntary. If you do not wish to take part, you do not have to. If you decide to take part and later change your mind, you are free to withdraw from the project at

any stage. This will not affect your routine treatment, your relationship with those treating you or with <<Insert Site Name>> .
If you do decide to take part, you will be given this Participant Information and Consent Form to sign and you will be given a copy to keep.

b. New information
Sometimes during the course of a research project, new information becomes available about the treatment that is being studied. If this happens, your study doctor will tell you about it and discuss with you whether you want to continue in the research project. If you decide to withdraw, your study doctor will make arrangements for your regular health care to continue. If you decide to continue in the research project you may be asked to sign an updated consent form.

Also, on receiving new information, your study doctor might consider it to be in your best interests to withdraw you from the research project. If this happens, he/ she will explain the reasons and arrange for your regular health care to continue.

c. Right to access information collected during the study

In accordance with relevant <<Insert Country Name>> privacy and other relevant laws, you have the right to request access to your information collected and stored by the research team. You also have the right to request that any information with which you disagree be corrected. Please contact the study team member named at the end of this document if you would like to access your information.

8. CONFIDENTIALITY

By signing the consent form you consent to the study doctor and relevant research staff collecting and using personal information about you for the research project. Any information obtained in connection with this research project that can identify you will remain confidential. Your name, address or phone number will not be on the study forms; instead you will be identified by a unique participant study number, date of birth and initials. Your information will only be used for the purpose of this research project and it will only be disclosed with your permission, except as required by law.

Information about you may be obtained from your health records held at this and other health services for the purpose of this research. By signing the consent form you agree to the study team accessing health records if they are relevant to your participation in this research project.

Your health records and any information obtained during the research project are subject to inspection (for the purpose of verifying the procedures and the data) by the relevant authorities (such as the Ethics Committee that has reviewed and approved this study) and authorised representatives of the Sponsor, Apellis, and <<Insert Site Name>> or as required by law. All personnel reviewing your medical records will be required to keep the information confidential in accordance with law. By signing the Consent Form, you authorise release of, or access to, this confidential information to the relevant study personnel and regulatory authorities as noted above.

When the research project ends the study data must be analysed, so the results of the study may not be available until about six months after the research finishes. The study doctors and/or Apellis may decide to discuss or publish the results of the study. This may include publication in journals, presentation at conferences or other professional forums. In any publication, information will be provided in such a way that you cannot be identified. Results of the study will be provided to you, if you wish.

Information about your participation in this research project may be recorded in your health records.

Any information obtained for the purpose of this research project that can identify you will be treated as confidential and securely stored. It will be disclosed only with your permission, or as required by law.

Upon completion of the trial, the study file containing information about you will be archived for a minimum of fifteen years.

9. COMPENSATION FOR INJURY

If you were injured as a result of treatment given as part of this study, compensation would be available from the study's sponsor, Apellis, in line with industry guidelines. We can give you a copy of these guidelines if you wish.

If you have private health or life insurance, you may wish to check with your insurer that taking part in this study won't affect your cover.

10. WHAT WILL HAPPEN AFTER THE STUDY ENDS, OR IF YOU PULL OUT?

If you decide to withdraw
Please notify a member of the research team before you withdraw. This notice will allow that person or the research supervisor to discuss any health risks or special requirements linked to withdrawing, such as follow up visits.

If you do withdraw your consent during the research project, information already collected will be retained to ensure that the results of the research project can be measured properly and to comply with law. You should be aware that data collected by the sponsor up to the time you withdraw will form part of the research project results. If you do not want them to do this, you must tell them before you join the research project.

Why the study might be unexpectedly stopped
This research project may be stopped unexpectedly for a variety of reasons. These may include reasons such as:
- Unacceptable side effects
- The drug being shown not to be effective
- The drug being shown to work and not need further testing.

Will the study treatment continue to be available after the study finished?

If it is found the drug does continue to have a beneficial effect in reducing haemolysis (breakdown of red blood cells), and no safety concerns are raised, the company may, at its sole discretion, elect to continue the drug therapy by offering a further extension to the study.

Where can you go for more information about the study, or to raise concerns or complaints?

If you have any questions, concerns or complaints about the study at any stage, you can contact:

<<Insert Name>>, lead study doctor
Phone: <<Insert Contact Number>>and ask operator to page or put you through to <<Insert Name>>
Research Coordinator: <<Insert Contact Number>>

If you want to talk to someone who isn't involved with the study, you can contact an independent health and disability advocate. Specialist advocates, are available here.

Phone:
Fax:
Email:

You can also contact the health and disability ethics committee that approved this study on:

Phone:
Email:

12. DO I HAVE TO DECIDE STRAIGHT AWAY?

No, you do not have to decide straight away. You should take some time to consider whether or not to continue your participation in the study. Study staff will let you know when they will need your decision. The following steps are useful in helping you reach a decision.

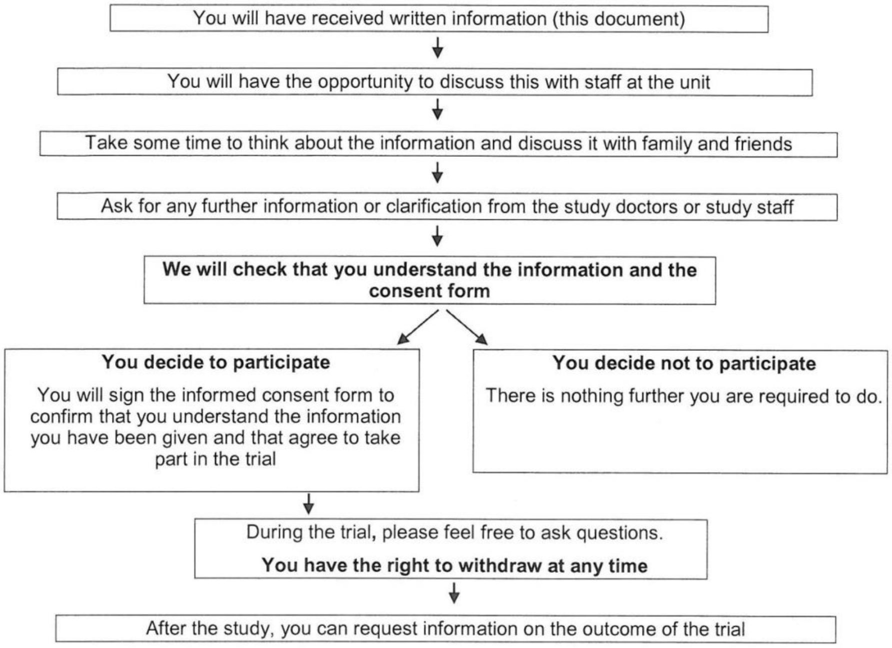

Insert Header with institution's name or institution's letterhead

INFORMED CONSENT FORM

**Short Title**	Nocturnal Hemoglobinuria (PNH) – PADDOCK
**Protocol Number**	APL2-CP-PNH-204
**Principal Investigator**	<<Insert Name>>

Declaration by participant:
I have read and I understand this version of the Participant Information Sheet. I have had the opportunity to ask questions and I am satisfied with the answers I have received.

I have had the opportunity to discuss the study with my family.

I freely agree to continue to participate in this study and understand that I may withdraw from the study at any time.

I understand that:

- My participation in this study is confidential and I will not be identified in study reports.
- My medical records may be reviewed by Apellis, companies working with them, auditors and health or regulatory agencies for the purpose of checking the accuracy of the information recorded for the study
- Some of my samples will be sent overseas for analysis and they will be destroyed as described in the Information Sheet.
- If I decide to withdraw from the study, information already collected may continue to be processed. I may be asked to attend follow-up visits.
- The study drugs or tests will be stopped if they appear harmful to me.
- I should contact site staff if I have any side effects.
- My GP and/or specialist will be informed of my participation in this study and relevant medical records may be accessed.
- I am not giving up any of my legal rights by signing this consent form.
- This trial is being carried out for the principal benefit of Apellis. If I suffer an injury as a result of my participation in the trial, they will pay me compensation in line with industry guidelines.
- I understand that there may be risks associated with the treatment in the event of myself or my partner becoming pregnant. I undertake to inform my partner of the risks and to take responsibility for the prevention of pregnancy

Statement by Participant I hereby consent to take part in this study. I understand that I will receive a signed copy of this consent form for my records.

_______________________________ (full name)

_______________________________ (signature)

__ __ / __ __ __ / __ __ (Date)

Statement by Consenter (Investigator/designee) I have discussed this study with the above named participant. The participant appeared to fully understand the information provided about the study.

_______________________________ (full name)

_______________________________ (signature)

__ __ / __ __ __ / __ __ (Date)

Version Date: January 24, 2018

INFORMED CONSENT

**TITLE:**	A Phase Ib, Open Label, Multiple Ascending Dose, Pilot Study to Assess the Safety, Preliminary Efficacy and Pharmacokinetics of Subcutaneously Administered APL-2 in Subjects with Paroxysmal Nocturnal Hemoglobinuria (PNH). – PADDOCK
**PROTOCOL NO.:**	APL2-CP-PNH-204
**SPONSOR:**	Apellis Pharmaceuticals, Inc.
**INVESTIGATOR:**	<*PI_First_Name*> <*PI_Last_Name*>
**TELEPHONE:**	<*Phone_Number*>

INTRODUCTION

This is a research study. It includes only people who choose to take part. Before agreeing to participate, it is important that you read and understand the following explanation of the proposed research study. This consent document describes the purpose, procedures, benefits, risks, discomforts and precautions of the study. It also describes the alternative procedures that are available to you and your right to withdraw from the study at any time. No guarantees or assurances can be made as to the results of the study.

The study is being conducted for Apellis Pharmaceuticals, Inc. Your study doctor is being paid by Apellis Pharmaceuticals, Inc. to conduct this study.

<*IRB_Name*> has approved the information in this consent document and has given approval for the study doctor to do the study. An institutional review board (IRB) is an independent committee established to help protect the rights of research subjects. This does not mean the IRB has approved your participation in the study. You must think about the information in this consent document for yourself. You must then decide if you want to be in the study. Be sure to ask questions about anything that you don't understand.

If you are not completely truthful with your study doctor regarding your health history, you may harm yourself by participating in this study.

BACKGROUND AND PURPOSE

This is a research study to test a new investigational drug. An investigational drug is one that is not approved by the United States Food and Drug Administration (FDA).

You are being asked to participate in the second cohort of this research study because you have been diagnosed with paroxysmal nocturnal hemoglobinuria (PNH). PNH is a rare disorder that causes the red blood cells to break down too early. Many PNH

patients suffer from complications such as blood clots, bleeding, or low red blood cell counts (anemia) even after receiving standard treatment.

The purpose of this research study is to gather scientific information about the safety and tolerability and continued evidence of activity of the investigational drug APL-2 in people with PNH who have not been treated with Soliris® in the past. The study will help determine how APL-2 behaves inside the body, how it is eliminated from the body, and the effect it has on the body after multiple doses and if it can prevent red blood cells from breaking down.

Because this is a research study, APL-2 will be given to you only during this study and not after the study is over.

NUMBER OF SUBJECTS / LENGTH OF PARTICIPATION

Cohort 1 has completed and 2 patients have already completed in excess of 169 days of dosing in Cohort 2. The study was initially set up in New Zealand but insufficient eligible PNH patients exist to complete the study.

Participation in the study involves 4 parts. Part 1 of this study will include the first 28 days of dosing and you will visit the study center at least 8 times.

If after this portion of the study there is evidence of clinical benefit, you may continue your participation into Part 2A which includes dosing from Day 29 through Day 84.

If it is determined that you're continuing to receive clinical benefit at the end of Part 2A, your doctor may recommend that you continue into Part 2B which includes dosing from Day 85 through Day 364. Part 2B will include at least 10 visits to the study center. If there is evidence that you are no longer getting clinical benefit, treatment will be stopped and you will skip Part 2B and enter Part 3 for safety follow-up visits

After completion of dosing, you will enter the Part 3 and return to the clinic for 3 follow-up visits at study days 365, 379 and 393 and exit procedures at day 414. Your participation for Parts 1, 2A, 2B and 3 in this study will last approximately 414 days and will include approximately 27 visits at the study center. In total, about 3 subjects are expected to participate in this study in the United States.

STUDY DRUG ADMINISTRATION

The study drug, APL-2, will be given as 1 or 2 bolus injection subcutaneously (under the skin). Each dose of study drug in Cohort 2 requires 2 injections if dose volume is <3 mL. If the dose volume is >3 mL the study drug will be administered as SC infusions.

You may self-administer the SC infusions via a syringe pump, after receiving appropriate training by a study nurse or other personnel. The preferred site for the injections is the abdomen however if you are unable to tolerate the injections in the abdomen the injections may be given in the thighs or upper arms.

You will receive 270 mg APL-2 each day for up to 364 days, although the study doctor may increase your dose up to 360 mg APL 2 each day if a higher dose is needed to reduce breakdown of your red blood cells

The safety monitoring committee will review all the safety data available from participants already dosed in the study approximately every 8 weeks.

The study can be stopped at any time, based on evaluation of the side effects of the study medication.

PROCEDURES

Before any study-related tests and procedures are performed, you will be asked to read and sign this consent document. The following tests and procedures will be performed to determine if you qualify to take part in this study:

Screening Visits

Visit Day -30 to Day -1

- Obtaining informed consent for participation in the study
- Review of your demographic (personal) information such as date of birth, ethnicity, and race
- Review of your medical history including history of transfusions and blood clots
- Review of your vaccination history
- Review of the medications you have been taking
- Your vital signs (temperature, resting pulse and blood pressure, respiratory rate) will be measured
- A physical examination including height and weight
- Electrocardiogram (ECG) – a test that records the electrical activity of your heart
- Collection of blood for safety lab tests, HIV and Hepatitis B and C. Positive HIV and hepatitis B and C results may be reportable to authorities according to the local law. Approximately 19 mL (about 4 teaspoons) of blood will be taken from one of your veins. The blood will be used to check for signs of general health and also for signs of disease or infection.
    - For women who are able to get pregnant, some of the blood will be used for a serum pregnancy test or hormone test for postmenopausal women. The result of the pregnancy test must be negative for you to participate in the study.
    - For women who are postmenopausal Follicle-Stimulating Hormone test will be performed
- Collection of a urine sample for routine lab tests

Study Center Visits

Each visit at the study center will include the following procedures:

- You will be asked what medications you are taking and if you have had any recent illnesses or changes in your condition.
- You will be questioned about your general health to assess for potential adverse events which might be related to the study medication.

- Your vital signs (temperature, resting pulse and blood pressure, respiratory rate) will be measured. If the visit includes study drug administration, vital signs will be measured before and after you receive the study drug. When study drug is given, the injection sites and the surrounding area will be checked for redness, swelling and hardness within 30 minutes after the injection. You will be asked about pain and tenderness.

Blood Collections:

A needle will be used to take blood samples from a vein in your arm about 24 times from Day 1 until the final exit visit, Day 414. The amount of blood that is taken each time will vary from approximately 2 mL (about ½ teaspoon) to approximately 25mL (about 1.5 tablespoons). The total blood taken for the study will be approximately 578mL (about 39 tablespoons). If the dose of study drug is increased during part 2B the total blood taken for the study will increase to a maximum of 623mL (about 42 tablespoons). Some of this blood will be used for routine safety tests and some will be used for research tests, such as measuring the amount of study drug in your blood.

Part 1: Site Visits - Day 1 - Day 28

- Review the entry criteria for the study to ensure that you remain eligible to participate (only ay visit 3)
- You will be given a prescription for an antibiotic to prevent infections, with instructions on when and how to take it. You will be asked to take the antibiotic, Ciprofloxacin until Day 14. On Day 15 you will stop Ciprofloxacin and start taking Penicillin V until 14 days after your last dose of the study drug. If you have an allergy to penicillin you must inform your doctor as this information will allow him or her to select the most appropriate antibiotic therapy for you.
- Electrocardiogram (ECG) – a test that records the electrical activity of your heart (Days 1, 3, 8, 15 and 22)
- Your vital signs (temperature, resting pulse and blood pressure, respiratory rate) will be measured. If the visit includes study drug administration, vital signs will be measured before and after you receive the study drug.
- Collection of blood – for this visit, blood samples will be taken before and after you receive study drug (Days 1, 8, 15 and 22)
- Collection of a urine sample for routine lab tests (Days 1, 8, 15 and 22)
  - For women who are able to get pregnant, some of this urine will be used for a pregnancy test. The result of the pregnancy test must be negative in order for you to receive the study drug
- You will be asked to complete a Fatigue Scale questionnaire which you will need to complete during clinic visits on Days 1 and 15
- You will receive the first 3 daily doses of APL-2 (Day 1 to 3 or beyond. You will continue to attend the hospital clinic on a daily basis until you are competent and confident to use the infusion pump to self-administer the study drug at home), as well as doses on Days 8, 15 and 22 at the hospital clinic. The injections will be given in your abdomen and you will be asked to remain at the study site for at least 2.5 hours after the study drug is given

- You will be vaccinated against Meningococcus (2 injections), Streptococcus pneumoniae (1 injections) and Haemophilus influenza (1 injection); unless you can provide evidence of having had these vaccinations within 2 years prior to Day 1 dosing on Day 15.
- You will receive study drug at Days 1, 2, 3, 8, 15 and 22

Part 2A: Site Visits - Day 29 – Day 84

- You will receive study drug on Day 29, 36, 43, 57 and 71 at the hospital clinic. You will self-administer the study drug on a daily basis on the following study days 30-35, 37-42, 44-56, 58-70 and 72-84.
- You will be asked to take Preventative Antibiotics
- A physical examination including height and weight on Days 29 and 57 (visit 31 and 59)
- Electrocardiogram (ECG) – a test that records the electrical activity of your heart on Days 29, 36, 43, 57 and 71
- Your vital signs (temperature, resting pulse and blood pressure, respiratory rate) will be measured. If the visit includes study drug administration, vital signs will be measured before and after you receive the study drug.
- Collection of blood – for this visit, blood samples will be taken before you receive study drug on Days 29, 36, 43, 57 and 71
- Collection of a urine sample for routine lab tests on Days 29, 36, 43, 57 and 71
    - For women who are able to get pregnant, some of this urine will be used for a pregnancy test. The result of the pregnancy test must be negative in order for you to receive the study drug
- You will be asked to complete a Fatigue Scale questionnaire during clinic visits on Days 29, 43 and 71

Part 2B: Site Visits 87 - 366 (Day 85 – Day 364)

- You will receive study drug on Days 85, 113, 141, 169, 197, 225, 253, 281, 309 and 337 at the hospital clinic. You will self-administer the study drug on a daily basis on all other days in between your hospital clinic visits
- If you received meningococcus and streptococcus pneumoniae vaccinations, you may receive booster shots for these vaccines on Day 85
- You will be asked to take Preventative Antibiotics
- A physical examination on Days 85, 253 and 337
- Electrocardiogram (ECG) – a test that records the electrical activity of your heart on Days 85, 113, 141, 169, 197, 225, 253, 281, 309, and 337
- Your vital signs (temperature, resting pulse and blood pressure, respiratory rate) will be measured. If the visit includes study drug administration, vital signs will be measured before and after you receive the study drug
- Collection of blood – for this visit, blood samples will be taken before you receive study drug Day 85, 113, 141, 169, 197, 225, 253, 281, 309 and 337
- Collection of a urine sample for routine lab tests Day 85, 113, 141, 169, 197, 225, 253, 281, 309 and 337

  - For women who are able to get pregnant, some of this urine will be used for a pregnancy test. The result of the pregnancy test must be negative in order for you to receive the study drug
- You will be asked to complete a Fatigue Scale questionnaire during clinic visits on Days 85,141,197, 253 and 309

Home visits will be conducted by a trained study nurse until you or your caregiver begin use of the self-administration syringe pump.

- You will be asked what medications you are taking and if you have had any illnesses or changes in your condition
- Your vital signs (temperature, resting pulse and blood pressure, respiratory rate) will be measured before and after you receive the study drug
- You will receive a dose of the study drug
- The injection sites where the study drug was given will be assessed and the surrounding areas will be checked for redness, swelling and hardness within 30 minutes after the injection. You will be asked about pain and tenderness.
- You will be questioned about your general health to assess for potential adverse events which might be related to the study medication.

You or your caregiver may self-administer the SC infusions via a syringe pump, after receiving appropriate training by a study nurse or other trained study staff. ~~You will be requested to complete a Record of Administration log for the daily study drug administration.~~ Once you begin use of the self-administration pump, the study nurses will continue to provide assistance as needed until you are competent and comfortable with the use of the syringe pump. The study nurses will no longer perform the assessments noted above once the use of the self-administration pump begins. The visits by the study nurses will cease once you have demonstrated you or your caregiver are competent and comfortable with the use of the syringe pump.

If you or your caregiver administer the daily doses of the study drug, you will need to report the following to the clinical site:

- Any potential adverse events which might be related to the study medication

- Any medications you might be taking and if you have had any recent illnesses or changes in your condition

Follow-up and Exit Visits

Three follow up visits will be conducted at the study center; Day 365, 379 and 393. Day 414 will be the final study exit visit. You will not receive any study drug at these visits. These visits will include the following tests and procedures:

- You will be asked what medications you are taking and if you have had any illnesses or changes in your condition
- Your vital signs (temperature, resting pulse and blood pressure, respiratory rate) will be measured

Version Date: January 24, 2018

- You will be questioned about your general health to assess for potential adverse events which might be related to the study medication.
- Electrocardiogram (ECG) – Day 365
- Physical examination – Day 365 and 414
- Collection of a urine sample for routine lab tests
  - For women who are able to get pregnant, some of this urine will be used for a pregnancy test at Day 365, 393 and 414
- Collection of blood. Some of your blood will be used to measure the amount of study drug in your blood.
- You will be asked to complete a Fatigue Scale questionnaire at Day 365, 393 and 414

Unscheduled Visits

You may be asked to return to the study center for additional follow up visits if your study doctor or the study Sponsor feels it is necessary. These unscheduled visits may include any of the study procedures described in this consent document. If the dose is increased to >270 mg/day, you may need to attend the clinical site for safety visits every 2 weeks (instead of every 4 weeks) for the first 6 weeks after the dose increase i.e. up to an additional 3 clinic visits.

You will receive study drug at an off-site location convenient to you, with the exception of the Follow-up and Exit Visits. Study drug is not administered at the Follow-up and Exit Visits.

EXPECTATIONS

If you participate in this study, you will be expected to:

- Attend each visit at the study center
- Self-administer the study drug per the instructions provided
- Follow the instructions given by the study doctor
- Take an antibiotic and antiviral throughout the study to prevent infections
- Have all procedures done to you
- Notify the study doctor if you experience any health problems or illnesses during the study

RISKS, SIDE EFFECTS AND/OR DISCOMFORTS

You may have side effects while taking this drug. You may feel discomfort during some of the tests and study procedures. Possible risks, side effects, and discomforts include:

- Infections – you may be at increased risk for infections caused by certain types of organisms such as *Streptococcus pneumonia, Neisseria Meningitidis,* or *Haemophilus influenza*
- Allergic reactions – all drugs have a potential risk of an allergic reaction, which if not treated promptly, could become life-threatening

- Blood samples - you may have discomfort or pain when your blood is collected. You may feel faint or pass out. There is also a slight possibility of infection, bruising, or bleeding at the puncture site. Your anemia (low red blood cell count) could worsen from having frequent blood draws.
- ECG - skin irritation is rare but could occur during an ECG from the electrodes or gel that is used
- Antibiotic/Antiviral therapy – there is a possibility that you will experience some side effects from the antibiotic treatment you will need to take during this study. Side effects such as nausea, vomiting, diarrhea, skin rashes and hives/welts may occur and if you experience any of these you should inform the nurse or doctor. The doctor may decide that you should change to a different antibiotic/antiviral to try and reduce these symptoms.
- Injection site reactions - there is a possibility that you could experience redness, swelling, and pain or tenderness at the site of injection. There is also a slight possibility of infection.
- Vaccination - Like any medication, vaccines, can cause side effects. The side effects associated with getting vaccines are almost always mild (such as redness and swelling where the shot was given) and usually go away within a few days.

UNFORESEEN RISKS

Since the study drug, APL-2 is investigational, when taken alone or in combination with other medications, there may be other risks that are unknown.

PREGNANCY / BIRTH CONTROL

Taking the study drug may involve unknown risks to a pregnant woman, an embryo, fetus (unborn baby) or nursing infant. Therefore, if you are pregnant, planning to become pregnant or are breastfeeding a child, you cannot participate in this study.

In order to reduce the risk of pregnancy, you must use an effective method of birth control while you are participating in this study. If you are already using a method of birth control, the study doctor or study staff will discuss with you whether your current method of birth control is acceptable for use during this study. The acceptable methods of birth control include: abstinence, surgical sterilization, oral contraceptives, intrauterine device, condoms with spermicidal jelly or foam, diaphragms in combination with spermicidal jelly or foam, implantable or injectable contraceptives like Norplant or DepoProvera, removable birth control devices like NuvaRing or Evra patches.

If, during this study, you become pregnant, you should notify the study doctor as soon as possible. The study drug will be stopped and your participation in this study will be ended. You will be asked to provide information about your pregnancy and its outcome and this information will be provided to Apellis Pharmaceuticals.

Males

Exposure to the study drug may involve unknown risks to a pregnant woman, an embryo, or a fetus (unborn baby). If you are having sexual intercourse with a woman who can become pregnant, you must use an acceptable form of birth control while you

are participating in this study. Acceptable methods of birth control for use in this study are listed in the **Pregnancy/Birth Control** section above. You must also agree to avoid donating sperm while you are participating in this study.

If you agree to participate in this study, you are expected to inform your female sexual partner(s) that you are participating in a research study of a drug, and that the effects of the drug on an unborn baby and on a pregnant woman are unknown. You are also expected to provide your female sexual partner(s) with the information in the **Pregnancy/Birth Control** section of this Informed Consent and to provide her with contact information for the study doctor for any additional questions.

If your female partner becomes pregnant while you are participating in this study, tell your study doctor or study nurse immediately. At that time the study doctor may seek the pregnant woman's permission to review her medical records and the infant's medical records after delivery, if applicable. The study doctor will share the information about your pregnant partner and the baby with the study sponsor to help understand the effects, if any, that the study drug may have on the pregnancy and the child.

Payment for all aspects of obstetrical care, child-or related care will be your responsibility.

ALTERNATIVES TO PARTICIPATION

You do not have to be in this study to receive treatment for your PNH*.* You have the option to receive standard treatment for PNH. Standard treatment may include medications such as Soliris®, prednisone, or blood thinners; blood transfusions; or bone marrow transplantation. The study doctor will discuss with you the risks and benefits of the alternative treatments.

NEW FINDINGS

Any new important information that is discovered during the study, either from this study or from other studies which are ongoing in both patients and healthy volunteers, and which may influence your willingness to continue participation in the study, will be made available to you.

BENEFITS

You may or may not benefit from participation in this study. There is no guarantee that you will benefit from participating in this study. Information learned from this study may benefit others in the future.

COMPENSATION FOR PARTICIPATION

You will not be paid to participate in the research study, however, a stipend of $ ____ may be paid to you to cover any reasonable travel, accommodation, parking, meals and other expenses associated with the visit, this will based on the distance that you have to travel to the hospital. If you withdraw or are removed from the study, you will receive the stipend for the visits you have completed.

Version Date: January 24, 2018

CONFIDENTIALITY

Records of your participation in this study will be held confidential except when sharing the information is required by law or as described in this informed consent. The study doctor, the sponsor or persons working on behalf of the sponsor, and under certain circumstances, the United States Food and Drug Administration (FDA), Safety Monitoring Committee and the Institutional Review Board (IRB) will be able to inspect and copy confidential study-related records which identify you by name. This means that absolute confidentiality cannot be guaranteed. If the results of this study are published or presented at meetings, you will not be identified.

A description of this clinical trial will be available on http://www.ClinicalTrials.gov, as required by U.S. Law. This web site will not include information that can identify you. At most, the web site will include a summary of the results. You can search this web site at any time.

COMPENSATION FOR INJURY

If you are injured as a result of taking the study drug(s) or from procedures done for the purpose of this study, the sponsor will pay for those medical expenses necessary to treat your injury that are not covered by your medical insurance or any other third party coverage. There are no plans to provide other compensation beyond that which is listed in this informed consent document. You will not lose any of your legal rights or release the Sponsor, the study doctor, the study staff, or study site from liability for mistakes or intentional misconduct by signing this consent document.

If you are injured during this study, your study doctor will discuss with you the available medical treatment options.

COSTS

There will be no charge to you for your participation in this study. The study drug, study-related procedures, and study visits will be provided at no charge to you or your insurance company.

EMERGENCY CONTACT / IRB CONTACT

During the study, if you experience any medical problems, suffer a research-related injury, or have questions, concerns or complaints about the study, please contact the study doctor at the telephone number listed on page one of this consent document. If you seek emergency care, or hospitalization is required, alert the treating physician that you are participating in a research study being conducted by the study doctor listed on page one of this document.

If you have any questions about your rights as a research subject, and/or concerns or complaints regarding this research study, you should write to <insert IRB contact info>

Version Date: January 24, 2018

VOLUNTARY PARTICIPATION / WITHDRAWAL

Your decision to participate in this study is voluntary. You may choose to not participate or you may withdraw from the study for any reason without penalty or loss of benefits to which you are otherwise entitled and without any effect on your future medical care.

The study doctor or the sponsor can stop your participation at any time without your consent for the following reasons:

- If it appears to be medically harmful to you;
- If you fail to follow directions for participating in the study;
- If it is discovered that you do not meet the study requirements; or
- If the study is canceled.

If you leave the study for any reason, the study doctor may ask you to have some end-of-study tests for your safety.

PRIMARY CARE PHYSICIAN / SPECIALIST NOTIFICATION OPTION

Please indicate below whether you want us to notify your primary care physician or your specialist of your participation in this study.

________ Yes, I want the study doctor to inform my primary care physician/specialist of my participation in this study.

________ No, I do not want the study doctor to inform my primary care physician/specialist of my participation in this study.

________ I do not have a primary care physician/specialist.

________ The study doctor is my primary care physician/specialist.

Version Date: January 24, 2018

CONSENT

I have read and understand the information in this informed consent document. I have had an opportunity to ask questions and all of my questions have been answered to my satisfaction. I voluntarily agree to participate in this study until I decide otherwise. I do not give up any of my legal rights by signing this consent document. I will receive a copy of this signed consent document.

For male subjects:

I will advise my sexual partner(s) that I am participating in a research study of a drug and that the effects of the drug on an unborn baby and on a pregnant woman are unknown. I will provide my female sexual partner(s) with the information in the Pregnancy/Birth Control section of this Informed Consent and will provide her with contact information for the study doctor for any additional questions.

____

Subject's Printed Name

____________________________________ _________

Subject's Signature **Date**

____

Printed Name of the Person Conducting the Consent Discussion

_____________________________________ ___________

Signature of the Person Conducting the Consent Discussion Date

Version Date: January 24, 2018

CONSENT FOR SUBJECTS WHO CANNOT READ

The study subject has indicated that he/she is unable to read. The consent document has been read to the subject by a member of the study staff, discussed with the subject by a member of the study staff, and the subject has been given an opportunity to ask questions of the study staff.

____

Printed Name of Impartial Witness

____________________________________ __________

Signature of Impartial Witness* Date

*Impartial Witness: A person, who is independent of the trial, who cannot be unfairly influenced by people involved with the trial, who attends the informed consent process if the subject or the subject's legally acceptable representative cannot read, and who reads the informed consent and any other written information supplied to the subject. **Guidance for Industry E6 Good Clinical Practice: Consolidated Guidance.**

Version Date: January 24, 2018

AUTHORIZATION TO USE AND DISCLOSE PROTECTED HEALTH INFORMATION

During your participation in this research study, the study doctor and study staff will collect or create personal health information about you (for example, medical histories and results of any tests, examinations or procedures you undergo while in the study) and record it on study documents. The study doctor will keep this personal health information in your study-related records (that we will refer to as "your study records"). In addition, the study doctor may obtain, and include in your records, information regarding your past, present and/or future physical or mental health and/or condition. Your study doctor may ask you to sign a separate authorization to obtain some or all of your medical records from your doctor. Your study records may include other personal information (such as social security number, medical record numbers, date of birth, etc.), which could be used to identify you. Health information that could identify you is called "Protected Health Information" (or "PHI").

Under federal law (the "Privacy Rule"), your PHI that is created or obtained during this research study cannot be "used" to conduct the research or "disclosed" (given to anyone) for research purposes without your permission. This permission is called an "Authorization". Therefore, you may not participate in this study unless you give your permission to use and disclose your PHI by signing this Authorization. By signing, you are agreeing to allow the study doctor and staff to use your PHI to conduct this study.

By signing this Authorization, you also are agreeing to allow the study doctor to disclose PHI as described below:

- The sponsor of this study and anyone working on behalf of the sponsor to conduct this study (referred to as "the sponsor"). The sponsor will analyze and evaluate the PHI and may use it to develop new tests, procedures and commercial products. The study staff will assign a code number and/or letters to your records, which means that you will not ordinarily be identified in the records sent to the sponsor. The sponsor may, however, look at your complete study records that identify you. In addition, the sponsor may visit the study site to oversee the way the study is being conducted and may review your PHI during these visits to make sure the information is correct.
- The Institutional Review Board ("IRB") may have access to your PHI in relation to its responsibilities as an Institutional Review Board.
- Safety Monitoring Committee

The study doctor or sponsor may disclose your PHI to the United States Food and Drug Administration ("FDA") or similar regulatory agencies in the United States and/or foreign countries.

These disclosures also help ensure that the information related to the research is available to all parties who may need it for research purposes.

Except for the disclosures described above, your PHI will not be shared with others unless required by law. If your PHI is given to the parties listed above and/or to others who are not required to comply with the federal law, your PHI will no longer be protected by this law and could possibly be used or disclosed in ways other than those listed here.

Version Date: January 24, 2018

You have a right to see and make copies of your PHI. You are agreeing, however, by signing this document, not to see or copy some or all of your PHI until the sponsor has completed all work related to this study. At that time, you may ask to see your records.

This Authorization will expire 50 years from the date you sign it unless you revoke (cancel or withdraw) it sooner.

You have a right to revoke your Authorization at any time. If you revoke it, your PHI will no longer be used for this study, except to the extent the parties to the research have already taken action based upon your Authorization or need the information to complete analysis and reports for this research. To revoke your Authorization, you must write to the study doctor, stating that you are revoking your Authorization to Use and Disclose Protected Health Information. If you revoke this Authorization, you will not be allowed to continue to be in this study.

____

Printed Name of Subject

____________________________________ __________

Signature of Subject Date

____

Printed Name of the Person Obtaining the Authorization

____________________________________ __________

Signature of the Person Obtaining the Authorization Date

Version Date: January 24, 2018

FOR SUBJECTS WHO CANNOT READ

The study subject has indicated that he/she is unable to read. This Authorization document has been read to the subject by a member of the study staff, discussed with the subject by a member of the study staff, and the subject has been given an opportunity to ask questions of the study staff.

____

Printed Name of Impartial Witness

____ ____________

Signature of Impartial Witness* Date

*Impartial Witness: A person, who is independent of the trial, who cannot be unfairly influenced by people involved with the trial, who attends the informed consent process if the subject or the subject's legally acceptable representative cannot read, and who reads the informed consent and any other written information supplied to the subject. **Guidance for Industry E6 Good Clinical Practice: Consolidated Guidance.**

Version Date: August 23, 2018

NOT FOR CONSENTING SUBJECTS
DRAFT 1 08/23/2018 JA

INFORMED CONSENT

**TITLE:**	A Phase Ib, Open Label, Multiple Ascending Dose, Pilot Study to Assess the Safety, Preliminary Efficacy and Pharmacokinetics of Subcutaneously Administered APL-2 in Subjects with Paroxysmal Nocturnal Hemoglobinuria (PNH). – PADDOCK
**PROTOCOL NO.:**	APL2-CP-PNH-204
**SPONSOR:**	Apellis Pharmaceuticals, Inc.
**INVESTIGATOR:**	«PI_FIRST_NAME» «PI_LAST_NAME»
**TELEPHONE:**	«PHONE_NUMBER»

INTRODUCTION

This is a research study. It includes only people who choose to take part. Before agreeing to participate, it is important that you read and understand the following explanation of the proposed research study. This consent document describes the purpose, procedures, benefits, risks, discomforts and precautions of the study. It also describes the alternative procedures that are available to you and your right to withdraw from the study at any time. No guarantees or assurances can be made as to the results of the study.

The study is being conducted for Apellis Pharmaceuticals, Inc. Your study doctor is being paid by Apellis Pharmaceuticals, Inc. to conduct this study.

Schulman IRB has approved the information in this consent document and has given approval for the study doctor to do the study. An institutional review board (IRB) is an independent committee established to help protect the rights of research subjects. This does not mean the IRB has approved your participation in the study. You must think about the information in this consent document for yourself. You must then decide if you want to be in the study. Be sure to ask questions about anything that you don't understand.

If you are not completely truthful with your study doctor regarding your health history, you may harm yourself by participating in this study.

BACKGROUND AND PURPOSE

This is a research study to test a new investigational drug. An investigational drug is one that is not approved by the United States Food and Drug Administration (FDA).

You are being asked to participate in the second cohort of this research study because you have been diagnosed with paroxysmal nocturnal hemoglobinuria (PNH). PNH is a rare disorder that causes the red blood cells to break down too early**.** Many PNH

patients suffer from complications such as blood clots, bleeding, or low red blood cell counts (anemia) even after receiving standard treatment.

The purpose of this research study is to gather scientific information about the safety and tolerability and continued evidence of activity of the investigational drug APL-2 in people with PNH who have not been treated with Eculizumab (Soliris®) in the past. The study will help determine how APL-2 behaves inside the body, how it is eliminated from the body, and the effect it has on the body after multiple doses and if it can prevent red blood cells from breaking down.

Because this is a research study, APL-2 will be given to you only during this study and not after the study is over.

NUMBER OF SUBJECTS / LENGTH OF PARTICIPATION

Cohort 1 has completed and 2 subjects have already completed in excess of 169 days of dosing in Cohort 2.

Participation in the study involves 3 parts. Part 1 of this study will include the first 28 days of dosing and you will visit the study center at least 8 times.

If after this portion of the study there is evidence of clinical benefit, you may continue your participation into Part 2A which includes dosing from Day 29 through Day 84.

If it is determined that you're continuing to receive clinical benefit at the end of Part 2A, your doctor may recommend that you continue into Part 2B which includes dosing from Day 85 through Day 364. Part 2B will include at least 10 visits to the study center. If there is evidence that you are no longer getting clinical benefit, study treatment will be stopped and you will skip Part 2B and enter Part 3 for safety follow-up visits

After completion of dosing, you will enter the Part 3 and return to the clinic for 3 follow-up visits at study days 365, 379 and 393 and exit procedures at day 414. Your participation for Parts 1, 2A, 2B and 3 in this study will last approximately 414 days and will include approximately 27 visits at the study center. In total, about 3 subjects are expected to participate in this study in the United States.

STUDY DRUG ADMINISTRATION

If the study drug, APL-2, dose volume is <3 mL it will be given as 1 or 2 injections subcutaneously (under the skin). If the dose volume is >3 mL the study drug will be administered as subcutaneous (SC) infusions.

You may self-administer the SC infusions via a syringe pump, after receiving appropriate training by a study nurse or other personnel. The preferred site for the injections is the abdomen however if you are unable to tolerate the injections in the abdomen the injections may be given in the thighs or upper arms.

You will receive 270 mg APL-2 each day for up to 364 days, although the study doctor may increase your dose up to 360 mg APL 2 each day if a higher dose is needed to reduce breakdown of your red blood cells

The safety monitoring committee will review all the safety data available from participants already dosed in the study approximately every 8 weeks.

The study can be stopped at any time, based on evaluation of the side effects of the study medication.

PROCEDURES

Before any study-related tests and procedures are performed, you will be asked to read and sign this consent document. The following tests and procedures will be performed to determine if you qualify to take part in this study:

Screening Visits

Visit Day -30 to Day -1

- Obtaining informed consent for participation in the study
- Review of your demographic (personal) information such as date of birth, ethnicity, and race
- Review of your medical history including history of transfusions and blood clots
- Review of your vaccination history
- Review of the medications you have been taking
- Your vital signs (temperature, resting pulse and blood pressure, respiratory rate) will be measured
- A physical examination including height and weight
- Electrocardiogram (ECG) – a test that records the electrical activity of your heart
- Collection of blood for safety lab tests, HIV and Hepatitis B and C. Positive HIV and hepatitis B and C results may be reportable to authorities according to the local law. If the hepatitis B test is positive, antiviral therapy can be given and a negative hepatitis B DNA test result must occur prior to study drug administration and continue until 2 weeks after the final dose of study drug. Approximately 19 mL (about 4 teaspoons) of blood will be taken from one of your veins. The blood will be used to check for signs of general health and also for signs of disease or infection.
    - For women who are able to get pregnant, some of the blood will be used for a serum pregnancy test. The result of the pregnancy test must be negative for you to participate in the study.
    - For women who are postmenopausal Follicle-Stimulating Hormone test will be performed
- Collection of a urine sample for routine lab tests

Study Center Visits

Each visit at the study center will include the following procedures:

- You will be asked what medications you are taking and if you have had any recent illnesses or changes in your condition.

NOT FOR CONSENTING SUBJECTS
DRAFT 1 08/23/2018 JA

Version Date: August 23, 2018

- You will be asked questions about your general health to assess for potential adverse events which might be related to the study medication.
- Your vital signs (temperature, resting pulse and blood pressure, respiratory rate) will be measured. If the visit includes study drug administration, vital signs will be measured before and after you receive the study drug. If the study drug is self-administered, pre and post-dose vital signs will not be measured.
- When study drug is given, the injection sites and the surrounding area will be checked for redness, swelling and hardness within 30 minutes after the injection. You will be asked about pain and tenderness.

Blood Collections:

A needle will be used to take blood samples from a vein in your arm about 24 times from Day 1 until the final exit visit, Day 414. The amount of blood that is taken each time will vary from approximately 2 mL (about ½ teaspoon) to approximately 25mL (about 1.5 tablespoons). The total blood taken for the study will be approximately 578mL (about 39 tablespoons). If the dose of study drug is increased during part 2B the total blood taken for the study will increase to a maximum of 623mL (about 42 tablespoons). For comparison, a standard blood donation at a blood collection center, once in any 56-day period, is about 32 tablespoons of blood. Some of this blood will be used for routine safety tests and some will be used for research tests, such as measuring the amount of study drug in your blood.

Part 1: Site Visits - Day 1 - Day 28

- Review the entry criteria for the study to ensure that you remain eligible to participate (only at visit 1)
- You will be given a prescription for an antibiotic to prevent infections, with instructions on when and how to take it. You will be asked to take the antibiotic, Ciprofloxacin (500 mg) until Day 14. On Day 15 you will stop Ciprofloxacin and start taking Penicillin V (500 mg) until 14 days after your last dose of the study drug. If you have an allergy to penicillin you must inform your doctor as this information will allow him or her to select the most appropriate antibiotic therapy for you.
- Electrocardiogram (ECG) – a test that records the electrical activity of your heart (Days 1, 3, 8, 15 and 22)
- Collection of blood –blood samples will be taken before and after you receive study drug (Days 1, 8, 15 and 22)
- Collection of a urine sample for routine lab tests (Days 1, 8, 15 and 22)
  - For women who are able to get pregnant, some of this urine will be used for a pregnancy test. The result of the pregnancy test must be negative in order for you to receive the study drug
- You will be asked to complete a Fatigue Scale questionnaire which you will need to complete during clinic visits on Days 1 and 15
- You will receive daily doses of APL-2. On Days 1, 2, 3, 8, 15, and 22 the dose will be administered by the study staff at the study site. The injections will be given in your abdomen and you will be asked to remain at the study site for at least 2.5 hours after the study drug is given. You will self-administer the study

drug on a daily basis on all other days in between your hospital clinic visits. However, home visits will be conducted by a trained study nurse on a daily basis until you are competent and confident to use the infusion pump to self-administer the study drug at home.

- You will be vaccinated against Meningococcus (2 injections), Streptococcus pneumoniae (1 injections) and Haemophilus influenza (1 injection); unless you can provide evidence of having had these vaccinations prior to Day 1 (Day 15)

Part 2A: Site Visits - Day 29 – Day 84

- You will receive study drug on Day 29, 36, 43, 57 and 71 at the hospital clinic. You will self-administer the study drug on a daily basis on the following study days 30-35, 37-42, 44-56, 58-70 and 72-84.
- You will be asked to take Preventative Antibiotics daily
- A physical examination including height and weight on Days 29 and 57
- Electrocardiogram (ECG) – a test that records the electrical activity of your heart on Days 29, 36, 43, 57 and 71
- Collection of blood – for this visit, blood samples will be taken before and after you receive study drug on Days 29, 36, 43, 57 and 71
- Collection of a urine sample for routine lab tests on Days 29, 36, 43, 57 and 71
  - For women who are able to get pregnant, some of this urine will be used for a pregnancy test. The result of the pregnancy test must be negative in order for you to receive the study drug
- You will be asked to complete a Fatigue Scale questionnaire during clinic visits on Days 29, 43 and 71

Part 2B: Site Visits 87 - 366 (Day 85 – Day 364)

- You will receive study drug on Days 85, 113, 141, 169, 197, 225, 253, 281, 309 and 337 at the hospital clinic. You will self-administer the study drug on a daily basis on all other days in between your hospital clinic visits
- If you received meningococcus and streptococcus pneumoniae vaccinations, you may receive booster shots for these vaccines on Day 85
- You will be asked to take Preventative Antibiotics daily
- You will have a physical examination on Days 85, 253 and 337
- Electrocardiogram (ECG) – a test that records the electrical activity of your heart on Days 85, 113, 141, 169, 197, 225, 253, 281, 309, and 337
- Collection of blood –, blood samples will be taken before and after you receive study drug Day 85, 113, 141, 169, 197, 225, 253, 281, 309 and 337
- Collection of a urine sample for routine lab tests Day 85, 113, 141, 169, 197, 225, 253, 281, 309 and 337
  - For women who are able to get pregnant, some of this urine will be used for a pregnancy test. The result of the pregnancy test must be negative in order for you to receive the study drug
- You will be asked to complete a Fatigue Scale questionnaire during clinic visits on Days 85,141,197, 253 and 309

Home visits will be conducted by a trained study nurse until you begin use of the self-administration syringe pump.

- You will be asked what medications you are taking and if you have had any illnesses or changes in your condition
- Your vital signs (temperature, resting pulse and blood pressure, respiratory rate) will be measured before and after you receive the study drug
- You will receive a dose of the study drug
- The injection sites where the study drug was given will be assessed and the surrounding areas will be checked for redness, swelling and hardness within 30 minutes after the injection. You will be asked about pain and tenderness.
- You will be questioned about your general health to assess for potential adverse events which might be related to the study medication.

You may self-administer the SC infusions via a syringe pump, after receiving appropriate training by a study nurse or other trained study staff. You will be requested to complete a Record of Administration log for the daily study drug administration. Once you begin use of the self-administration pump, the study nurses will continue to provide assistance as needed until you are competent and comfortable with the use of the syringe pump. The study nurses will no longer perform the assessments noted above once the use of the self-administration pump begins. The visits by the study nurses will cease once you have demonstrated you are competent and comfortable with the use of the syringe pump.

If you administer the daily doses of the study drug, you will need to report the following to the clinical site:

- Any potential adverse events which might be related to the study medication
- Any medications you might be taking and if you have had any recent illnesses or changes in your condition

Follow-up and Exit Visits

Three follow up visits will be conducted at the study center; Day 365, 379 and 393. Day 414 will be the final study exit visit. You will not receive any study drug at these visits. These visits will include the following tests and procedures:

- You will be asked what medications you are taking and if you have had any illnesses or changes in your condition
- Your vital signs (temperature, resting pulse and blood pressure, respiratory rate) will be measured
- You will be questioned about your general health to assess for potential adverse events which might be related to the study medication.
- Electrocardiogram (ECG) – Day 365
- Physical examination – Day 365 and 414
- Collection of a urine sample for routine lab tests

NOT FOR CONSENTING SUBJECTS
DRAFT 1 08/23/2018 JA

  - For women who are able to get pregnant, some of this urine will be used for a pregnancy test at Day 365, 393 and 414
- Collection of blood. Some of your blood will be used to measure the amount of study drug in your blood.
- You will be asked to complete a Fatigue Scale questionnaire at Day 365, 393 and 414
- You will stop taking the preventative antibiotics at visit 379

Unscheduled Visits

You may be asked to return to the study center for additional follow up visits if your study doctor or the study Sponsor feels it is necessary. These unscheduled visits may include any of the study procedures described in this consent document. If the dose is increased to >270 mg/day, you may need to attend the clinical site for safety visits every 2 weeks (instead of every 4 weeks) for the first 6 weeks after the dose increase i.e. up to an additional 3 clinic visits.

You will receive study drug at an off-site location convenient to you, with the exception of the Follow-up and Exit Visits. Study drug is not administered at the Follow-up and Exit Visits.

EXPECTATIONS

If you participate in this study, you will be expected to:

- Attend each visit at the study center
- Self-administer the study drug per the instructions provided
- Follow the instructions given by the study doctor
- Take an antibiotic and antiviral throughout the study to prevent infections
- Have all procedures done to you
- Notify the study doctor if you experience any health problems or illnesses during the study

RISKS, SIDE EFFECTS AND/OR DISCOMFORTS

You may have side effects while taking this drug. You may feel discomfort during some of the tests and study procedures. Possible risks, side effects, and discomforts include:

- Infections – you may be at increased risk for infections caused by certain types of organisms such as *Streptococcus pneumonia, Neisseria Meningitidis,* or *Haemophilus influenza*
- Allergic reactions – all drugs have a potential risk of an allergic reaction, which if not treated promptly, could become life-threatening
- Headache– in the ongoing and completed studies with APL-2 there have been some reports of headache which have been considered as possibly related to APL-2

NOT FOR CONSENTING SUBJECTS
DRAFT 1 08/23/2018 JA

Version Date: August 23, 2018

- Other events from ongoing and completed studies with APL-2 - there have been some sporadic reports of upper respiratory tract infection, urinary tract infection, hypersensitivity reaction, constipation, abdominal pain, and increased liver function tests. These events have all resolved whilst continuing treatment with APL-2 but have been considered to be possibly related to APL-2 by the investigators
- Blood samples - you may have discomfort or pain when your blood is collected. You may feel faint or pass out. There is also a slight possibility of infection, bruising, or bleeding at the puncture site. Your anemia (low red blood cell count) could worsen from having frequent blood draws.
- ECG - skin irritation is rare but could occur during an ECG from the electrodes or gel that is used
- Antibiotic/Antiviral therapy – there is a possibility that you will experience some side effects from the antibiotic/antiviral treatment you will need to take during this study. Side effects such as nausea, vomiting, diarrhea, skin rashes and hives/welts may occur and if you experience any of these you should inform the nurse or doctor. The doctor may decide that you should change to a different antibiotic/antiviral to try and reduce these symptoms.
- Injection site reactions - there is a possibility that you could experience redness, swelling, and pain or tenderness at the site of injection. There is also a slight possibility of infection.
- Vaccination - Like any medication, vaccines, can cause side effects. The side effects associated with getting vaccines are almost always mild (such as redness and swelling where the shot was given) and usually go away within a few days.

UNFORESEEN RISKS

Since the study drug, APL-2 is investigational, when taken alone or in combination with other medications, there may be other risks that are unknown.

PREGNANCY / BIRTH CONTROL

Taking the study drug may involve unknown risks to a pregnant woman, an embryo, fetus (unborn baby) or nursing infant. Therefore, if you are pregnant, planning to become pregnant or are breastfeeding a child, you cannot participate in this study.

In order to reduce the risk of pregnancy, you must use an effective method of birth control while you are participating in this study and for 3 months after the final dose of study drug. If you are already using a method of birth control, the study doctor or study staff will discuss with you whether your current method of birth control is acceptable for use during this study. The acceptable methods of birth control include: abstinence, surgical sterilization (at least 6 months before dosing), oral contraceptives, intrauterine device, condoms, diaphragms, implantable or injectable contraceptives like Norplant or DepoProvera, removable birth control devices like NuvaRing or Evra patches.

NOT FOR CONSENTING SUBJECTS
DRAFT 1 08/23/2018 JA

Version Date: August 23, 2018

If, during this study, you become pregnant, you should notify the study doctor as soon as possible. The study drug will be stopped and your participation in this study will be ended. You will be asked to provide information about your pregnancy and its outcome and this information will be provided to Apellis Pharmaceuticals.

Males

Exposure to the study drug may involve unknown risks to a pregnant woman, an embryo, or a fetus (unborn baby). If you are having sexual intercourse with a woman who can become pregnant, you must use an acceptable form of birth control while you are participating in this study. Acceptable methods of birth control for use in this study are listed in the **Pregnancy/Birth Control** section above. You must also agree to avoid donating sperm while you are participating in this study.

If you agree to participate in this study, you are expected to inform your female sexual partner(s) that you are participating in a research study of a drug, and that the effects of the drug on an unborn baby and on a pregnant woman are unknown. You are also expected to provide your female sexual partner(s) with the information in the **Pregnancy/Birth Control** section of this Informed Consent and to provide her with contact information for the study doctor for any additional questions.

If your female partner becomes pregnant while you are participating in this study, tell your study doctor or study nurse immediately. At that time the study doctor may seek the pregnant woman's permission to review her medical records and the infant's medical records after delivery, if applicable. The study doctor will share the information about your pregnant partner and the baby with the study sponsor to help understand the effects, if any, that the study drug may have on the pregnancy and the child.

Payment for all aspects of obstetrical care, child-or related care will be your responsibility.

ALTERNATIVES TO PARTICIPATION

You do not have to be in this study to receive treatment for your PNH. You have the option to receive standard treatment for PNH. The only curative treatment for this disorder is bone marrow transplant. Standard treatment to manage symptoms may include medications such as Eculizumab (Soliris®), prednisone, or blood thinners; blood transfusions. The study doctor will discuss with you the risks and benefits of the alternative treatments.

NEW FINDINGS

Any new important information that is discovered during the study, either from this study or from other studies which are ongoing in both subjects and healthy volunteers, and which may influence your willingness to continue participation in the study, will be made available to you.

Version Date: August 23, 2018

NOT FOR CONSENTING SUBJECTS
DRAFT 1 08/23/2018 JA

BENEFITS

You may or may not benefit from participation in this study. There is no guarantee that you will benefit from participating in this study. Information learned from this study may benefit others in the future.

COMPENSATION FOR PARTICIPATION

<<Site Specific Compensation language>>

CONFIDENTIALITY

Records of your participation in this study will be held confidential except when sharing the information is required by law or as described in this informed consent. The study doctor, the sponsor or persons working on behalf of the sponsor, and under certain circumstances, the United States Food and Drug Administration (FDA), Safety Monitoring Committee and the Institutional Review Board (IRB) will be able to inspect and copy confidential study-related records which identify you by name. This means that absolute confidentiality cannot be guaranteed. If the results of this study are published or presented at meetings, you will not be identified.

A description of this clinical trial will be available on http://www.ClinicalTrials.gov, as required by U.S. Law. This Web site will not include information that can identify you. At most, the Web site will include a summary of the results. You can search this Web site at any time.

COMPENSATION FOR INJURY

If you are injured as a result of taking the study drug or from procedures done for the purpose of this study, the sponsor will pay for those medical expenses necessary to treat your injury that are not covered by your medical insurance or any other third party coverage. There are no plans to provide other compensation beyond that which is listed in this informed consent document. You will not lose any of your legal rights or release the Sponsor, the study doctor, the study staff, or study site from liability for mistakes or intentional misconduct by signing this consent document.

If you are injured during this study, your study doctor will discuss with you the available medical treatment options.

COSTS

There will be no charge to you for your participation in this study. The study drug, vaccinations, antiviral/antibiotic, study-related procedures, and study visits will be provided at no charge to you or your insurance company.

NOT FOR CONSENTING SUBJECTS
DRAFT 1 08/23/2018 JA

EMERGENCY CONTACT / IRB CONTACT

During the study, if you experience any medical problems, suffer a research-related injury, or have questions, concerns or complaints about the study, please contact the study doctor at the telephone number listed on page one of this consent document. If you seek emergency care, or hospitalization is required, alert the treating physician that you are participating in a research study being conducted by the study doctor listed on page one of this document.

If you have any questions about your rights as a research subject, and/or concerns or complaints regarding this research study, you should write to Schulman IRB 4445 Lake Forest Drive - Suite 300, Cincinnati, Ohio 45242, or call toll-free 1-888-557-2472 during business hours Monday - Friday 8:00 a.m. to 6:00 p.m. EST. An institutional review board (IRB) is an independent committee established to help protect the rights of research subjects.

VOLUNTARY PARTICIPATION / WITHDRAWAL

Your decision to participate in this study is voluntary. You may choose to not participate or you may withdraw from the study for any reason without penalty or loss of benefits to which you are otherwise entitled and without any effect on your future medical care.

The study doctor or the sponsor can stop your participation at any time without your consent for the following reasons:

- If it appears to be medically harmful to you;
- If you fail to follow directions for participating in the study;
- If it is discovered that you do not meet the study requirements; or
- If the study is canceled.

If you leave the study for any reason, the study doctor may ask you to have some end-of-study tests for your safety.

Version Date: August 23, 2018

NOT FOR CONSENTING SUBJECTS
DRAFT 1 08/23/2018 JA

PRIMARY CARE PHYSICIAN / SPECIALIST NOTIFICATION OPTION

Please indicate below whether you want us to notify your primary care physician or your specialist of your participation in this study.

________ Yes, I want the study doctor to inform my primary care physician/specialist of my participation in this study.

________ No, I do not want the study doctor to inform my primary care physician/specialist of my participation in this study.

________ I do not have a primary care physician/specialist.

________ The study doctor is my primary care physician/specialist.

NOT FOR CONSENTING SUBJECTS
DRAFT 1 08/23/2018 JA

Version Date: August 23, 2018

CONSENT

I have read and understand the information in this informed consent document. I have had an opportunity to ask questions and all of my questions have been answered to my satisfaction. I voluntarily agree to participate in this study until I decide otherwise. I do not give up any of my legal rights by signing this consent document. I will receive a copy of this signed consent document.

If I am a male, I will advise my sexual partner(s) that I am participating in a research study of a drug and that the effects of the drug on an unborn baby and on a pregnant woman are unknown. I will provide my female sexual partner(s) with the information in the Pregnancy/Birth Control section of this Informed Consent and will provide her with contact information for the study doctor for any additional questions.

____

Subject's Printed Name

____________________________________ _________

Subject's Signature **Date**

____

Printed Name of the Person Conducting the Consent Discussion

_____________________________________ ___________

Signature of the Person Conducting the Consent Discussion Date

NOT FOR CONSENTING SUBJECTS
DRAFT 1 08/23/2018 JA

Version Date: August 23, 2018

CONSENT FOR SUBJECTS WHO CANNOT READ

The study subject has indicated that he/she is unable to read. The consent document has been read to the subject by a member of the study staff, discussed with the subject by a member of the study staff, and the subject has been given an opportunity to ask questions of the study staff.

____

Printed Name of Impartial Witness

____________________________________ __________

Signature of Impartial Witness* Date

*Impartial Witness: A person, who is independent of the trial, who cannot be unfairly influenced by people involved with the trial, who attends the informed consent process if the subject or the subject's legally acceptable representative cannot read, and who reads the informed consent and any other written information supplied to the subject. **Guidance for Industry E6 Good Clinical Practice: Consolidated Guidance.**

Version Date: August 23, 2018

NOT FOR CONSENTING SUBJECTS
DRAFT 1 08/23/2018 JA

AUTHORIZATION TO USE AND DISCLOSE PROTECTED HEALTH INFORMATION

During your participation in this research study, the study doctor and study staff will collect or create personal health information about you (for example, medical histories and results of any tests, examinations or procedures you undergo while in the study) and record it on study documents. The study doctor will keep this personal health information in your study-related records (that we will refer to as "your study records"). In addition, the study doctor may obtain, and include in your records, information regarding your past, present and/or future physical or mental health and/or condition. Your study doctor may ask you to sign a separate authorization to obtain some or all of your medical records from your doctor. Your study records may include other personal information (such as social security number, medical record numbers, date of birth, etc.), which could be used to identify you. Health information that could identify you is called "Protected Health Information" (or "PHI").

Under federal law (the "Privacy Rule"), your PHI that is created or obtained during this research study cannot be "used" to conduct the research or "disclosed" (given to anyone) for research purposes without your permission. This permission is called an "Authorization". Therefore, you may not participate in this study unless you give your permission to use and disclose your PHI by signing this Authorization. By signing, you are agreeing to allow the study doctor and staff to use your PHI to conduct this study.

By signing this Authorization, you also are agreeing to allow the study doctor to disclose PHI as described below:

- The sponsor of this study and anyone working on behalf of the sponsor to conduct this study (referred to as "the sponsor"). The sponsor will analyze and evaluate the PHI and may use it to develop new tests, procedures and commercial products. The study staff will assign a code number and/or letters to your records, which means that you will not ordinarily be identified in the records sent to the sponsor. The sponsor may, however, look at your complete study records that identify you. In addition, the sponsor may visit the study site to oversee the way the study is being conducted and may review your PHI during these visits to make sure the information is correct.
- The Institutional Review Board ("IRB") may have access to your PHI in relation to its responsibilities as an Institutional Review Board.

- Safety Monitoring Committee

The study doctor or sponsor may disclose your PHI to the United States Food and Drug Administration ("FDA") or similar regulatory agencies in the United States and/or foreign countries.

These disclosures also help ensure that the information related to the research is available to all parties who may need it for research purposes.

Except for the disclosures described above, your PHI will not be shared with others unless required by law. If your PHI is given to the parties listed above and/or to others who are not required to comply with the federal law, your PHI will no longer be protected by this law and could possibly be used or disclosed in ways other than those listed here.

You have a right to see and make copies of your PHI. You are agreeing, however, by signing this document, not to see or copy some or all of your PHI until the sponsor has completed all work related to this study. At that time, you may ask to see your records.

This Authorization will expire 50 years from the date you sign it unless you revoke (cancel or withdraw) it sooner.

You have a right to revoke your Authorization at any time. If you revoke it, your PHI will no longer be used for this study, except to the extent the parties to the research have already taken action based upon your Authorization or need the information to complete analysis and reports for this research. To revoke your Authorization, you must write to the study doctor, stating that you are revoking your Authorization to Use and Disclose Protected Health Information. If you revoke this Authorization, you will not be allowed to continue to be in this study.

NOT FOR CONSENTING SUBJECTS
DRAFT 1 08/23/2018 JA

Version Date: August 23, 2018

Printed Name of Subject

Signature of Subject Date

Printed Name of the Person Obtaining the Authorization

Signature of the Person Obtaining the Authorization Date

Version Date: August 23, 2018

NOT FOR CONSENTING SUBJECTS
DRAFT 1 08/23/2018 JA

FOR SUBJECTS WHO CANNOT READ

The study subject has indicated that he/she is unable to read. This Authorization document has been read to the subject by a member of the study staff, discussed with the subject by a member of the study staff, and the subject has been given an opportunity to ask questions of the study staff.

____

Printed Name of Impartial Witness

____________________________________ __________

Signature of Impartial Witness* Date

*Impartial Witness: A person, who is independent of the trial, who cannot be unfairly influenced by people involved with the trial, who attends the informed consent process if the subject or the subject's legally acceptable representative cannot read, and who reads the informed consent and any other written information supplied to the subject. **Guidance for Industry E6 Good Clinical Practice: Consolidated Guidance.**

16.1.3.3 Written Subject Information

Not applicable.
