# Supplementary material for: Improvements in hematologic markers and decreases in fatigue with pegcetacoplan for patients with paroxysmal nocturnal hemoglobinuria and mild or moderate anemia (hemoglobin ≥10 g/dL) who had received eculizumab or were naive to complement inhibitors
Source: PLoS One. 2024 Jul 29;19(7):e0306407. doi: 10.1371/journal.pone.0306407 (PMC11285951; doi:10.1371/journal.pone.0306407)
Supplement: S6 File — De-identified patient data. S1 Fig A. Hemoglobin concentrations (baseline vs. 16 weeks of pegcetacoplan) for patients with PNH and mild/moderate anemia (hemoglobin ≥10 g/dL) in the PADDOCK, PRINCE, and PEGASUS trials. S1 Fig B. ARCs (baseline vs. 16 weeks of pegcetacoplan) for patients with PNH and mild/moderate anemia (hemoglobin ≥10 g/dL) in the PADDOCK, PRINCE, and PEGASUS trials. S1 Fig C. LDH concentrations (baseline vs. 16 weeks of pegcetacoplan) for patients with PNH and mild/moderate anemia (hemoglobin ≥10 g/dL) in the PADDOCK, PRINCE, and PEGASUS trials. S1 Fig D. FACIT-Fatigue score analysis (baseline vs. 16 weeks of pegcetacoplan) for patients with PNH and mild/moderate anemia (hemoglobin ≥10 g/dL) in the PADDOCK, PRINCE, and PEGASUS trials. (PDF) [file pone.0306407.s006.pdf]

Supplement

Improvements in hematologic markers and decreases in fatigue with pegcetacoplan for patients with paroxysmal nocturnal hemoglobinuria and mild or moderate anemia (hemoglobin ≥10 g/dL) who had received eculizumab or were naive to complement inhibitors

Jens Panse, MD[1,2*], Nicolas Daguindau, MD[3], Sonia Okuyama, MD[4], Régis Peffault de Latour, MD, PhD[5], Philippe Schafhausen, MD[6], Nicole Straetmans, MD, PhD[7], Mohammed Al-Adhami, PhD[8], Emmelie Persson, PhD[9], Raymond Siu Ming Wong, MBChB, MD[10]

[1]Department of Hematology, Oncology, Hemostaseology and Stem Cell Transplantation, University Hospital RWTH Aachen, Aachen, Germany; [2]Center for Integrated Oncology (CIO), Aachen, Bonn, Cologne, Dusseldorf (ABCD), Germany; [3]Centre Hospitalier Annecy Genevois, Épagny-Metz-Tessy, France; [4]Hematology/Oncology Division, Denver Health and Hospital Authority, Denver, CO, USA; [5]French Reference Center for Aplastic Anemia and Paroxysmal Nocturnal Hemoglobinuria, Assistance Publique - Hôpitaux de Paris; Université de Paris, Paris, France; [6]Department of Oncology, Hematology, and Bone Marrow Transplantation with Section of Pneumology, University Medical Center Hamburg-Eppendorf, Hamburg; [7]Department of Hematology, Cliniques Universitaires Saint-Luc, Woluwe-Saint-Lambert, Belgium; [8]Apellis Pharmaceuticals, Waltham, MA, USA; [9]Swedish Orphan Biovitrum AB, Stockholm, Sweden; [10]Sir Y.K. Pao Centre for Cancer & Department of Medicine and Therapeutics, Prince of Wales Hospital, The Chinese University of Hong Kong, Hong Kong

Table of Contents

S1 Table. De-identified patient data 3

S1 Fig A. Hemoglobin concentrations (baseline vs. 16 weeks of pegcetacoplan) for patients with PNH and mild/moderate anemia (hemoglobin ≥10 g/dL) in the PADDOCK, PRINCE, and PEGASUS trials 4

S1 Fig B. ARCs (baseline vs. 16 weeks of pegcetacoplan) for patients with PNH and mild/moderate anemia (hemoglobin ≥10 g/dL) in the PADDOCK, PRINCE, and PEGASUS trials 5

S1 Fig C. LDH concentrations (baseline vs. 16 weeks of pegcetacoplan) for patients with PNH and mild/moderate anemia (hemoglobin ≥10 g/dL) in the PADDOCK, PRINCE, and PEGASUS trials 6

S1 Fig D. FACIT-Fatigue score analysis (baseline vs. 16 weeks of pegcetacoplan) for patients with PNH and mild/moderate anemia (hemoglobin ≥10 g/dL) in the PADDOCK, PRINCE, and PEGASUS trials 7

Supplement reference 8

S1 Table. De-identified patient data.

#	Trial	Sex	Transfusion	Hb-BL	Hb-W2	Hb-W4	Hb-W6	Hb-W8	Hb-W12	Hb-W16	ARC-BL	ARC-W2	ARC-W4	ARC-W6	ARC-W8	ARC-W12	ARC-W16	LDH-BL	LDH-W2	LDH-W4	LDH-W6	LDH-W8	LDH-W12	LDH-W16	FACIT-F-BL	FACIT-F-W2	FACIT-F-W4	FACIT-F-W6	FACIT-F-W8	FACIT-F-W12	FACIT-F-W16
1	PADDOCK	M	NA*	11.0	11.4	11.9	NA	NA	NA	NA	192	96	89	NA	NA	NA	NA	1681	251	175	NA	NA	NA	NA	27	40	47	NA	NA	NA	NA
2	PADDOCK	F	N	10.0	11.6	12.2	12.6	11.9	12.5	11.4	123	232	100	98	112	135	164	2989	348	222	226	278	335	338	34	29	32	39	NA	37	38
3	PADDOCK	F	N	10.1	11.7	12.9	13.1	13.5	12.4	12.7	322	106	88	76	78	NA	110	2301	287	239	191	221	235	209	46	41	46	45	NA	43	48
4	PADDOCK	M	N	10.8	12.2	12.8	13.3	NA	13.2	13.5	183	57	40	45	NA	77	108	1579	184	140	128	NA	179	198	28	44	36	40	NA	43	43
5	PADDOCK	F	N	10.8	11.8	11.3	11.3	11.6	11.4	11.9	138	56	66	69	80	86	87	1199	205	128	162	151	188	223	43	47	45	47	NA	46	46
6	PADDOCK	M	N	10.4	12	12.4	13.1	13.2	13.8	14.2	235	122	110	95	100	117	109	1866	260	179	180	190	280	246	42	42	44	49	NA	51	51
7	PRINCE	M	N	10.6	12.2	12	13.1	13.9	14.2	14.3	275	90	80	70	50	80	90	2260.5	190	99	120	120	148	196	44	NA	36	NA	51	52	49
8	PRINCE	M	N	10.2	11.6	12.2	13.1	12.7	12.4	13.1	185	60	70	70	70	80	NA	1534.5	248	151	147	151	167	179.5	49	NA	50	NA	43	40	NA
9	PRINCE	M	N	11.3	NA	12.6	14.6	14	14.6	13.3	255	NA	NA	NA	NA	NA	60	2201.5	NA	181.4	251.5	NA	181.4	121	28	NA	NA	NA	NA	NA	46
10	PRINCE	F	N	10.5	12.2	12.1	11.9	11.9	12.1	11.7	290	150	100	150	130	130	100	1390	165	141	171	221	215.7	208	NA	NA	45	NA	42	50	50
11	PRINCE	M	N	12	14.4	16	15.2	14.3	14.9	14.8	295	70	50	40	70	150	110	2677	209	149	157	170	212	149	39	NA	45	NA	44	44	47
12	PRINCE	M	N	11.3	14.1	15.3	15.6	15.6	14.7	14.5	285	70	30	70	40	110	70	2777.5	260	150	154	167	211	213	19	NA	29	NA	30	24	31
13	PRINCE	F	N	11.3	12.9	13.4	14.4	15.1	14.9	14.4	163.3	100	50	60	50	90	110	1409.5	283	155	144	149	192	219	39	NA	51	NA	50	49	51
14	PRINCE	M	N	13.1	12.2	13.2	13.6	NA	15.1	16.1	180	70	60	50	NA	NA	NA	2974	157	120	132	157	182	175.9	26	NA	47	NA	51	49	50
15	PEGASUS	F	Y	10.3	12.2	11.8	12.2	NA	12.9	6.0	190	160	170	110	NA	NA	NA	258	149	535	393	NA	180	NA	31	42	41	48	NA	39	NA
16	PEGASUS	F	N	10.0	11.4	11.6	11.1	11.3	11.6	11.4	160	60	80	80	80	70	50	227.5	177	195	186	187	206	199	19	37	32	35	31	38	44
17	PEGASUS	F	N	10.2	14.2	14.1	13.9	13.1	13.6	13.9	205	20	30	30	60	70	50	171	119	122	121	166	115	111	30	46	48	35	48	42	39
18	PEGASUS	M	N	10.1	12	12.8	13.4	13.3	13.3	13.4	390	80	50	100	90	100	100	211	130	115	146	145	161	169	36	47	49	49	51	51	51
19	PEGASUS	F	N	10.0	13.1	12.8	13.3	12.8	12.5	12.3	260	80	110	120	70	120	100	180.5	127	141	136	157	138	156	16	40	38	36	45	42	44
20	PEGASUS	M	N	10.8	15.2	14.4	14.8	14.8	14.7	14.4	420	120	150	130	120	110	100	286	229	231	189	211	199	190	19	39	35	37	39	44	42
21	PEGASUS	M	N	10.1	14	14.1	13.5	14	14.4	14.4	290	80	100	70	70	70	80	194.5	154	177	174	177	157	159	29	40	37	39	40	43	42
22	PEGASUS	M	Y	10.0	13.9	6.6	NA	NA	NA	NA	350	50	NA	NA	NA	NA	NA	210	113	NA	NA	NA	NA	NA	39	47	NA	NA	9	NA	NA
23	PEGASUS	F	N	10.4	13.2	12.9	12.6	11.1	12.3	13.2	250	40	60	40	80	100	70	132.7	74	76	75	70	90	86	18	38	27	13	31	33	27
24	PEGASUS	F	N	10.2	12.4	13.8	14.4	13.2	14.1	13.3	200	50	30	60	40	70	80	204	639	161	149	166	191	175	41	43	51	50	52	52	52
25	PEGASUS	M	N	10.1	14.6	16.1	15.6	15.7	15.3	15.3	200	70	90	70	110	110	110	155	117	112	171	143	141	149	34	36	44	24	43	42	43

ARC, absolute reticulocyte count; BL, baseline; F, female; FACIT-F, Functional Assessment of Chronic Illness Therapy-Fatigue; Hb, hemoglobin; LDH, lactate dehydrogenase; M, male; NA, not available; W, week.

*This patient was excluded from normalization analysis.

S1 Fig A. Hemoglobin concentrations (baseline vs. 16 weeks of pegcetacoplan) for patients with PNH and mild/moderate anemia (hemoglobin ≥10 g/dL) in the PADDOCK, PRINCE, and PEGASUS

trials. The top and bottom edges of the boxes indicate the upper and lower values of the range for patients in this analysis. Horizontal black lines in the boxes indicate mean values of the patients in this analysis, and the diamonds indicate the mean value for all pegcetacoplan-treated patients in each study. Individual values for patients in this study are shown by circles.

LLN, lower limit of normal; PNH, paroxysmal nocturnal hemoglobinuria.

[a]This analysis was performed using week 16 as the baseline for patients who received eculizumab.

[b]Data are missing for 1 patient who stopped dosing at day 29 and left the study per physician decision.

[c]Excludes 1 patient with missing data at this timepoint. This patient discontinued after week 8 of the randomized controlled period.

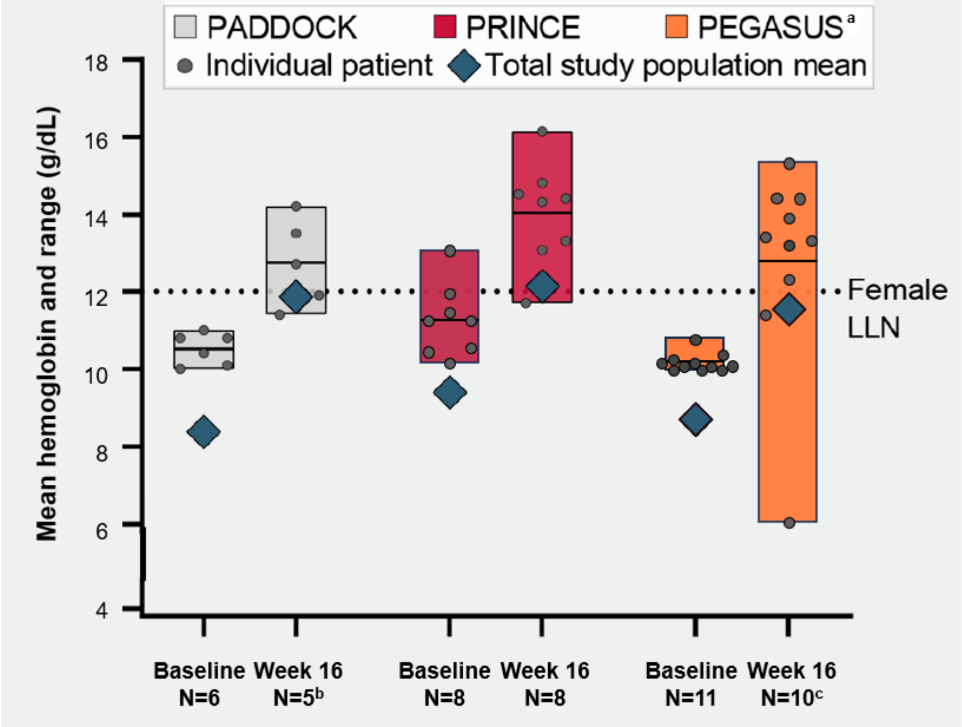

S1 Fig B. ARCs (baseline vs. 16 weeks of pegcetacoplan) for patients with PNH and mild/ moderate anemia (hemoglobin ≥10 g/dL) in the PADDOCK, PRINCE, and PEGASUS trials. The top and bottom edges of the boxes indicate the upper and lower values of the range for patients in this analysis. Horizontal black lines in the boxes indicate mean values of the patients in this analysis, and the diamonds indicate the mean value for all pegcetacoplan-treated patients in each study. Individual values for patients in this study are shown by circles.

ARC, absolute reticulocyte count; LLN, lower limit of normal; PNH, paroxysmal nocturnal hemoglobinuria; ULN, upper limit of normal.

[a]This analysis was performed using week 16 as the baseline for patients who received eculizumab.

[b]Data are missing for 1 patient who stopped dosing at day 29 and left the study per physician decision.

[c]Excludes 2 patients with missing ARC data at this timepoint.

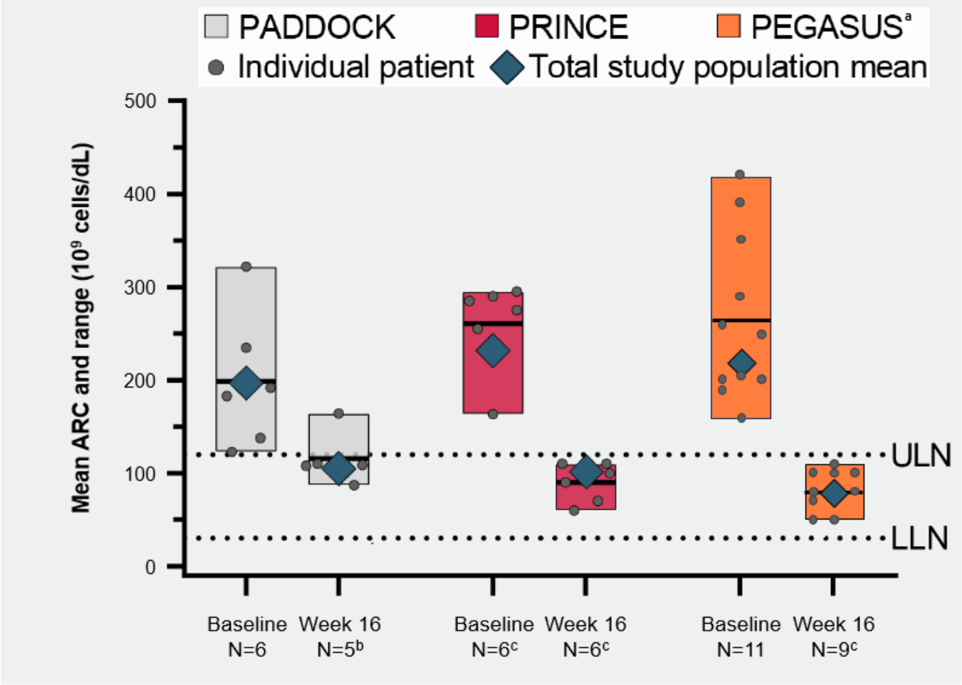

S1 Fig C. LDH concentrations (baseline vs. 16 weeks of pegcetacoplan) for patients with PNH and mild/moderate anemia (hemoglobin ≥10 g/dL) in the PADDOCK, PRINCE, and PEGASUS trials. The top and bottom edges of the boxes indicate the upper and lower values of the range for patients in this analysis. Horizontal black lines in the boxes indicate mean values of the patients in this analysis, and the diamonds indicate the mean value for all pegcetacoplan-treated patients in each study. Individual values for patients in this study are shown by circles.

LDH, lactate dehydrogenase; LLN, lower limit of normal; PNH, paroxysmal nocturnal hemoglobinuria; ULN, upper limit of normal.

[a]This analysis was performed using week 16 as the baseline for patients who received eculizumab.

[b]Data are missing for 1 patient who stopped dosing at day 29 and left the study per physician decision.

[c]Excludes 2 patients with missing data at this timepoint.

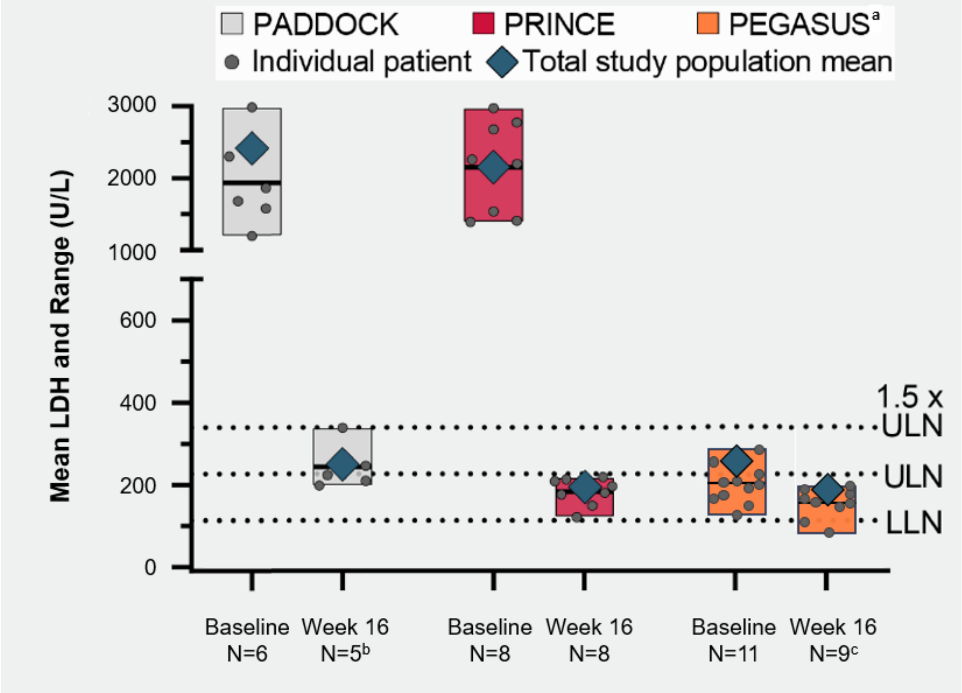

S1 Fig D. FACIT-Fatigue score analysis (baseline vs. 16 weeks of pegcetacoplan) for patients with PNH and mild/moderate anemia (hemoglobin ≥10 g/dL) in the PADDOCK, PRINCE, and PEGASUS trials. Mean FACIT-Fatigue scores at baseline and after 16 weeks of pegcetacoplan treatment are shown for patients with PNH and mild or moderate anemia (hemoglobin ≥10.0 g/dL) in the PADDOCK, PRINCE, and PEGASUS trials. The top and bottom edges of the boxes indicate the upper and lower values of the range for patients in this analysis. Horizontal black lines in the boxes indicate mean values of the patients in this analysis, and the diamonds indicate the mean value for all pegcetacoplan-treated patients in each study. Individual values for patients in this study are shown by circles.

FACIT-Fatigue, Functional Assessment of Chronic Illness Therapy-Fatigue; PNH, paroxysmal nocturnal hemoglobinuria.

[a]This analysis was performed using week 16 as the baseline for patients who received eculizumab.

[b]Defined by Cella et al [1].

[c]Data are missing for 1 patient who stopped dosing at day 29 and left the study per physician decision.

[d]Data were collected at day 141.

[e]Excludes 1 patient with missing FACIT-Fatigue data at this timepoint.

[f]Excludes 2 patients with missing FACIT-Fatigue data at this timepoint.

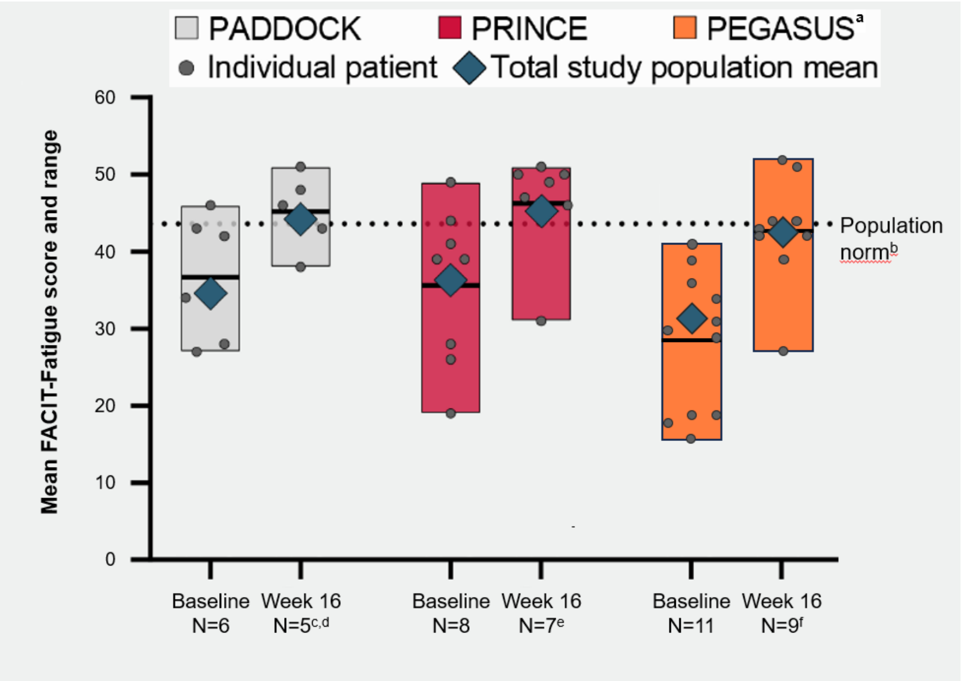

SUPPLEMENT REFERENCE

1. Cella D, Lai J-S, Chang C-H, Peterman A, Slavin M. Fatigue in cancer patients compared with fatigue in the general United States population. Cancer. 2002;94(2): 528-538. doi: 10.1002/cncr.10245.
